# Comparing Non-invasive Inverse Electrocardiography With Invasive Endocardial and Epicardial Electroanatomical Mapping During Sinus Rhythm

**DOI:** 10.3389/fphys.2021.730736

**Published:** 2021-10-04

**Authors:** Robert W. Roudijk, Machteld J. Boonstra, Rolf Brummel, Wil Kassenberg, Lennart J. Blom, Thom F. Oostendorp, Anneline S. J. M. te Riele, Jeroen F. van der Heijden, Folkert W. Asselbergs, Peter M. van Dam, Peter Loh

**Affiliations:** ^1^Division Heart and Lungs, Department of Cardiology, University Medical Center Utrecht, Utrecht University, Utrecht, Netherlands; ^2^Radboud University Nijmegen Medical Centre, Donders Institute for Brain, Cognition and Behaviour, Nijmegen, Netherlands; ^3^Faculty of Population Health Sciences, Institute of Cardiovascular Science, University College London, London, United Kingdom; ^4^Health Data Research UK, Institute of Health Informatics, University College London, London, United Kingdom; ^5^ECG Excellence BV, Nieuwerbrug, Netherlands

**Keywords:** inverse problem of electrocardiography, sudden cardiac death, electrocardiographic imaging (ECGI), equivalent dipole layer, cardiac arrhythmia, electroanatomical mapping, non-invasive mapping

## Abstract

This study presents a novel non-invasive equivalent dipole layer (EDL) based inverse electrocardiography (*i*ECG) technique which estimates both endocardial and epicardial ventricular activation sequences. We aimed to quantitatively compare our *i*ECG approach with invasive electro-anatomical mapping (EAM) during sinus rhythm with the objective of enabling functional substrate imaging and sudden cardiac death risk stratification in patients with cardiomyopathy. Thirteen patients (77% males, 48 ± 20 years old) referred for endocardial and epicardial EAM underwent 67-electrode body surface potential mapping and CT imaging. The EDL-based *i*ECG approach was improved by mimicking the effects of the His-Purkinje system on ventricular activation. EAM local activation timing (LAT) maps were compared with *i*ECG-LAT maps using absolute differences and Pearson’s correlation coefficient, reported as mean ± standard deviation [95% confidence interval]. The correlation coefficient between *i*ECG-LAT maps and EAM was 0.54 ± 0.19 [0.49–0.59] for epicardial activation, 0.50 ± 0.27 [0.41–0.58] for right ventricular endocardial activation and 0.44 ± 0.29 [0.32–0.56] for left ventricular endocardial activation. The absolute difference in timing between *i*ECG maps and EAM was 17.4 ± 7.2 ms for epicardial maps, 19.5 ± 7.7 ms for right ventricular endocardial maps, 27.9 ± 8.7 ms for left ventricular endocardial maps. The absolute distance between right ventricular endocardial breakthrough sites was 30 ± 16 mm and 31 ± 17 mm for the left ventricle. The absolute distance for latest epicardial activation was median 12.8 [IQR: 2.9–29.3] mm. This first in-human quantitative comparison of *i*ECG and invasive LAT-maps on both the endocardial and epicardial surface during sinus rhythm showed improved agreement, although with considerable absolute difference and moderate correlation coefficient. Non-invasive *i*ECG requires further refinements to facilitate clinical implementation and risk stratification.

## Introduction

Non-invasive imaging of cardiac depolarization and repolarization sequences, known as electrocardiographic imaging, is based on body surface potentials maps and cardiovascular imaging (Huiskamp and Van Oosterom, 1988; Ramanathan et al., 2004; van Dam et al., 2009; Rudy, 2013). Two major methods have been introduced: (1) the potential based Equivalent Potential Distribution (EPD) method (Ramanathan et al., 2004; Sapp et al., 2012; Rudy, 2013; Cluitmans et al., 2017; Duchateau et al., 2019; Graham et al., 2019; Hohmann et al., 2019), which estimates electrograms on the epicardium in a linear relation whereof activation and recovery timings are determined on the epicardium, and (2) the wave-front formulation based on the equivalent dipole layer (EDL) (Huiskamp and Van Oosterom, 1988; van Dam et al., 2009; van Oosterom, 2014; Oosterhoff et al., 2016). The EDL-based method, used in this study and referred to as inverse electrocardiography (*i*ECG), calculates transmembrane potentials at both the endocardium and epicardium as a local source, whereof activation and recovery times are derived (van Dam et al., 2009; van Oosterom, 2014). More precisely, these transmembrane potentials represented in the EDL-based method create currents that are proportional to the second derivative of the local transmembrane potentials (Leon and Witkowski, 1995). Since the relation between the transmembrane potentials and the body surface potential map is non-linear, an initial estimation of the activation sequence is required (Huiskamp and Van Oosterom, 1988; van Dam et al., 2009; Oosterhoff et al., 2016).

The implementation of electrocardiographic imaging in clinical practice is limited, which may partly be explained by poor results for estimations during sinus rhythm (Cluitmans et al., 2018). Whereas estimation of rhythms with a single ventricular focus, i.e., ventricular pacing or premature ventricular complexes, is promising (Rudy, 2013; Oosterhoff et al., 2016; Cluitmans et al., 2018; Duchateau et al., 2019; Graham et al., 2019; Hohmann et al., 2019). Estimation of ventricular activation during sinus rhythm is complicated by the nearly simultaneous initiation of activation waves from multiple endocardial sites mediated by the His-Purkinje system (Durrer et al., 1970). Quantitative comparison studies during sinus rhythm are limited and have shown poor performance, represented by low correlation coefficients between non-invasive estimations and invasive mapping (Duchateau et al., 2019).

The proposed *i*ECG method mimics the effects of the His-Purkinje system on the initiation of ventricular activation waves to improve accuracy of estimation during sinus rhythm (van Dam et al., 2009; Sapp et al., 2012; Rudy, 2013; van Oosterom, 2014; Cluitmans et al., 2017; Duchateau et al., 2019; Graham et al., 2019). With improved accuracy of estimation during sinus rhythm, *i*ECG techniques may enable functional imaging of electro-anatomical substrates on both the epicardium and endocardium and aid early detection and non-invasive risk stratification of patients with cardiomyopathies (Tung et al., 2020). Therefore, a quantitative comparison of this novel *i*ECG method for estimation of ventricular activation during sinus rhythm was performed. In this study, invasive endocardial and epicardial high-resolution local activation timing (LAT) maps obtained during electro-anatomical mapping (EAM) were compared to non-invasively estimated activation patterns.

## Materials and Methods

### Patient Population

Patients referred for endocardial and epicardial EAM and ablation were enrolled. Epicardial mapping was indicated because of either recurrent ventricular tachycardia with a suspected epicardial substrate or symptomatic premature ventricular complexes with a prior failed endocardial ablation. Anti-arrhythmic drugs, except amiodarone, were discontinued for a minimum of three half-lives prior to the ablation procedure. Amiodarone was continued because of its long half-life. The study protocol was approved by the local institutional review board (University Medical Center Utrecht, Utrecht, Netherlands; protocol nr.17/628). The study was conducted according to the declaration of Helsinki and all patients gave informed consent prior to non-invasive and invasive mapping.

### Data Acquisition

**FIGURE 1:**
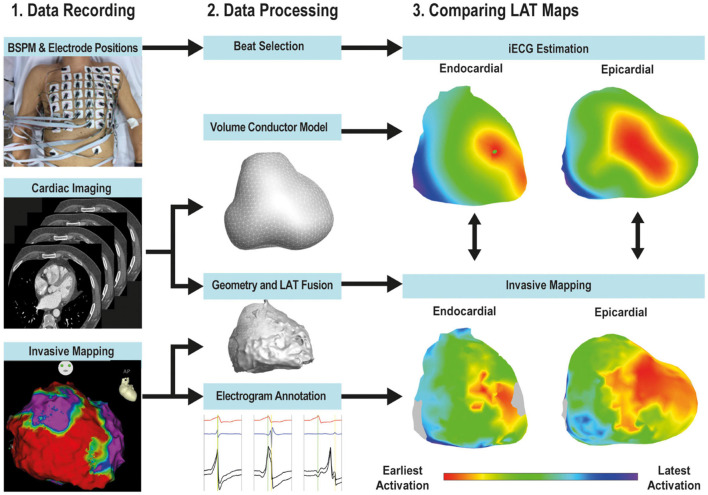
Workflow. The workflow of the study consisted of data recording (left panel), data processing (middle panel) and quantitative comparison (right panel). Body surface potential mapping (BSPM) using 67-electrodes was performed. CT imaging of the thorax and cardiac anatomy was performed and used to construct patient specific anatomical models and compute the volume conductor. The EAM anatomical point clouds were registered to the CT-based ventricular anatomy and LAT and bipolar values were projected on the CT-based anatomy. EAM-LAT maps were quantitatively compared to *i*ECG-LAT maps.

The workflow of the study is depicted in Figure 1. Patients underwent 67-electrode body surface potential mapping (sampling frequency 2048 Hz, Biosemi, Amsterdam, Netherlands) prior to the invasive mapping procedure and the electrode positions were captured using a 3-dimensional camera (Intel Realsense D435, Santa Clara, CA, United States) (Hoekema et al., 1999). Per patient, cardiac computed tomography (CT, Philips Healthcare, Best, Netherlands) was performed to manually create patient specific anatomical models of the ventricles with both epicardial and endocardial surfaces, ventricular blood pool, lungs and thorax. The ventricular anatomical models were supplemented with patient specific endocardial structures associated with early ventricular activation through the His-Purkinje system (e.g., the left ventricular papillary muscles and right ventricular moderator band) (Durrer et al., 1970). Electrode positions were reconstructed by registering 3-dimensional images to the thorax model. The volume conductor model was computed using the boundary element method. Conductivity values of 0.2 S/m for the thorax and ventricular tissue, 0.04 S/m for the lungs and 0.6 S/m for the blood cavities were used (Supplementary Methods).

### Signal Processing

Baseline drift and 50 Hz noise were removed from the body surface potential map signals. Per patient, five subsequent sinus rhythm complexes were selected to be analyzed in the *i*ECG procedure. Premature ventricular complexes and sinus rhythm complexes prior to premature ventricular complexes were excluded from analysis. The root mean square of all recorded signals was used to annotate QRS onset, J-point and T-wave end. One lead from the standard 12-lead ECG was used as timing reference to allow comparison of absolute timings between *i*ECG estimations and invasive EAM timings.

### Inverse Electrocardiographic Imaging Procedure

The novel *i*ECG method has been described in more detail in the Supplementary Methods (Greensite et al., 1990; Huiskamp and Greensite, 1997; van Dam et al., 2009; van Oosterom, 2014). In short: the *i*ECG method simulates body surface potential maps using the patient specific EDL cardiac source model, the patient specific volume conductor model and the estimated ventricular activation sequence. Nine regions containing potential foci were localized: four at the left ventricular septum, two at the base of both the posterior and anterior papillary muscles of the left ventricle, two at the right ventricular septum and one at the insertion of the moderator band at the right ventricular free wall free wall (Durrer et al., 1970). The fastest route algorithm was used to compute activation sequences emerging from these locations and combinations of foci (van Dam et al., 2009). All possible combinations of foci were tested as the initial estimation (Supplementary Methods). The activation sequence from the initial estimation with the highest correlation between the simulated body surface potential map and the recorded body surface potential map, was selected as input for the optimization step (Supplementary Methods) (van Dam et al., 2009). The optimized activation sequence was used to assign LAT to each node in the patient specific ventricular anatomical model (Figure 1 and Supplementary Figure 1).

### Invasive Electro-Anatomical Mapping

**FIGURE 2:**
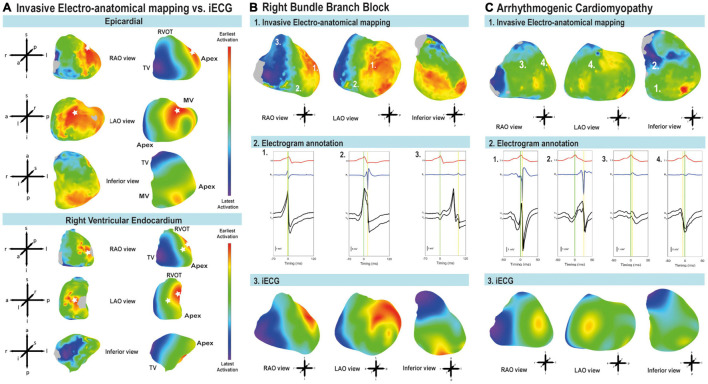
Comparison of early and latest activated myocardium and annotation of local activation timing. **(A)** LAT maps derived from iECG and EAM from early (red) to late (blue) activation. Breakthroughs of ventricular activation are indicated with a white asterisk. Epicardium: both EAM and iECG estimation showed breakthrough of activation at the right ventricular free wall and the left ventricular free wall. Endocardial activation of the right ventricular free wall: iECG estimation corresponds to EAM, intraventricular septum activation was located more toward the apex in the EAM. Imaging views are based on the anatomical approach for EAM (Cosio et al., 1999). MV, mitral valve; RVOT, right ventricular outflow tract; TV, tricuspid valve. **(B)** Patient with healed myocarditis and right bundle branch block. Imaging views are based on the anatomical approach for EAM (Cosio et al., 1999). 1: Epicardial EAM-LAT map from early (red) to late (blue) activation. The early regions in the left ventricle and late regions in the right ventricle suggest a right bundle branch block. 2: EGM annotation of bipolar electrograms. The green line corresponds to the timing reference. The yellow line shows annotation to the maximal amplitude of the bipolar signal. 3: *i*ECG-LAT map of epicardial activation from early (red) to late (blue) activation. **(C)** Epicardial activation and electrogram annotation in a patient with arrhythmogenic cardiomyopathy. Imaging views are based on the anatomical approach for EAM (Cosio et al., 1999). 1: EAM-LAT maps from early (red) to latest (blue) activation. 2: Electrogram annotation of bipolar signals. The green line corresponds to the timing reference. The yellow line shows annotation to the maximal amplitude of the bipolar signal. 3: *i*ECG-LAT map from early (red) to late (blue) activation.

Invasive EAM was performed under general anesthesia during sinus rhythm or atrial pacing. Ventricular paced complexes and premature ventricular complexes were excluded from analysis. Epicardial access was obtained by percutaneous subxiphoid approach (Sosa et al., 1996) and endocardial access was obtained through the right femoral vein. Access to the left ventricle was gained through a transseptal puncture, using a steerable sheath (Mobicath, Biosense-Webster Inc. Irvine, CA, United States). Anatomical coordinates, LAT maps and voltage maps were automatically created with EAM systems (Carto-3, Biosense-Webster Inc. Irvine, CA, United States or EnSite Precision, Abbott, Chicago, IL, United States) without prior integration of cardiac CT images. Endocardial and epicardial EAM was performed with multi-electrode catheters (PENTARAY^®^ catheter, Biosense-Webster Inc. Irvine, United States or ADVISOR^TM^ HD Grid mapping Catheter, Abbott, Chicago, Il, United States). Unipolar and bipolar electrograms were simultaneously recorded with standard 12-lead ECG (band pass filters 30–500 Hz, sampling frequency 1000 Hz), and one of these leads was used as timing reference for electrograms. Post-procedure, bipolar and corresponding unipolar electrograms were manually reviewed by investigators who were blinded to the information from the corresponding *i*ECG map. LAT was set at the maximal amplitude of the bipolar signal, corresponding to maximum downslope (dV/dt) in unipolar electrograms (see Figure 2 for examples) (Cantwell et al., 2015). In case of doubt, recordings from neighboring electrograms were taken into consideration to determine LAT. Epicardial and endocardial myocardium with abnormal voltage electrograms was defined as bipolar voltage amplitude < 1.5 mV.

### Comparison of Non-invasive Mapping and Invasive Mapping

Anatomical coordinates with corresponding annotated LAT and bipolar voltage, obtained during EAM, were exported (MATLAB-2017a, The Mathworks Inc, Natick, United States). These anatomical coordinates were semi-automatically aligned to the CT-based ventricular anatomical model, according to anatomical landmarks (right ventricular outflow tract and the apex of the ventricles, Figure 1). Endocardial alignment was optimized using a closest point matching algorithm (Bergquist et al., 2019). Surface Laplacian interpolation was used for areas with incomplete EAM, within a distance of 10 mm. To reduce misalignment errors, invasively collected datapoints for myocardial surfaces were projected onto the nearest node of the CT-based model and all projections per node were averaged. *i*ECG-LAT maps were referenced to the same timing reference used during the EAM procedure. Pearson’s inter-map correlation coefficient and inter-map absolute difference in milliseconds (ms) were determined for the epicardium, right ventricular endocardium and left ventricular endocardium. Breakthrough of activation was defined as nodes with the lowest LAT value, and sites of latest activation were defined as the node with the highest LAT value. Euclidian distances between sites of earliest and latest activation were determined in millimeters (mm). Myocardial conduction velocity over surfaces was calculated as the minimum positive velocity between nodes, velocities more than 3 mm/ms were excluded. A relatively high cut-off of 3 mm/ms was used to account for velocities observed in regions with a high density of Purkinje-myocardial junctions as the conduction velocity of Purkinje fibers ranges between 2 and 3 mm/ms. This cut-off was used to take into account that the electrical pulse may spread via the Purkinje fibers to the neighboring myocardial tissue instead of via the myocardial tissue itself. Ventricular activation sequences were presented in right anterior oblique, left anterior oblique and inferior views (Cosio et al., 1999).

### Statistical Analysis

Data were presented as mean ± standard deviation or median [interquartile range], supplemented with 95% confidence interval (CI). Continuous data were compared using (un)paired Student’s *t*-test or Mann–Whitney *U* test as appropriate. Differences between *i*ECG*-*LAT maps and EAM-LAT maps were presented as absolute difference in ms for timings or absolute difference in mm for differences in sites of breakthrough, earliest activation or latest activation. *i*ECG-LAT and EAM-LAT maps were compared with Pearson’s linear correlation and presented as correlation coefficient. Agreement between *i*ECG and EAM-LAT timings was quantitatively compared by Bland-Altman plots. A 2-sided *P*-value of <0.05 was considered significant. Statistical analysis was performed in MATLAB (MATLAB 2017a, The Mathworks Inc, Natick, MA, United States).

## Results

### Study Population

Thirteen patients (77% males, age 48 ± 20 years) referred for epicardial and endocardial mapping and ablation of ventricular tachycardia (*n* = 10) or symptomatic premature ventricular complexes (*n* = 3) were included. Patients were diagnosed with arrhythmogenic cardiomyopathy (*n* = 5), dilated cardiomyopathy (*n* = 2), symptomatic premature ventricular complexes (*n* = 3), or ventricular arrhythmias after healed myocarditis (*n* = 3). Patients had either sinus rhythm (*n* = 10) or atrial pacing by an implanted permanent pacemaker (*n* = 3) during body surface potential recording and the EAM procedure, see Supplementary Table 1 for a summary of the included population and Supplementary Table 2 for a detailed description per included patient.

### Electrocardiographic Imaging Procedure Quality

The patient cardiac anatomical models had an inter-node spatial resolution of 8 ± 1 mm. The QRS complex morphology of the recorded body surface potential maps correlated with the QRS complex morphology of the simulated body surface potential maps in the *i*ECG procedure (correlation coefficient = 0.97 ± 0.02). The QRS morphology of the timing reference lead during EAM correlated with the timing reference lead of the recorded body surface potential map (correlation coefficient = 0.94 ± 0.02).

### Electro-Anatomical Mapping Quality

Epicardial EAM was performed in all patients, right ventricular endocardial EAM in 10 patients and left ventricular endocardial EAM in four patients. EAM consisted of median 4611 [3369–5633] epicardial electrograms, 910 [280–1638] right ventricular endocardial electrograms and 605 [247–1412] left ventricular endocardial electrograms. The number of annotations per square mm was 20 ± 11 for the epicardium, 10 ± 5 for the right ventricular endocardium and 8 ± 4 for the left ventricular endocardium. The percentage of EAM per surface was on average 67 [range: 48–82]% of anatomical nodes for the epicardium, 45 [range: 15–79]% for the right ventricular endocardium and 48 [range: 22–71]% for the left ventricular endocardium. The anatomical EAM model was limited to the locations where the catheter had been positioned during the EAM procedure.

### Local Activation Timing

**FIGURE 3:**
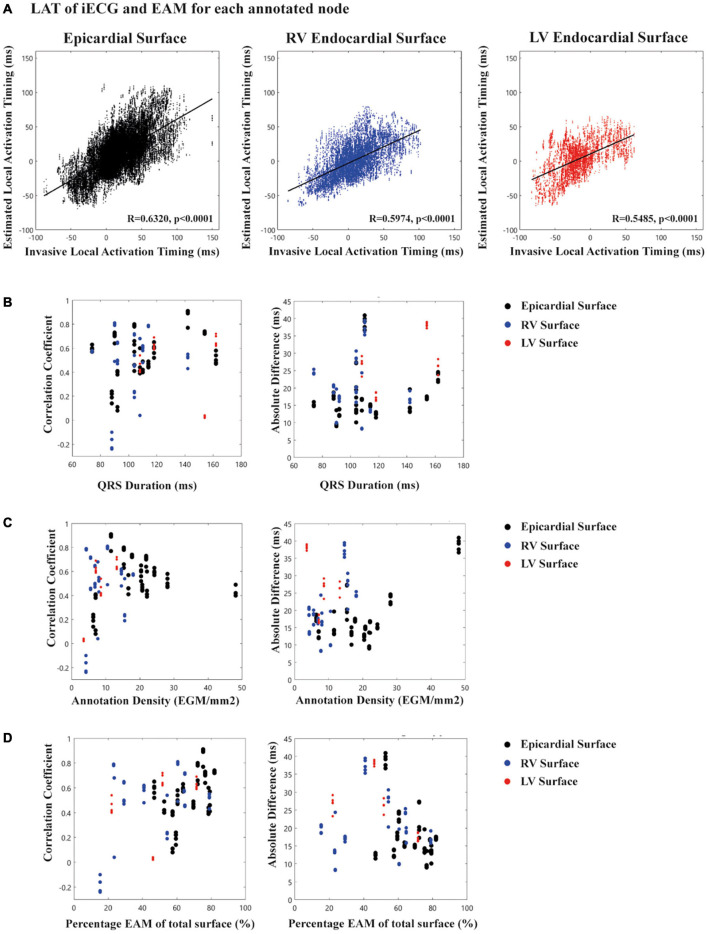
Scatter plots of local activation timing stratified for epicardial and endocardial surfaces. **(A)** For each node in the ventricular anatomy the EAM-LAT values (*X*-axis) are scattered against *i*ECG-LAT values (*Y*-axis). The black line in each plot represents the linear regression line and R-value and *p*-value are shown in each plot. **(B)** Relation between QRS duration (*X*-axis) for the 5 selected complexes in the *i*ECG procedure and correlation coefficient/absolute difference for the LAT values (*Y*-axis). **(C)** Relation between annotation density (*X*-axis) per mm^2^ and correlation coefficient/absolute difference for LAT values for the 5 selected complexes in the *i*ECG procedure (*Y*-axis). **(D)** Relation between percentage of EAM of the total surface (*X*-axis) and correlation coefficient/absolute difference for LAT values (*Y*-axis).

Figure 2A shows an example of the comparison of *i*ECG and EAM for LAT maps, and the comparison between earliest and latest activated nodes for both the epicardium and endocardium. The ranges between earliest and latest ventricular activation were not significantly different between *i*ECG-LAT maps and EAM-LAT maps (111 ± 23 vs. 124 ± 39 ms, *p* = 0.311). The ranges of earliest and latest activation per patient are included in Supplementary Table 3. Figure 2B shows an example of the *i*ECG and the EAM approach in a patient with a healed myocarditis with right bundle branch block. The fast and His-Purkinje mediated activation of the left ventricular myocardium is shown in contrast to the relatively slower activation of the right ventricle due to the right bundle branch block. Figure 2C shows an example of the activation pattern and epicardial electrogram annotation in a patient with arrhythmogenic cardiomyopathy. Furthermore, all LAT and voltage maps of each included patient are available as Supplementary Figure 1. The mean correlation coefficient between *i*ECG-LAT maps and EAM-LAT maps was 0.54 ± 0.19; [95% CI:  0.49–0.59] for epicardial maps, 0.50 ± 0.27; [95% CI: 0.41–0.58] for endocardial right ventricular maps and 0.44 ± 0.29; [95% CI: 0.32–0.56] for endocardial left ventricular maps (Table 1). The moderate agreement of LAT between *i*ECG and EAM maps is shown in Figure 3A for all included electrograms on the epicardium (*R* = 0.632, *p* < 0.001), right ventricular endocardium (*R* = 0.597, *p* < 0.001) and left ventricular endocardium (*R* = 0.546, *p* < 0.001). Figure 3B shows that a prolonged QRS duration of the included complexes did not affect correlation coefficient or absolute difference. Figure 3C suggest that a higher density of mapped electrograms per mm2 reduces the scatter of correlation coefficients. The absolute difference for epicardial LAT maps was 17.4 ± 7.2 ms; [95% CI: 15.6–19.2], for endocardial right ventricular maps 19.5 ± 7.7 ms; [95% CI: 17.2–21.7], and for endocardial left ventricular maps 27.9 ± 8.7 ms; [95% CI: 24.2–31.5]. The relation between the percentage of mapped anatomical points during EAM and the agreement for LAT values is shown in Figure 3D. The correlation coefficient between *i*ECG-LAT maps and EAM-LAT maps was not significantly affected by the absolute number of EAM electrograms (*p* = 0.324), the number of electrograms with abnormal voltage (*p* = 0.306) or the QRS duration (*p* = 0.485) (see Supplementary Figure 2). However, the annotation density and the percentage of mapped anatomical points per map affected the agreement between *i*ECG and EAM. In maps with a low annotation density or lower percentage of mapped anatomical points the correlation coefficients were low (Figures 3C,D). This may have negatively affected the observed correlation coefficients in this study because endocardial EAM was often limited to either the right ventricular or left ventricular surface. The *i*ECG estimations were based on five QRS complexes selected from the body surface potential maps, but a Bland-Altman analysis did not result in divergent results per included QRS complex. These scatter plots and Bland-Altman plots for each included patient are available in Supplementary Figure 3.

**TABLE 1:** Comparison between *i*ECG and EAM.

**Parameters**	**Mean ± SD**	**Median [IQR]**
**Epicardium**		
Correlation coefficient	54.1 ± 19.0	51.0 [44.0 – 71.5]
Absolute difference (ms)	17.4 ± 7.2	15.1 [12.8 – 19.6]
Absolute difference earliest breakthrough (mm)	42.1 ± 18.6	37.9 [28.4 – 58.5]
Absolute difference terminal site of activation (mm)	54.1 ± 26.9	51.0 [33.4 - 69.6]
Absolute difference timing of latest activation (ms)	19.1 ± 20.9	12.8 [2.9 – 29.3]
EAM breakthroughs (n)	3.15 ± 0.9	3.0 [2.5 - 4.0]
*i*ECG breakthroughs (n)	3.3 ± 0.8	3.4 [2.9 – 4.0]
**Right ventricular endocardium**		
Correlation coefficient	49.6 ± 27.3	55.5 [46.0 – 62.0]
Absolute difference (ms)	19.5 ± 7.7	17.4 [13.2 - 24.4]
Absolute difference earliest breakthrough (mm)	29.9 ± 16.0	28.3 [22.3 - 47.4]
Absolute difference terminal site of activation (mm)	46.7 ± 28.8	37.0 [24.5 - 69.4]
Absolute difference timing of latest activation (ms)	20.4 ± 16.7	15.2 [10.1 - 28.7]
EAM breakthroughs (n)	2.1 ± 0.6	2.0 [2.0 - 2.25]
*i*ECG breakthroughs (n)	1.8 ± 0.6	2.0 [1.2 - 2.3]
**Left ventricular endocardium**		
Correlation coefficient	44.0 ± 28.8	53.5 [13.5 - 65.0]
Absolute difference (ms)	27.9 ± 8.7	27.3 [20.1 - 36.2]
Absolute difference earliest breakthrough (mm)	31.0 ± 16.8	31.1 [14.7 - 47.1]
Absolute difference terminal site of activation (mm)	32.7 ± 17.2	39.2 [14.8 – 44.1]
Absolute difference timing of latest activation (ms)	29.5 ± 26.3	20.8 [10.4 - 57.3]
EAM breakthroughs (n)	1.8 ± 1.0	1.5 [1.0 - 2.8]
*i*ECG breakthroughs (n)	1.8 ± 0.5	2.0 [1.3 - 2.0]

### Localization of Earliest Breakthrough and Areas of Latest Activation

The number of endocardial breakthrough points was similar when comparing *i*ECG-LAT maps and EAM-LAT maps: 3.3 ± 0.8 vs. 3.2 ± 0.9 for epicardial maps, 1.8 ± 0.6 vs. 2.1 ± 0.6 for right ventricular endocardial maps and 1.8 ± 0.5 vs. 1.8 ± 1.0 for left ventricular endocardial maps (Table 1). These findings were in line with the observations of Durrer et al. and the assumptions of the *i*ECG initial estimation (Durrer et al., 1970). Epicardial breakthrough of activation had an absolute difference between *i*ECG-LAT maps and EAM-LAT maps of 42.1 ± 18.6 mm; [95% CI: 36.7–47.5]. For endocardial breakthrough of activation, the absolute difference was 29.9 ± 16.0 mm; [95% CI:  25.1–34.8] for the right ventricular endocardium and 31.0 ± 16.8 mm; [95% CI: 23.8–38.1] for the left ventricular endocardium. The latest activated nodes had an absolute difference between *i*ECG and EAM of 54.1 ± 26.9 mm; [95% CI: 47.5–60.7] for epicardial maps, 46.7 ± 28.8 mm; [95% CI: 38.8–54.7] for right ventricular endocardial maps and 32.7 ± 17.2mm; [95% CI: 25.1–40.4] for left ventricular endocardial maps (Table 1). The timing of the latest activated nodes differed 12.8 [2.9–29.3] ms; [95% CI: 6.4–31.7] for epicardial maps, 15.2 [10.1–28.7] ms; [95% CI:  8.5–32.5] for right ventricular endocardial maps and 20.8 [10.4–57.3] ms; [95% CI: 12.5–71.4] for left ventricular endocardial maps. The myocardial conduction velocity was not significantly different between *i*ECG and EAM maps for, respectively, the epicardium (1.26 ± 0.16 vs. 1.26 ± 0.20 m/s, *p* > 0.999), right ventricular endocardium (1.13 ± 0.09 vs.. 0.94 ± 0.17 m/s, *p* = 0.069) or left ventricular endocardium (1.03 ± 0.11 vs. 0.92 ± 0.07 m/s, *p* = 0.968).

## Discussion

This is the first study to quantitatively compare non-invasive, EDL-based *i*ECG estimation of ventricular activation sequences during sinus rhythm with invasive high density endocardial and epicardial EAM in humans. Comparison of agreement between *i*ECG-LAT maps with EAM-LAT maps showed moderate agreement. However, this observed agreement (correlation coefficient = 0.54 ± 0.19) was remarkably higher compared to a recent validation study (correlation coefficient = −0.04 ± 0.3) performed during sinus rhythm (Duchateau et al., 2019). Mimicking the effects of the His-Purkinje system on ventricular activation in the *i*ECG method resulted in activation patterns corresponding to observations of Durrer et al. in experiments with explanted human hearts (Durrer et al., 1970). In contrast to prior EPD-based studies which were limited to estimations on the epicardium, estimation of both the endocardial and epicardial activation sequences was achieved. Although accuracy and spatial resolution require further improvement before implementation of this diagnostic tool in clinical practice, these findings may be of clinical importance for functional non-invasive substrate imaging during sinus rhythm to improve the value of ECG screening and risk stratification of sudden cardiac death (Tung et al., 2020).

### Quantitative Comparison

Previous quantitative EPD-based validation studies showed higher agreement between ventricular paced complexes and EAM, compared to sinus rhythm complexes (Duchateau et al., 2019; Graham et al., 2019). Duchateau et al. (2019) showed poor epicardial inter-map correlation coefficient (−0.04 ± 0.3) during sinus rhythm, although correlation coefficients increased with increasing QRS duration. This relation is most likely explained by the complexity of multiple simultaneous ventricular activation waveforms occurring during sinus rhythm, which decreases in rhythms with a single focus (Duchateau et al., 2019). In the present study, a considerably higher agreement (correlation coefficient 0.54 ± 0.19) between EAM and the novel *i*ECG-LAT maps was observed during sinus rhythm. This improved performance is attributed to the incorporation of the effects of the His-Purkinje system on the initiation of ventricular activation (Oosterhoff et al., 2016). Previously reported absolute difference for breakthrough of epicardial pacing was smaller compared to the present study (13.2–20.7 mm vs. 42.1 ± 18.6 mm) (Oosterhoff et al., 2016; Graham et al., 2019; Hohmann et al., 2019). However, previously reported absolute difference for epicardial breakthrough during sinus rhythm was higher compared to our results (75.6 ± 38.1 mm vs. 42.1 ± 18.6 mm) (Duchateau et al., 2019). Again, these differences may be explained by estimations of rhythms originating from a single ventricular focus and sinus rhythm. Thus, spatial resolution observed in this study was comparable to the earlier studies in paced complexes (Cluitmans et al., 2017; Hohmann et al., 2019). Due to the complex nature of the His-Purkinje system and the Purkinje-myocardial coupling, the implemented methods remain an approximation of the true myocardial activation and His-Purkinje physiology (Durrer et al., 1970; Myerburg, 1971; Veenstra et al., 1984).

We observed a high agreement between estimated and measured body surface potential maps, whereas the inter-map agreement was less. As the inverse problem is ill-posed, completely different ventricular activation sequences can result in similar body surface potential map waveforms, consequently we found a high agreement between body surface potential maps but a lower agreement in myocardial activation patterns.

The conduction velocities calculated on the epicardial and endocardial surfaces in this study for both the EAM-LAT maps and *i*ECG-LAT maps were quite high (>1 m/s). However, we note that these conduction velocities are mostly determined by the velocity estimated at the surface of the myocardium. Consequently, in a Purkinje dense region, surface velocity may appear high because it also reflects the effect of the activation spread by the Purkinje fibers and not only by the myocardial tissue at the endocardial surface. Furthermore, at the epicardial surface, velocities may appear high due to the occurrence of transmural waves.

### Modeling the Effects of the His-Purkinje System During Sinus Rhythm

In this study, initial sites of activation were determined in the *i*ECG method based on the observations of Durrer et al. and nine possible sites of early activation were localized (Durrer et al., 1970). Sets of these initial sites of activation were tested based on the correlation coefficient between the computed and recorded body surface potential maps, as described in more detail in the Supplementary Methods. This hypothesis was partially tested by comparing the EAM-LAT maps to the *i*ECG-LAT maps. However, as endocardial EAM-LAT maps were often either of the right or the left endocardial surface and also did not cover the complete endocardial surface for each patient, the comparison between the number of identified EAM foci and *i*ECG foci was hampered. This was also reflected in the absolute difference in location of identified foci of approximately 30 mm comparing *i*ECG foci to EAM foci.

Previous versions of EDL-based methods estimating His-Purkinje mediated activation (e.g., sinus rhythm) were based on a multi-focal search algorithm over the complete endocardium and epicardium, where the first identified focus was chosen based on the highest correlation between recorded and simulated body surface potentials (van Dam et al., 2009; Oosterhoff et al., 2016). Consequently, this algorithm directly assumed that by using one focus, most of the underlying activation sequence could be ‘explained’. However, sinus rhythm, and especially narrow QRS complex sinus rhythm is an interplay between multiple activation wavefronts. Implementation of the His-Purkinje system excludes these unrealistic estimates and provides the possibility to test multiple near simultaneous foci. At the same time the initial estimation is restricted to the physiologically realistic anatomical areas and the computational burden of the *i*ECG algorithm is minimized.

### Post-processing and Reference Standard

Post-processing of ECG signals, electrogram signals, and cardiac imaging influences *i*ECG accuracy (Cluitmans et al., 2018; Graham et al., 2019). To achieve high quality EAM-LAT maps, which were used as gold standard for comparison, electrograms derived from multi-electrode catheters required re-annotation using bipolar and unipolar signals and timing to a timing reference (Cantwell et al., 2015; Graham et al., 2019). However, inhomogeneity in LAT distributions of EAM-LAT maps were observed even after re-annotation, which may have influenced the observed agreement between *i*ECG and EAM-LAT maps. Both the epicardial and endocardial surfaces had an adequate spatial distribution of electrograms as reflected in the number of LAT per mm2 (see Figure 3C). Furthermore, the percentage of mapped surfaces was variable and some EAM procedures resulted in incomplete endocardial EAM anatomical point clouds, which affects calculated inter-map correlation coefficient (see Figures 3C,D).

### Clinical Implications and Future Directions

Despite a considerable improvement of the *i*ECG approach for sinus rhythm, the technique requires further adaptations and refinements that will facilitate implementation in clinical practice. Further integration of cardiovascular imaging techniques may improve performance and spatial resolution (Tung et al., 2020). Currently, the patient specific anatomical models were limited in spatial resolution by the computational models of the *i*ECG procedure, allowing at maximum 3000 cardiac nodes, which directly affects the resolution of the cardiac anatomical model resulting in an inter-node spatial resolution of 8 ± 1mm. Diffuse or local myocardial fibrosis affects ventricular activation patterns in structurally diseased hearts. Integration of these structural abnormalities in the *i*ECG method and refinement of the cardiac anatomical models is likely to improve imaging of electro-anatomical substrates (van Dam et al., 2009; Oosterhoff et al., 2016; Tung et al., 2020). Since electro-anatomical substrates are not limited to solely the epicardium or endocardium, *i*ECG may allow functional imaging of such 3-dimentional substrates in patients with arrhythmias or cardiomyopathy (Tung et al., 2020). Besides diagnostic implications, non-invasive sinus rhythm *i*ECG may play a role in the monitoring of disease progression and in sudden cardiac death risk stratification in patients with complex electroanatomical substrates, such as inherited cardiomyopathies. Eventually, reducing the number of electrodes of the body surface potential map that currently ranges from 67 to 256 electrodes, may improve clinical applicability (Hoekema et al., 1999). For EDL-based studies, also this study, the 64-electrode setup is often used (van Dam et al., 2009; Oosterhoff et al., 2016). Mathematically this setup suffices, as the number of independent signals is adequately captured using this number of electrodes and additionally, the electrodes are distributed with a high resolution in the high-gradient potential regions on the surface of the thorax (Hoekema et al., 1999).

### Limitations

This single center study with a small sample size included patients with structural heart disease, which may influence the generalizability of the results. Additionally, we used a set conduction velocity over the model to determine the initial estimation. This assumption may not hold in the presence of pathologies or myocardial scarring after prior ablation, but the EDL holds for homogeneous anisotropic tissue (Geselowitz, 1992).

Electro-anatomical mapping procedures and body surface potential maps were not simultaneously recorded, but in similar conditions especially concerning anti-arrhythmic drugs. During EAM, complexes were selected using dedicated Carto/Ensite EAM systems. Furthermore, sinus rhythm complexes directly following a premature ventricular complex were excluded for analysis in both the EAM and *i*ECG-LAT map. However, a possible influence of variations in heart rate, autonomic tonus or general anesthesia cannot be excluded. The quality of gold standard EAM may have been influenced by vendor specific algorithms within the EAM systems and regional mapping by the operator during the procedure. Inherent to invasive electrophysiological studies, EAM maps consisted of electrograms recorded from consecutive sinus rhythm complexes, whereas *i*ECG maps were derived from five sinus rhythm complexes selected from the body surface potential map.

### Conclusion

Quantitative comparison of EDL-based *i*ECG during sinus rhythm in patients undergoing invasive endocardial and epicardial electro-anatomical mapping showed improved agreement when compared to prior validation studies, although with considerable absolute difference in both timing and breakthrough of ventricular activation. Non-invasive *i*ECG of both the epicardium and endocardium may prove valuable as a diagnostic tool for functional imaging of electro-anatomical substrates in sinus rhythm where activation always starts at the endocardial surface, to improve the value of the ECG in screening for cardiomyopathy and sudden cardiac death risk stratification. Future research should focus on improving accuracy and spatial resolution before implementation into clinical practice to enable imaging of functional electro-anatomical substrates.

## Data Availability Statement

The raw data supporting the conclusions of this article will be made available by the authors, without undue reservation.

## Ethics Statement

The studies involving human participants were reviewed and approved by METC UMC Utrecht. Written informed consent to participate in this study was provided by the participants’ legal guardian/next of kin.

## Author Contributions

RR, MB, JH, TO, FA, PD, and PL contributed to conception and design of the study. RB, WK, LB, RR, and MB organized the database. RR and MB performed the statistical analysis. RR wrote the first draft of the manuscript. RR, MB, PD, and PL wrote sections of the manuscript. All authors contributed to manuscript revision, read, and approved the submitted version.

## Conflict of Interest

PD is owner of ECG Excellence BV. The remaining authors declare that the research was conducted in the absence of any commercial or financial relationships that could be construed as a potential conflict of interest.

## Publisher’s Note

All claims expressed in this article are solely those of the authors and do not necessarily represent those of their affiliated organizations, or those of the publisher, the editors and the reviewers. Any product that may be evaluated in this article, or claim that may be made by its manufacturer, is not guaranteed or endorsed by the publisher.

## Supplementary Material

Supplementary Figure 1 All *i*ECG, EAM maps, and voltage maps.

Supplementary Figure 2 Factors associated with correlation coefficients and absolute differences.

Supplementary Figure 3 Scatterplot and Bland-Altman plot per included patient.

Supplementary Table 1 Summary data of the study population.

Supplementary Table 2 Detailed study population per included patient.

Supplementary Table 3 Ranges of earliest and latest activation per subject.

Supplementary Methods Detailed methods section on *i*ECG multi-wave initial estimation.

## References

[B1] Bergquist J. A. Good W. W. Zenger B. Tate J. D. MacLeod R. S. (2019). GRÖMeR: a pipeline for geodesic refinement of mesh registration. *Funct. Imaging Model. Heart* 11504 37–45. 10.1007/978-3-030-21949-9_5 31799512 PMC6889814

[B2] Cantwell C. D. Roney C. H. Ng F. S. Siggers J. H. Sherwin S. J. Peters N. S. (2015). Techniques for automated local activation time annotation and conduction velocity estimation in cardiac mapping. *Comput. Biol. Med.* 65 229–242. 10.1016/j.compbiomed.2015.04.027 25978869 PMC4593301

[B3] Cluitmans M. Brooks D. H. MacLeod R. Dossel O. Guillem M. S. van Dam P. M. (2018). Validation and opportunities of electrocardiographic imaging: from technical achievements to clinical applications. *Front. Physiol.* 9:1305. 10.3389/fphys.2018.01305 30294281 PMC6158556

[B4] Cluitmans M. J. M. Bonizzi P. Karel J. M. H. Das M. Kietselaer B. de Jong M. M. J. (2017). In vivo validation of electrocardiographic imaging. *JACC Clin. Electrophysiol.* 3 232–242. 10.1016/j.jacep.2016.11.012 29759517

[B5] Cosio F. G. Anderson R. H. Kuck K. H. Becker A. Borggrefe M. Campbell R. W. (1999). Living anatomy of the atrioventricular junctions. A guide to electrophysiologic mapping. A consensus statement from the cardiac nomenclature study group, working group of arrhythmias, European society of cardiology, and the task force on cardiac nomenclature from NASPE. *Circulation* 100 e31–e37. 10430823 10.1161/01.cir.100.5.e31

[B6] Duchateau J. Sacher F. Pambrun T. Derval N. Chamorro-Servent J. Denis A. (2019). Performance and limitations of noninvasive cardiac activation mapping. *Heart Rhythm* 16 435–442. 10.1016/j.hrthm.2018.10.010 30385382

[B7] Durrer D. van Dam R. T. Freud G. E. Janse M. J. Meijler F. L. Arzbaecher R. C. (1970). Total excitation of the isolated human heart. *Circulation* 41 899–912. 10.1161/01.cir.41.6.899 5482907

[B8] Geselowitz D. B. (1992). Description of cardiac sources in anisotropic cardiac muscle. Application of bidomain model. *J. Electrocardiol.* 25 65–67. 10.1016/0022-0736(92)90063-6 1297711

[B9] Graham A. J. Orini M. Zacur E. Dhillon G. Daw H. Srinivasan N. T. (2019). Simultaneous comparison of electrocardiographic imaging and epicardial contact mapping in structural heart disease. *Circ. Arrhythm Electrophysiol.* 12:e007120. 30947511 10.1161/CIRCEP.118.007120

[B10] Greensite F. Huiskamp G. van Oosterom A. (1990). New quantitative and qualitative approaches to the inverse problem of electrocardiology: their theoretical relationship and experimental consistency. *Med. Phys.* 17 369–379. 10.1118/1.596568 2385194

[B11] Hoekema R. Uijen G. J. van Oosterom A. (1999). On selecting a body surface mapping procedure. *J. Electrocardiol.* 32 93–101. 10.1016/s0022-0736(99)90088-2 10338028

[B12] Hohmann S. Rettmann M. E. Konishi H. Borenstein A. Wang S. Suzuki A. (2019). Spatial accuracy of a clinically established noninvasive electrocardiographic imaging system for the detection of focal activation in an intact porcine model. *Circ. Arrhythm. Electrophysiol.* 12:e007570. 31707808 10.1161/CIRCEP.119.007570

[B13] Huiskamp G. Greensite F. (1997). A new method for myocardial activation imaging. *IEEE Trans. Biomed. Eng.* 44 433–446. 10.1109/10.581930 9151476

[B14] Huiskamp G. Van Oosterom A. (1988). The depolarization sequence of the human heart surface computed from measured body surface potentials. *IEEE Trans. Biomed. Eng.* 35 1047–1058. 3220498 10.1109/10.8689

[B15] Leon L. J. Witkowski F. X. (1995). Calculation of transmembrane current from extracellular potential recordings: a model study. *J. Cardiovasc. Electrophysiol.* 6 379–390. 10.1111/j.1540-8167.1995.tb00411.x 7551307

[B16] Myerburg R. J. (1971). The gating mechanism in the distal atrioventricular conducting system. *Circulation* 43 955–960. 10.1161/01.cir.43.6.955 4102930

[B17] Oosterhoff P. Meijborg V. M. van Dam P. M. van Dessel P. F. Belterman C. N. Streekstra G. J. (2016). Experimental validation of noninvasive epicardial and endocardial activation imaging. *Circ. Arrhythm. Electrophysiol.* 9:e004104. 27439651 10.1161/CIRCEP.116.004104

[B18] Ramanathan C. Ghanem R. N. Jia P. Ryu K. Rudy Y. (2004). Noninvasive electrocardiographic imaging for cardiac electrophysiology and arrhythmia. *Nat. Med.* 10 422–428. 10.1038/nm1011 15034569 PMC1950745

[B19] Rudy Y. (2013). Noninvasive electrocardiographic imaging of arrhythmogenic substrates in humans. *Circ. Res.* 112 863–874. 10.1161/circresaha.112.279315 23449548 PMC3596167

[B20] Sapp J. L. Dawoud F. Clements J. C. Horacek B. M. (2012). Inverse solution mapping of epicardial potentials: quantitative comparison with epicardial contact mapping. *Circ. Arrhythm Electrophysiol.* 5 1001–1009. 10.1161/circep.111.970160 22923272

[B21] Sosa E. Scanavacca M. d’Avila A. Pilleggi F. (1996). A new technique to perform epicardial mapping in the electrophysiology laboratory. *J. Cardiovasc. Electrophysiol.* 7 531–536. 10.1111/j.1540-8167.1996.tb00559.x 8743758

[B22] Tung R. Raiman M. Liao H. Zhan X. Chung F. P. Nagel R. (2020). Simultaneous endocardial and epicardial delineation of 3D reentrant ventricular tachycardia. *J. Am. Coll. Cardiol.* 75 884–897. 10.1016/j.jacc.2019.12.044 32130924

[B23] van Dam P. M. Oostendorp T. F. Linnenbank A. C. van Oosterom A. (2009). Non-invasive imaging of cardiac activation and recovery. *Ann. Biomed. Eng.* 37 1739–1756. 10.1007/s10439-009-9747-5 19562487 PMC2721141

[B24] van Oosterom A. (2014). A comparison of electrocardiographic imaging based on two source types. *Europace* 16 (Suppl. 4) iv120–iv128. 25362162 10.1093/europace/euu268

[B25] Veenstra R. D. Joyner R. W. Rawling D. A. (1984). Purkinje and ventricular activation sequences of canine papillary muscle. Effects of quinidine and calcium on the Purkinje-ventricular conduction delay. *Circ. Res.* 54 500–515. 10.1161/01.res.54.5.500 6722999

