# Supplementary material for: Comparing Non-invasive Inverse Electrocardiography With Invasive Endocardial and Epicardial Electroanatomical Mapping During Sinus Rhythm
Source: Front Physiol. 2021 Oct 4;12:730736. doi: 10.3389/fphys.2021.730736 (PMC8521153; doi:10.3389/fphys.2021.730736)
Supplement: Supplementary Figure 1 — All iECG, EAM maps, and voltage maps. [file Image_1.PDF]

Supplementary Figure 1

All *i*ECG, EAM maps and voltage maps

Legend: Epicardial and endocardial local activation timing (LAT) maps and voltage maps derived from *i*ECG estimation and invasive electroanatomical mapping for each patient. Areas of earliest activation are depicted with red and areas with latest activation are depicted in blue. Grey areas in the invasive maps represent areas without annotated electrograms. Abnormal voltage EGMs were defined as bipolar voltage amplitude <0.5 mV for both the epicardial and endocardial surface. Areas with the lowest voltage EGMs are depicted with red and areas with >1.5 mV voltage EGMs are depicted with purple. See **Supplementary Table 3** for LAT ranges. Imaging views are based on the anatomical approach of Cosio et al. Abbreviations: iECG: inverse electrocardiography; LAO = left anterior oblique; RAO = right anterior oblique.

Per slide, a representative example per subject of all invasively mapped surfaces are displayed in RAO, LAO and Inferior view. Maps are displayed from early (red) to late activation for the *i*ECG estimation (**left panel**) and invasive map (**middle panel**). Per surface, time scales are displayed below the maps. Additionally, the invasive voltage map (**right panel**) is displayed. Correlation coefficient (CC) and absolute difference (AD) of the activation times between the *i*ECG and invasive map are displayed at the top of each slide per presented surface.

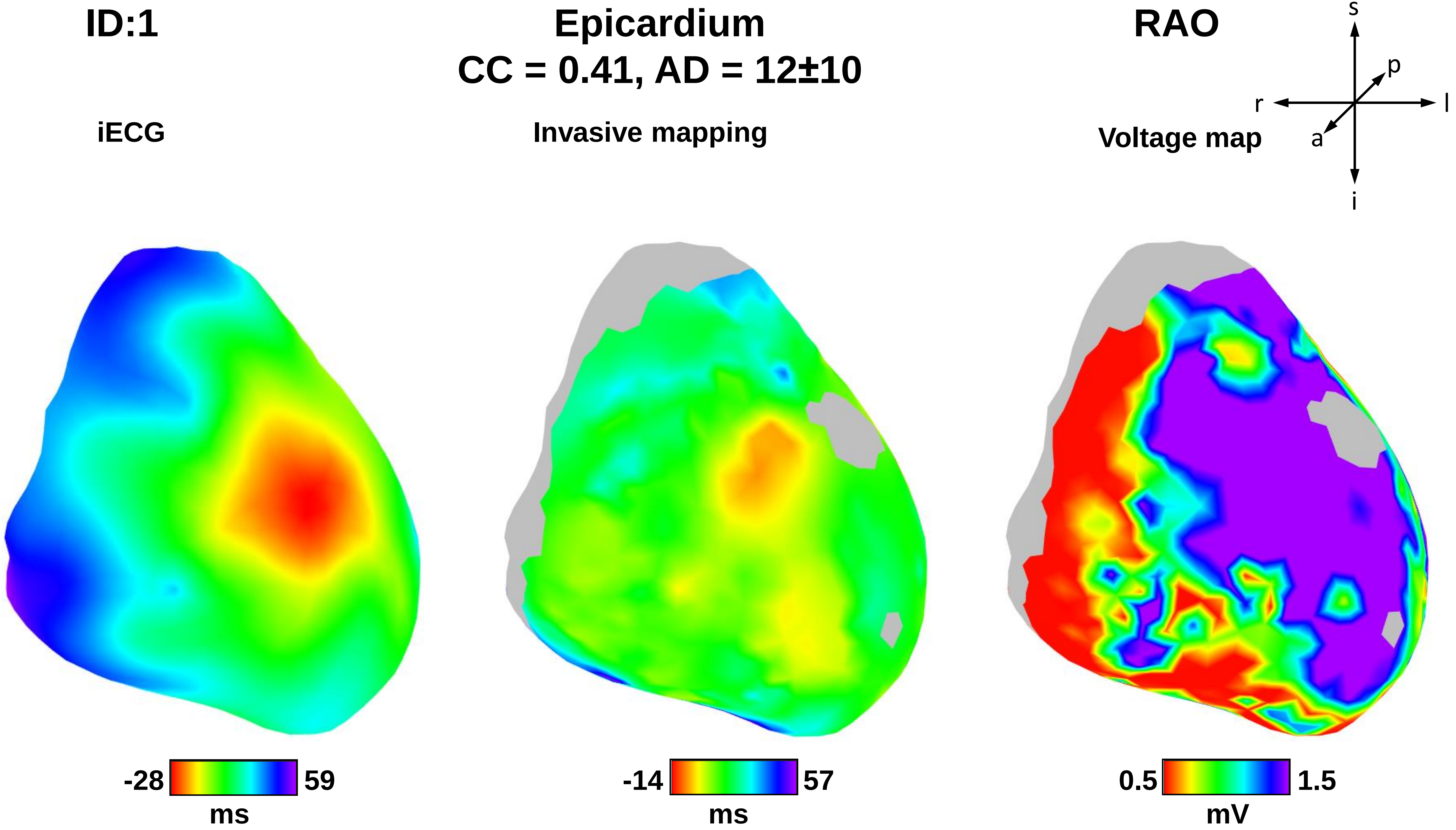
ID:1
Epicardium
CC = 0.41, AD = 12±10
RAO
s
p
r
l
a
i
iECG
Invasive mapping
Voltage map
-28
59
ms
-14
57
ms
0.5
1.5
mV

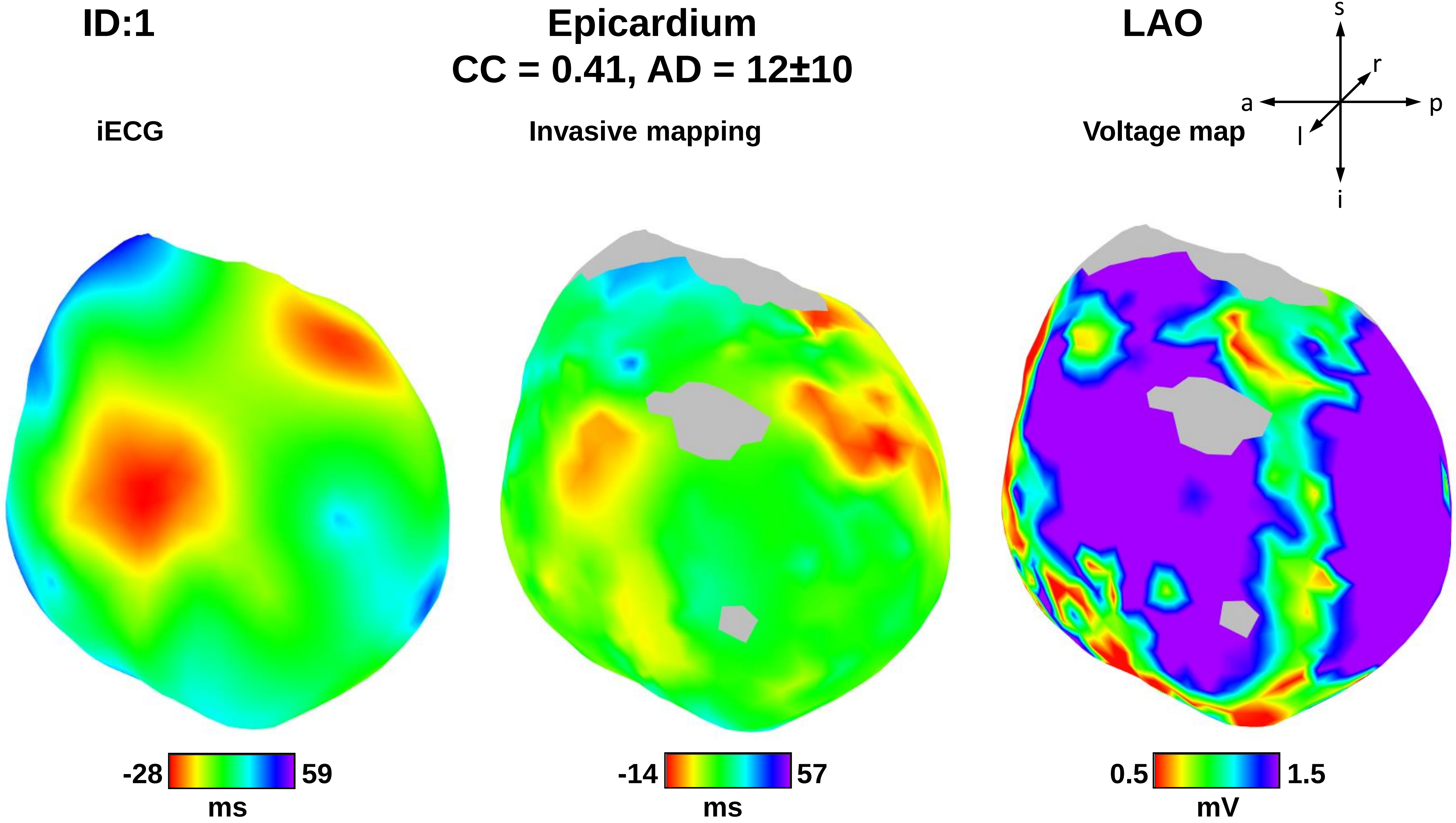
ID:1
Epicardium
CC = 0.41, AD = 12±10
LAO
s
r
a
p
l
i
iECG
Invasive mapping
Voltage map
-28
59
ms
-14
57
ms
0.5
1.5
mV

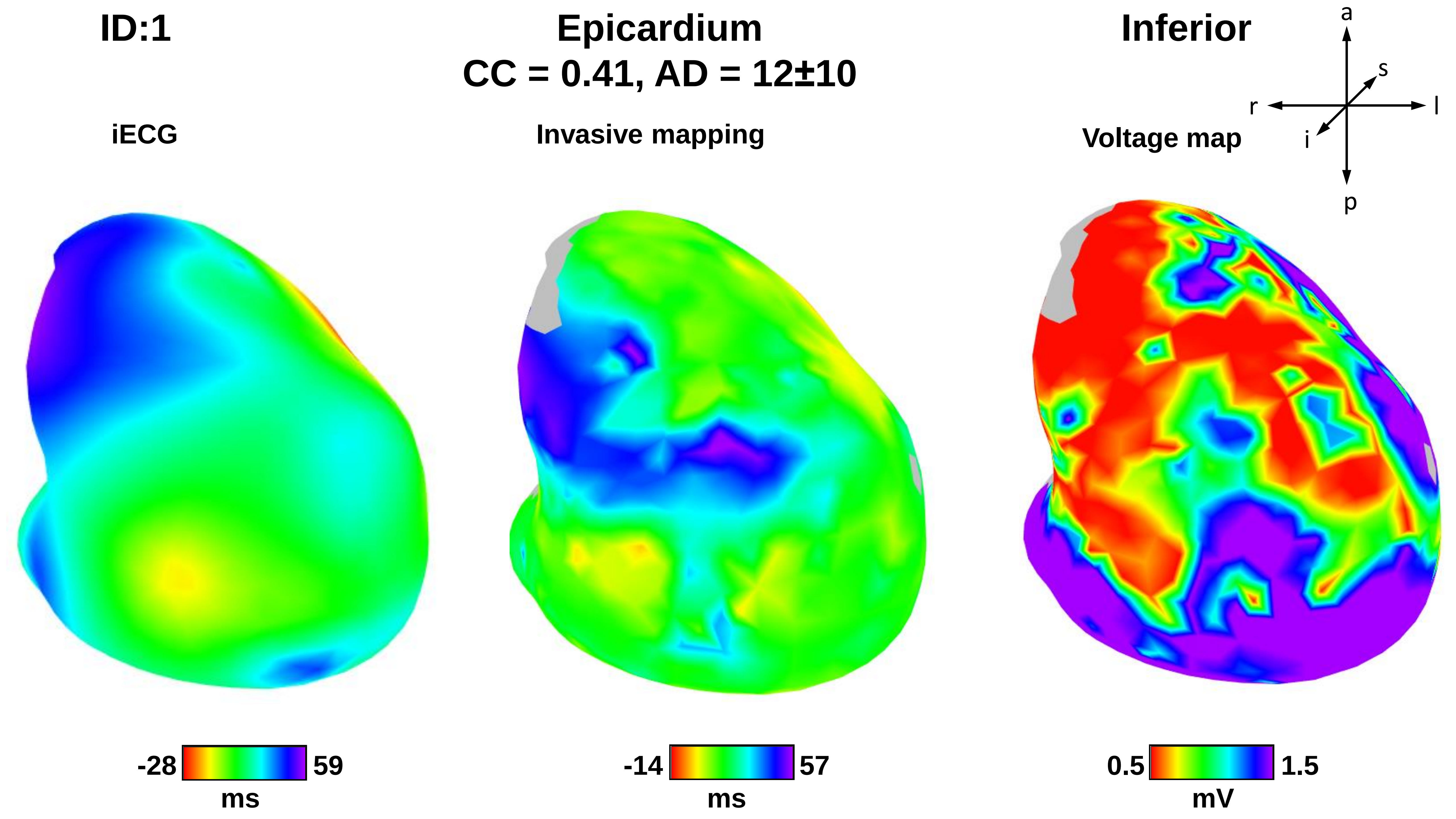
ID:1
Epicardium
CC = 0.41, AD = 12±10
Inferior
a
s
r
l
i
p
iECG
Invasive mapping
Voltage map
-28
59
ms
-14
57
ms
0.5
1.5
mV

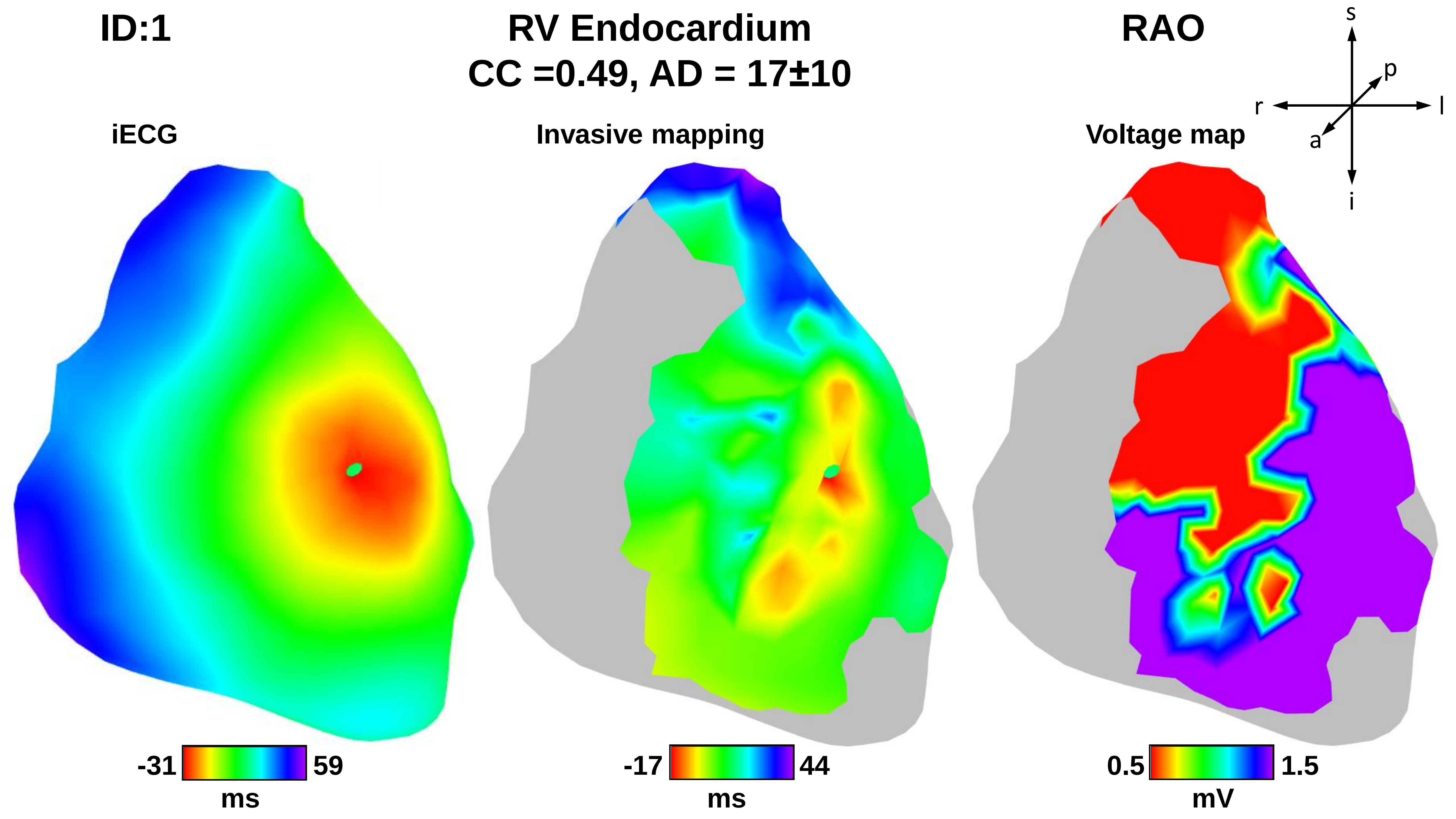
ID:1
RV Endocardium
CC =0.49, AD = 17±10
RAO
s
p
r
l
a
i
iECG
Invasive mapping
Voltage map
-31
59
ms
-17
44
ms
0.5
1.5
mV

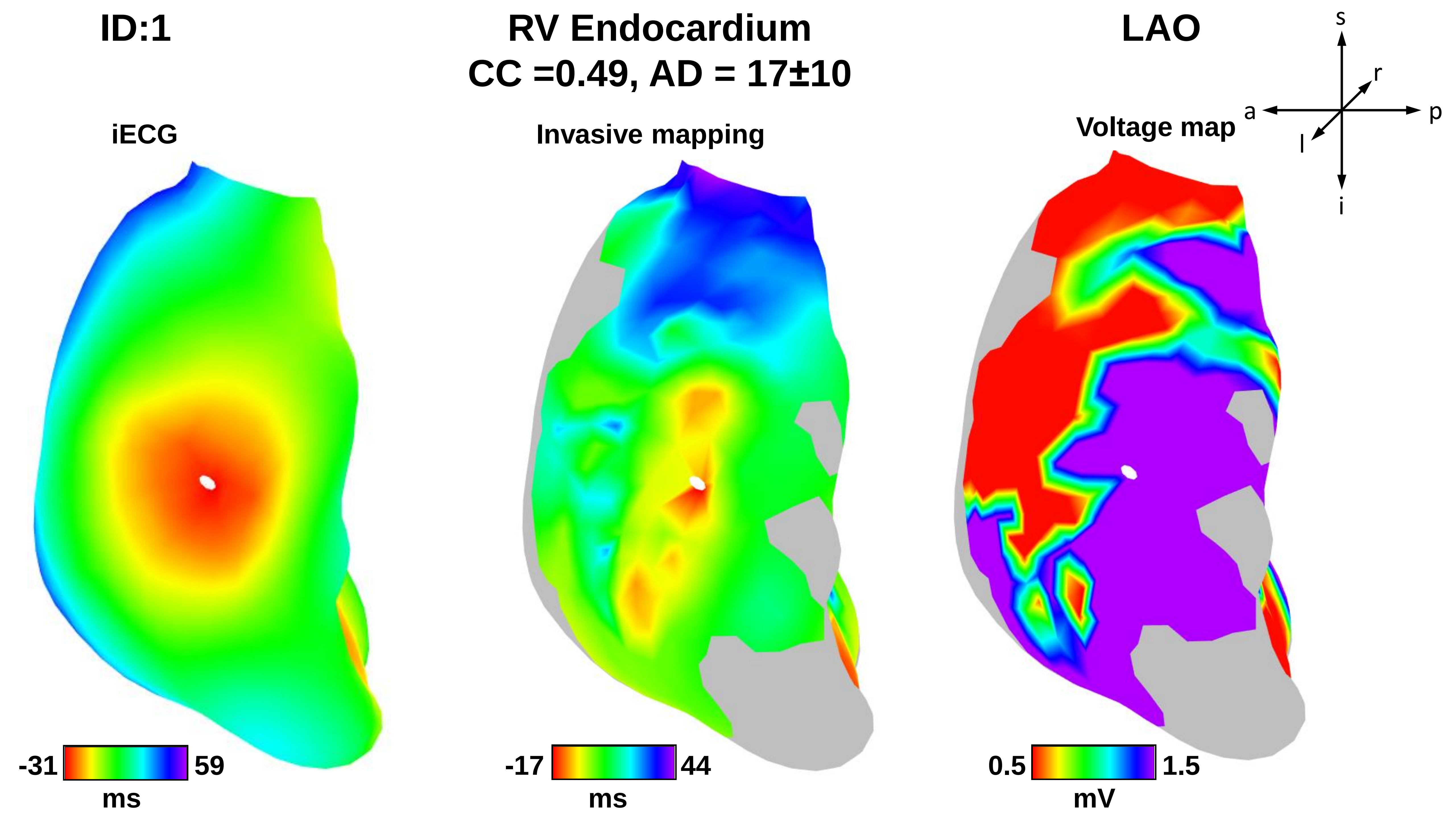

ID:1
RV Endocardium
CC =0.49, AD = 17±10
LAO
iECG
Invasive mapping
Voltage map
s
r
a
p
l
i
-31
59
ms
-17
44
ms
0.5
1.5
mV

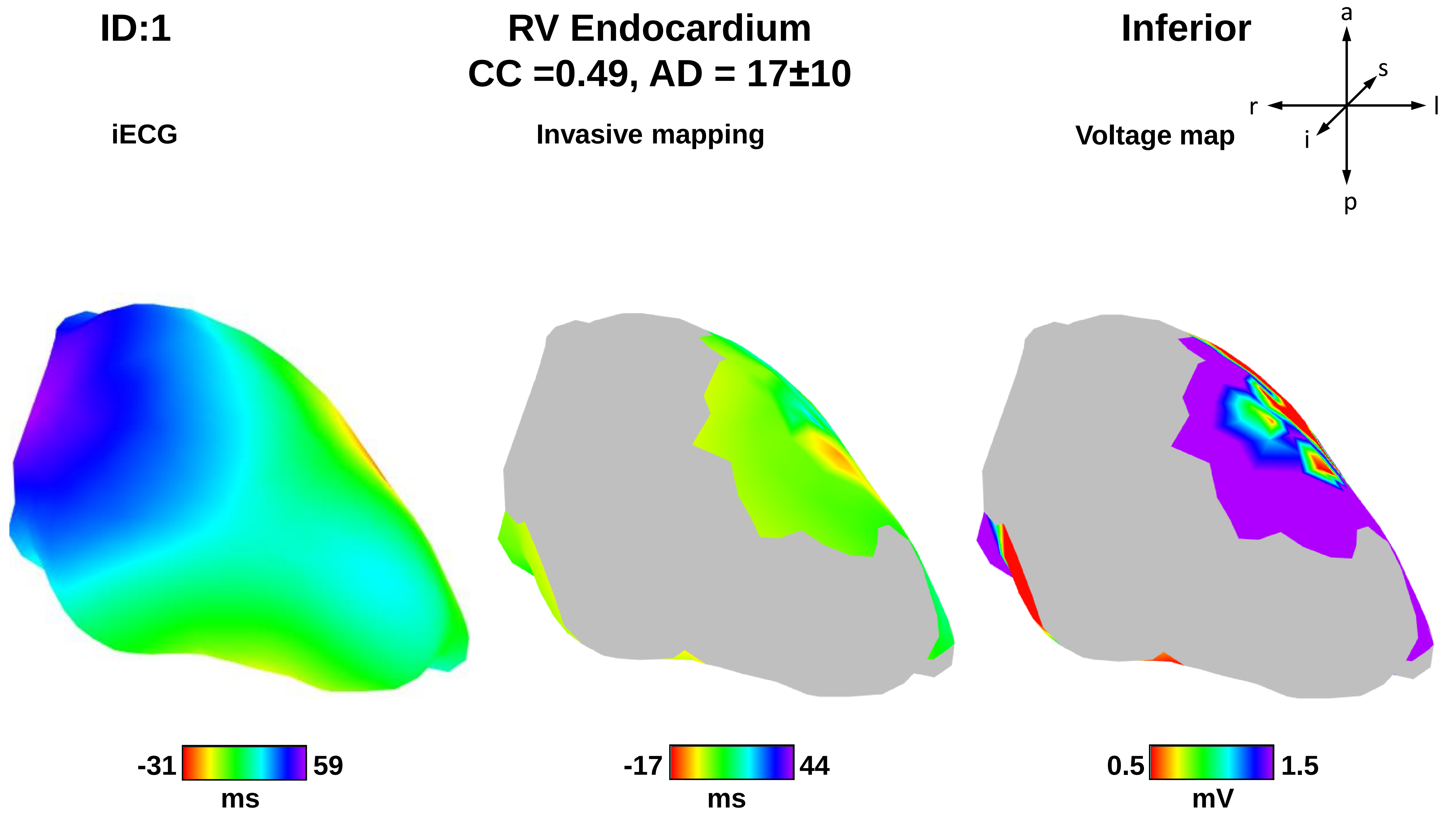

ID:1
RV Endocardium
CC =0.49, AD = 17±10
Inferior
a
s
r
l
i
p
iECG
Invasive mapping
Voltage map
-31
59
ms
-17
44
ms
0.5
1.5
mV

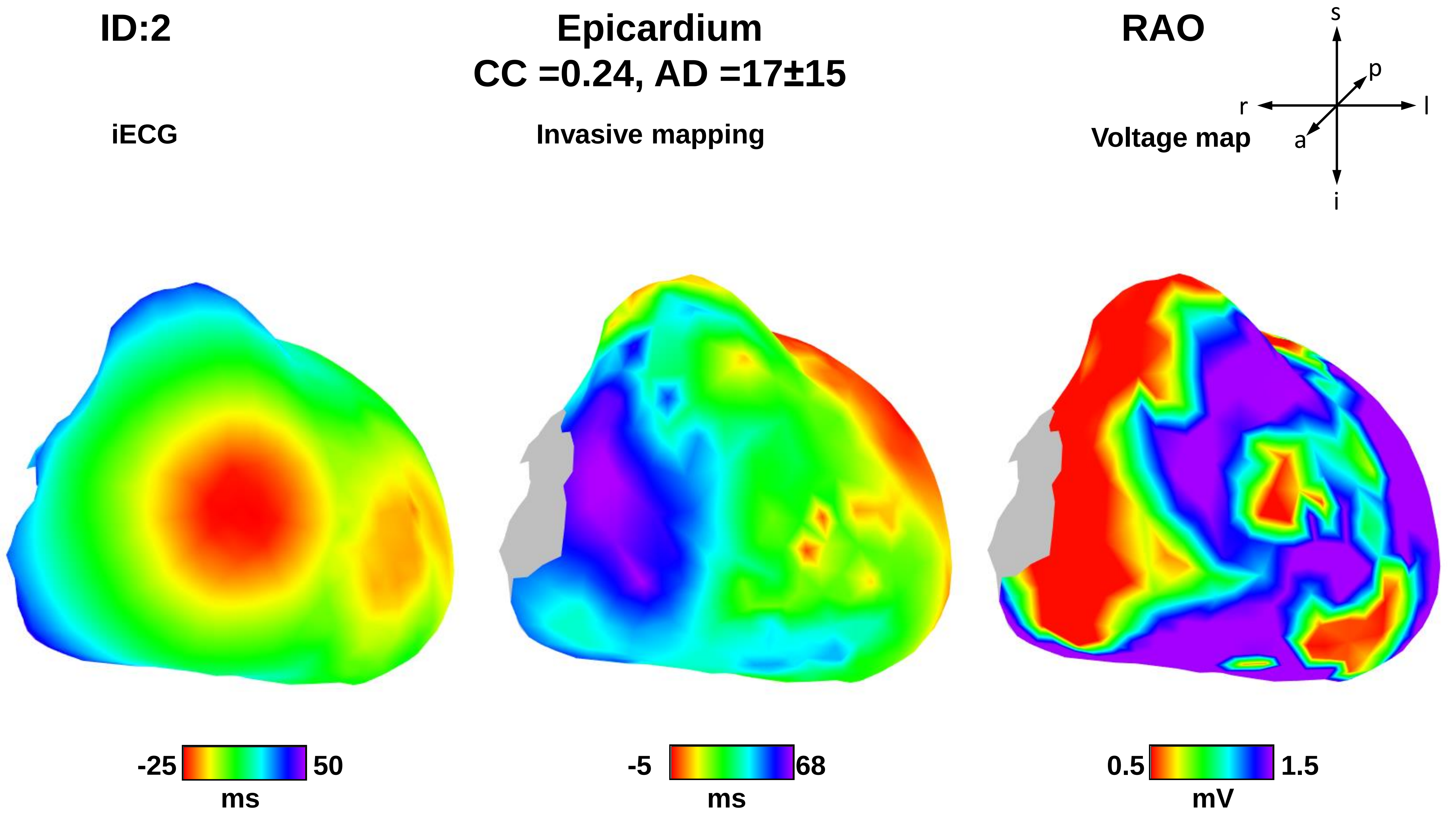

ID:2
Epicardium
CC =0.24, AD =17±15
RAO
s
p
r
l
a
i
iECG
Invasive mapping
Voltage map
-25
50
ms
-5
68
ms
0.5
1.5
mV

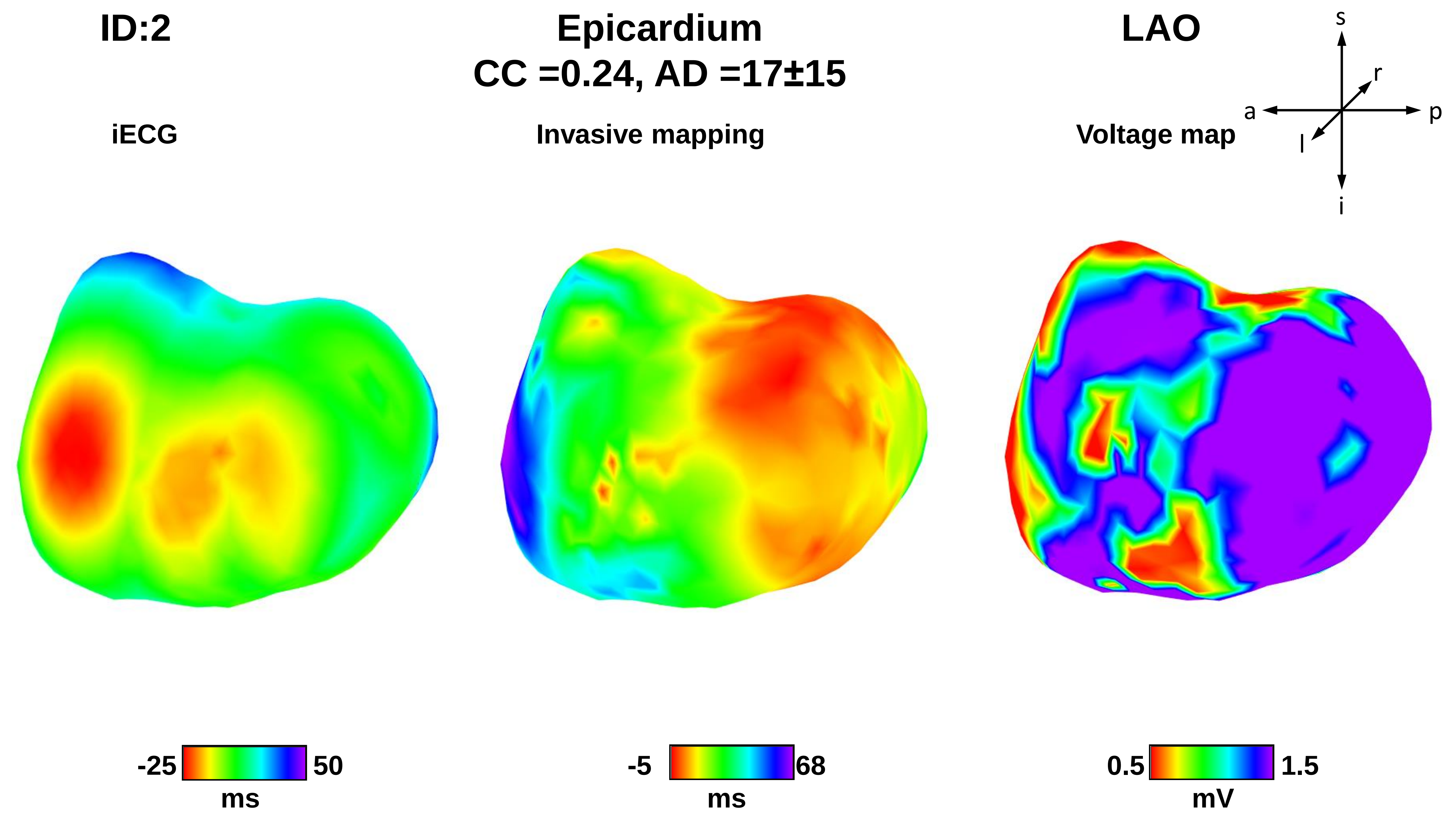
ID:2
Epicardium
CC =0.24, AD =17±15
LAO
iECG
Invasive mapping
Voltage map
s
r
a
p
l
i
-25
50
ms
-5
68
ms
0.5
1.5
mV

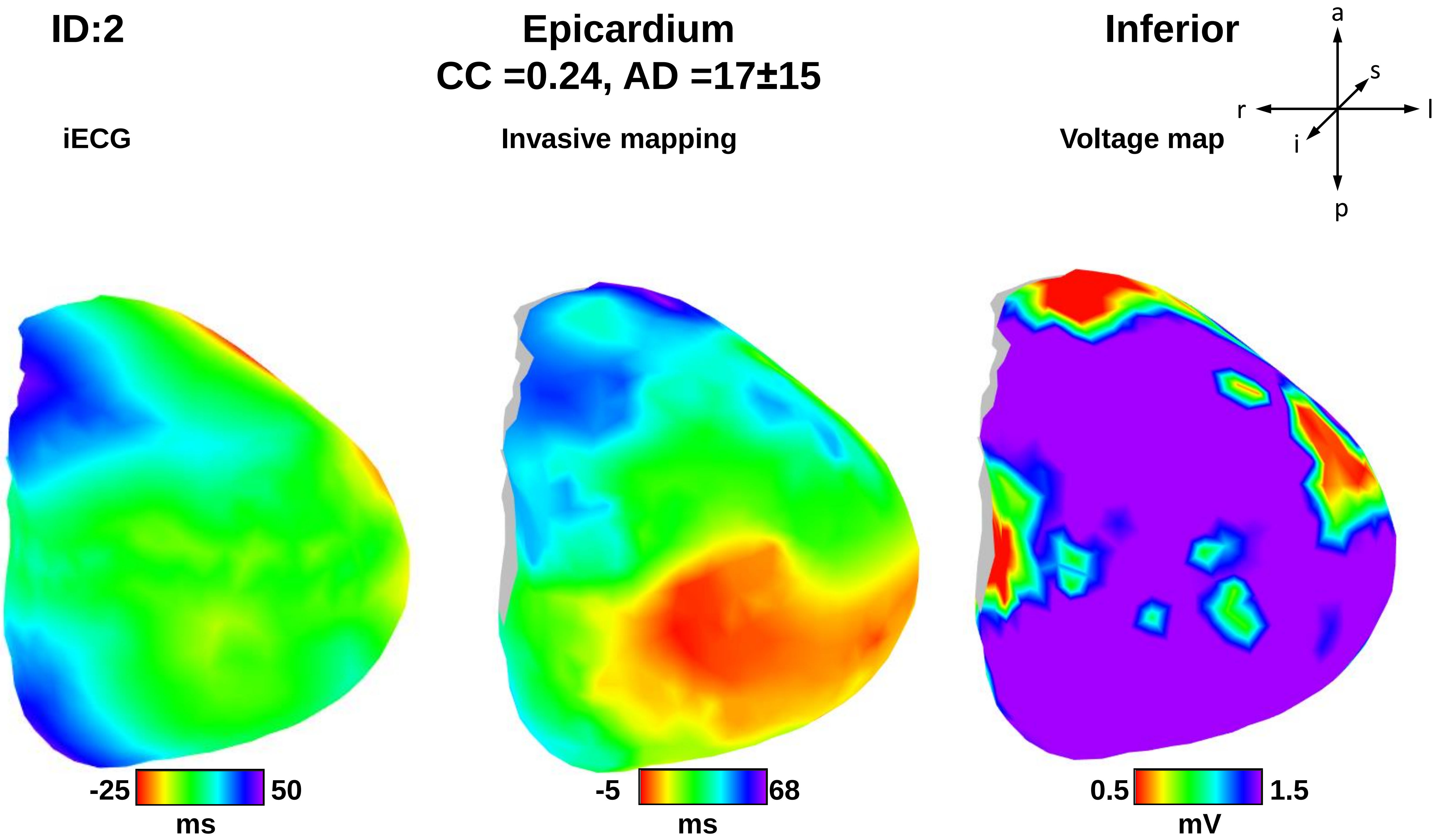
ID:2
Epicardium
CC =0.24, AD =17±15
Inferior
a
s
r
l
i
p
iECG
Invasive mapping
Voltage map
-25
50
ms
-5
68
ms
0.5
1.5
mV

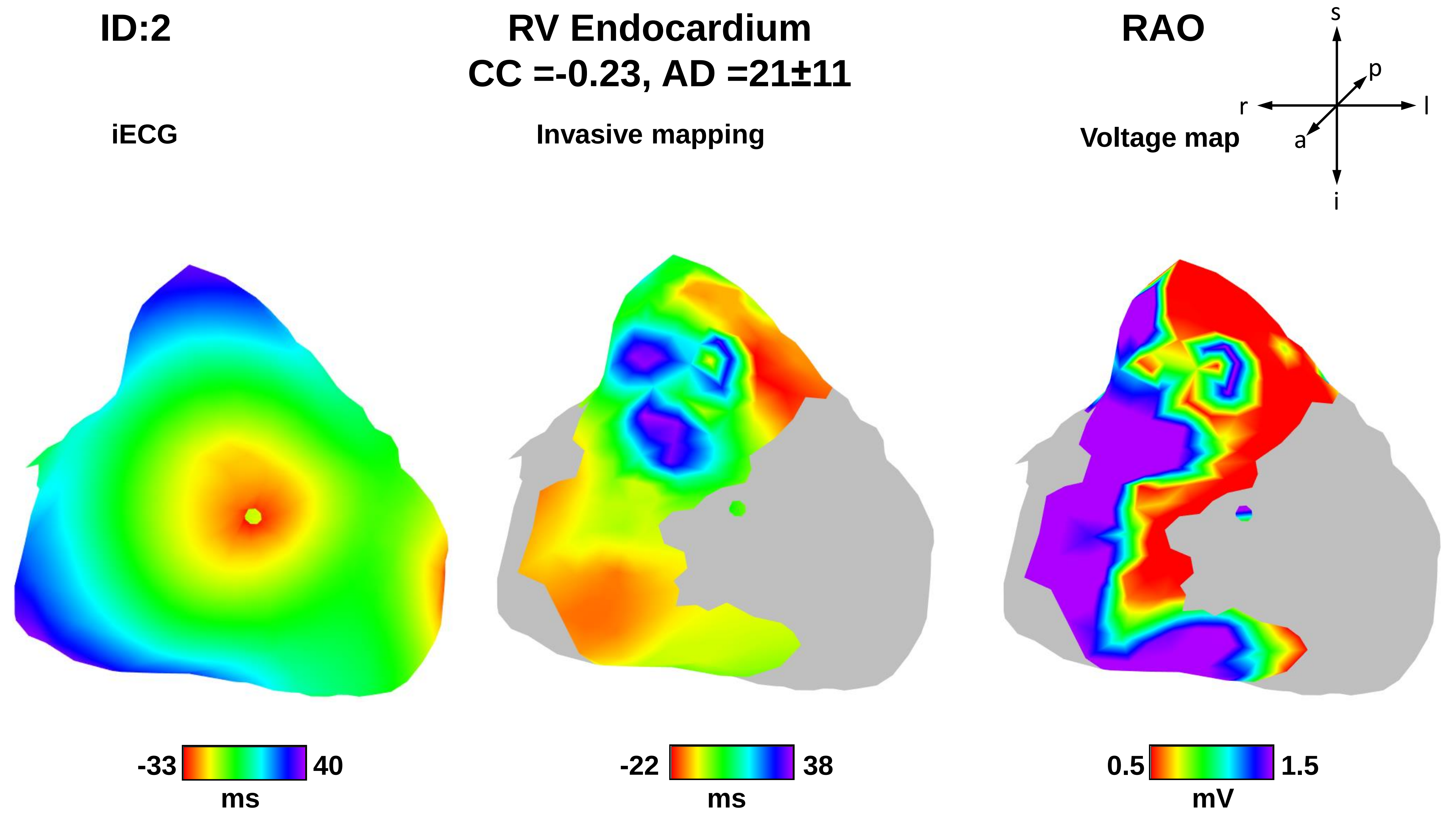

ID:2
RV Endocardium
CC =-0.23, AD =21±11
RAO
s
p
r
l
a
i
iECG
Invasive mapping
Voltage map
-33
40
ms
-22
38
ms
0.5
1.5
mV

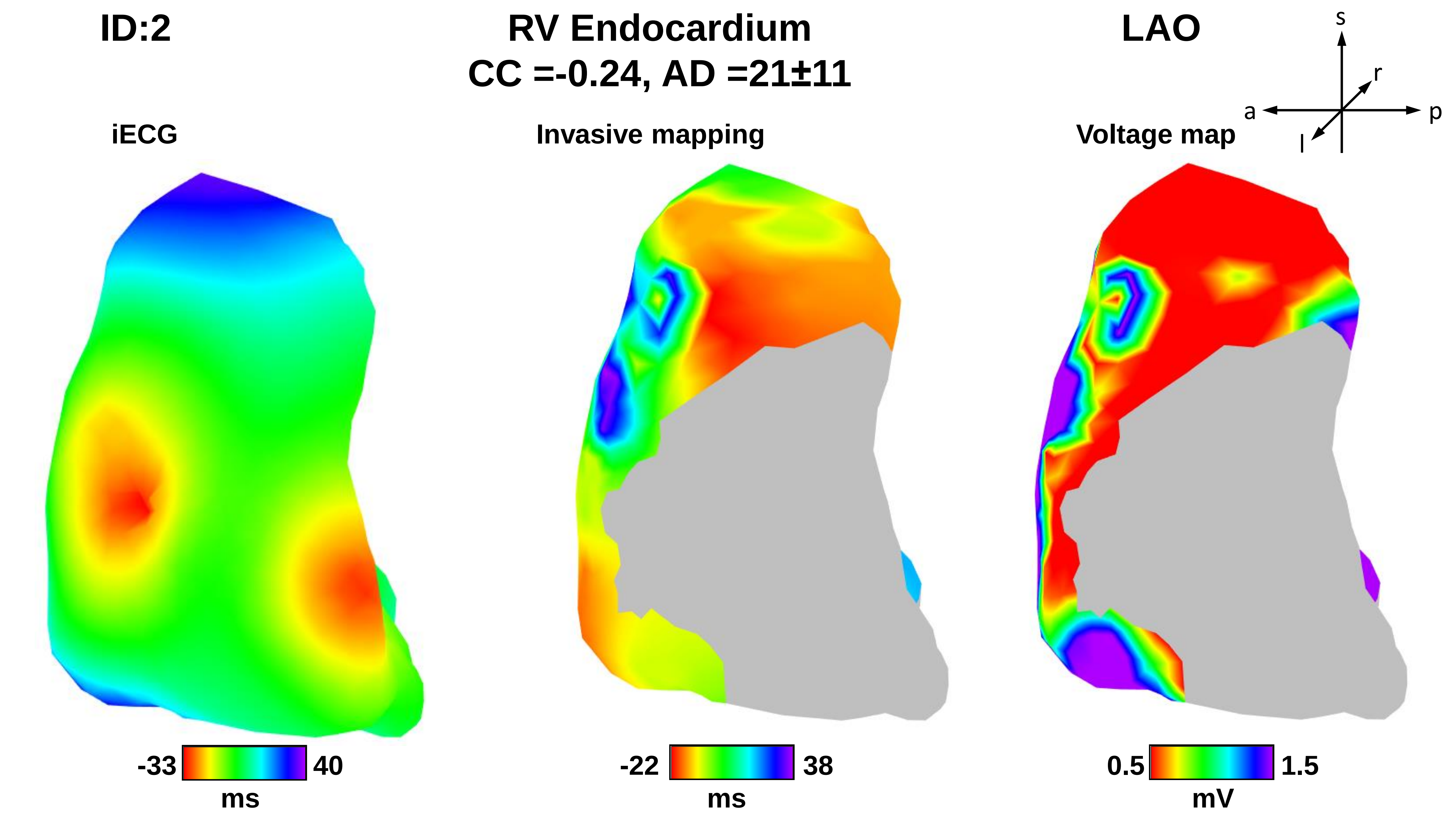
ID:2
RV Endocardium
CC =-0.24, AD =21±11
LAO
s
r
a
p
l
iECG
Invasive mapping
Voltage map
-33
40
ms
-22
38
ms
0.5
1.5
mV

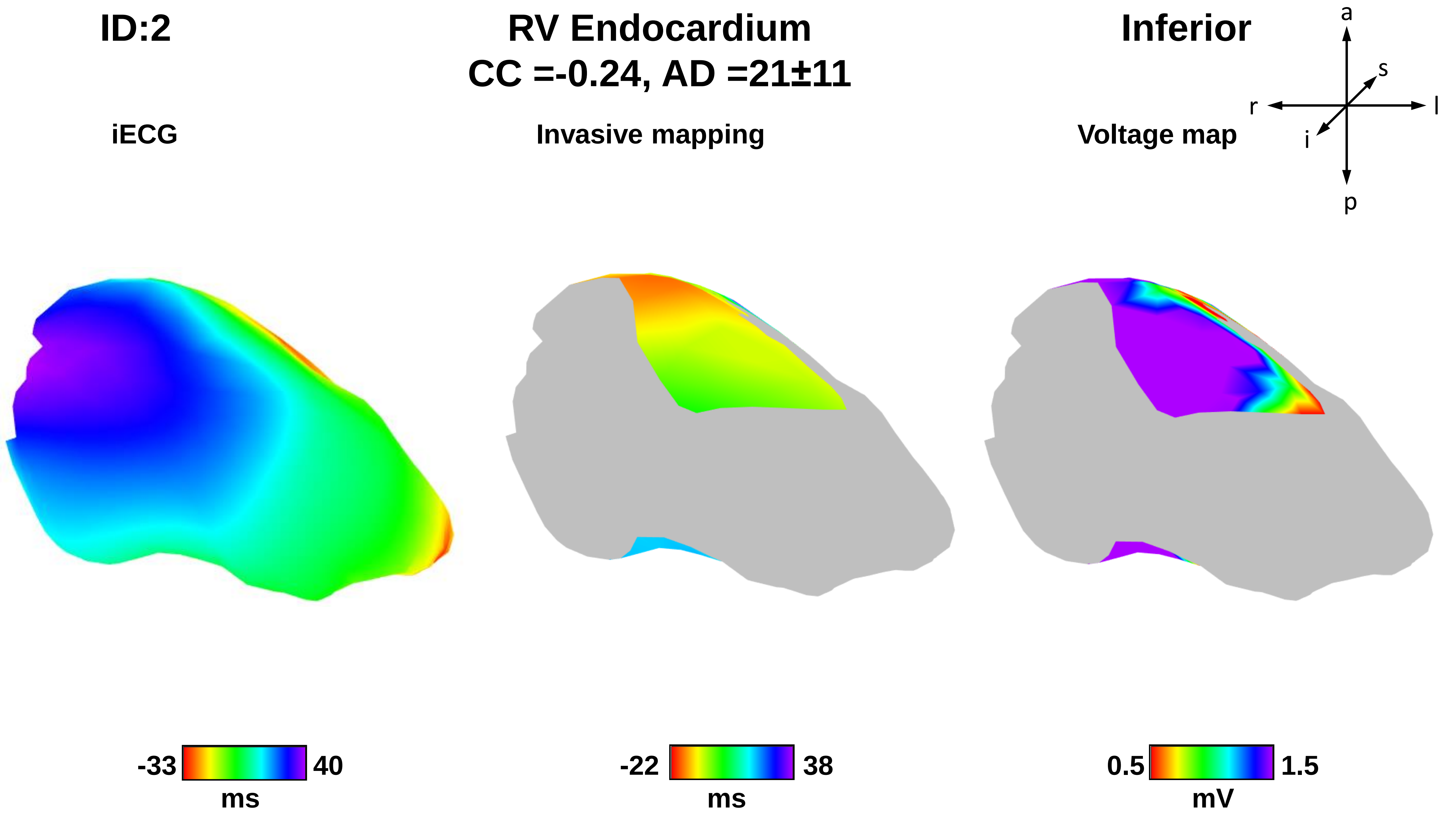
ID:2
RV Endocardium
CC =-0.24, AD =21±11
Inferior
iECG
Invasive mapping
Voltage map
a
s
r
l
i
p
-33
40
ms
-22
38
ms
0.5
1.5
mV

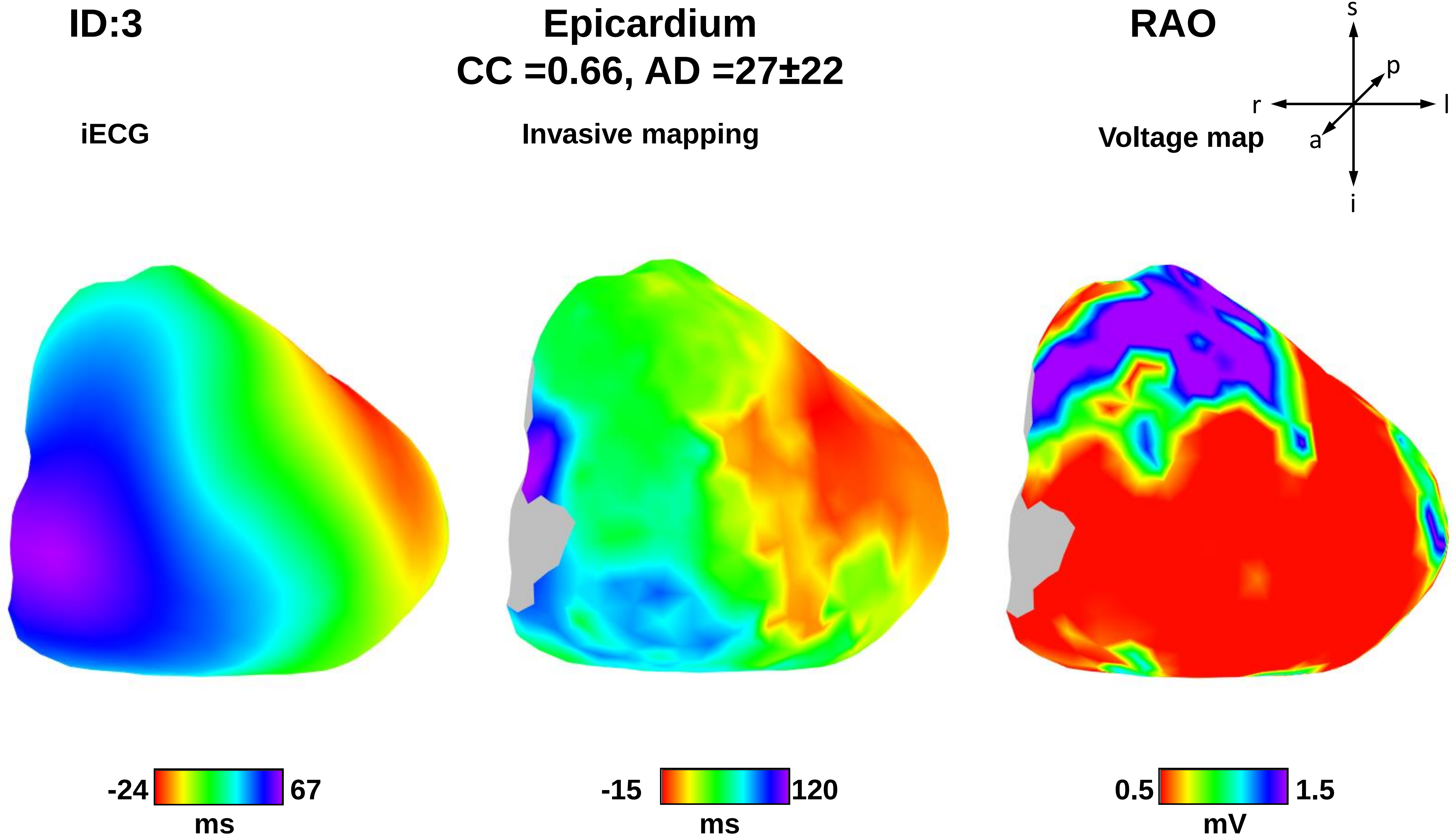
ID:3
Epicardium
CC =0.66, AD =27±22
RAO
s
p
r
l
a
i
iECG
Invasive mapping
Voltage map
-24
67
ms
-15
120
ms
0.5
1.5
mV

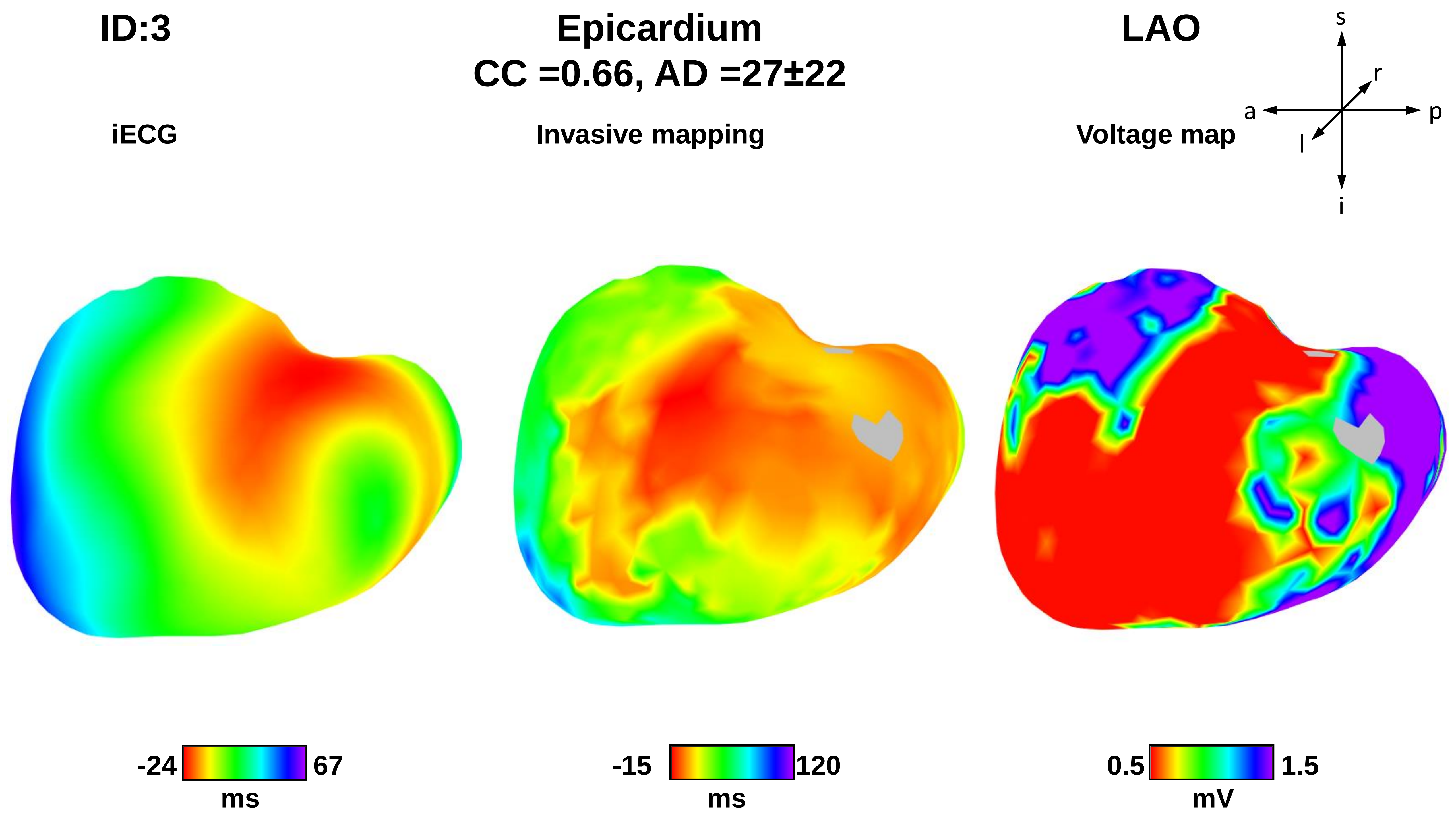

ID:3
Epicardium
CC =0.66, AD =27±22
LAO
iECG
Invasive mapping
Voltage map
s
r
a
p
l
i
-24
67
ms
-15
120
ms
0.5
1.5
mV

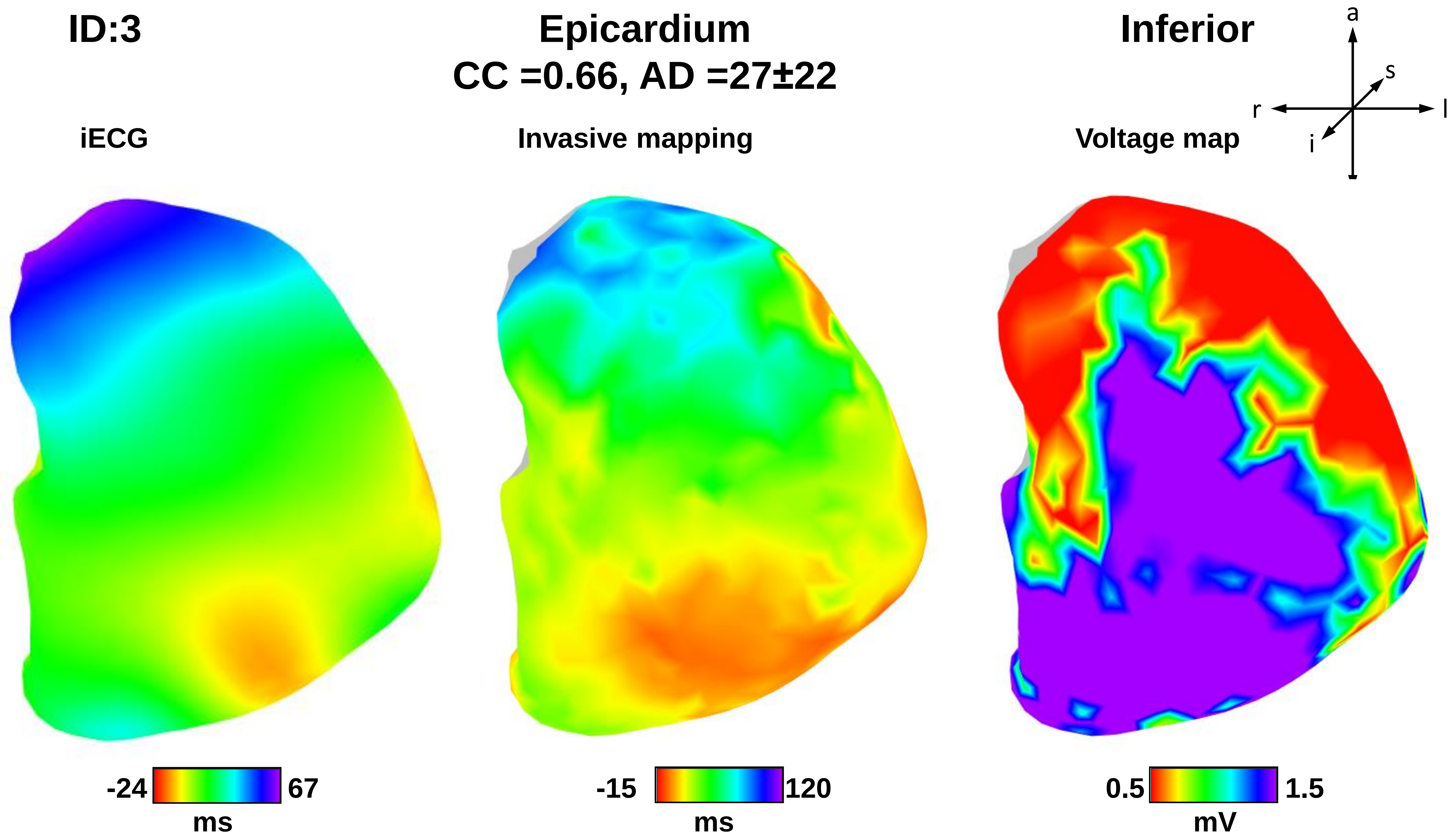
ID:3
Epicardium
CC =0.66, AD =27±22
Inferior
a
s
r
l
i
iECG
Invasive mapping
Voltage map
-24
67
ms
-15
120
ms
0.5
1.5
mV

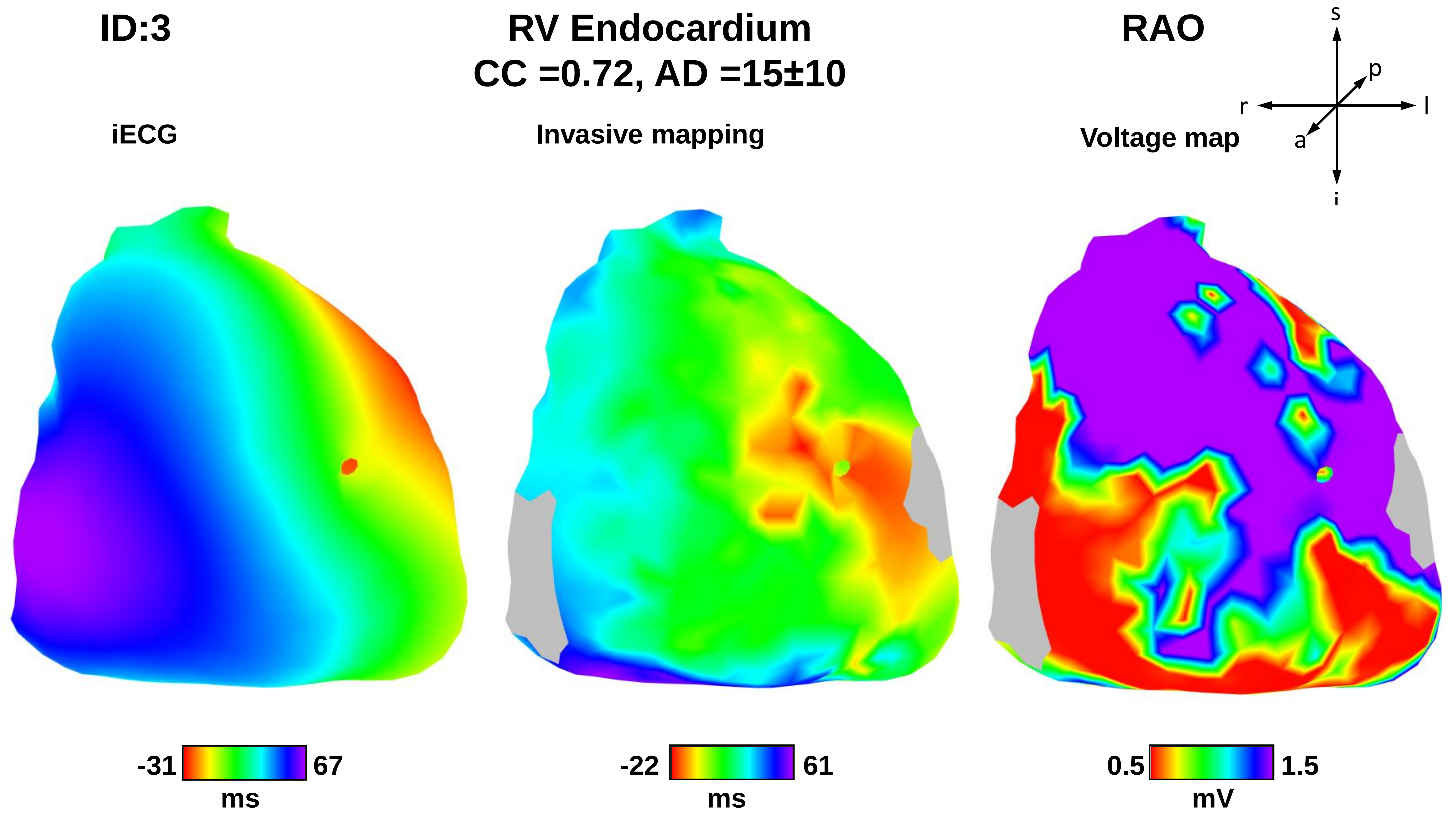
ID:3
RV Endocardium
CC =0.72, AD =15±10
RAO
s
p
r
l
a
i
iECG
Invasive mapping
Voltage map
-31
67
ms
-22
61
ms
0.5
1.5
mV

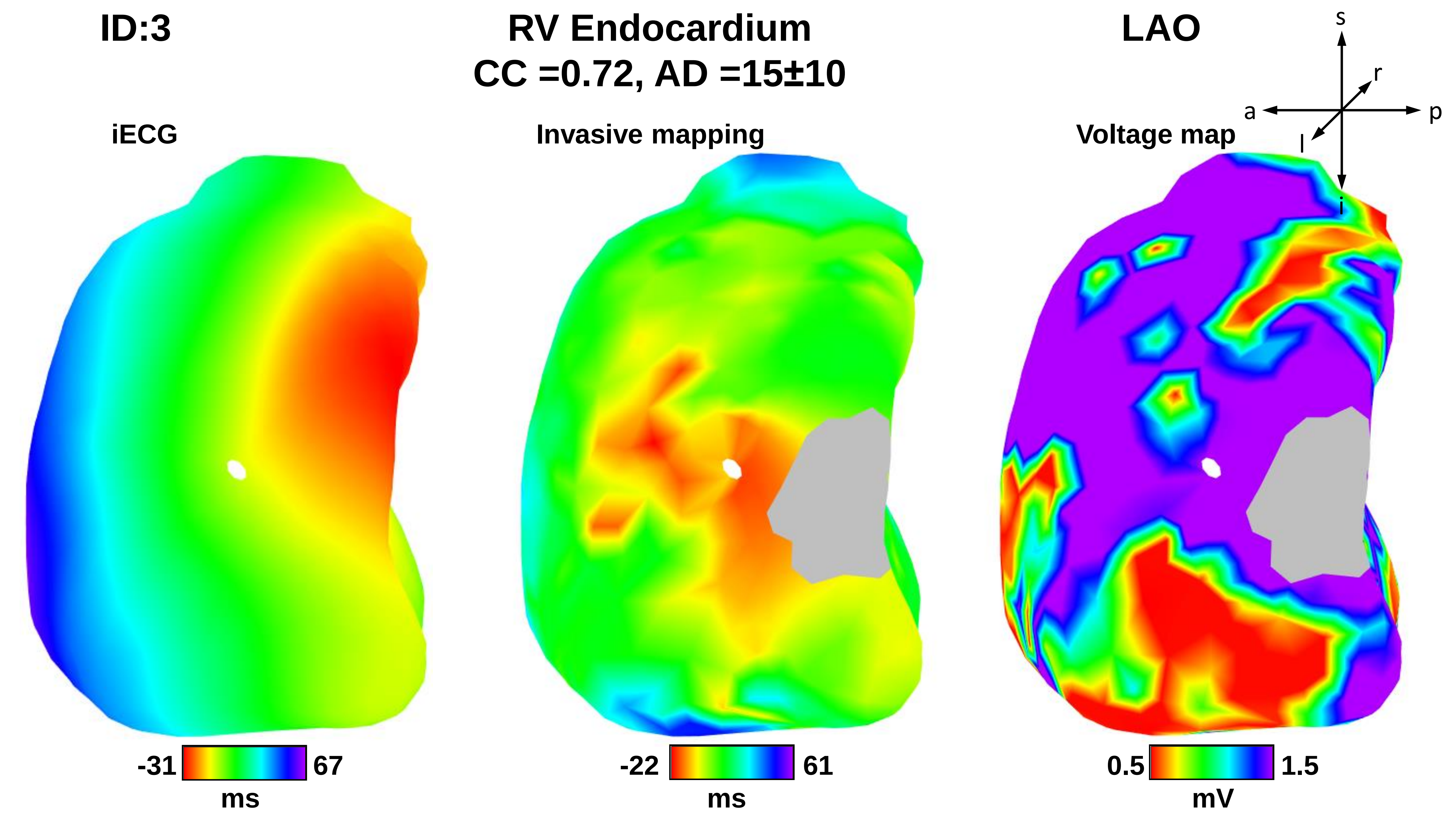
ID:3
RV Endocardium
CC =0.72, AD =15±10
LAO
s
r
a
p
l
i
iECG
Invasive mapping
Voltage map
-31
67
ms
-22
61
ms
0.5
1.5
mV

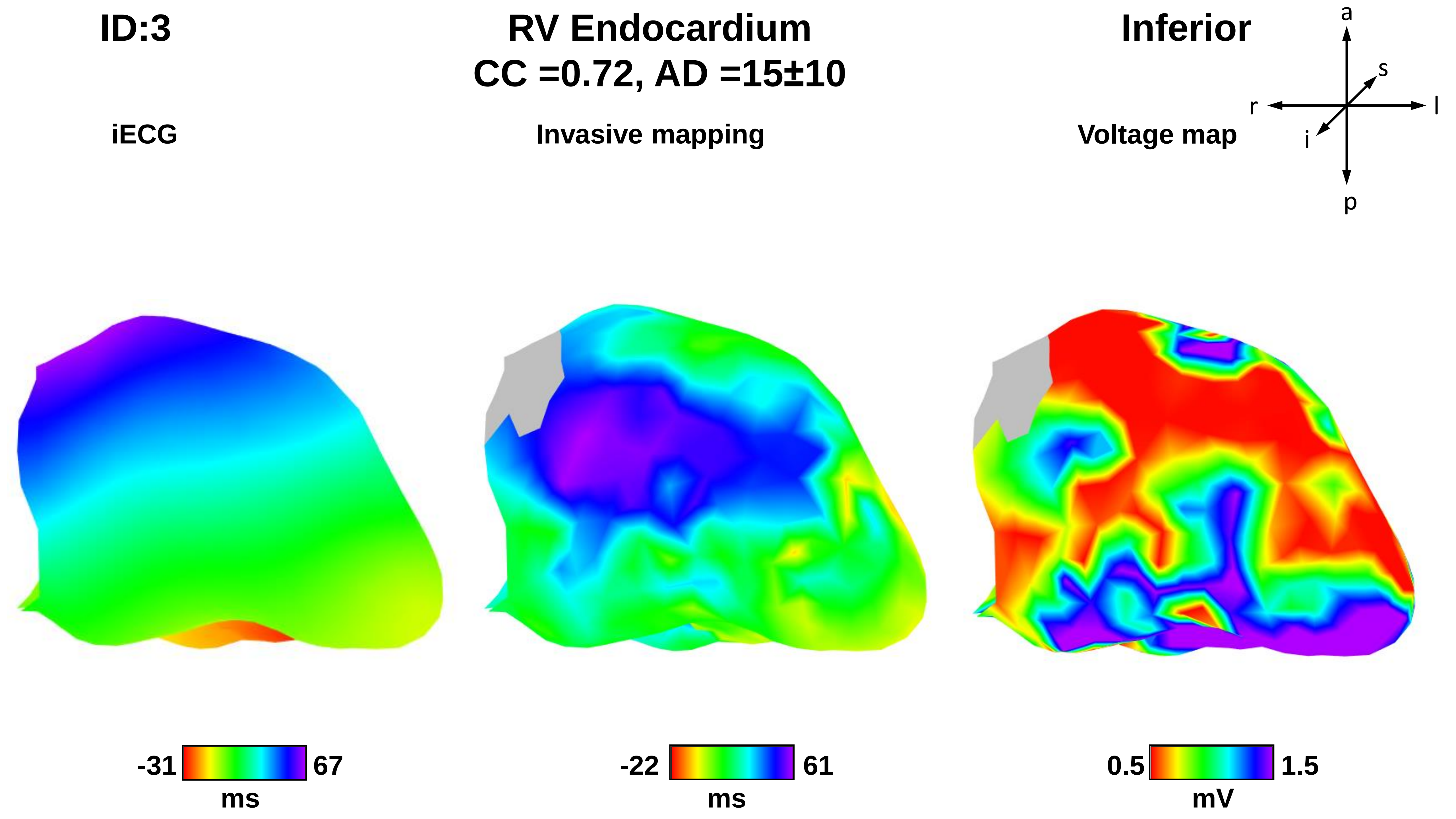
ID:3
RV Endocardium
CC =0.72, AD =15±10
Inferior
a
s
r
l
i
p
iECG
Invasive mapping
Voltage map
-31
67
ms
-22
61
ms
0.5
1.5
mV

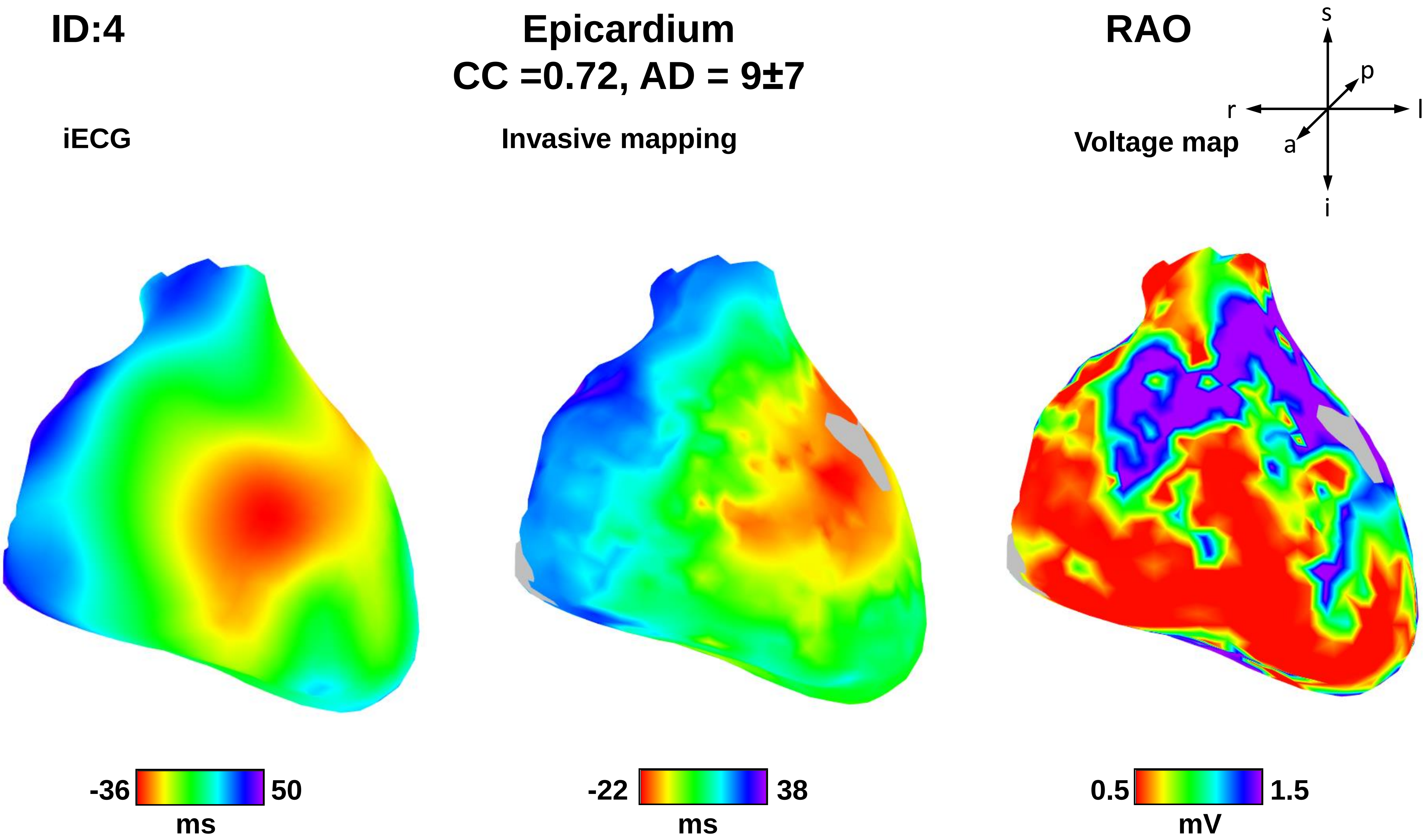
ID:4
Epicardium
CC =0.72, AD = 9±7
RAO
s
p
r
l
a
i
iECG
Invasive mapping
Voltage map
-36
50
ms
-22
38
ms
0.5
1.5
mV

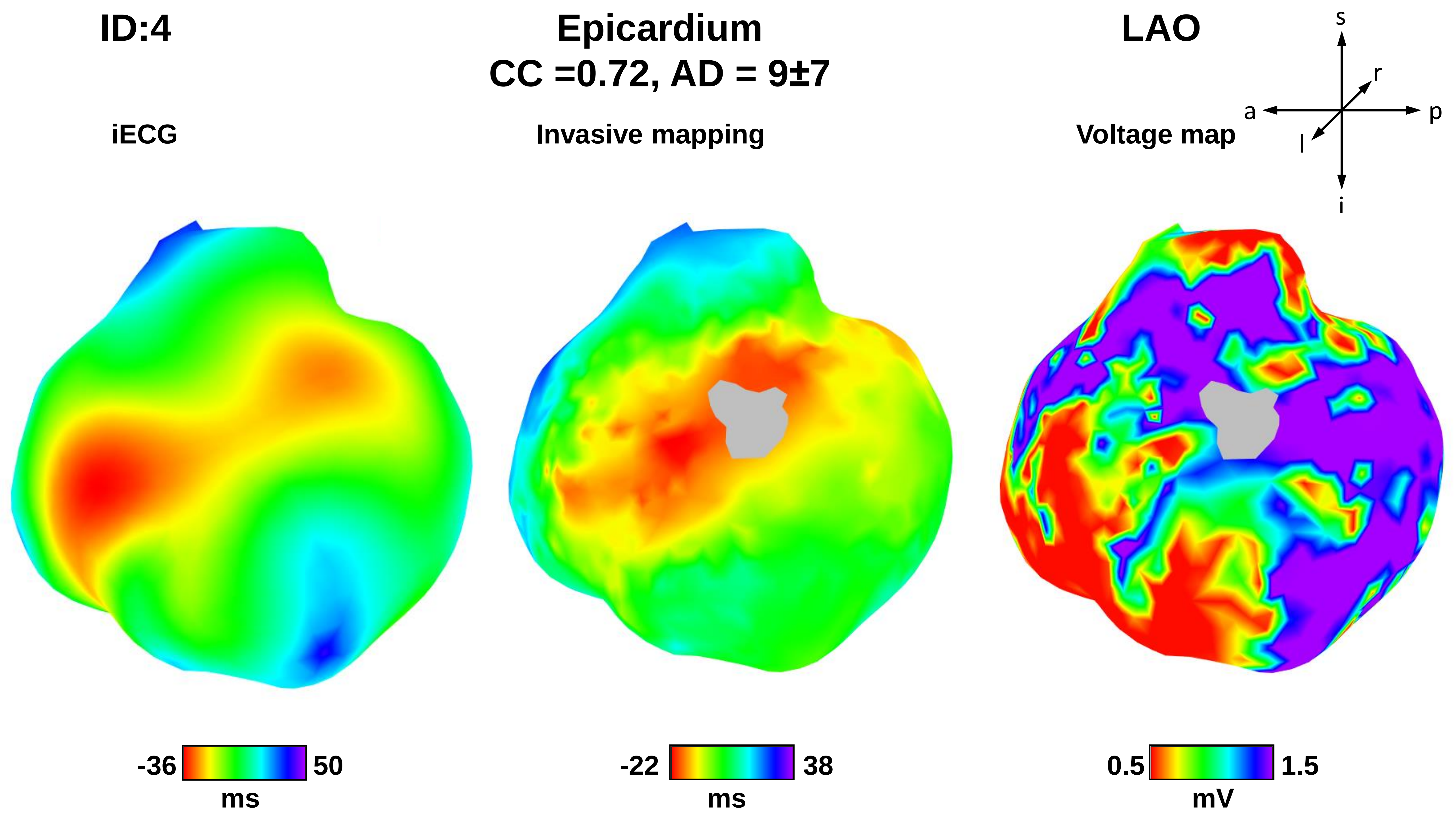

ID:4
Epicardium
CC =0.72, AD = 9±7
LAO
s
r
a
p
l
i
iECG
Invasive mapping
Voltage map
-36
50
ms
-22
38
ms
0.5
1.5
mV

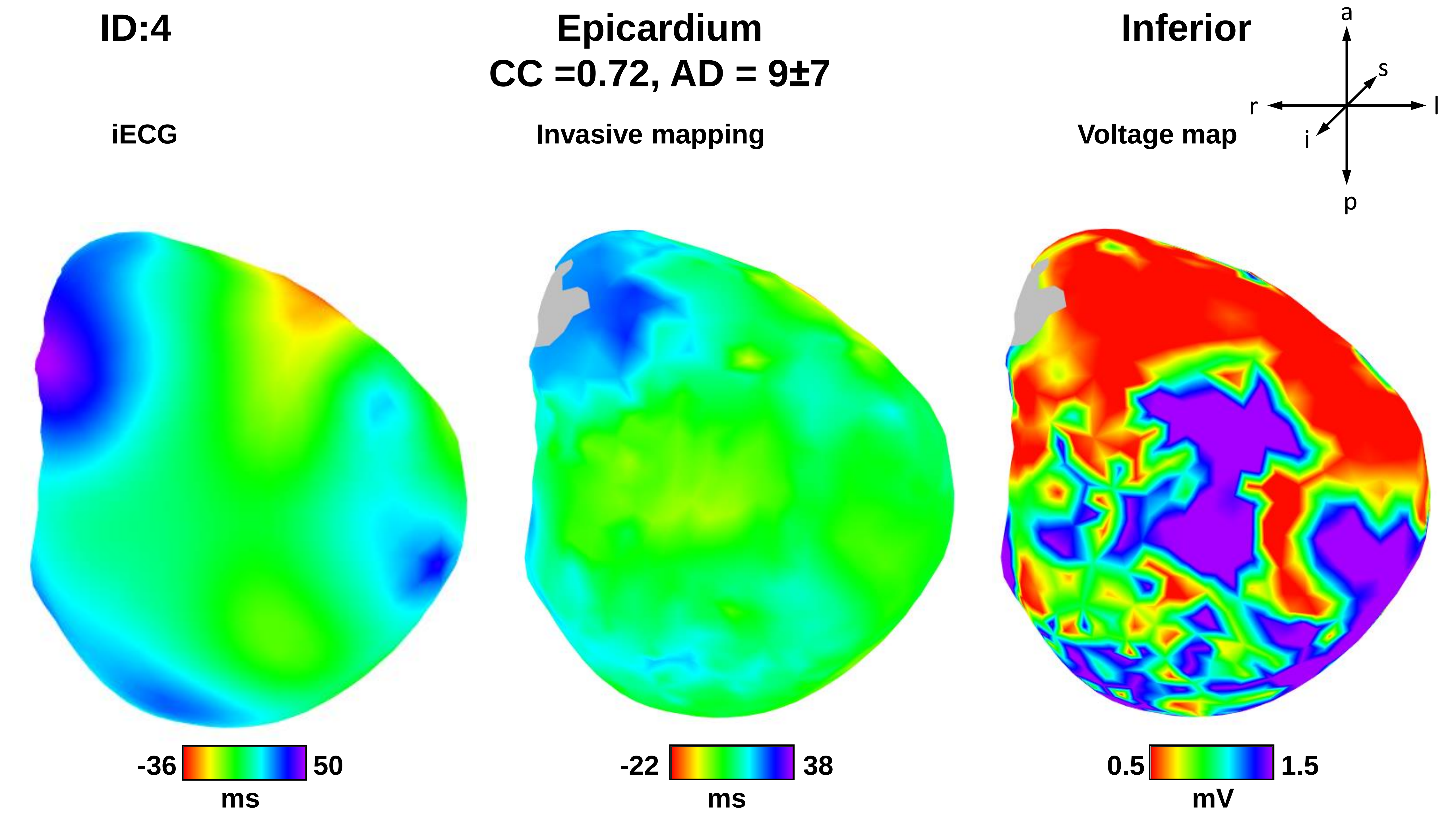

ID:4
Epicardium
CC =0.72, AD = 9±7
Inferior
a
s
r
l
i
p
iECG
Invasive mapping
Voltage map
-36
50
ms
-22
38
ms
0.5
1.5
mV

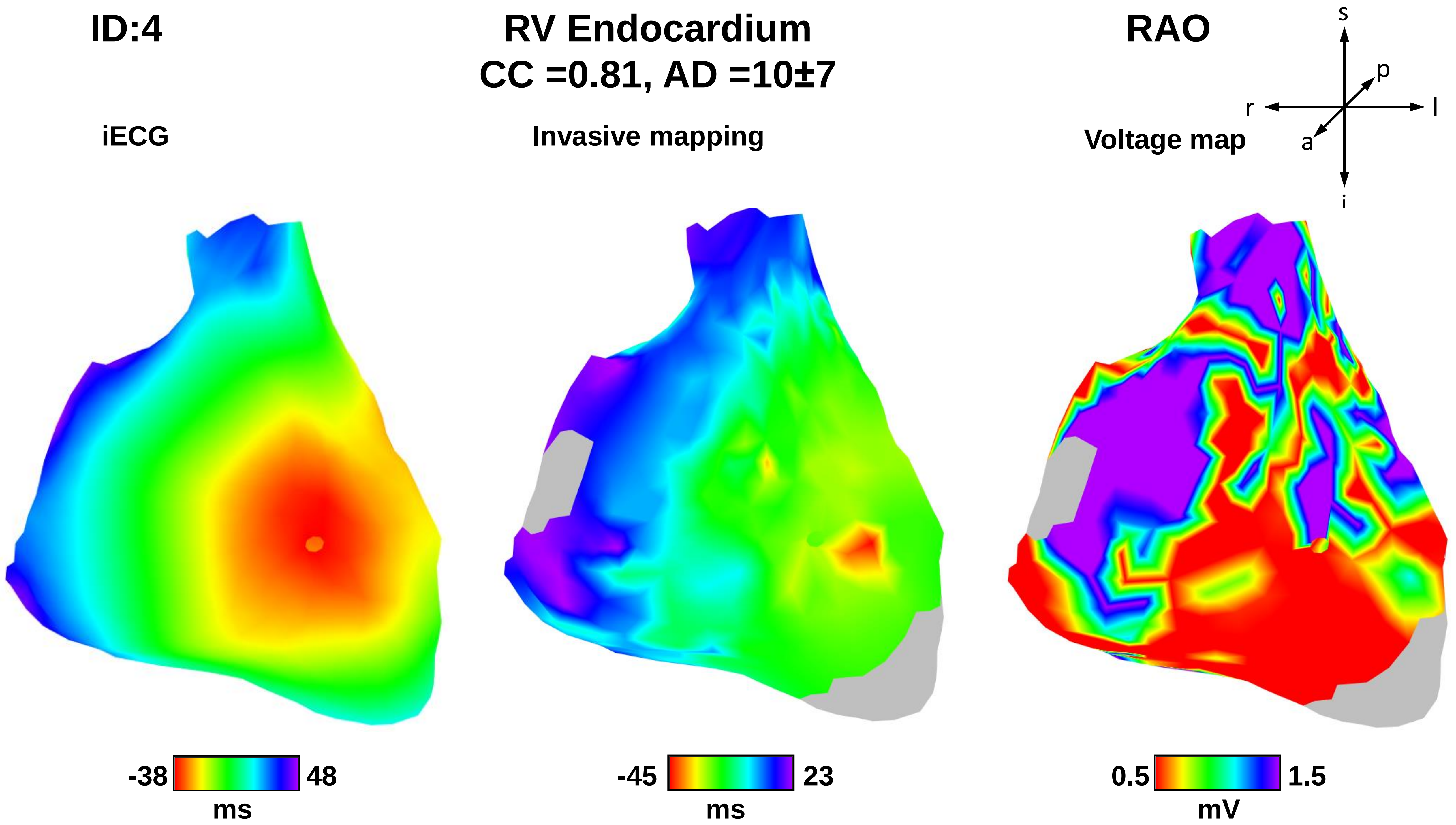
ID:4
RV Endocardium
CC =0.81, AD =10±7
RAO
s
p
r
l
a
i
iECG
Invasive mapping
Voltage map
-38
48
ms
-45
23
ms
0.5
1.5
mV

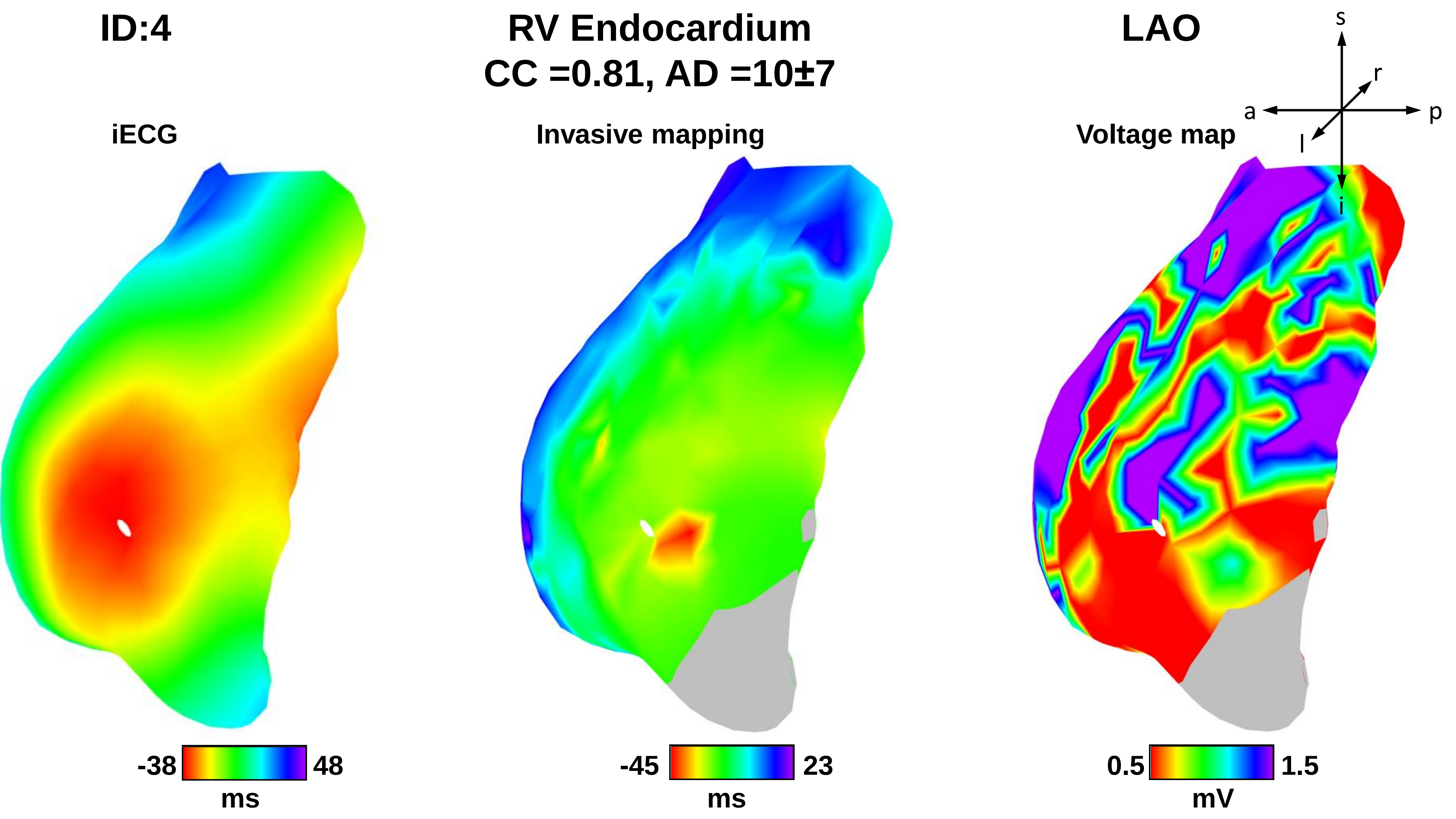
ID:4
RV Endocardium
CC =0.81, AD =10±7
LAO
iECG
Invasive mapping
Voltage map
s
r
a
p
l
i
-38
48
ms
-45
23
ms
0.5
1.5
mV

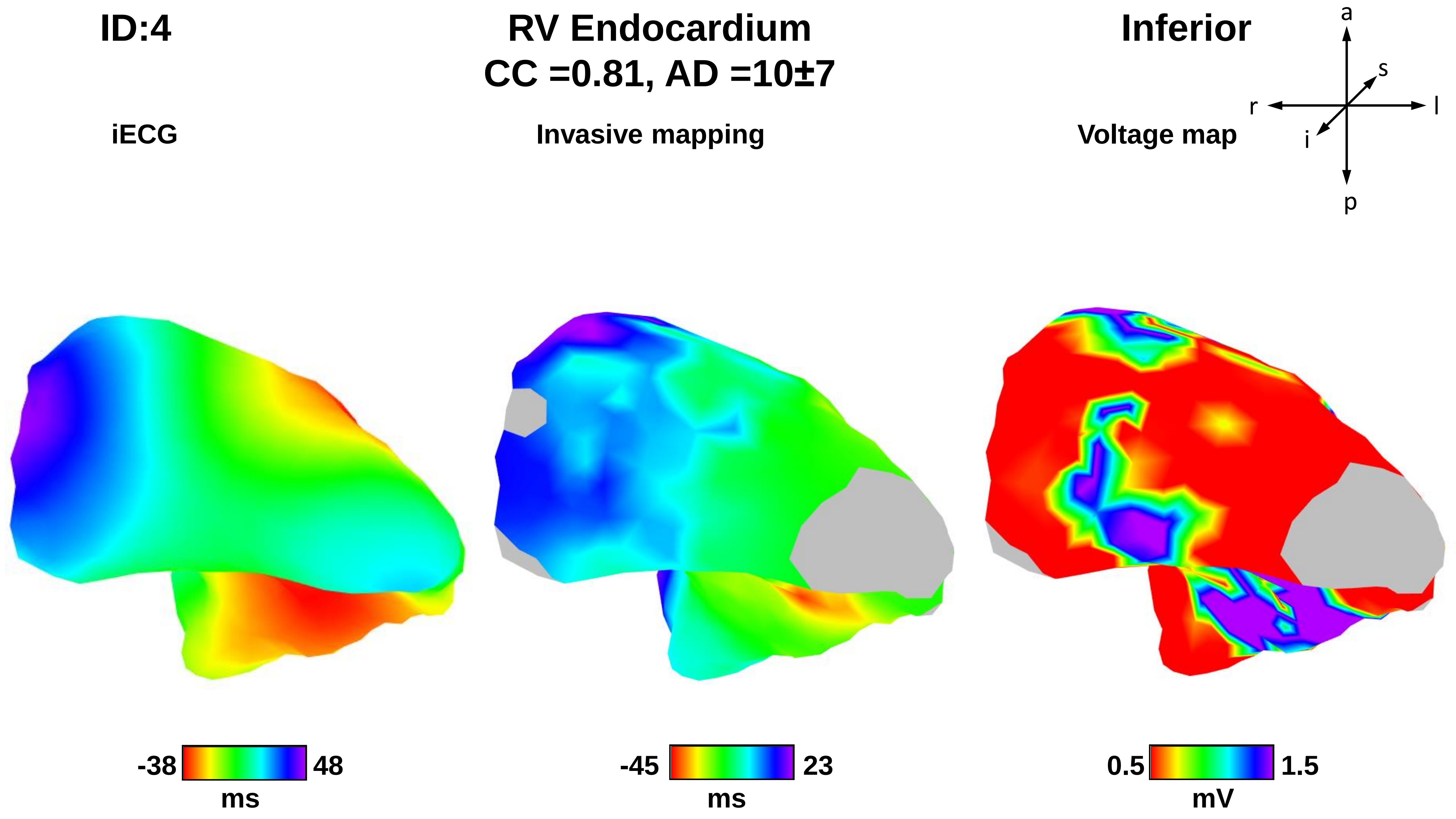
ID:4
RV Endocardium
CC =0.81, AD =10±7
Inferior
a
s
r
l
i
p
iECG
Invasive mapping
Voltage map
-38
48
ms
-45
23
ms
0.5
1.5
mV

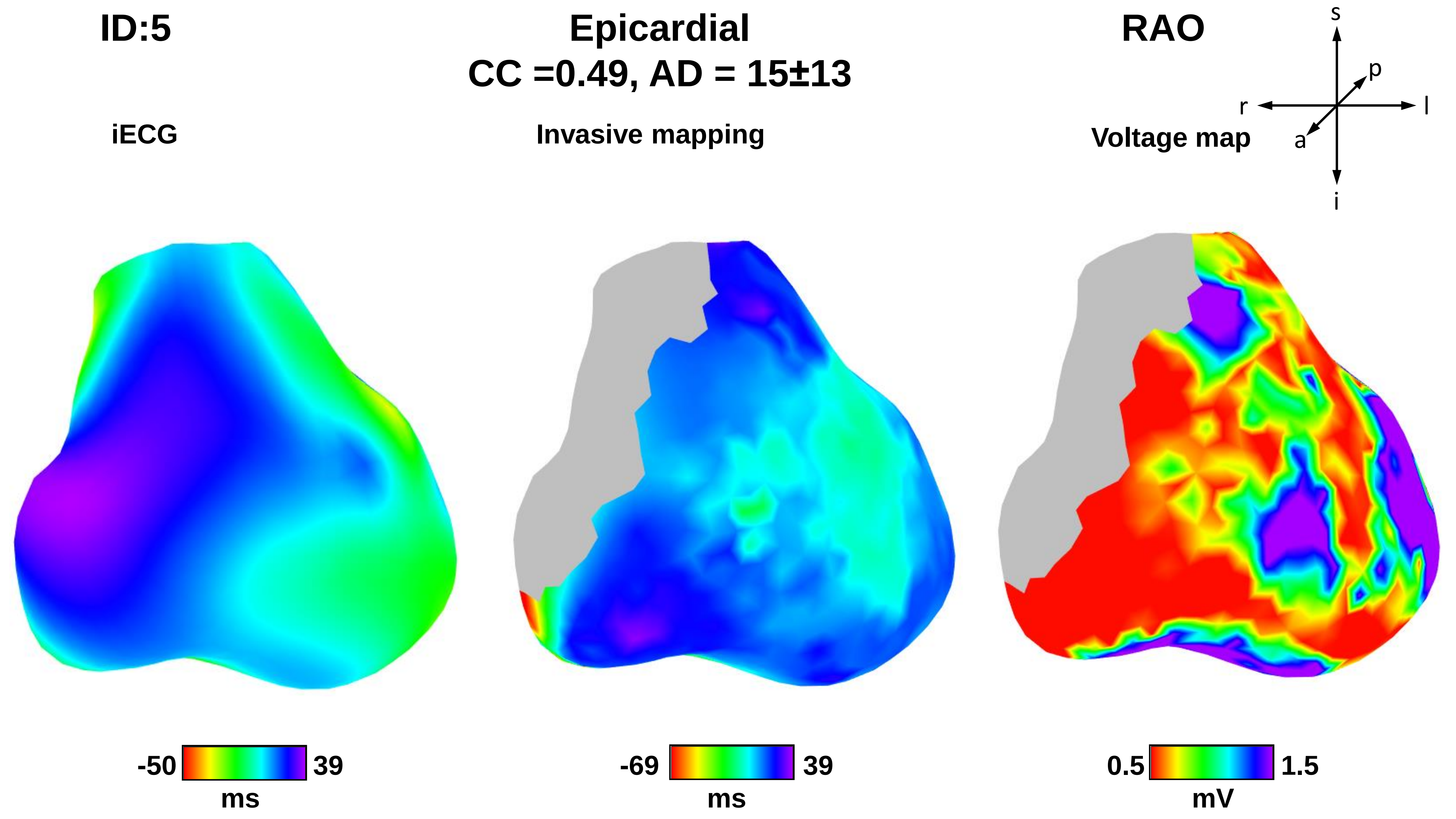
ID:5
Epicardial
CC =0.49, AD = 15±13
RAO
iECG
Invasive mapping
Voltage map
s
p
r
l
a
i
-50
39
ms
-69
39
ms
0.5
1.5
mV

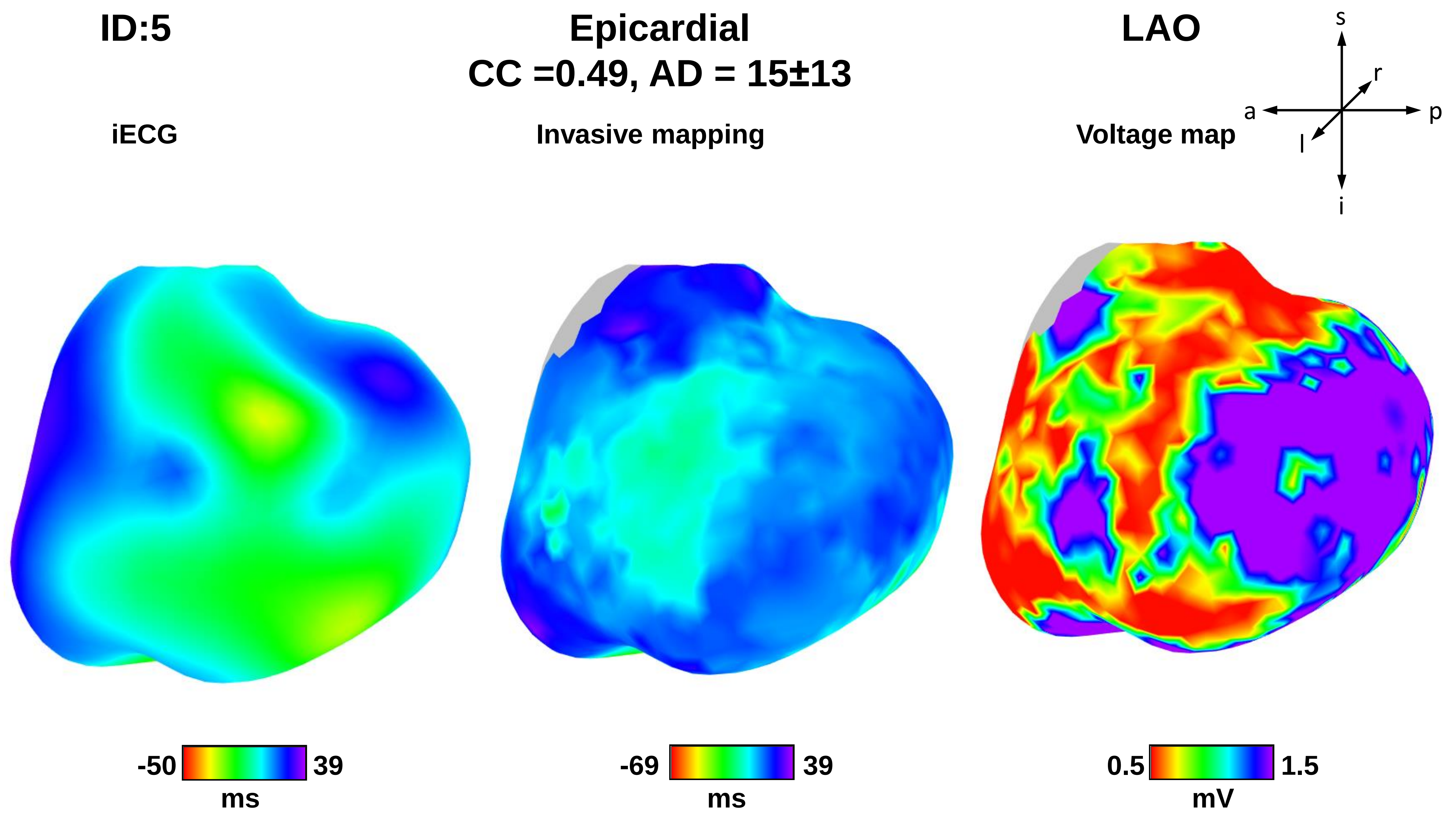
ID:5
Epicardial
CC =0.49, AD = 15±13
LAO
s
r
a
p
l
i
iECG
Invasive mapping
Voltage map
-50
39
ms
-69
39
ms
0.5
1.5
mV

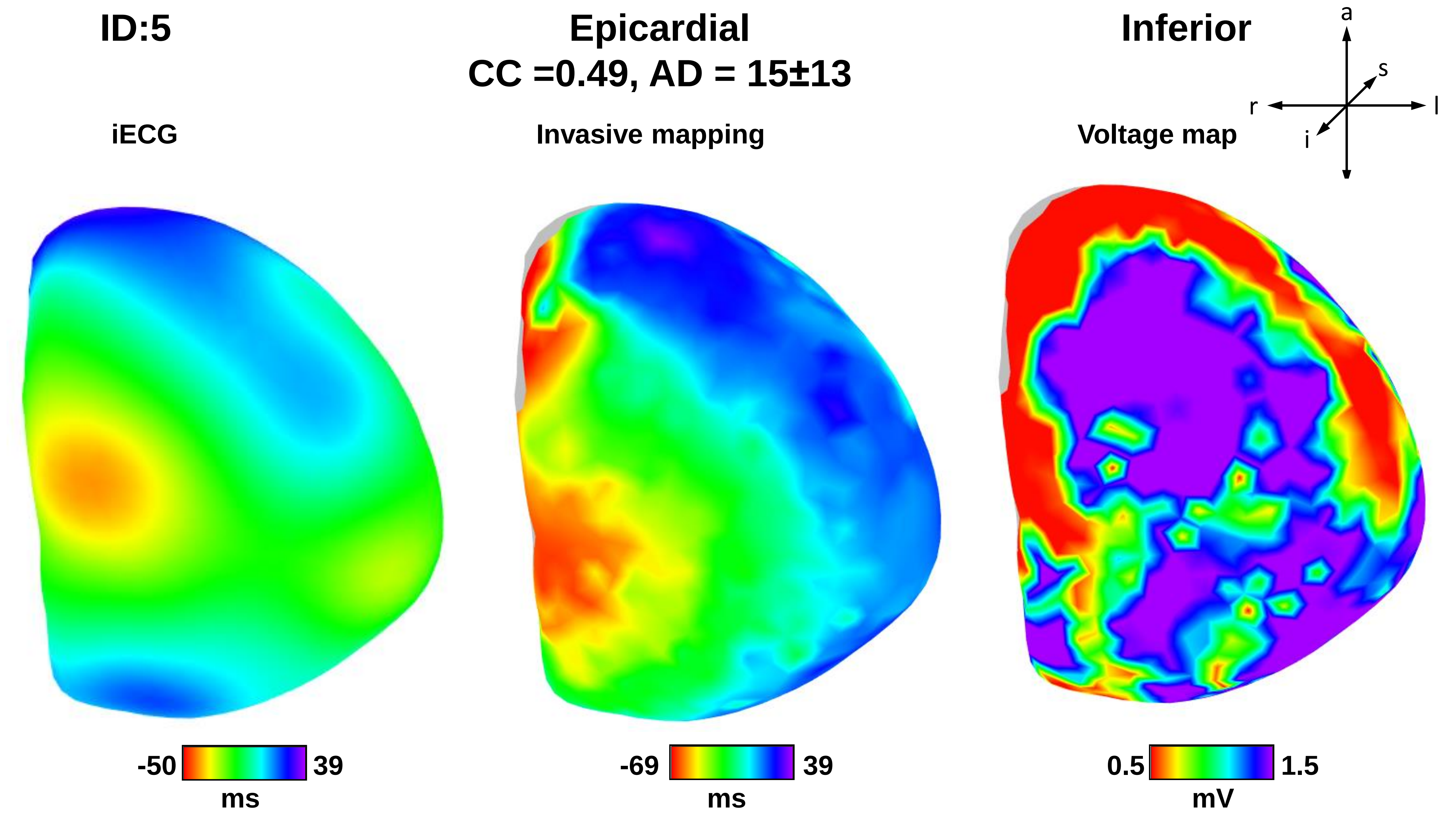

ID:5
Epicardial
CC =0.49, AD = 15±13
Inferior
a
s
r
l
i
iECG
Invasive mapping
Voltage map
-50
39
ms
-69
39
ms
0.5
1.5
mV

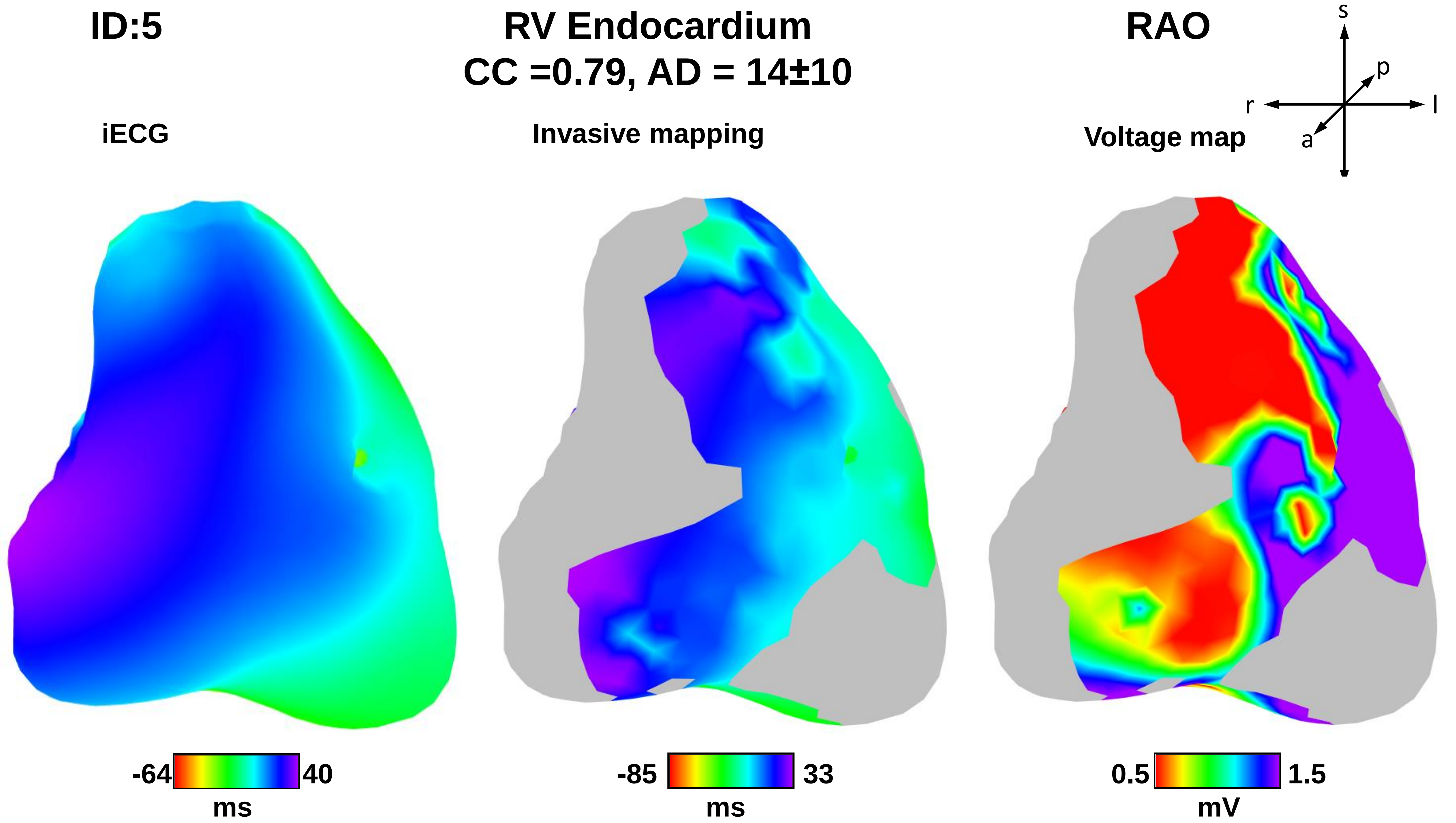
ID:5
RV Endocardium
CC =0.79, AD = 14±10
RAO
s
p
r
l
a
iECG
Invasive mapping
Voltage map
-64
40
ms
-85
33
ms
0.5
1.5
mV

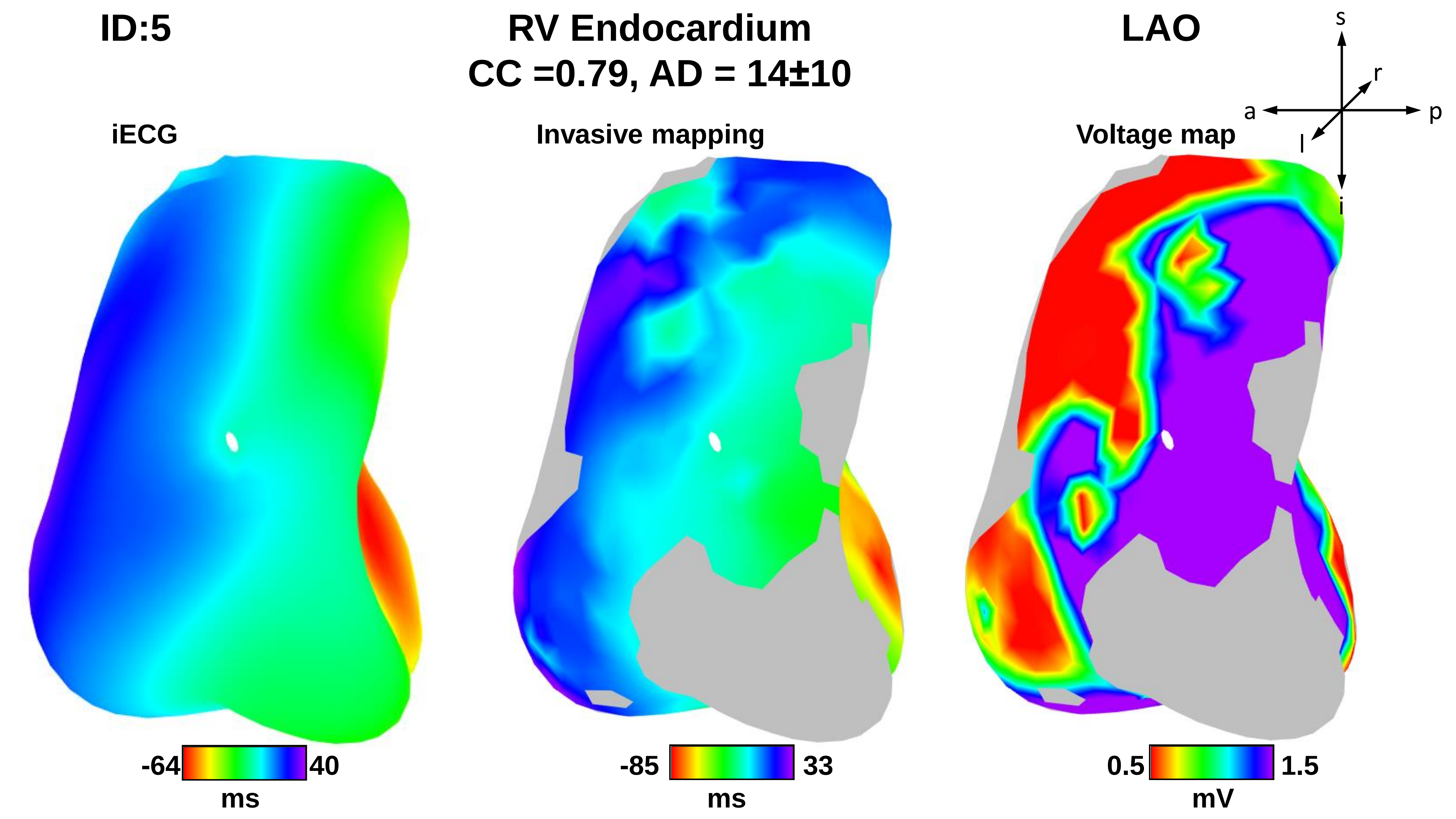
ID:5
RV Endocardium
CC =0.79, AD = 14±10
LAO
iECG
Invasive mapping
Voltage map
s
r
a
p
l
i
-64
40
ms
-85
33
ms
0.5
1.5
mV

ID:5

RV Endocardium
CC =0.79, AD = 14±10

Inferior

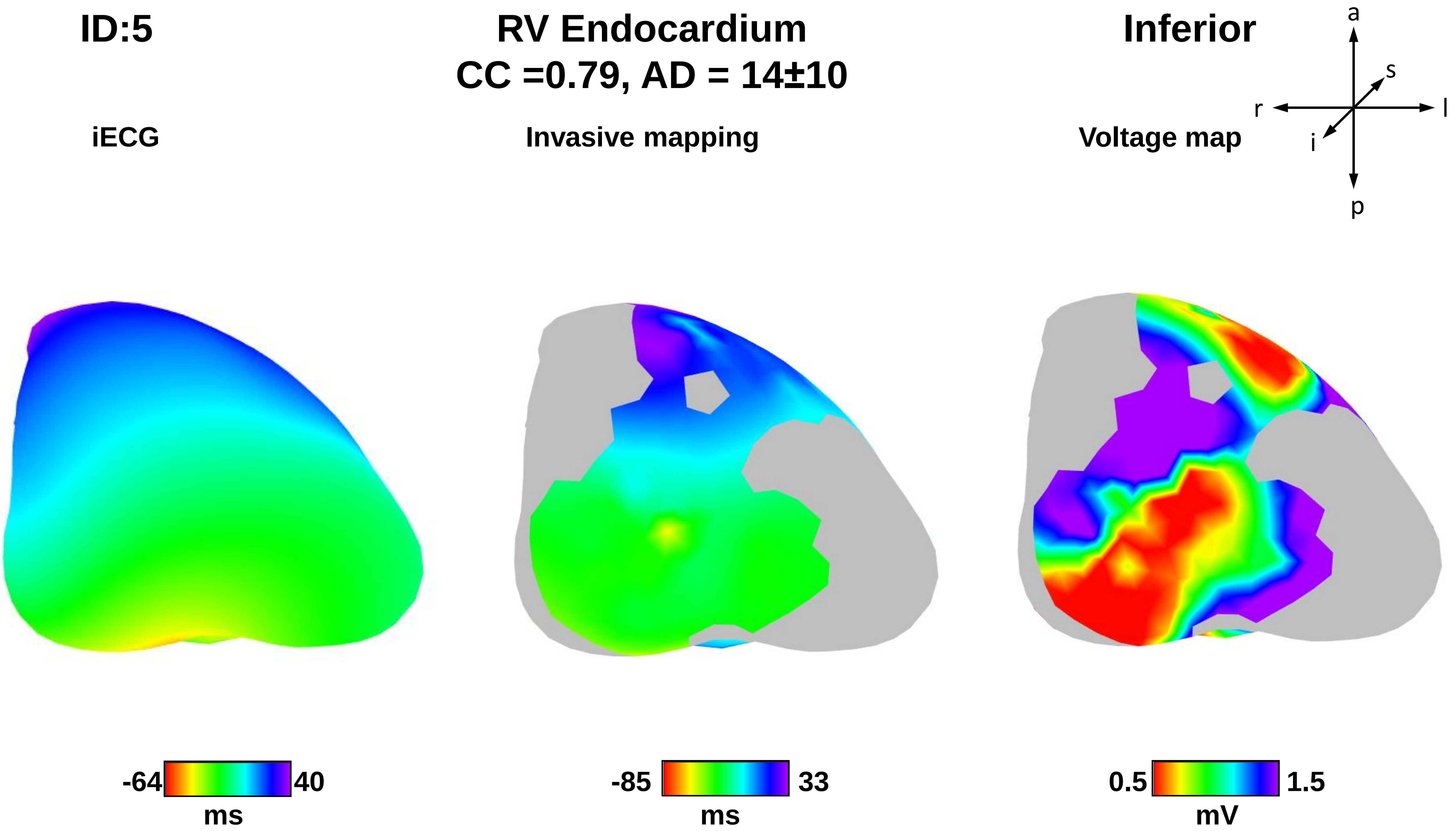

ID:6

Epicardium

CC =0.57, AD = 10±7

RAO

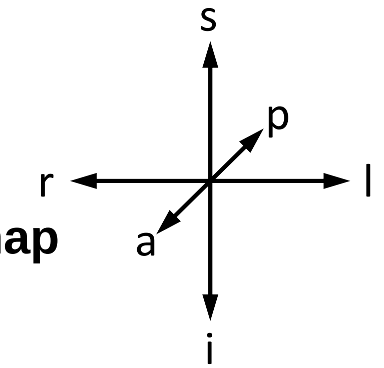

iECG

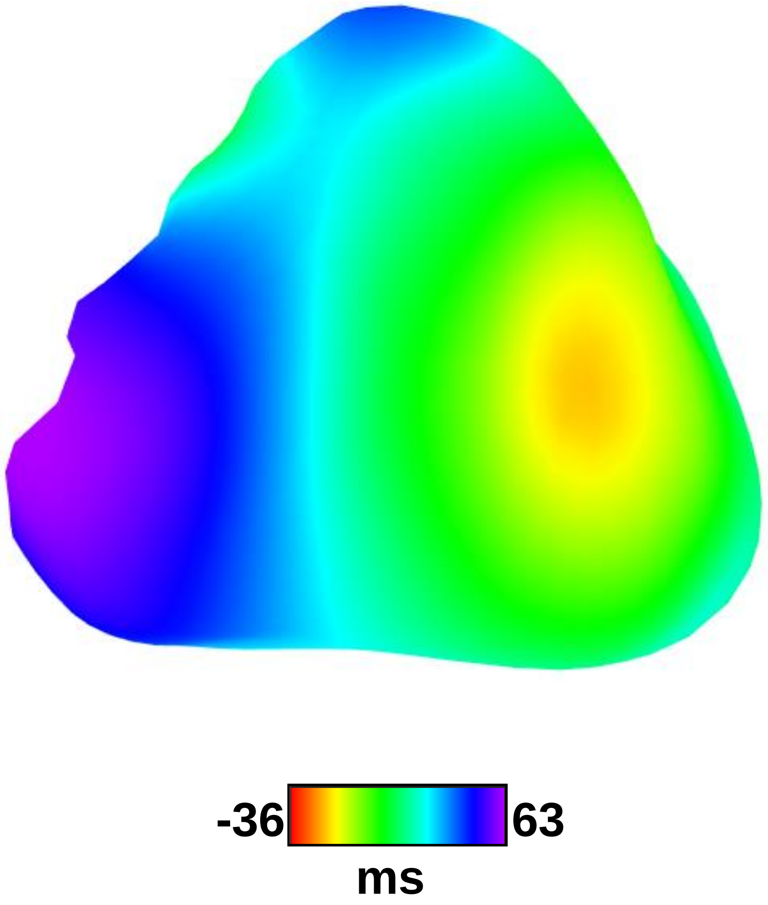

Invasive mapping

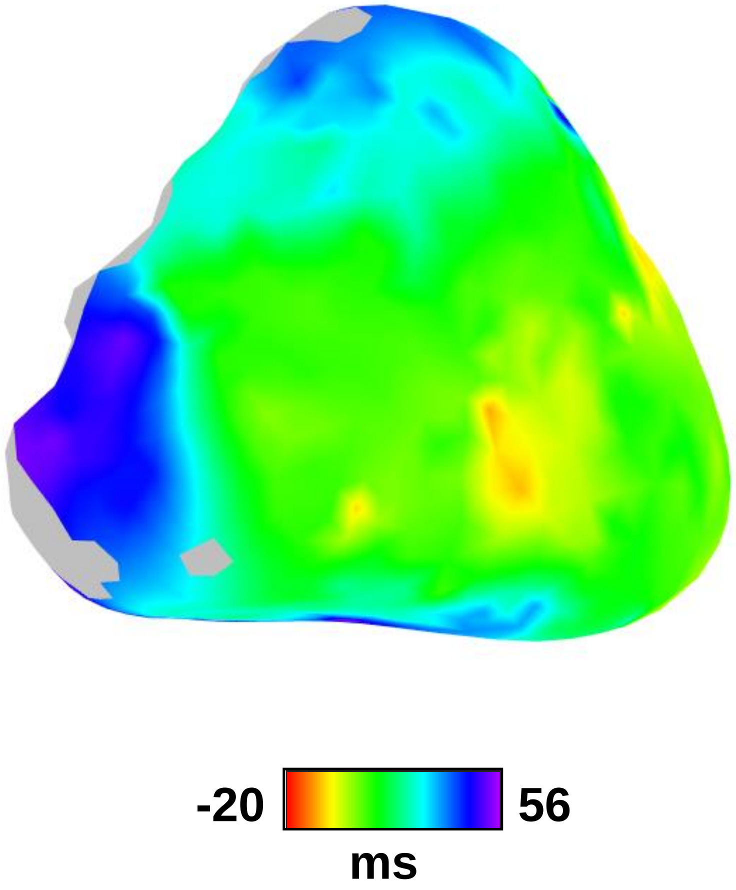

Voltage map

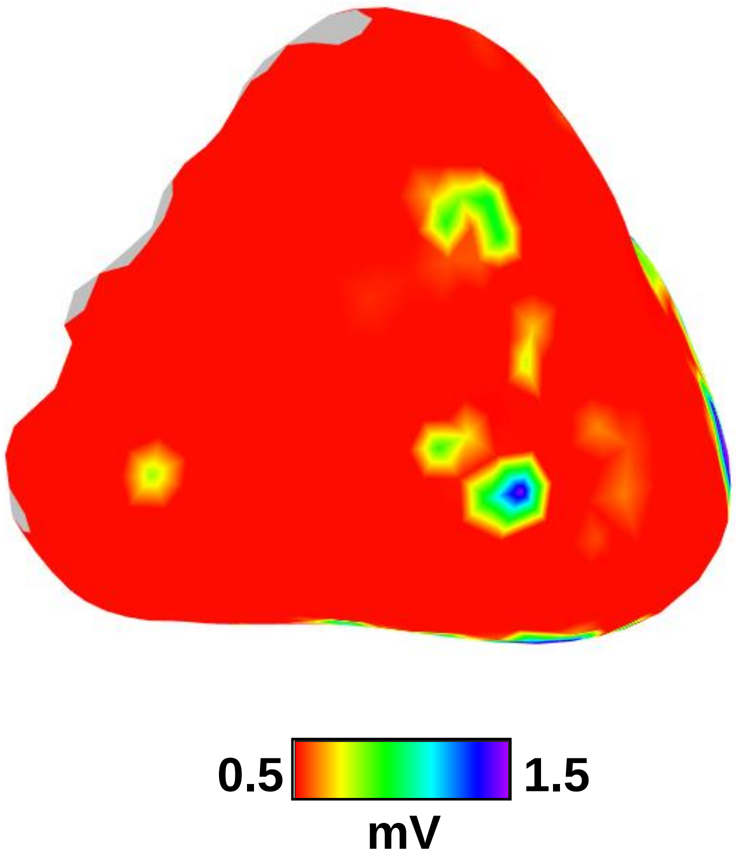

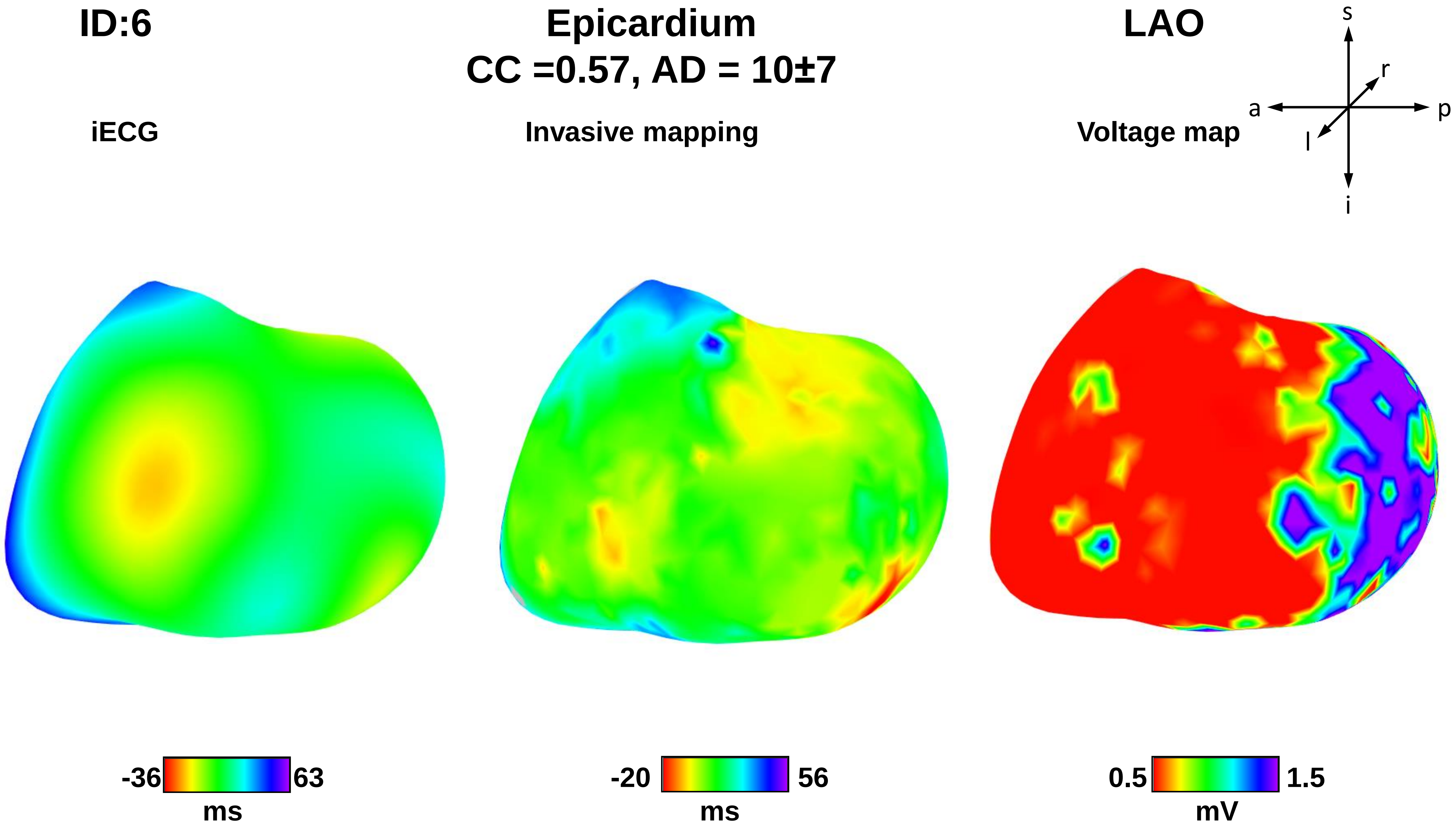
ID:6
Epicardium
CC =0.57, AD = 10±7
LAO
iECG
Invasive mapping
Voltage map
s
r
a
p
l
i
-36
63
ms
-20
56
ms
0.5
1.5
mV

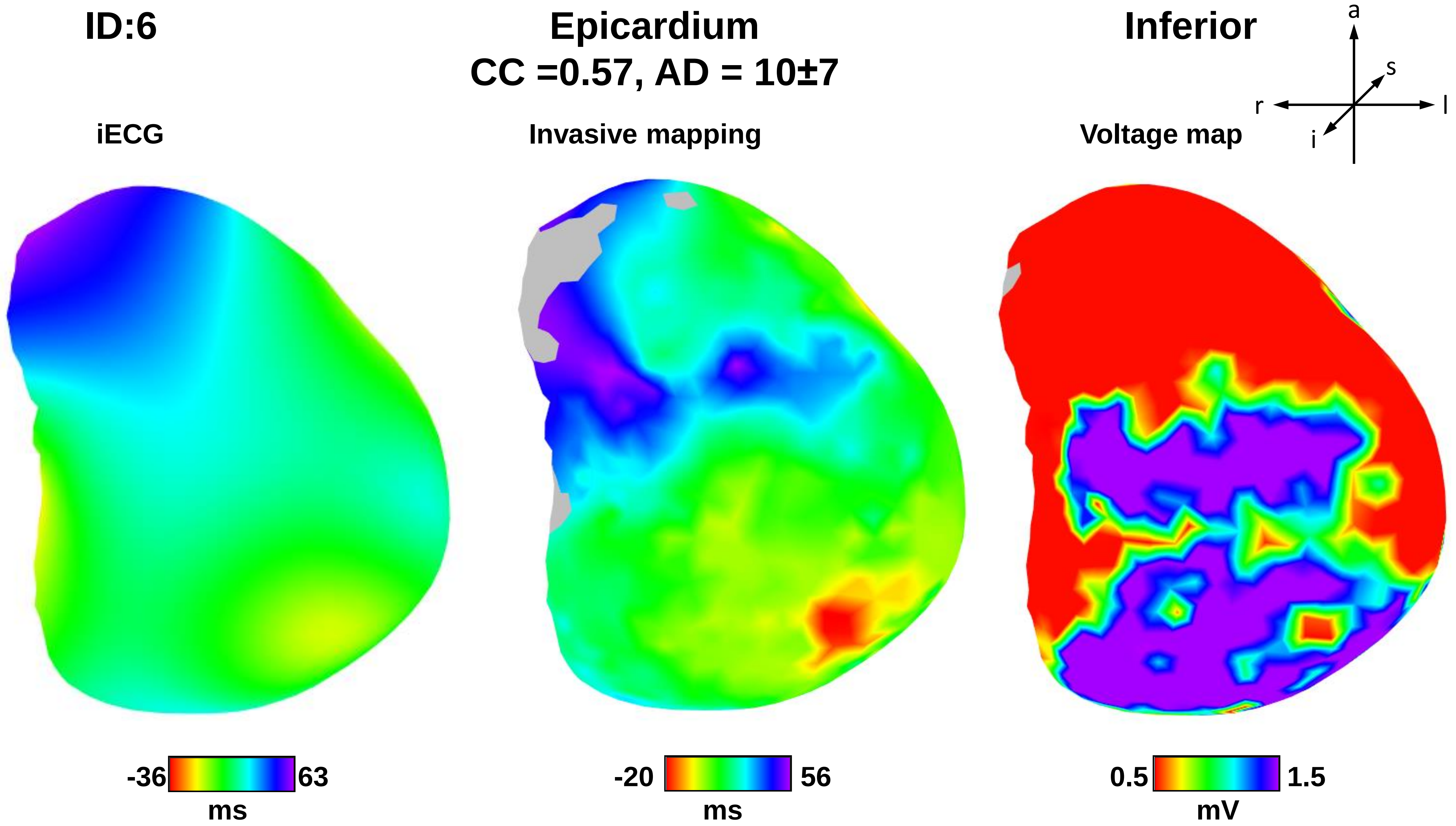
ID:6
Epicardium
CC =0.57, AD = 10±7
Inferior
a
s
r
l
i
iECG
Invasive mapping
Voltage map
-36
63
ms
-20
56
ms
0.5
1.5
mV

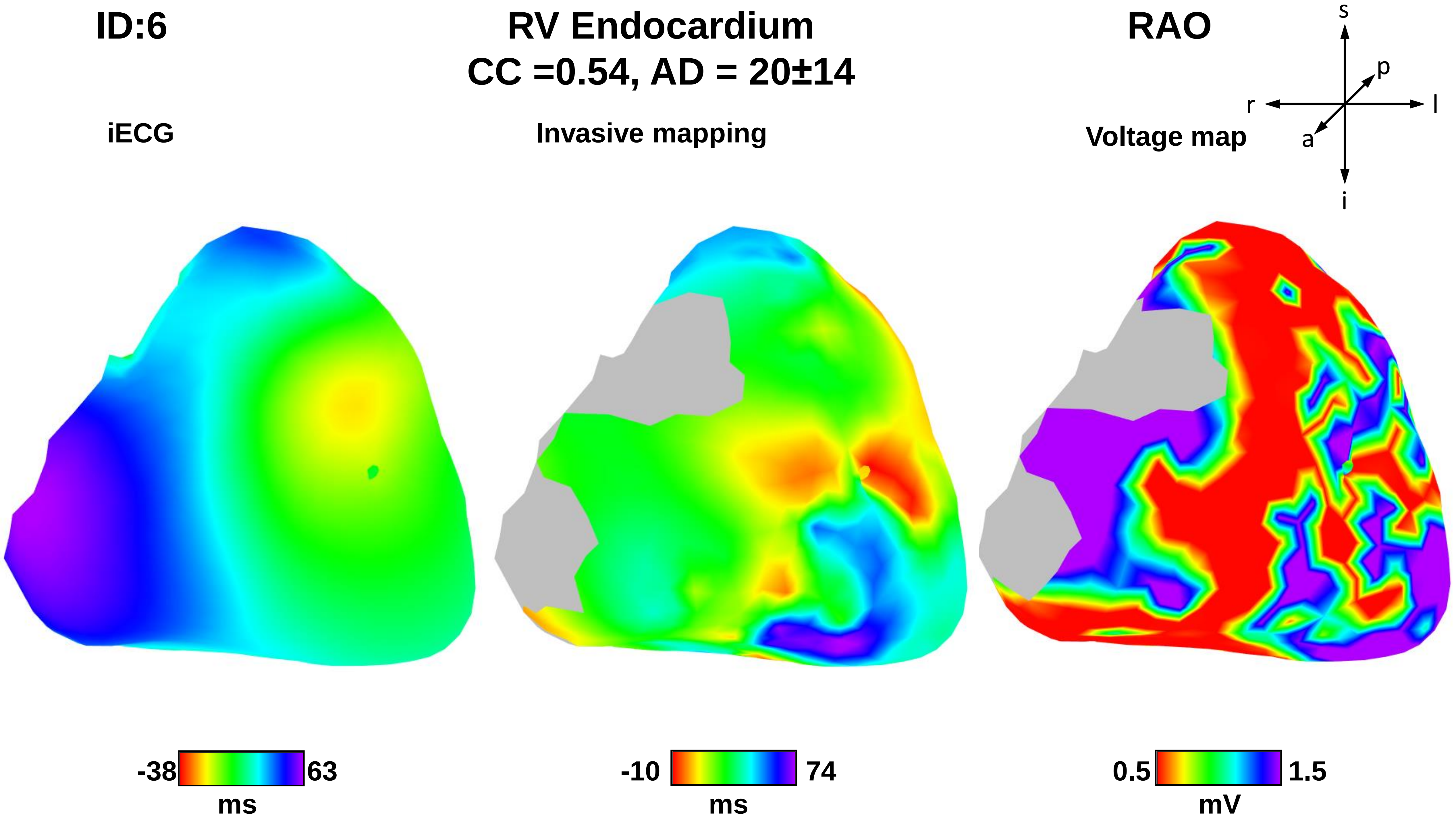
ID:6
RV Endocardium
CC =0.54, AD = 20±14
RAO
s
p
r
l
a
i
iECG
Invasive mapping
Voltage map
-38
63
ms
-10
74
ms
0.5
1.5
mV

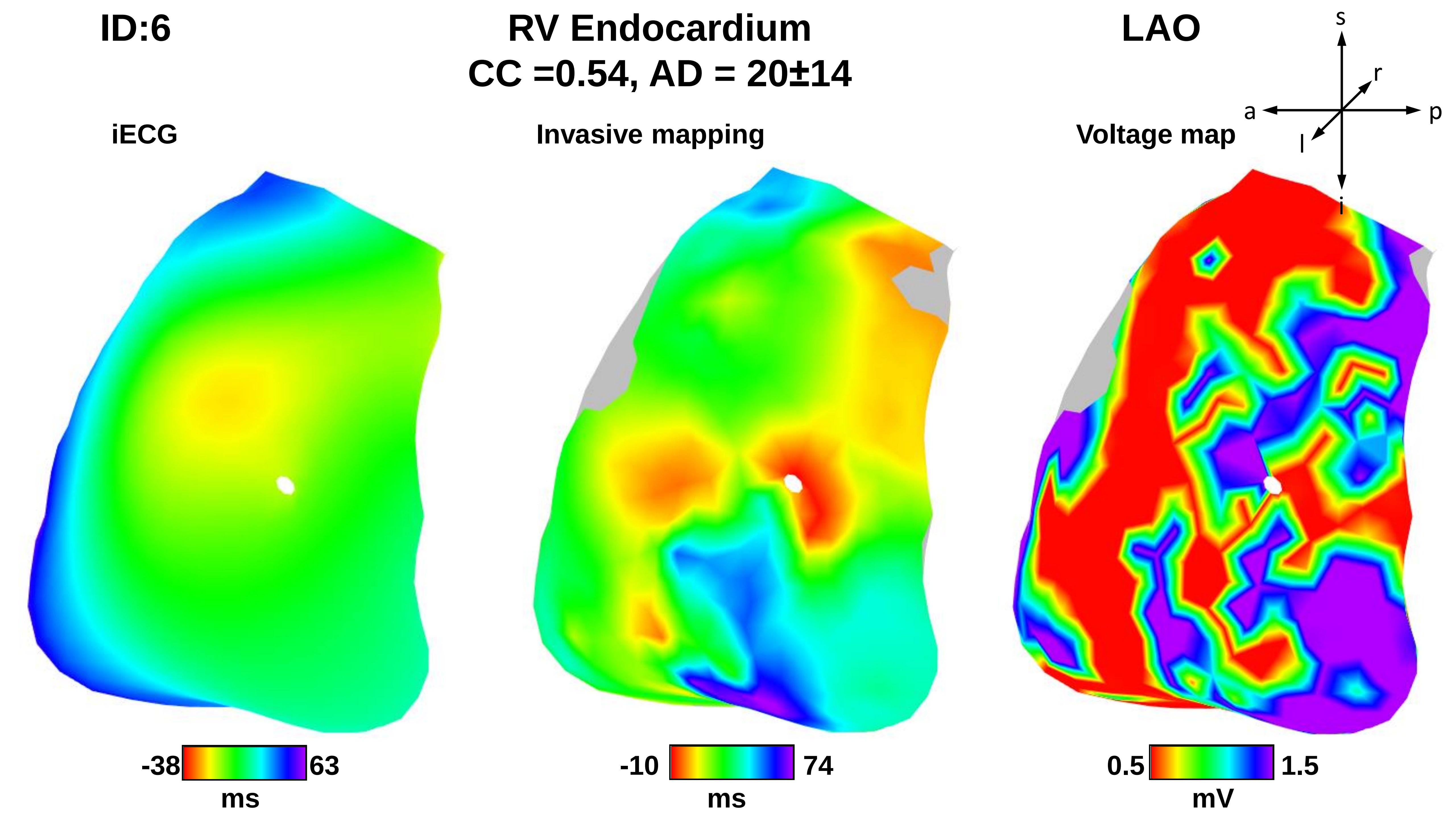

ID:6
RV Endocardium
CC =0.54, AD = 20±14
LAO
iECG
Invasive mapping
Voltage map
s
r
a
p
l
i
-38
63
ms
-10
74
ms
0.5
1.5
mV

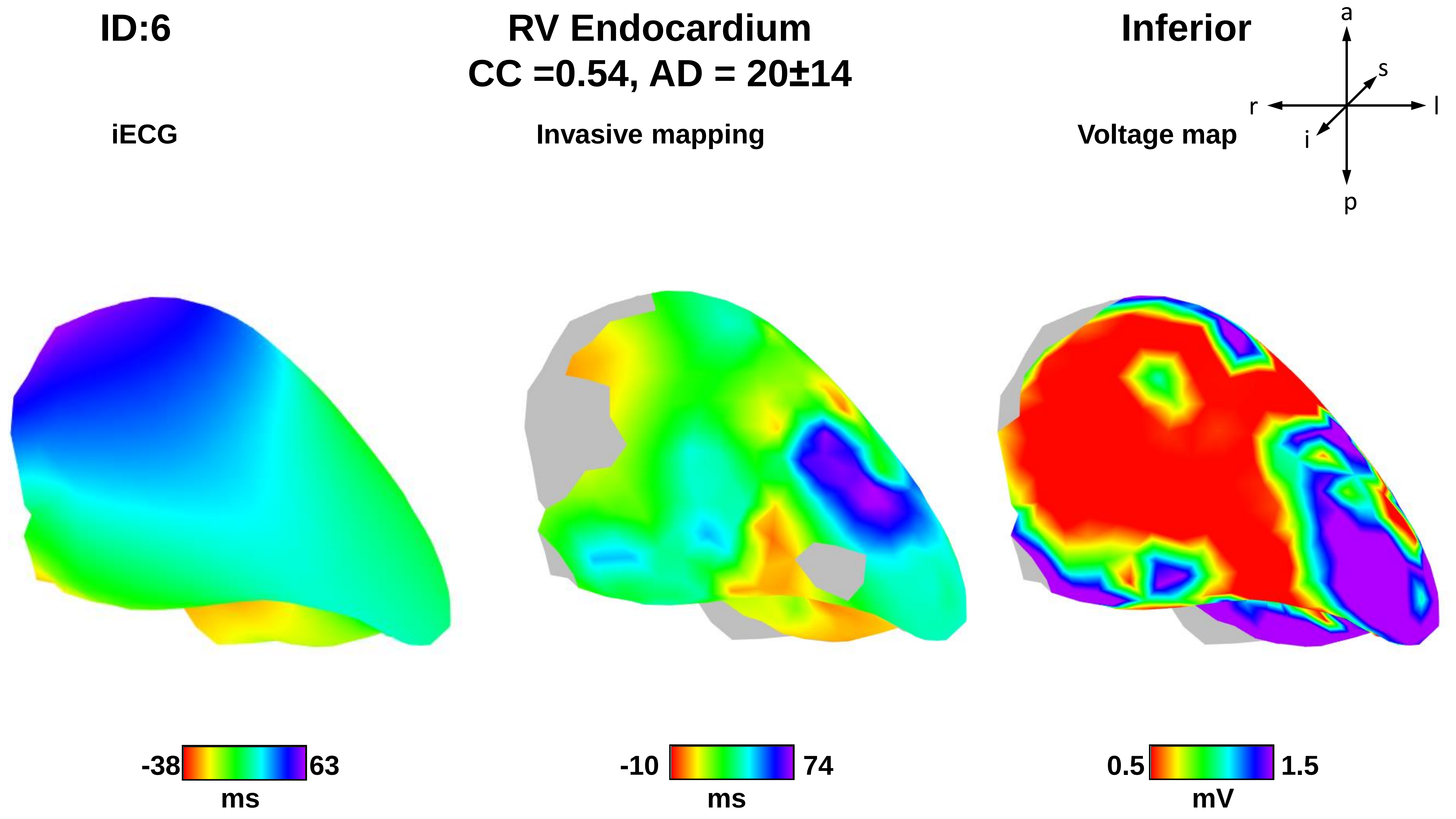
ID:6
RV Endocardium
CC =0.54, AD = 20±14
Inferior
a
s
r
l
i
p
iECG
Invasive mapping
Voltage map
-38
63
ms
-10
74
ms
0.5
1.5
mV

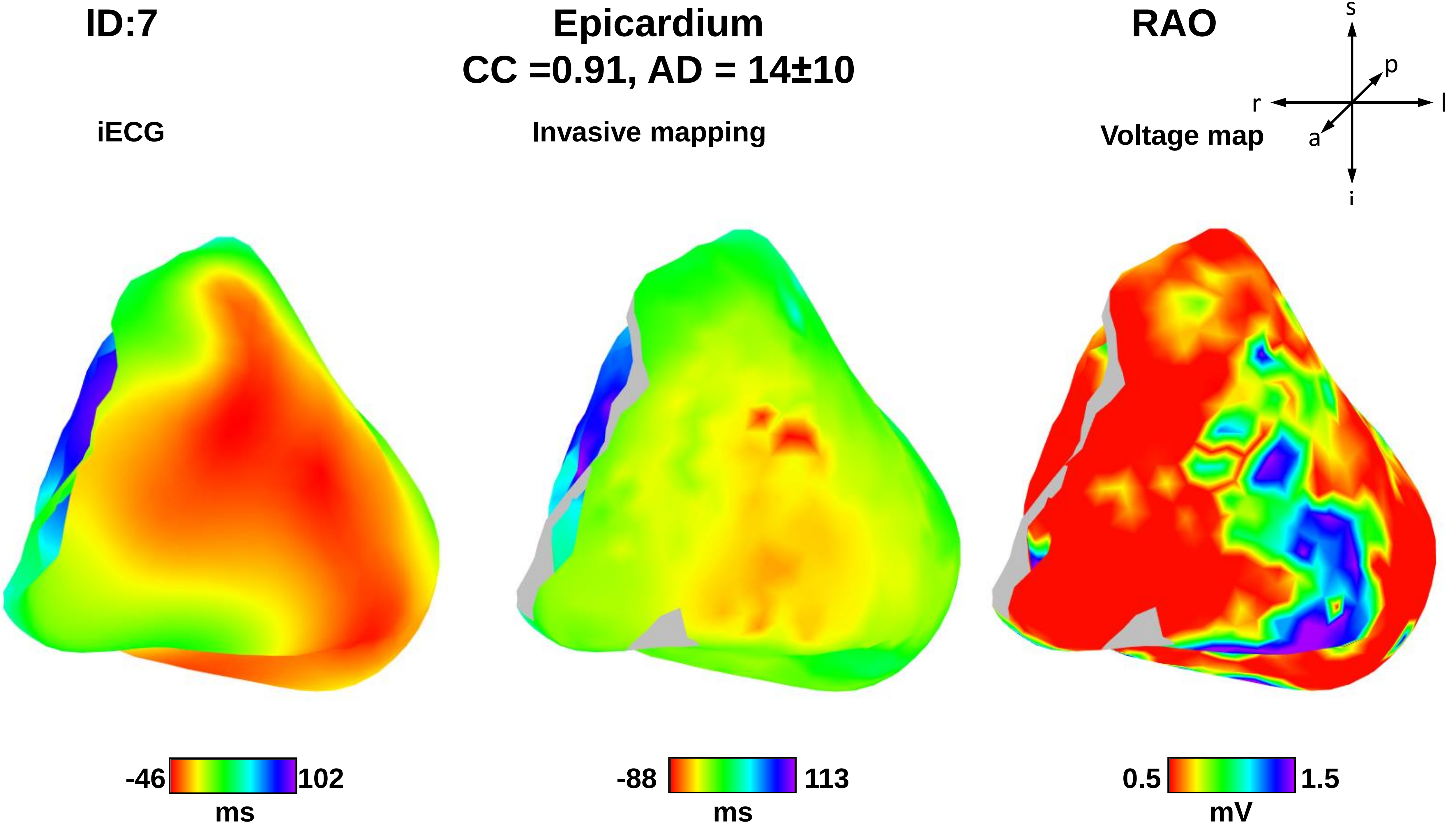
ID:7
Epicardium
CC =0.91, AD = 14±10
RAO
iECG
Invasive mapping
Voltage map
s
p
r
l
a
i
-46
102
ms
-88
113
ms
0.5
1.5
mV

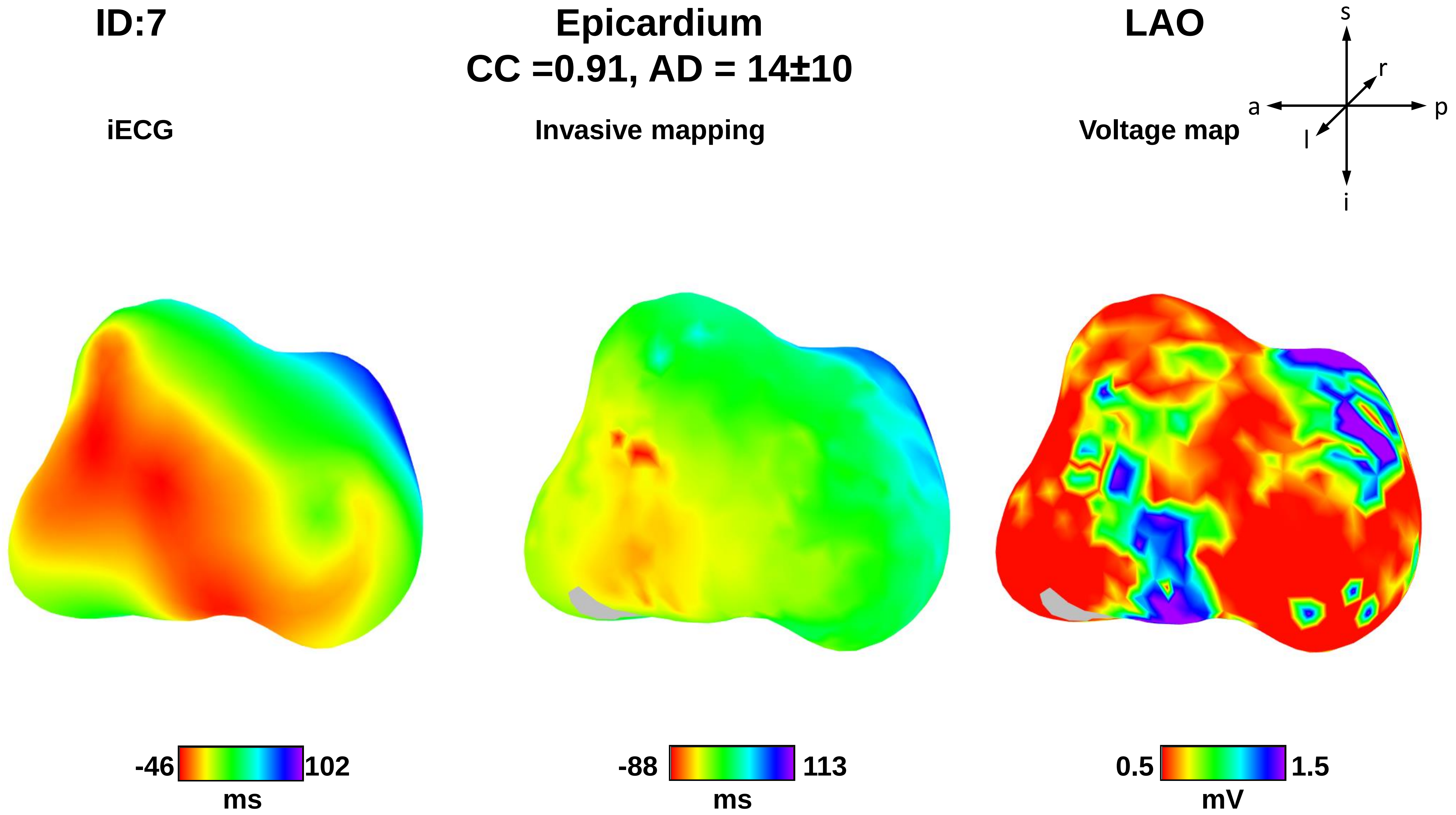
ID:7
Epicardium
CC =0.91, AD = 14±10
LAO
s
r
a
p
l
i
iECG
Invasive mapping
Voltage map
-46
102
ms
-88
113
ms
0.5
1.5
mV

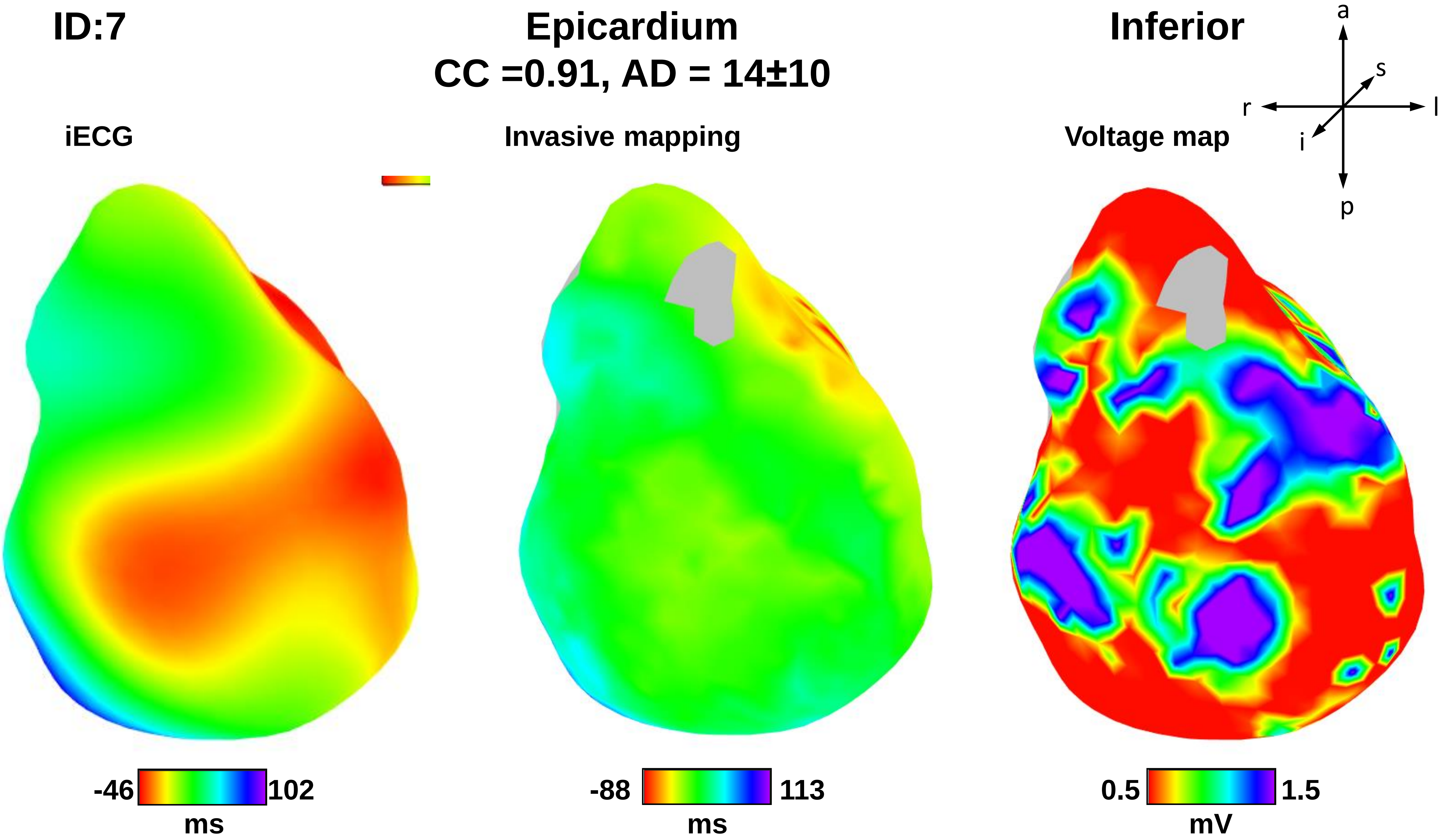
ID:7
Epicardium
CC =0.91, AD = 14±10
Inferior
a
s
r
l
i
p
iECG
Invasive mapping
Voltage map
-46
102
ms
-88
113
ms
0.5
1.5
mV

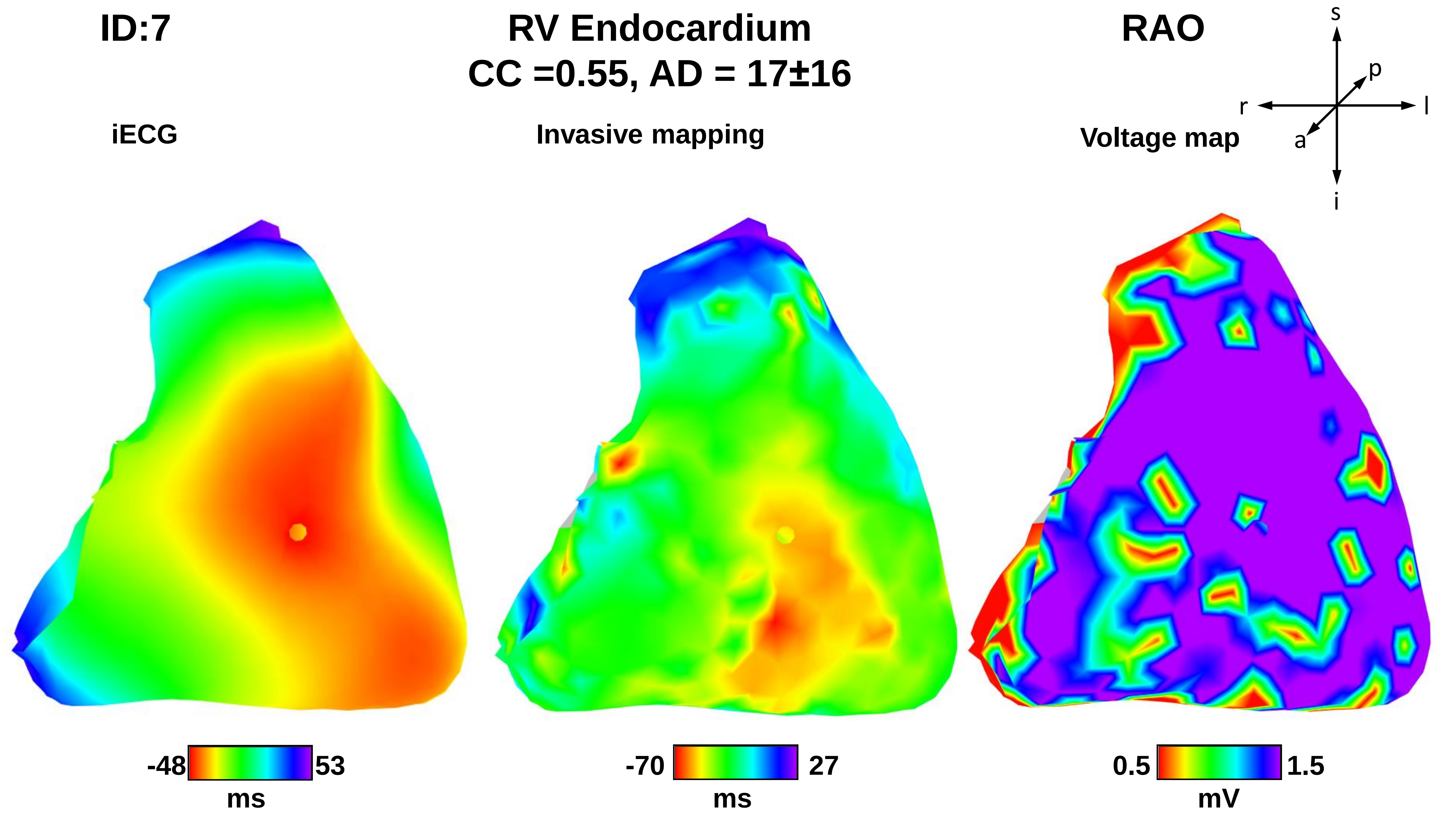
ID:7
RV Endocardium
CC =0.55, AD = 17±16
RAO
s
p
r
l
a
i
iECG
Invasive mapping
Voltage map
-48
53
ms
-70
27
ms
0.5
1.5
mV

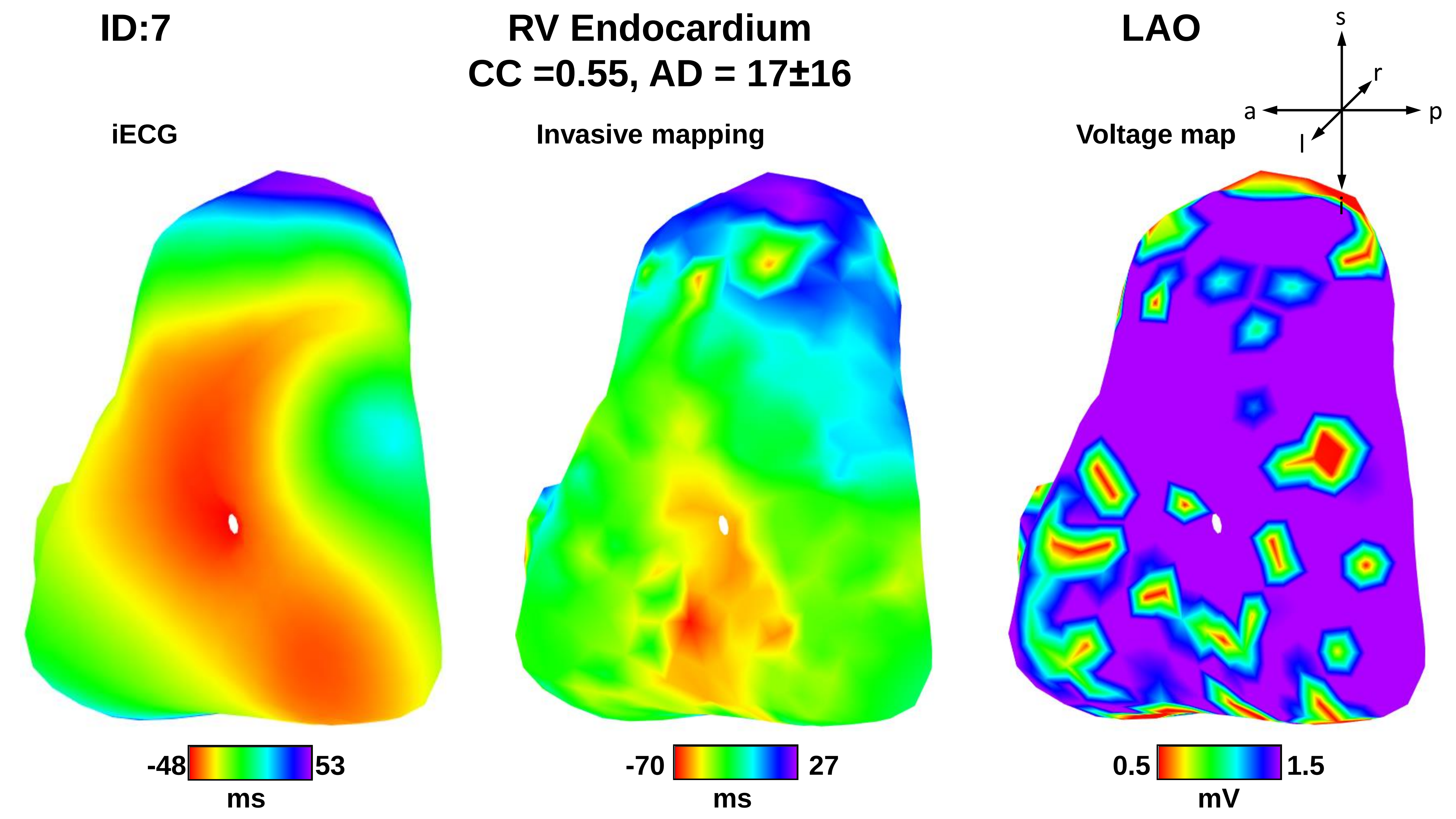
ID:7
RV Endocardium
CC =0.55, AD = 17±16
LAO
s
r
a
p
l
i
iECG
Invasive mapping
Voltage map
-48
53
ms
-70
27
ms
0.5
1.5
mV

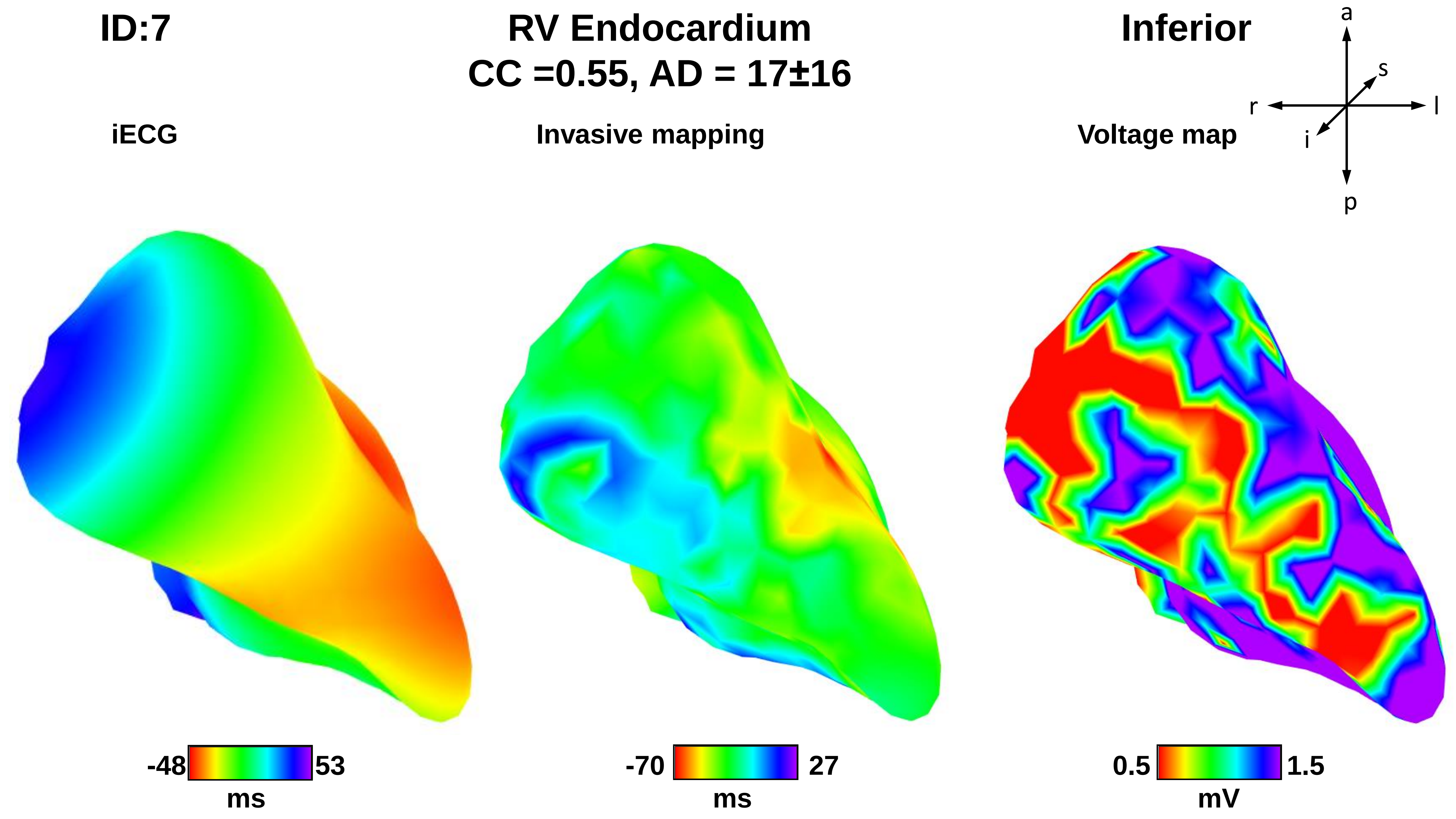
ID:7
RV Endocardium
CC =0.55, AD = 17±16
Inferior
a
s
r
l
i
p
iECG
Invasive mapping
Voltage map
-48
53
ms
-70
27
ms
0.5
1.5
mV

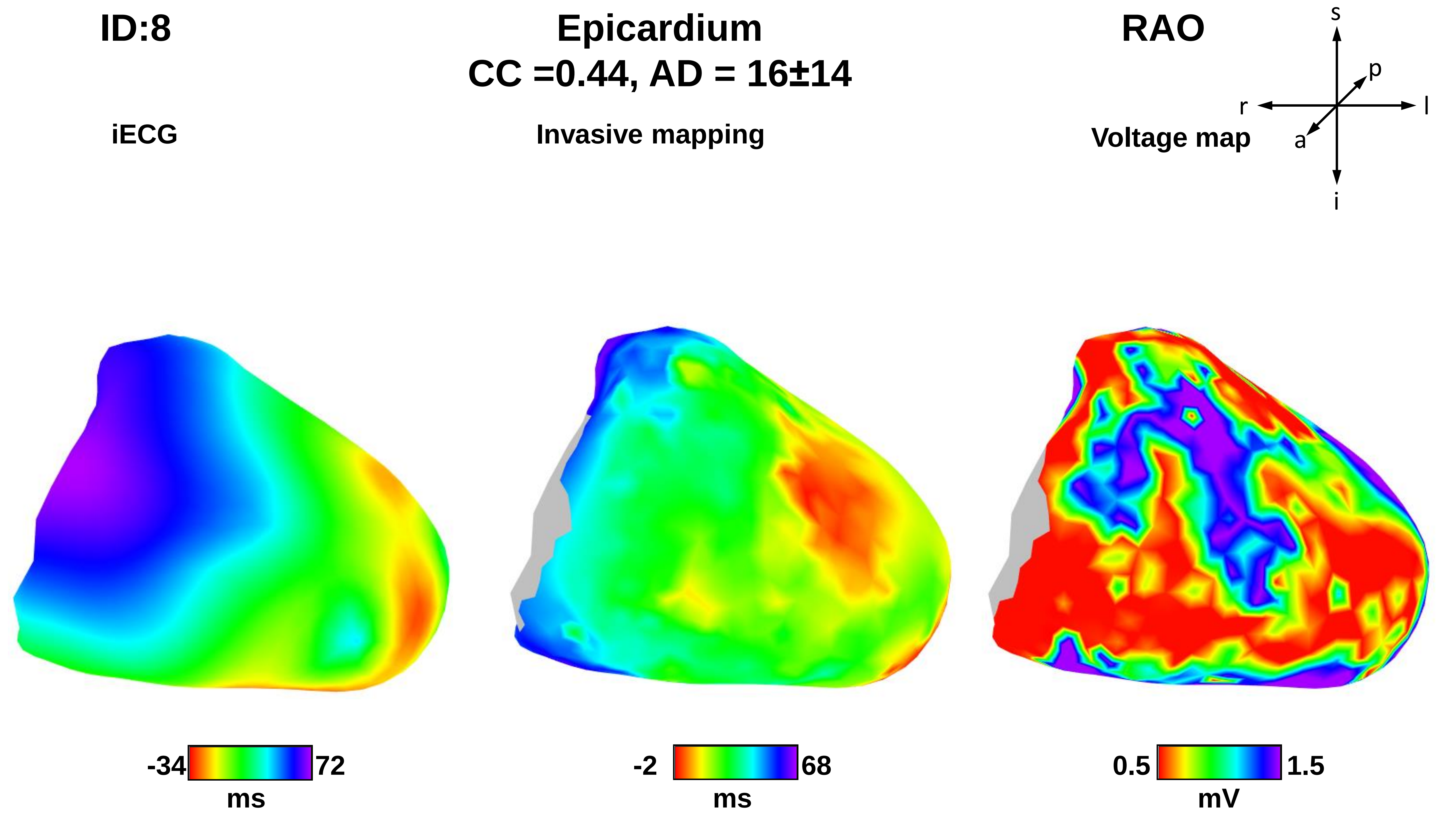
ID:8
Epicardium
CC =0.44, AD = 16±14
RAO
iECG
Invasive mapping
Voltage map
s
p
r
l
a
i
-34
72
ms
-2
68
ms
0.5
1.5
mV

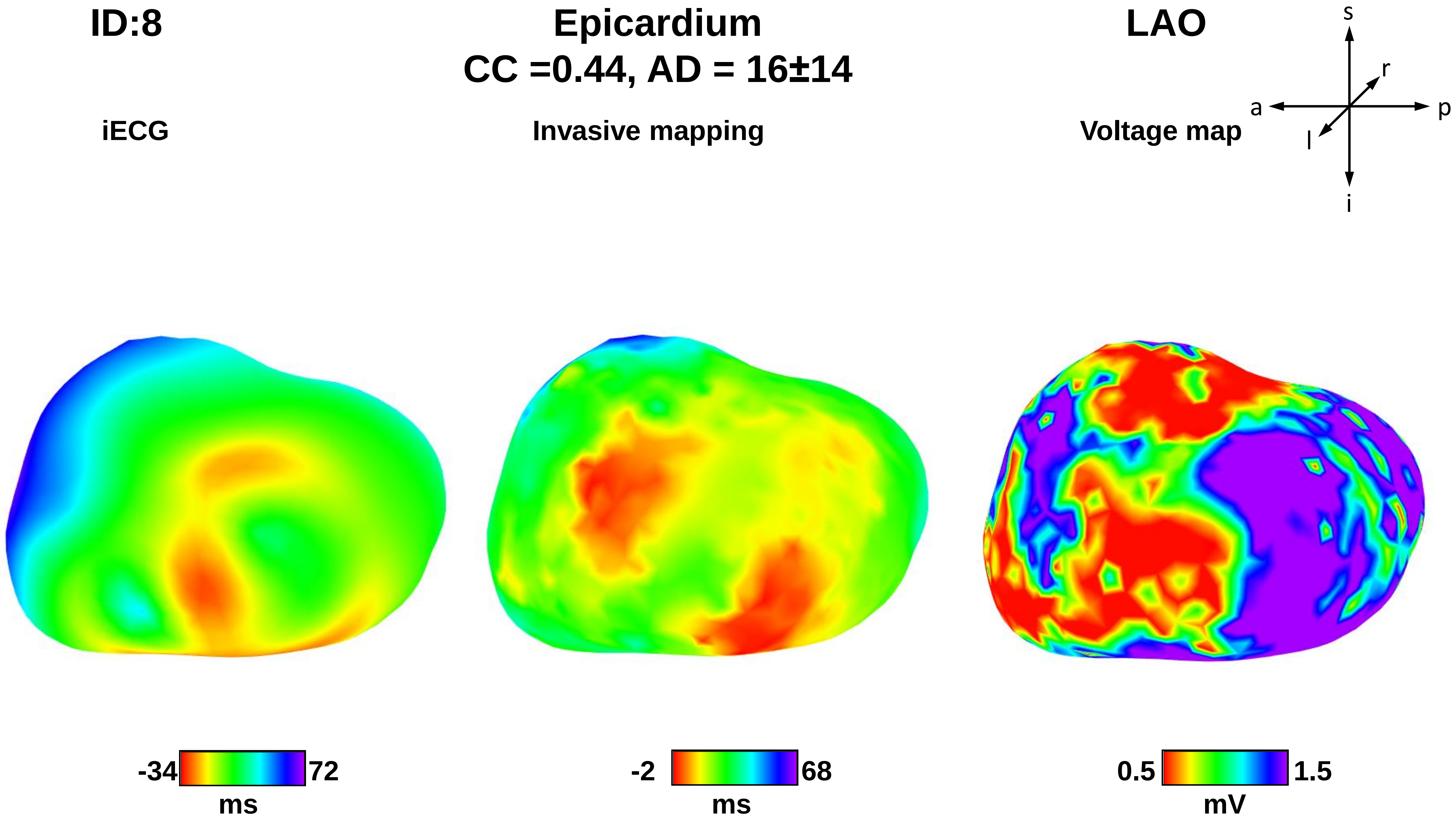

ID:8
Epicardium
CC =0.44, AD = 16±14
LAO
iECG
Invasive mapping
Voltage map
s
r
a
p
l
i
-34
72
ms
-2
68
ms
0.5
1.5
mV

ID:8

Epicardium
CC =0.44, AD = 16±14

Inferior

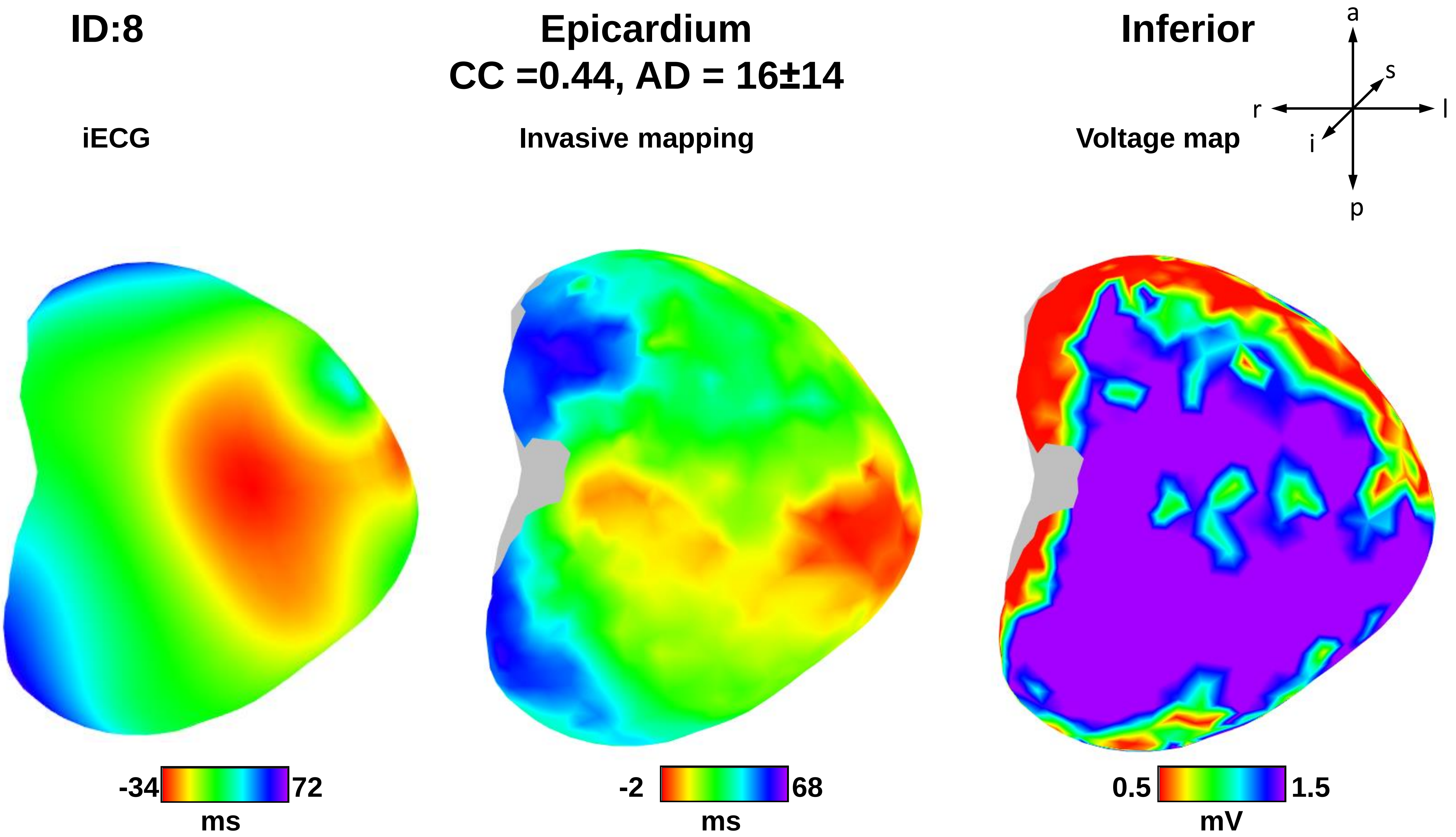

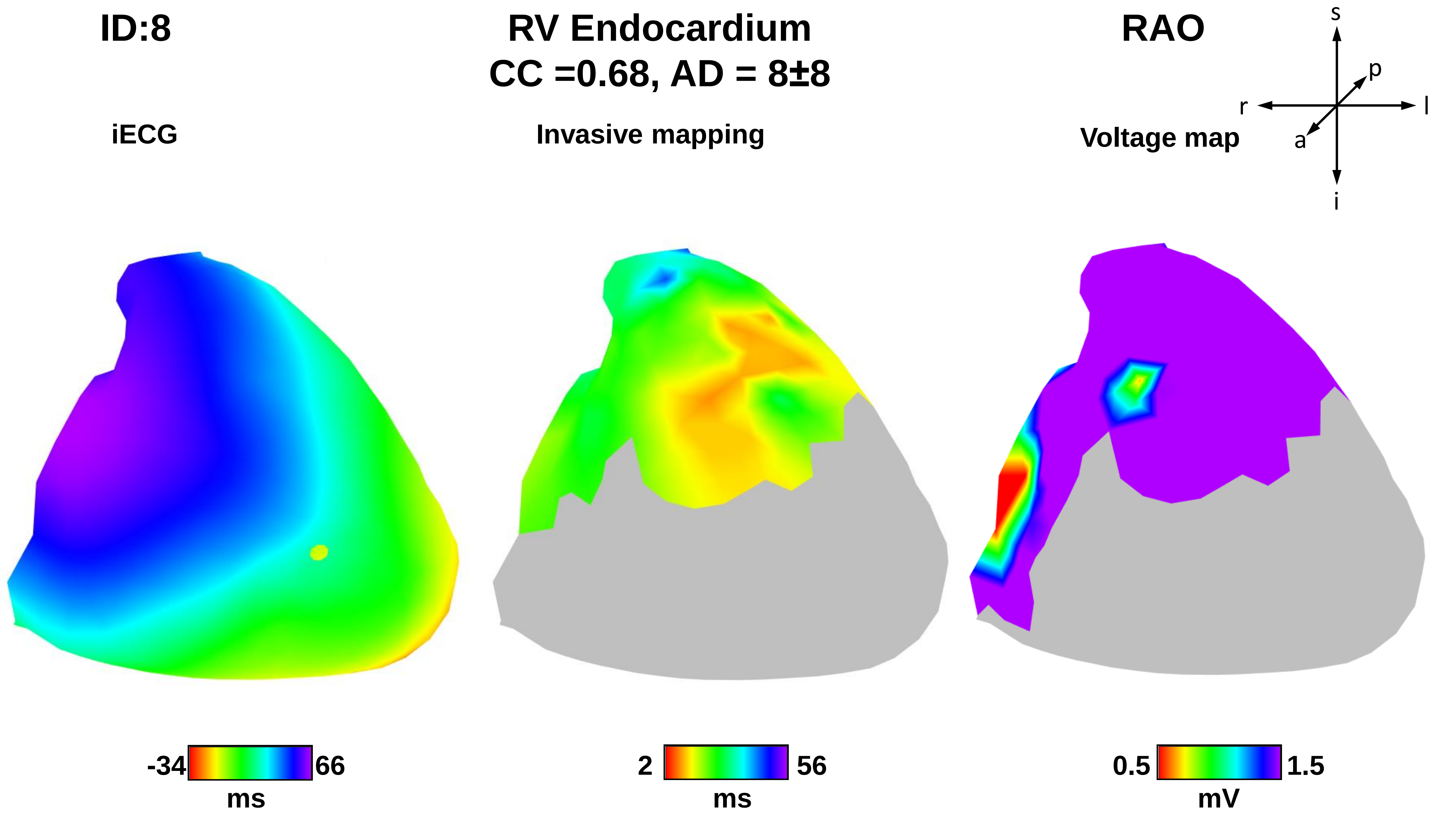
ID:8
RV Endocardium
CC =0.68, AD = 8±8
RAO
s
p
r
l
a
i
iECG
Invasive mapping
Voltage map
-34
66
ms
2
56
ms
0.5
1.5
mV

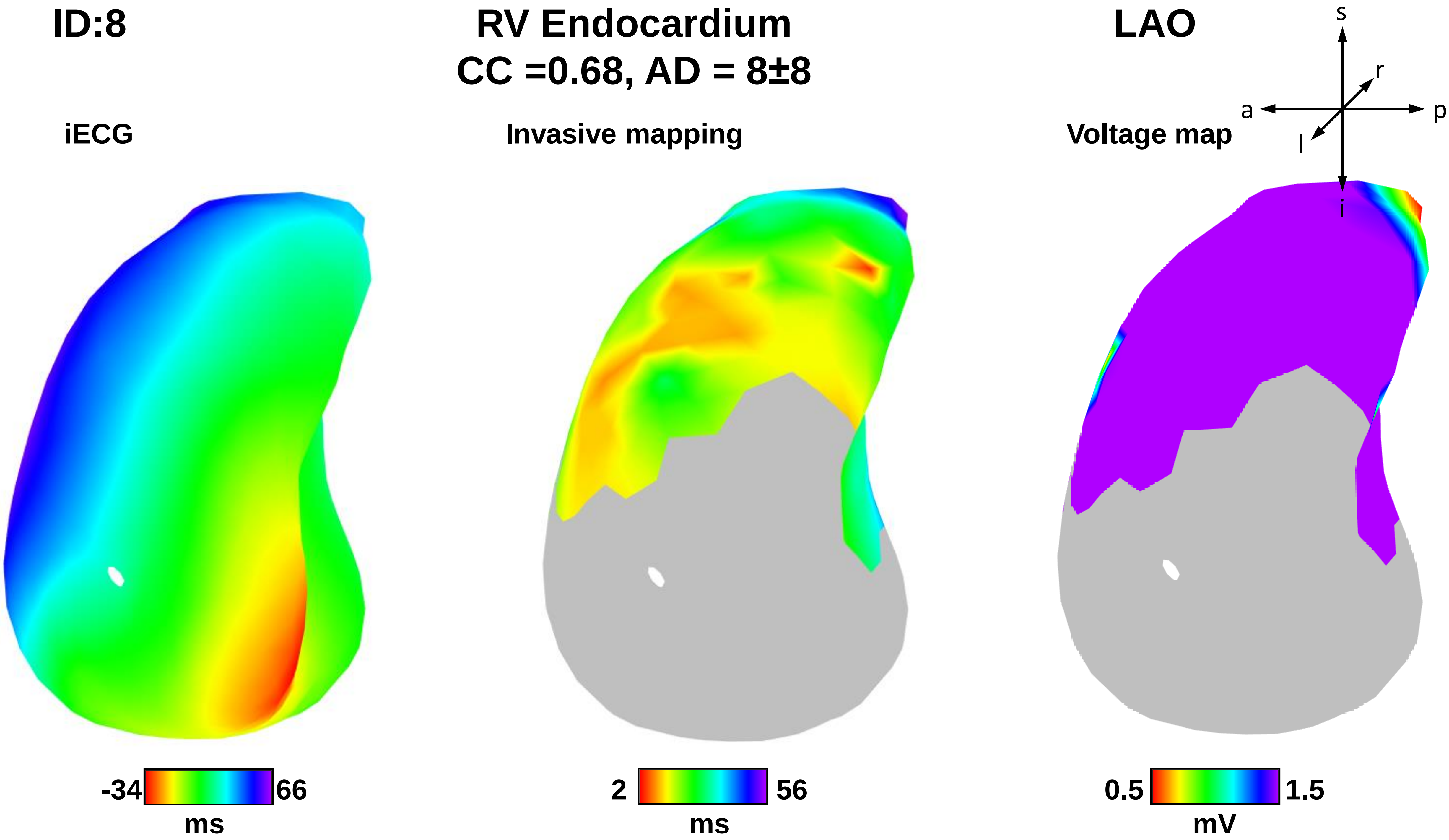
ID:8
RV Endocardium
CC =0.68, AD = 8±8
LAO
s
r
a
p
l
i
iECG
Invasive mapping
Voltage map
-34
66
ms
2
56
ms
0.5
1.5
mV

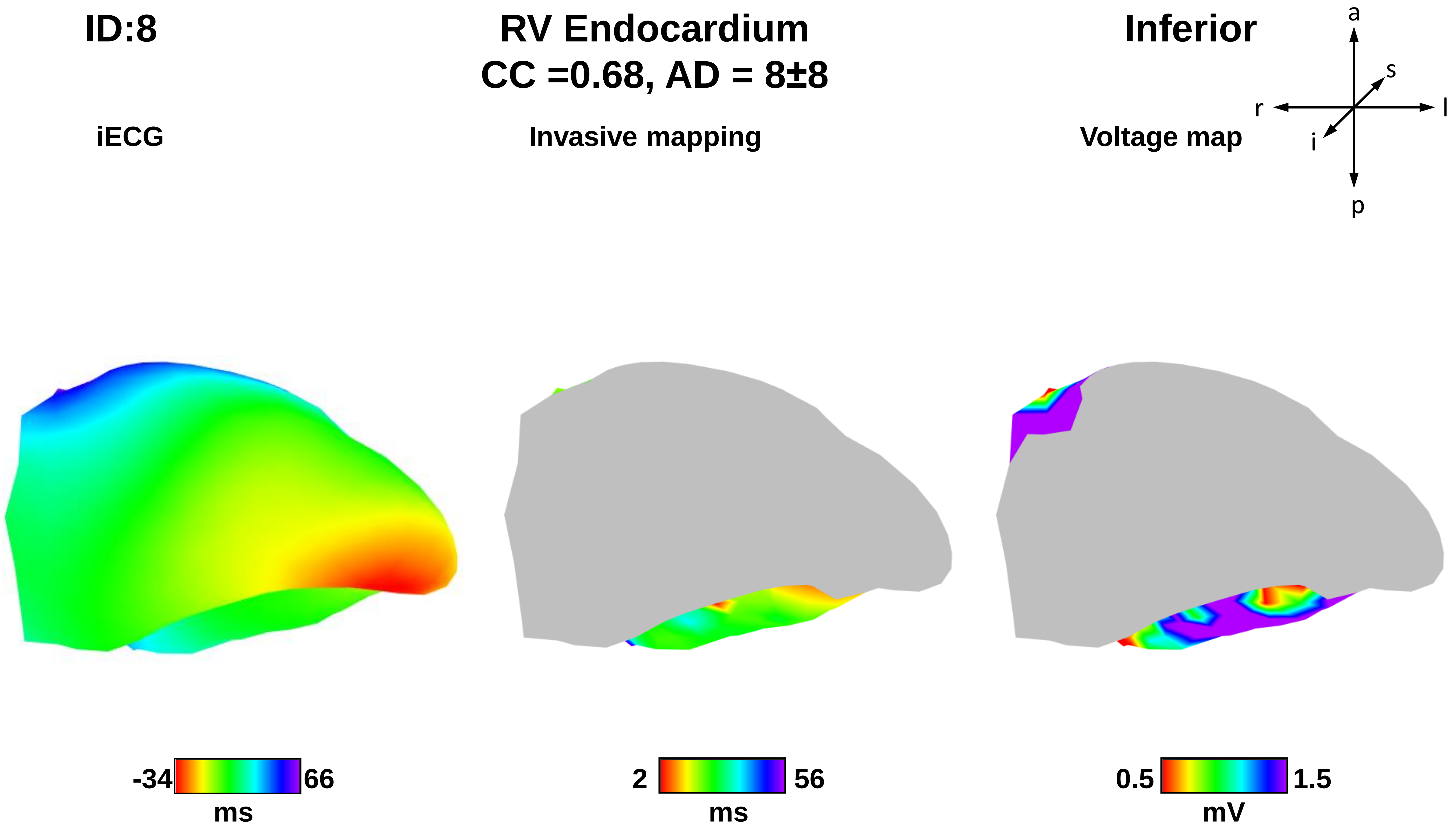
ID:8
RV Endocardium
CC =0.68, AD = 8±8
Inferior
a
s
r
l
i
p
iECG
Invasive mapping
Voltage map
-34
66
ms
2
56
ms
0.5
1.5
mV

ID:8

LV Endocardium
CC =0.40 AD = 30±20

RAO

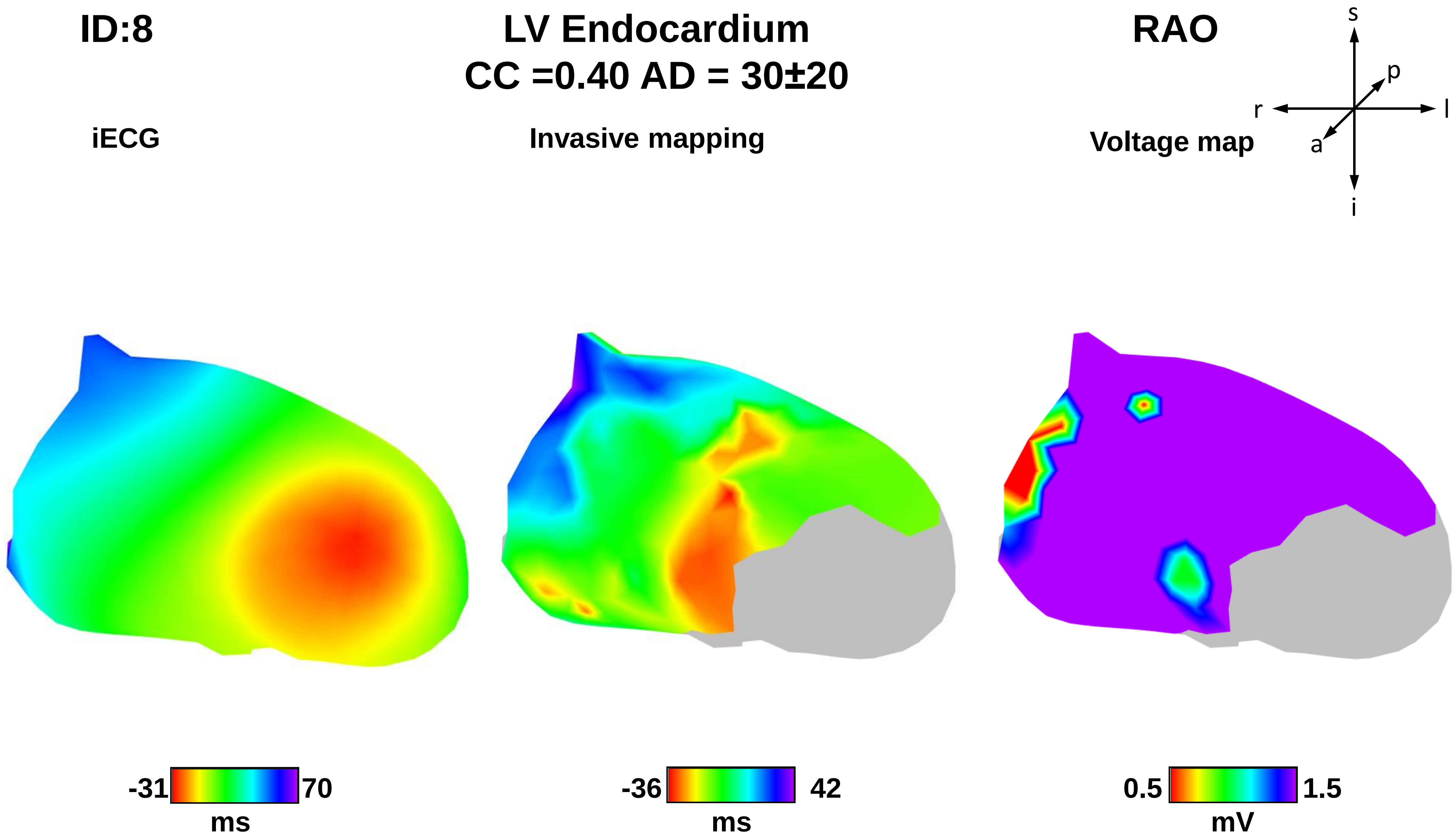

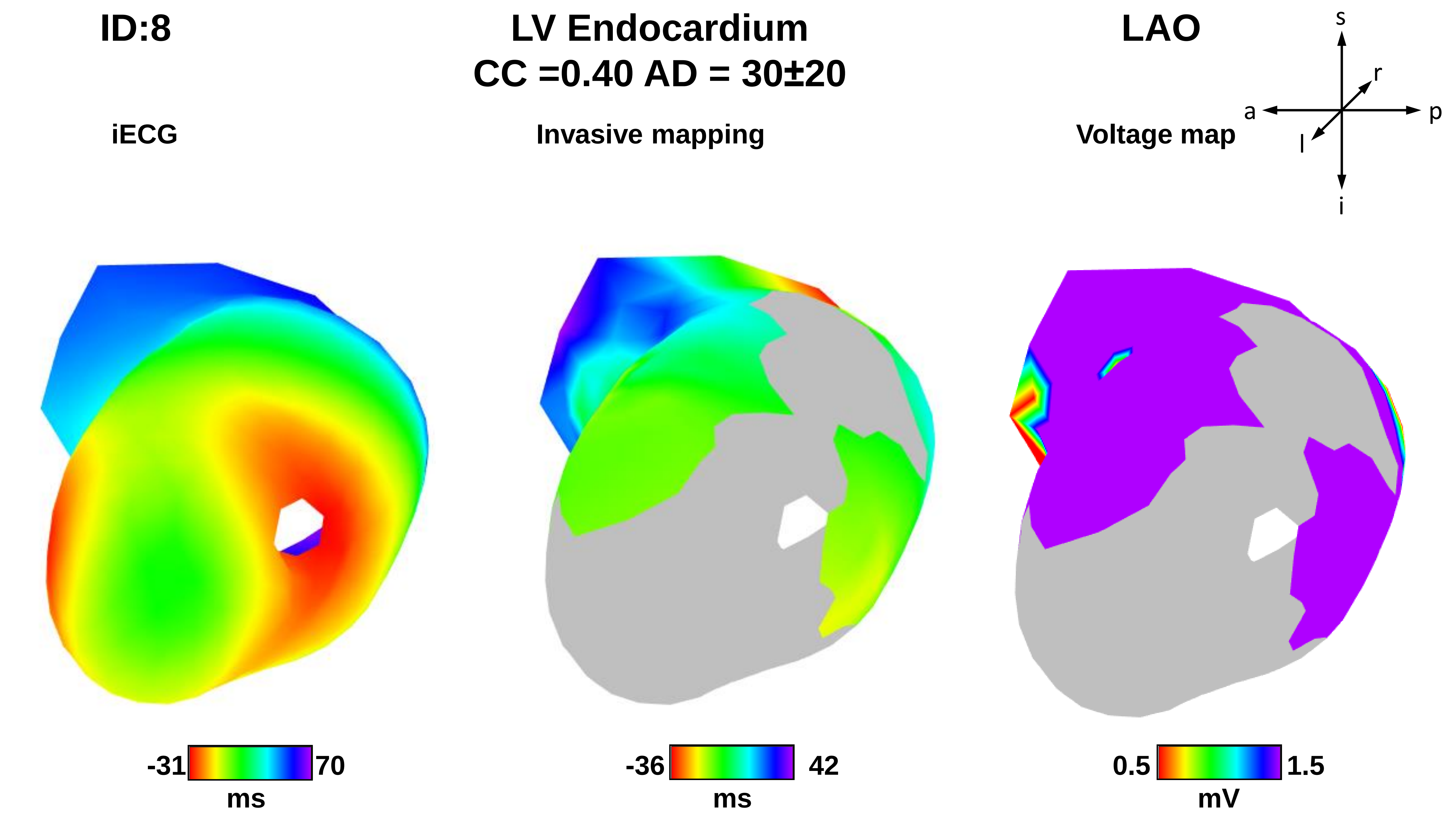
ID:8
LV Endocardium
CC =0.40 AD = 30±20
LAO
iECG
Invasive mapping
Voltage map
s
r
a
p
l
i
-31
70
ms
-36
42
ms
0.5
1.5
mV

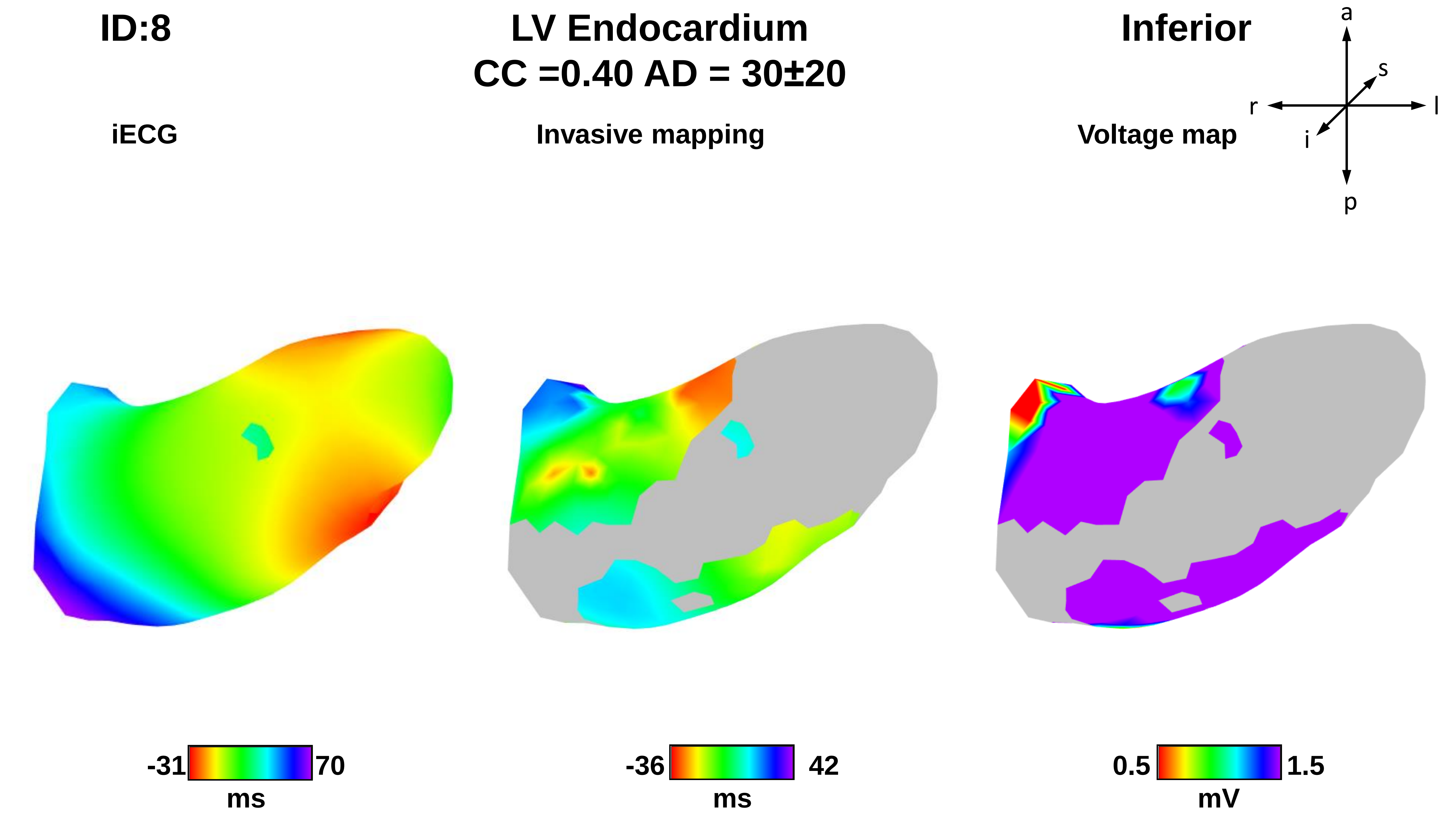

ID:8
LV Endocardium
CC =0.40 AD = 30±20
Inferior
iECG
Invasive mapping
Voltage map
a
s
r
l
i
p
-31
70
ms
-36
42
ms
0.5
1.5
mV

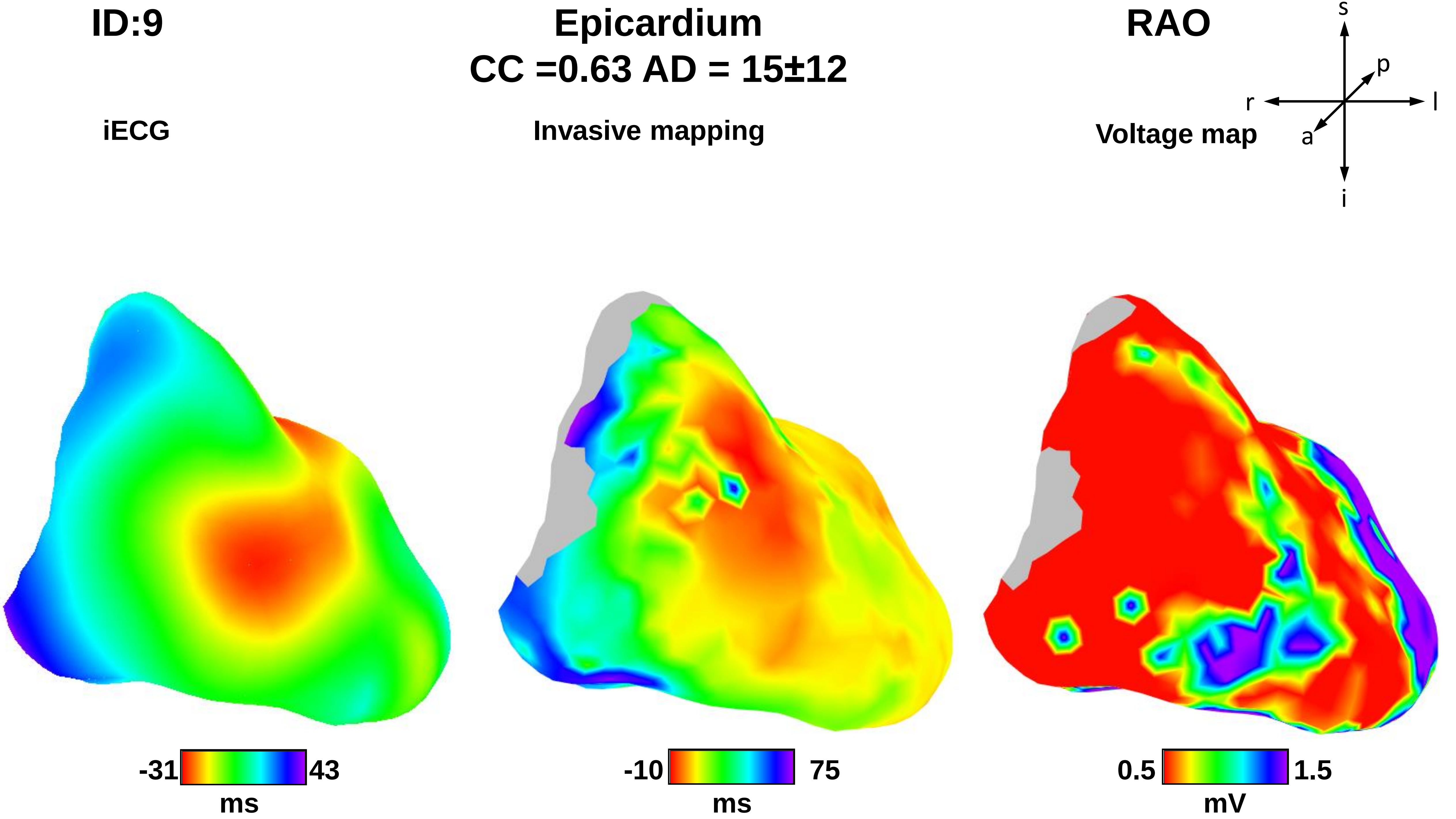
ID:9
Epicardium
CC =0.63 AD = 15±12
RAO
s
p
r
l
a
i
iECG
Invasive mapping
Voltage map
-31
43
ms
-10
75
ms
0.5
1.5
mV

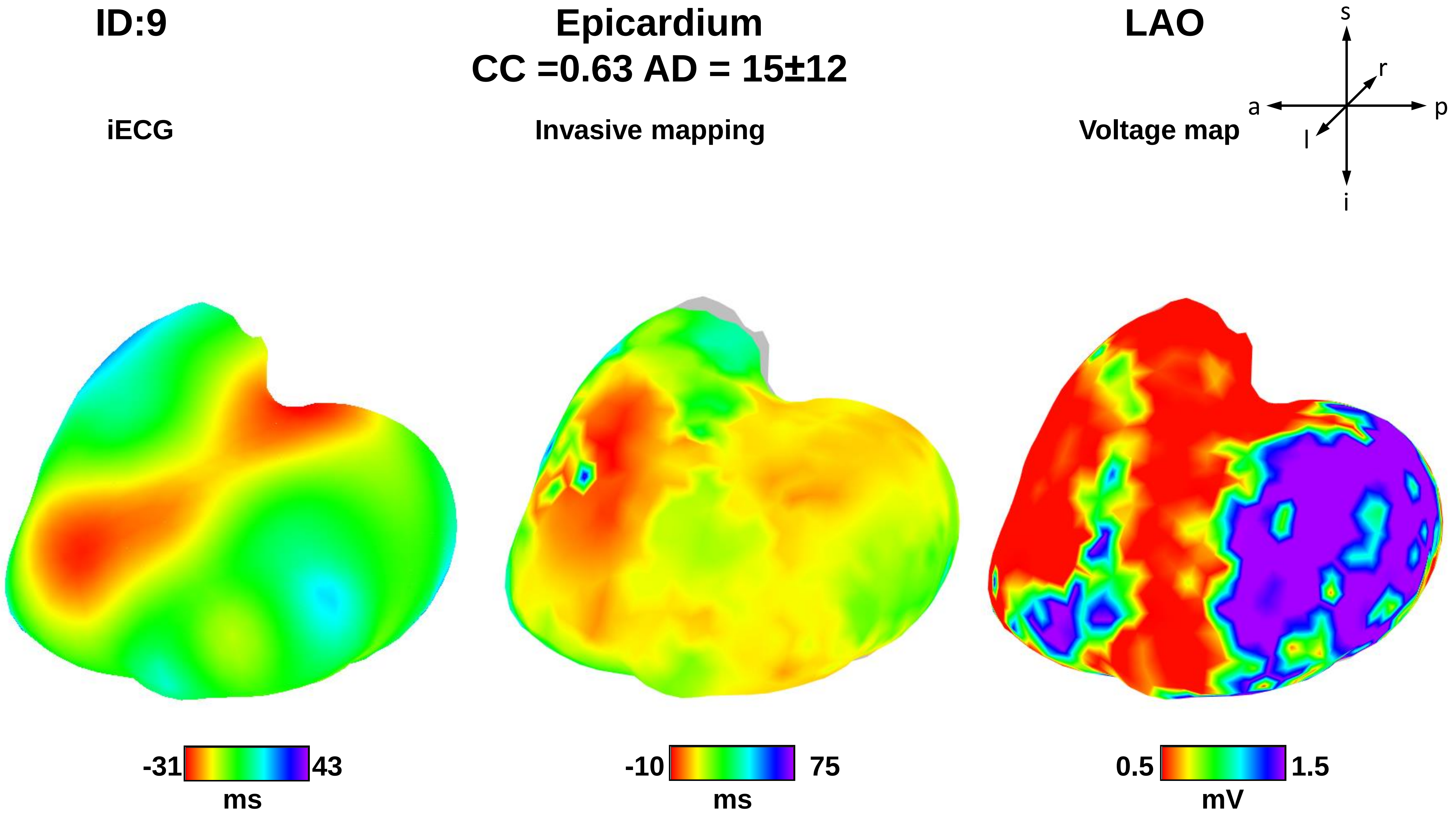

ID:9
Epicardium
CC =0.63 AD = 15±12
LAO
s
r
a
p
l
i
iECG
Invasive mapping
Voltage map
-31
43
ms
-10
75
ms
0.5
1.5
mV

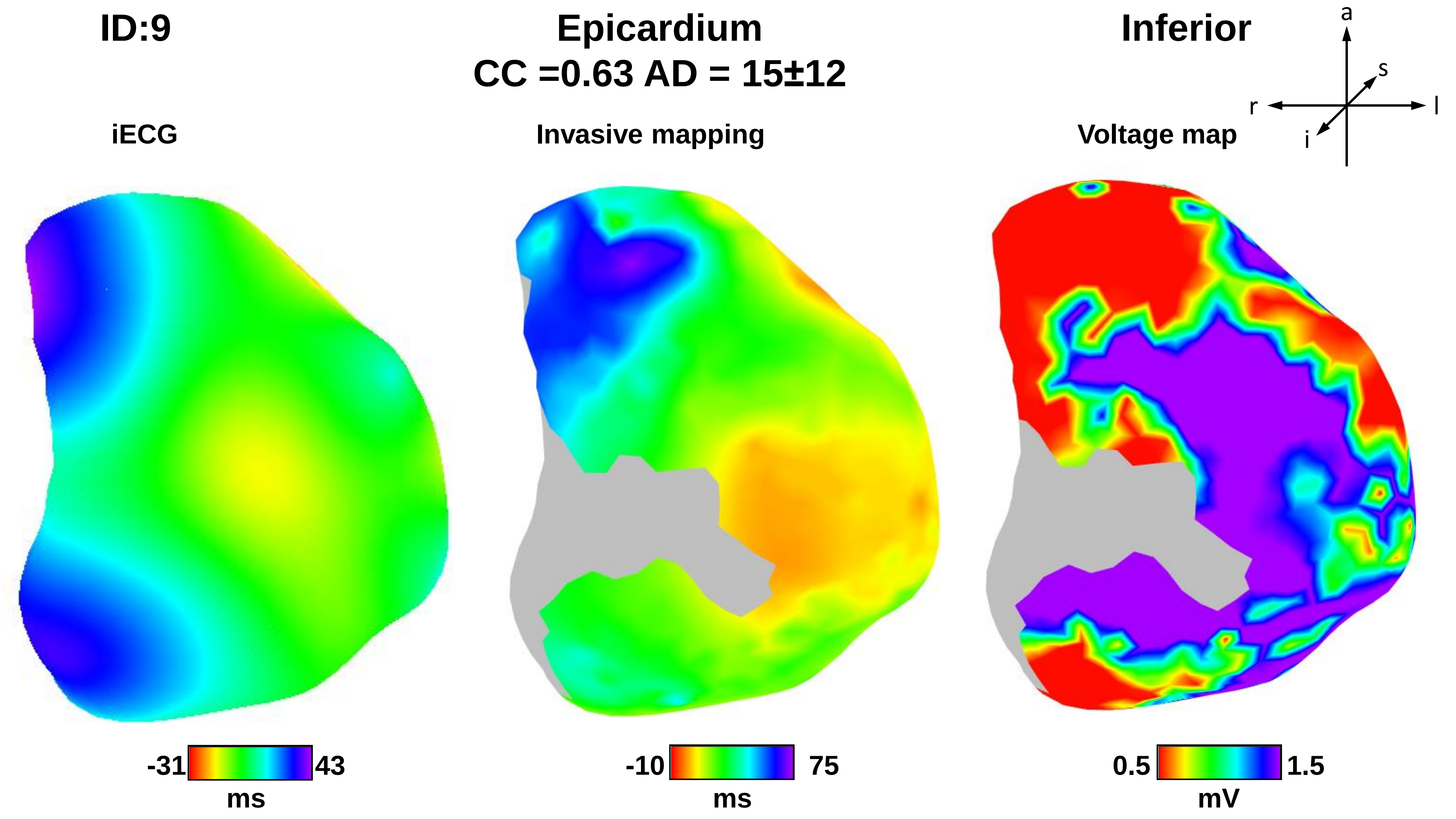
ID:9
Epicardium
CC =0.63 AD = 15±12
Inferior
a
s
r
l
i
iECG
Invasive mapping
Voltage map
-31
43
ms
-10
75
ms
0.5
1.5
mV

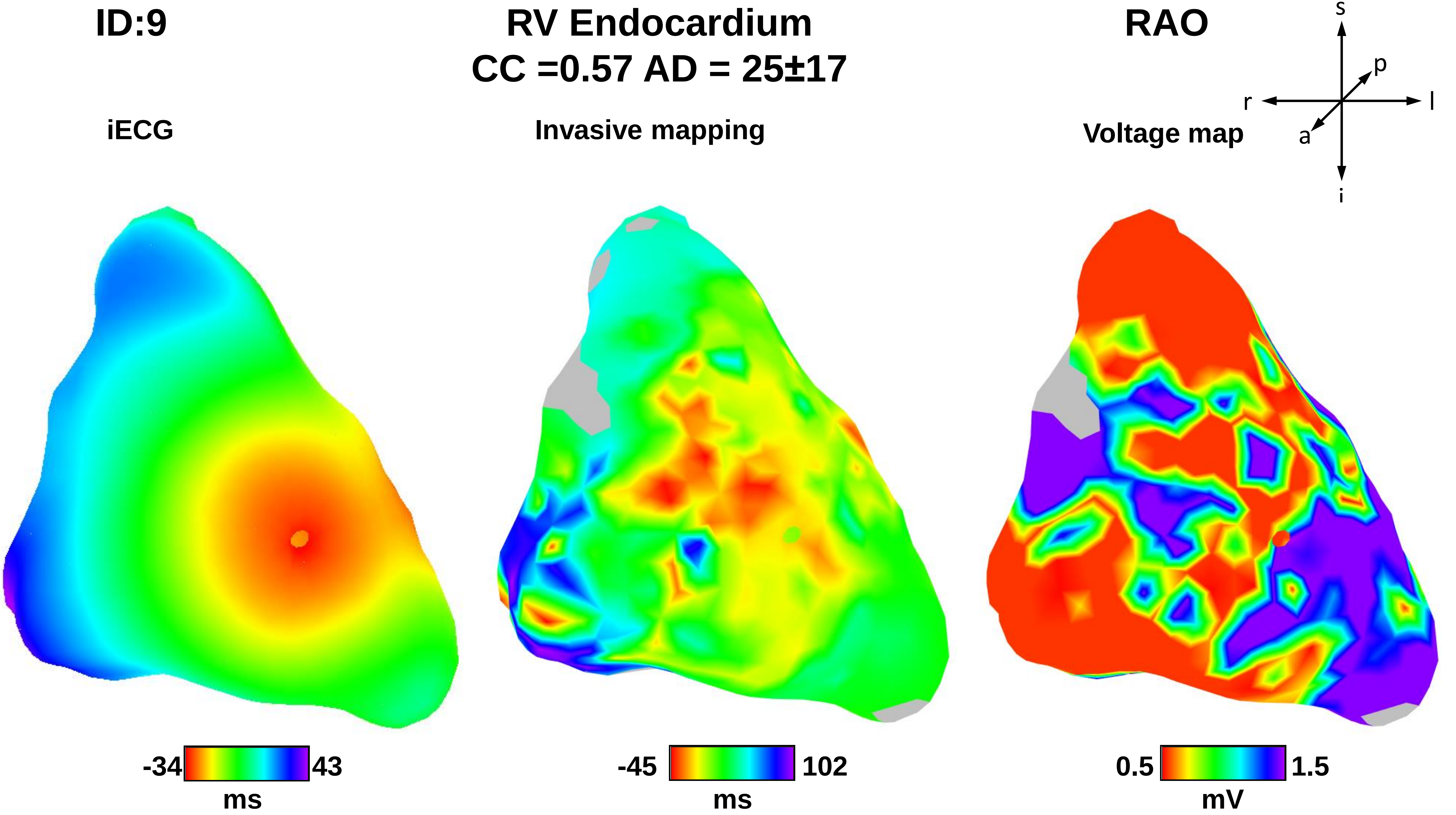

ID:9
RV Endocardium
CC =0.57 AD = 25±17
RAO
s
p
r
l
a
i
iECG
Invasive mapping
Voltage map
-34
43
ms
-45
102
ms
0.5
1.5
mV

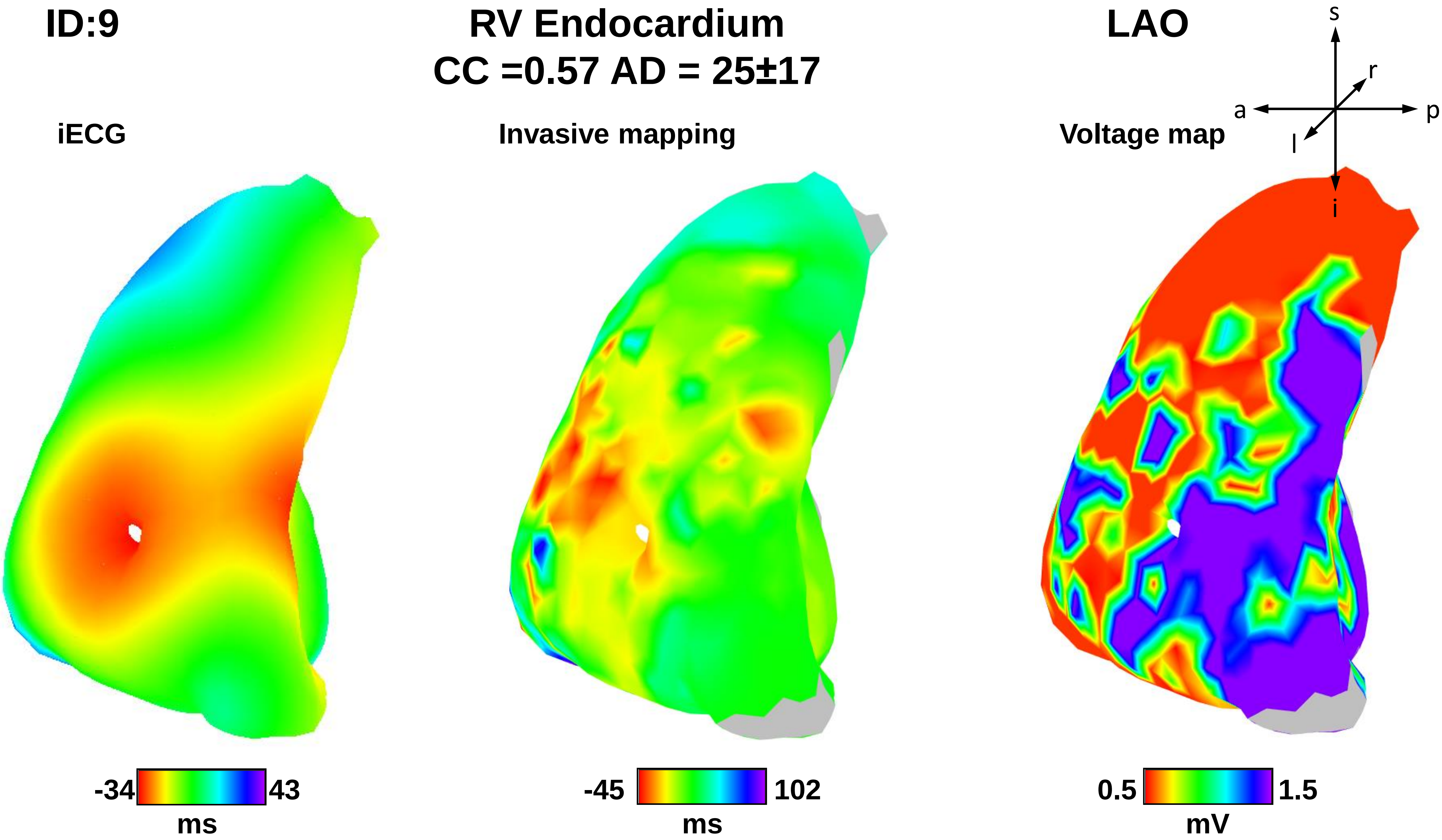
ID:9
RV Endocardium
CC =0.57 AD = 25±17
LAO
iECG
Invasive mapping
Voltage map
s
r
a
p
l
i
-34
43
ms
-45
102
ms
0.5
1.5
mV

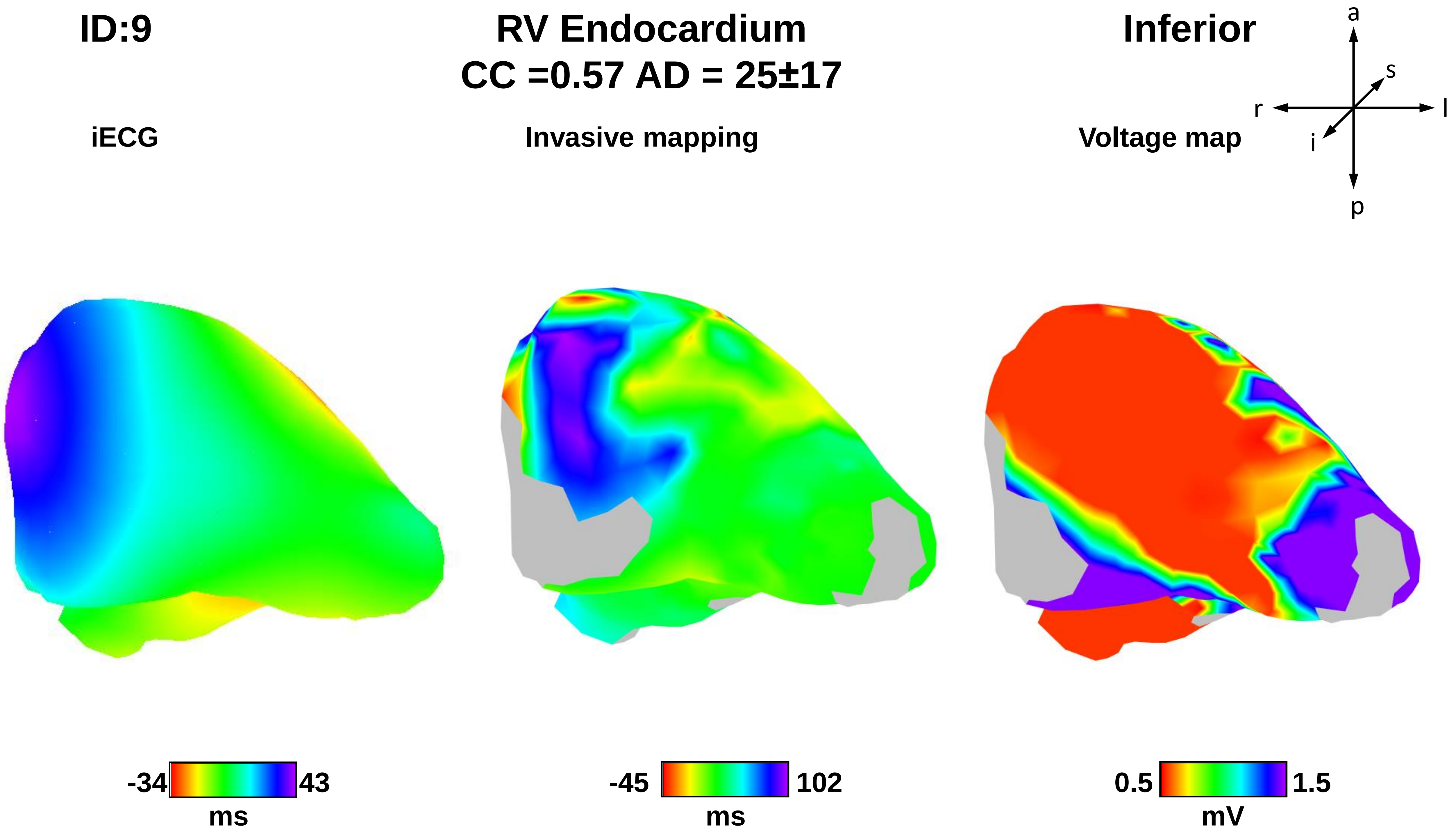
ID:9
RV Endocardium
CC =0.57 AD = 25±17
Inferior
iECG
Invasive mapping
Voltage map
a
s
r
l
i
p
-34
43
ms
-45
102
ms
0.5
1.5
mV

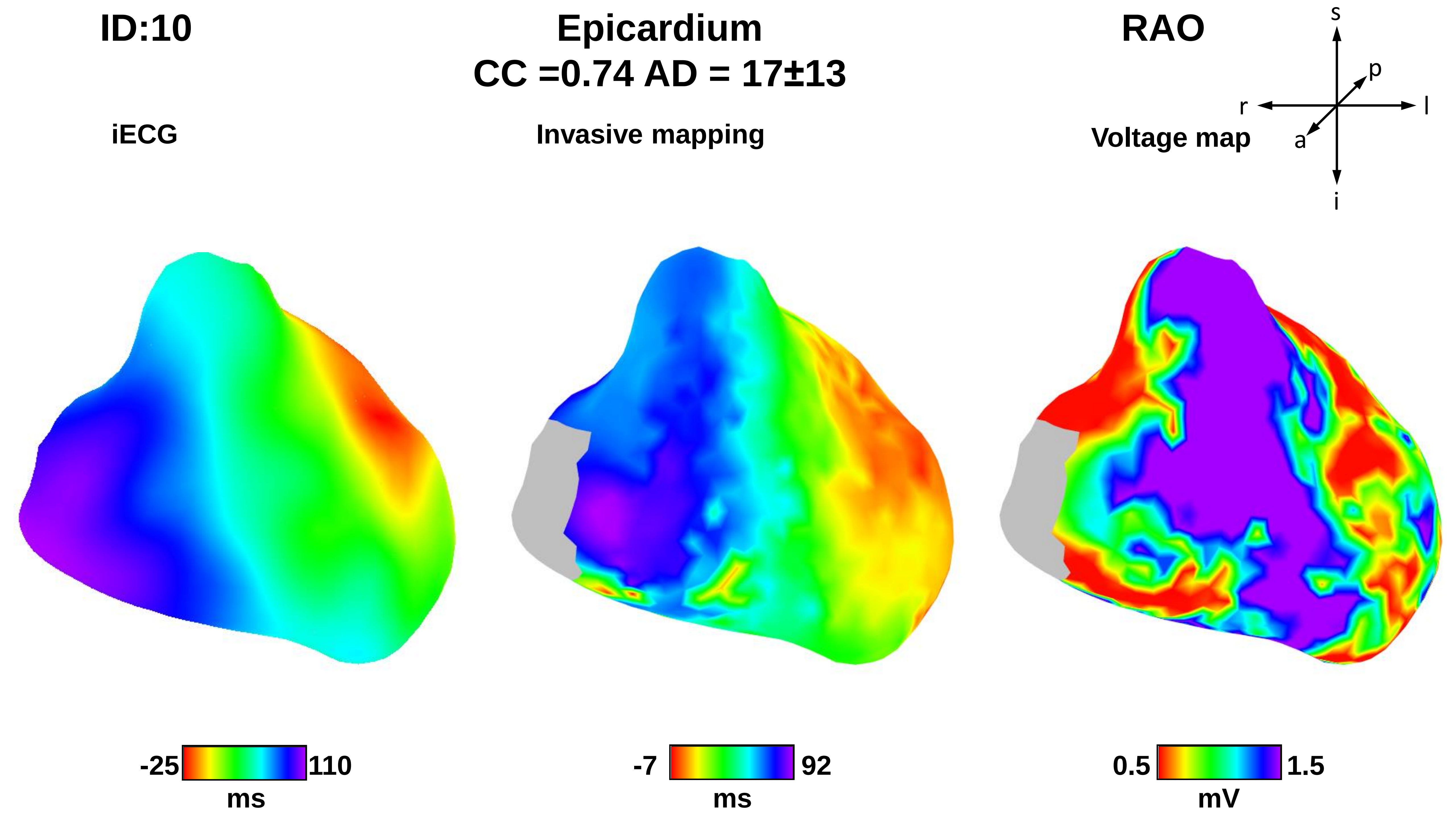
ID:10
Epicardium
CC =0.74 AD = 17±13
RAO
s
p
r
l
a
i
iECG
Invasive mapping
Voltage map
-25
110
ms
-7
92
ms
0.5
1.5
mV

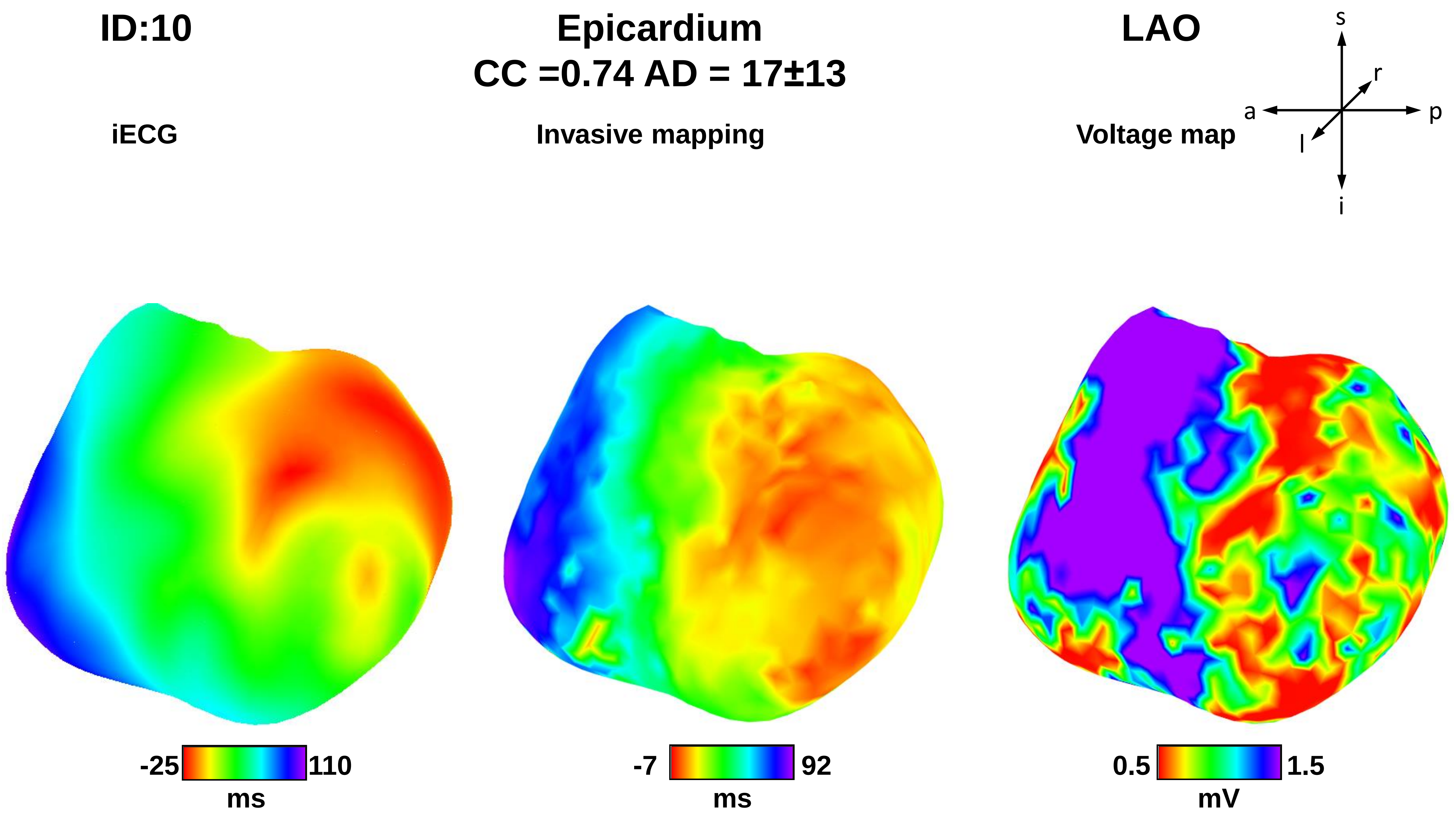
ID:10
Epicardium
CC =0.74 AD = 17±13
LAO
iECG
Invasive mapping
Voltage map
s
r
a
p
l
i
-25
110
ms
-7
92
ms
0.5
1.5
mV

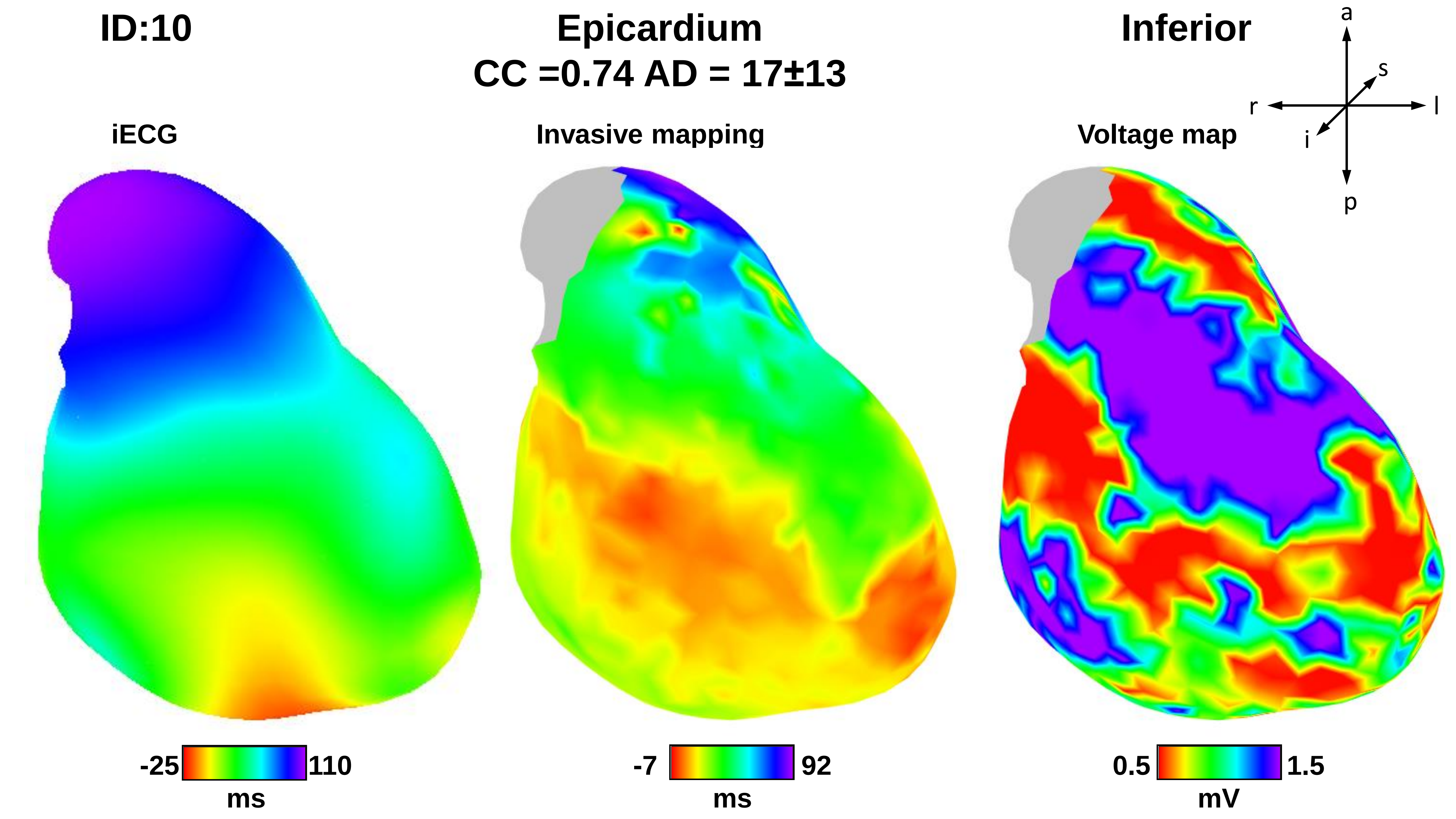
ID:10
Epicardium
CC =0.74 AD = 17±13
Inferior
a
s
r
l
i
p
iECG
Invasive mapping
Voltage map
-25
110
ms
-7
92
ms
0.5
1.5
mV

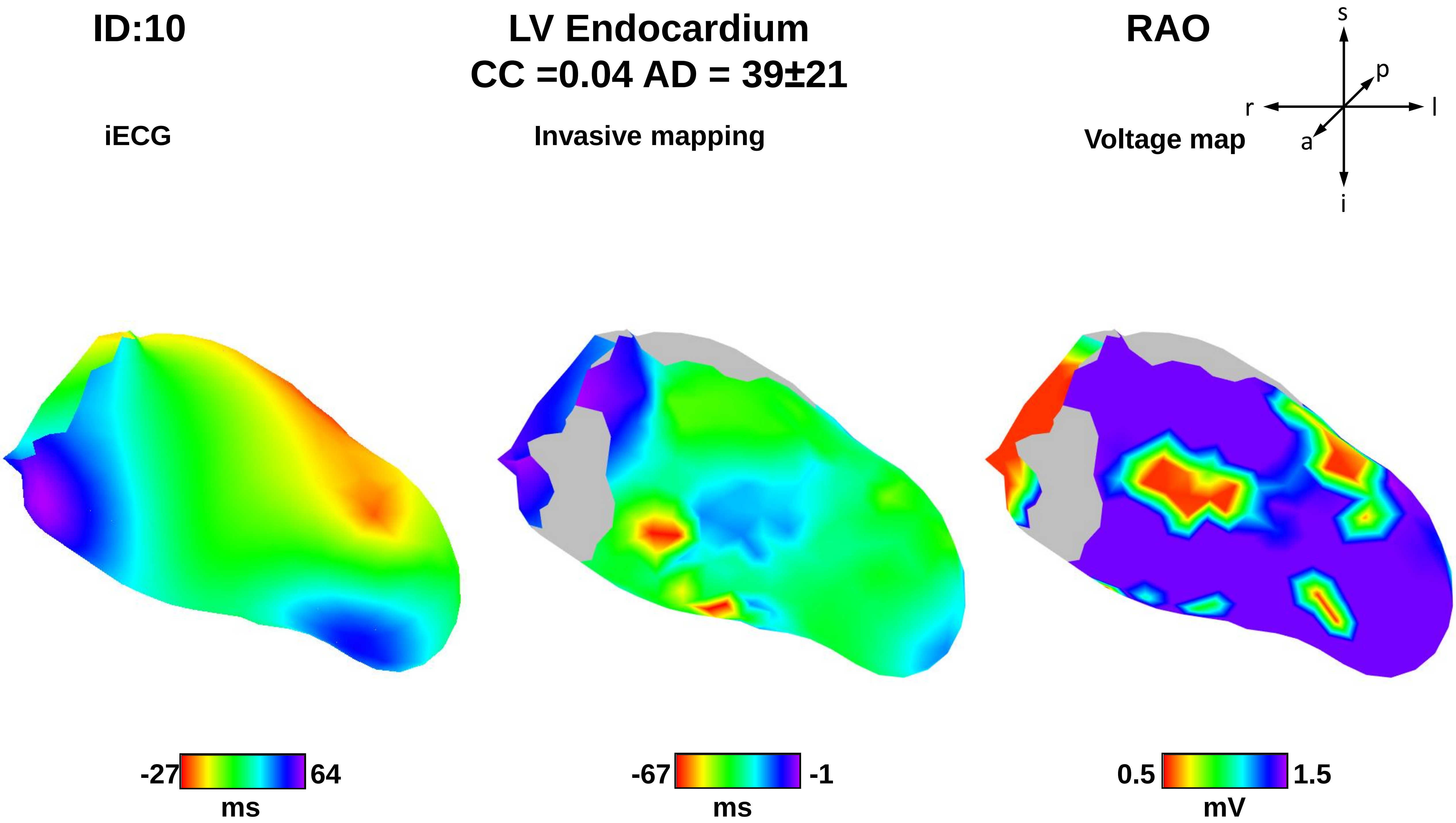

ID:10
LV Endocardium
CC =0.04 AD = 39±21
RAO
s
p
r
l
a
i
iECG
Invasive mapping
Voltage map
-27
64
ms
-67
-1
ms
0.5
1.5
mV

ID:10

LV Endocardium
CC =0.04 AD = 39±21

LAO

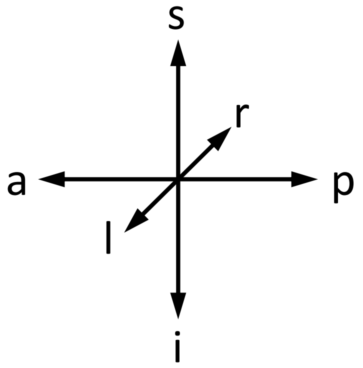

iECG **Invasive mapping** **Voltage map**

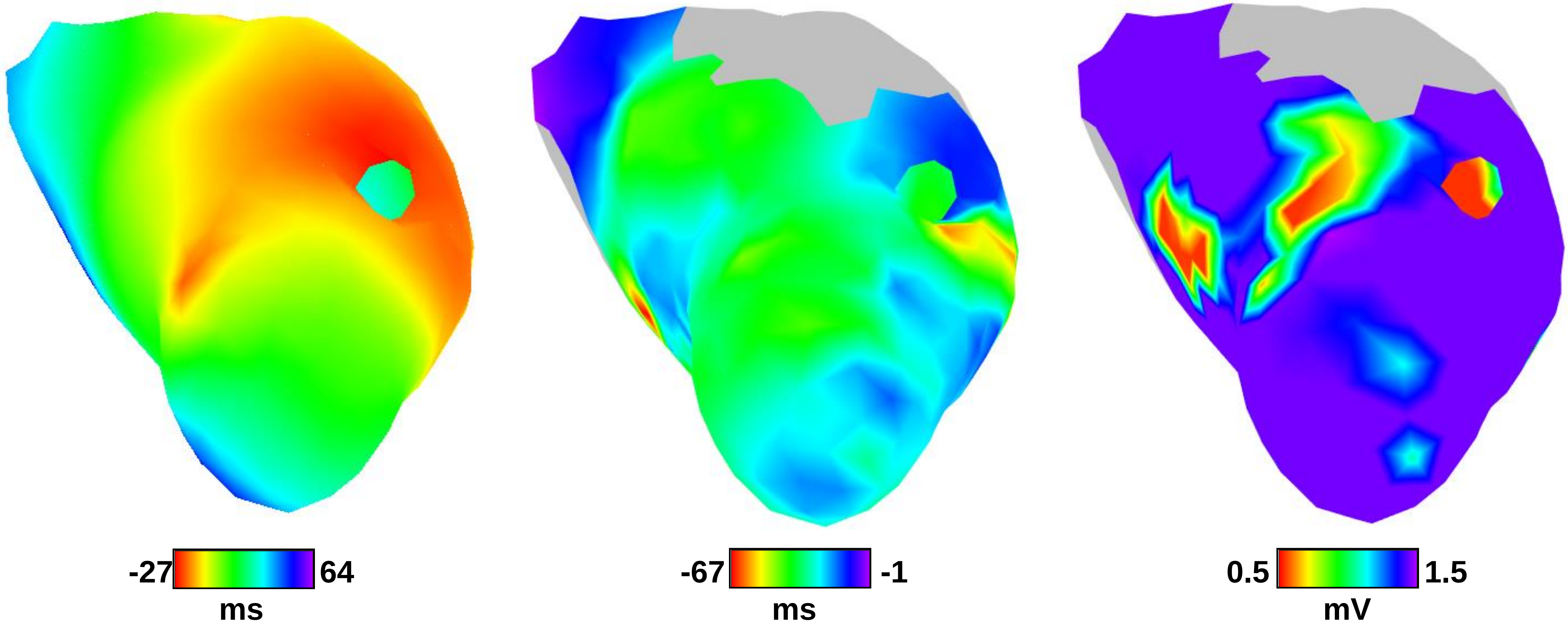

ID:10

LV Endocardium
CC =0.04 AD = 39±21

Inferior

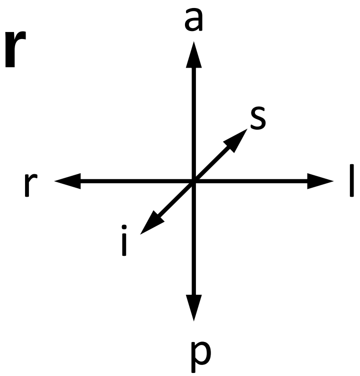

iECG

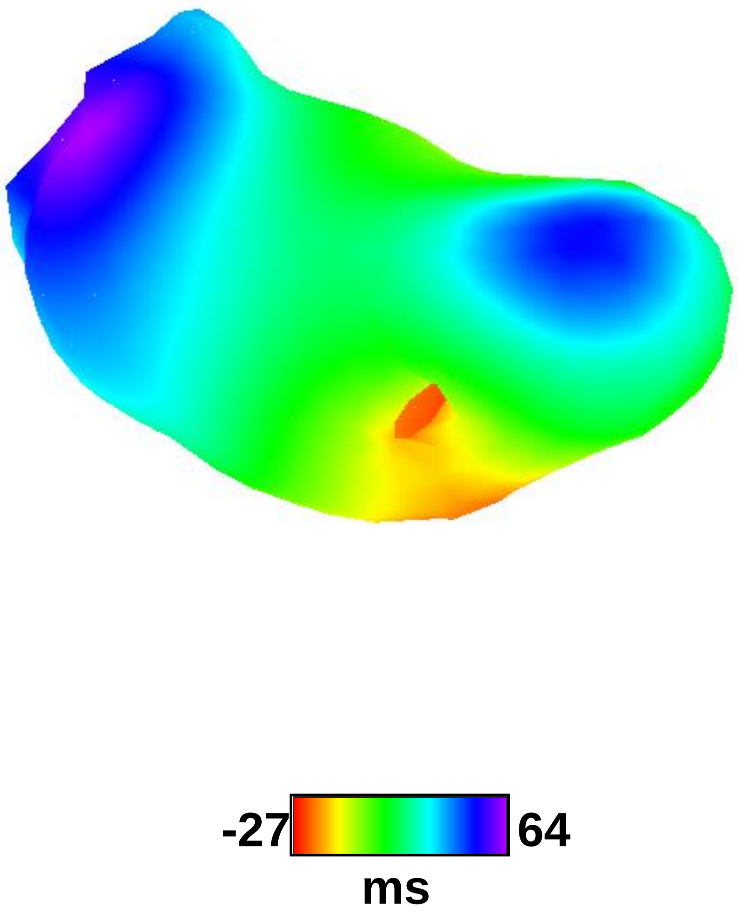

Invasive mapping

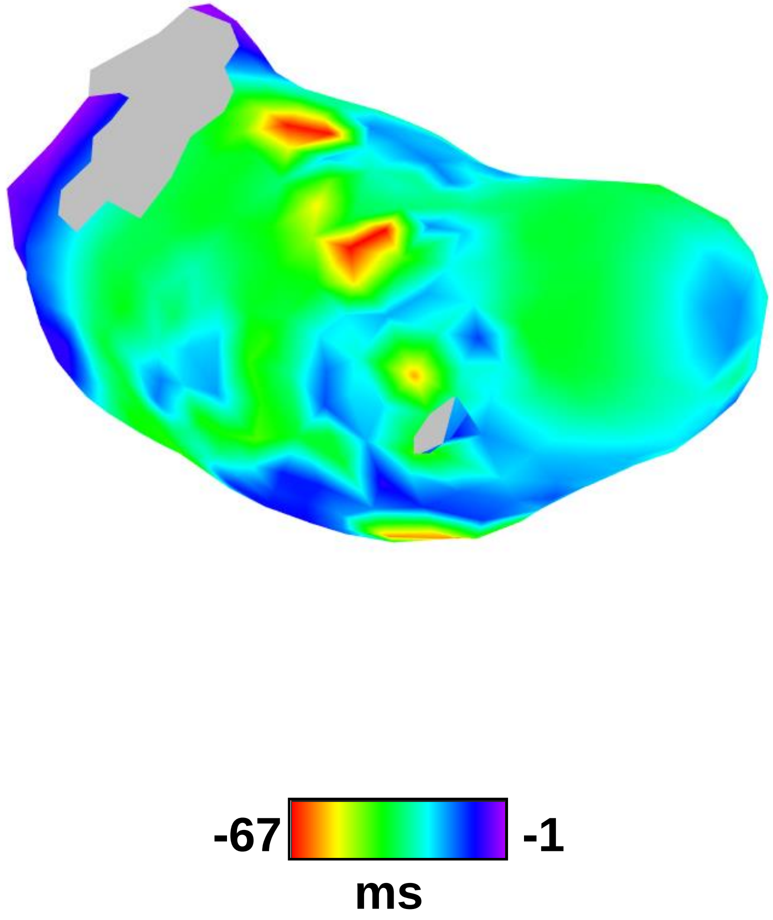

Voltage map

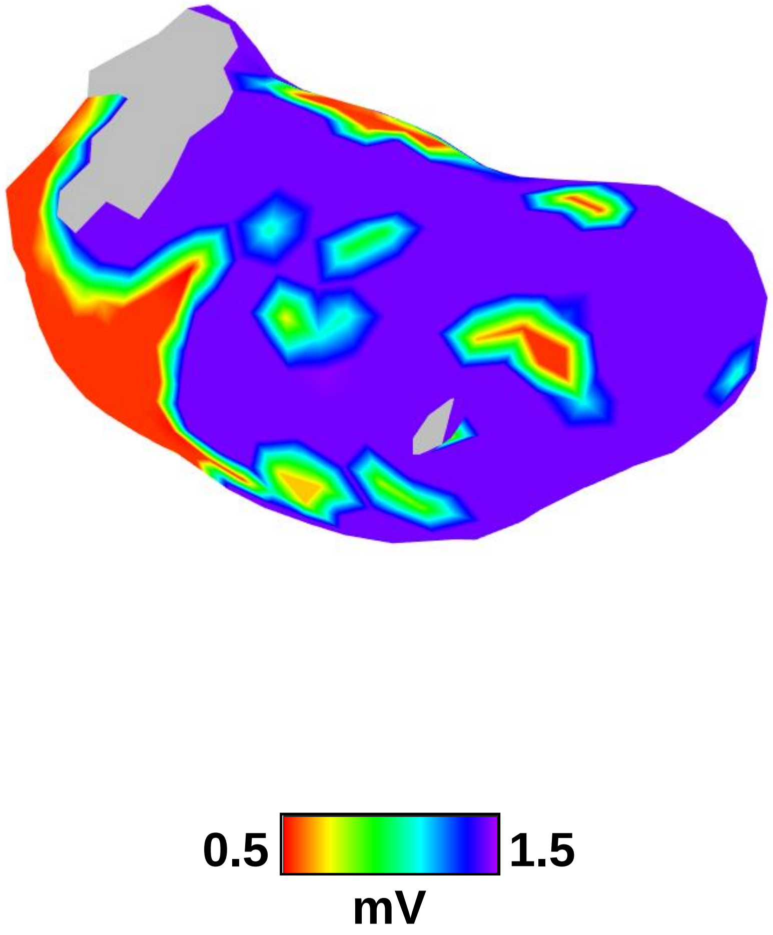

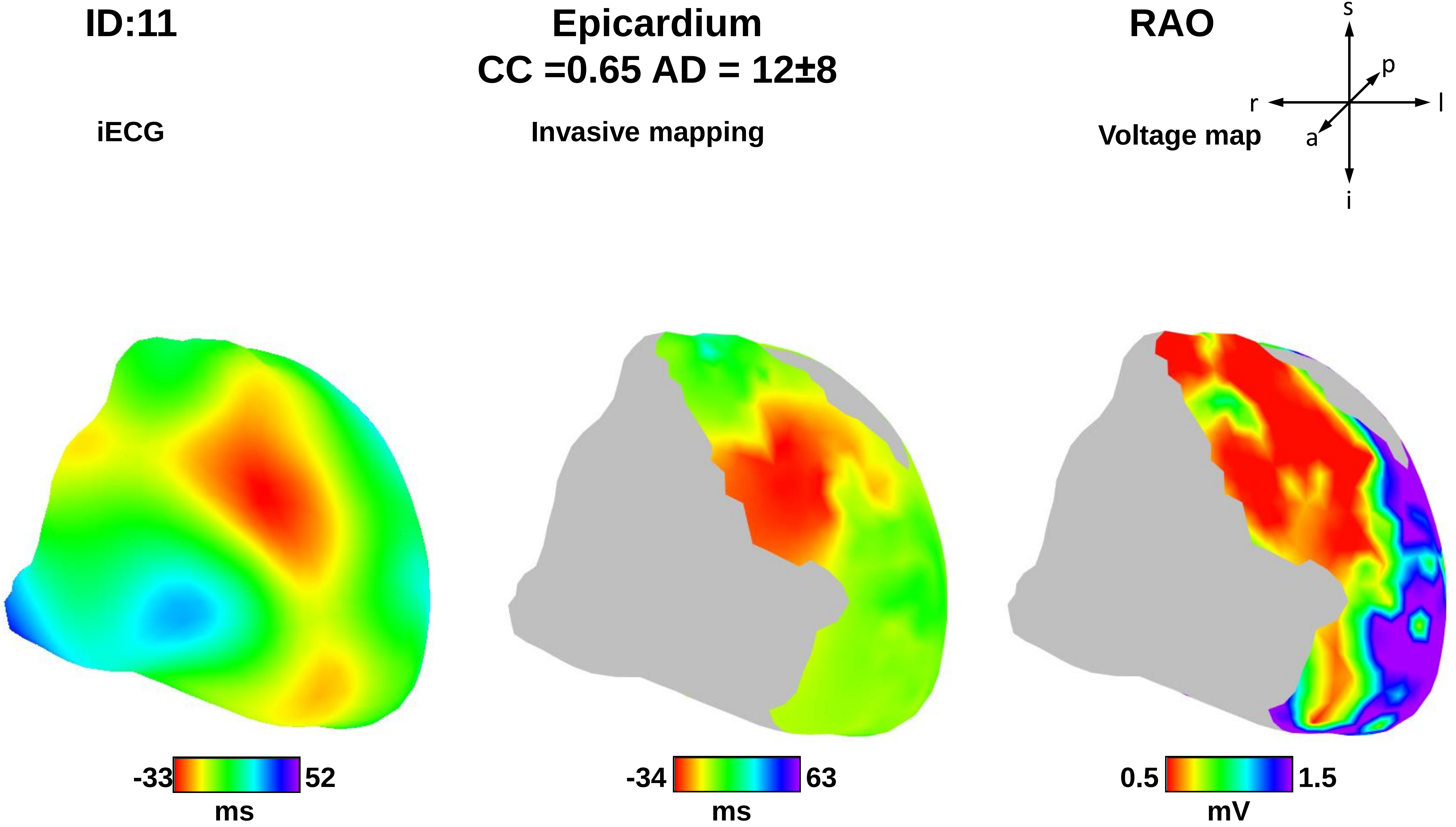
ID:11
Epicardium
CC =0.65 AD = 12±8
RAO
s
p
r
l
a
i
iECG
Invasive mapping
Voltage map
-33
52
ms
-34
63
ms
0.5
1.5
mV

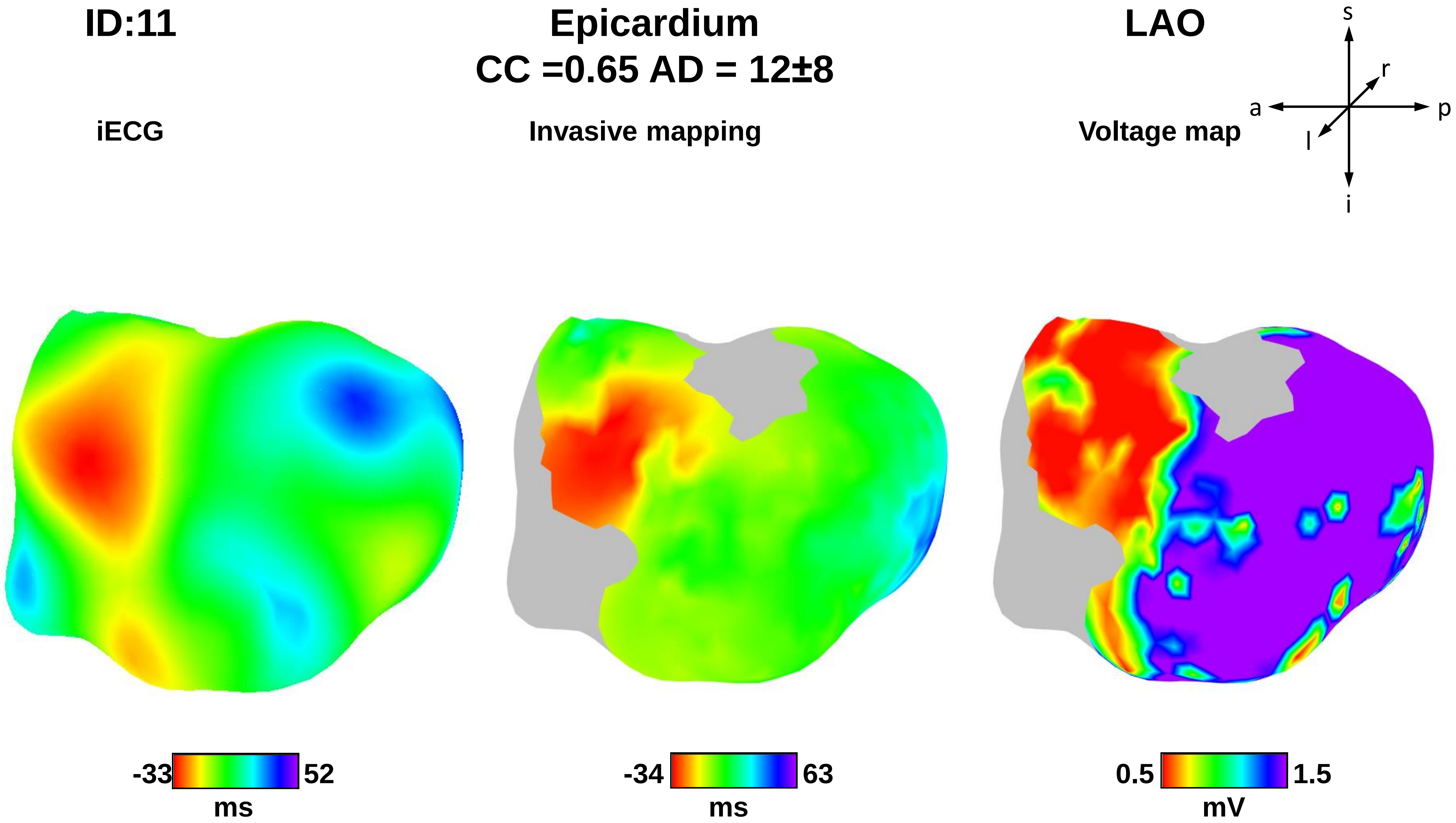
ID:11
Epicardium
CC =0.65 AD = 12±8
LAO
s
r
a
p
l
i
iECG
Invasive mapping
Voltage map
-33
52
ms
-34
63
ms
0.5
1.5
mV

ID:11 **Epicardium** **Inferior**

CC =0.65 AD = 12±8

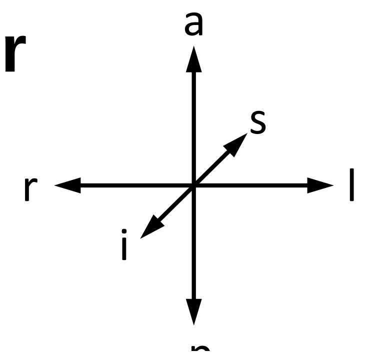

iECG **Invasive mapping** **Voltage map**

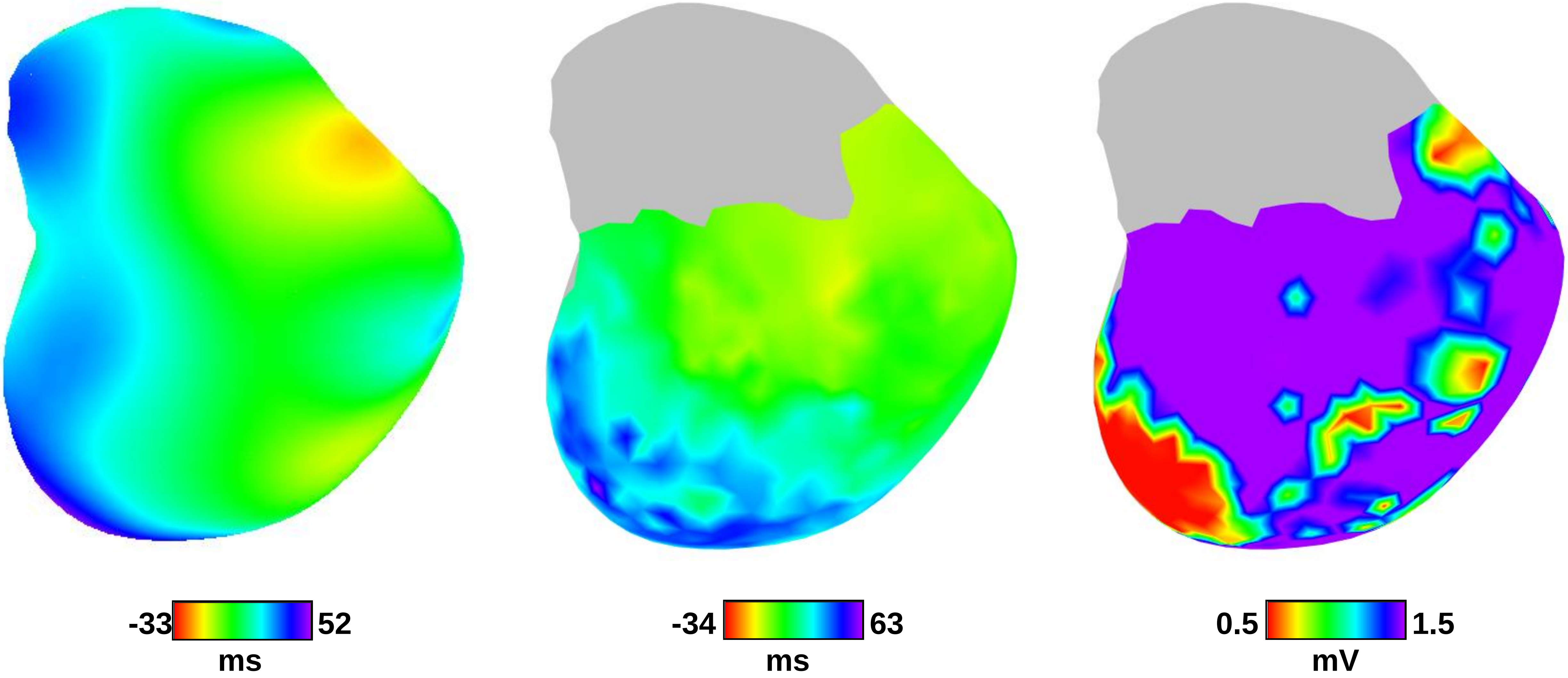

ID:11

LV Endocardium
CC =0.69 AD = 17±12

RAO

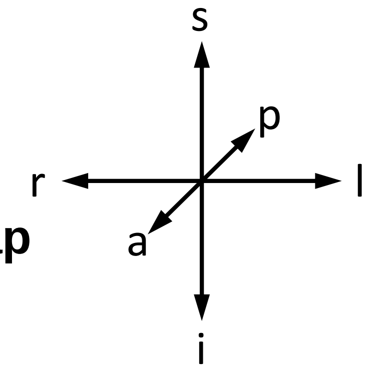

iECG | **Invasive mapping** | **Voltage map**

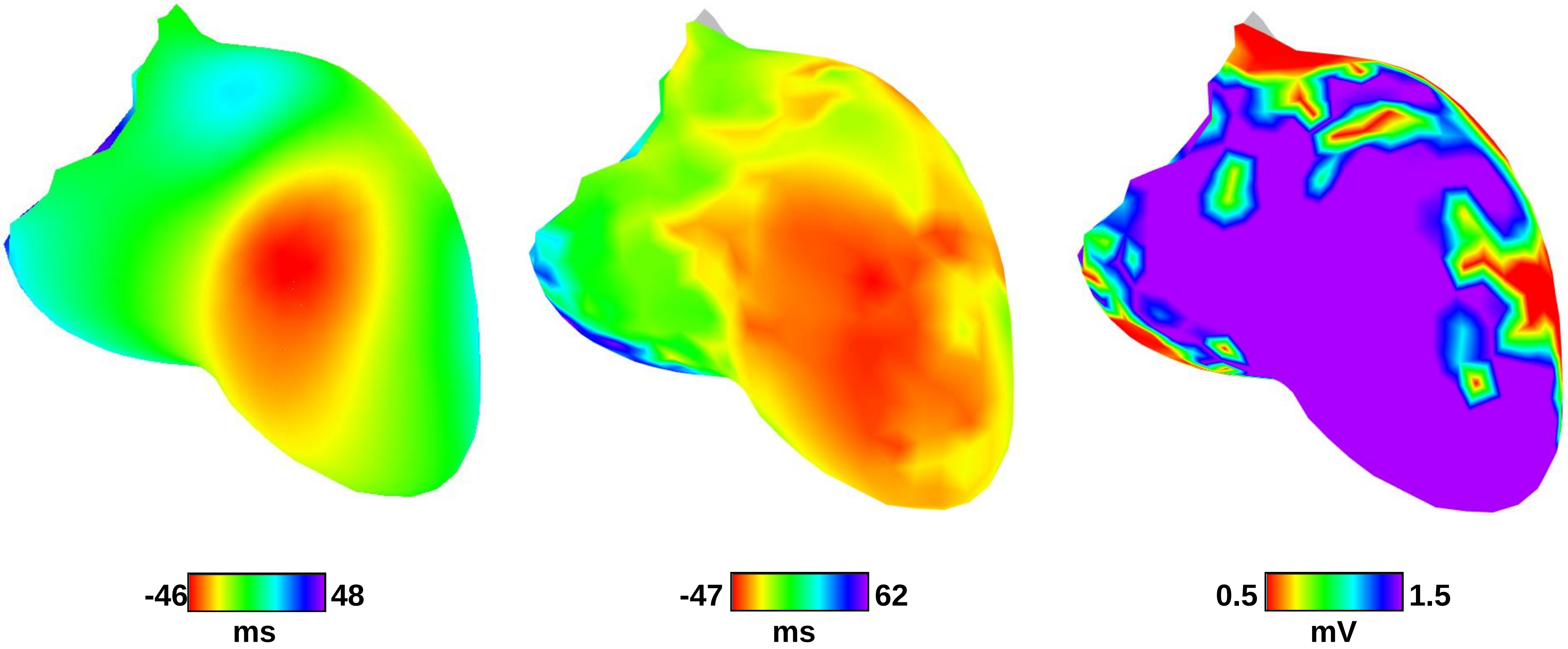

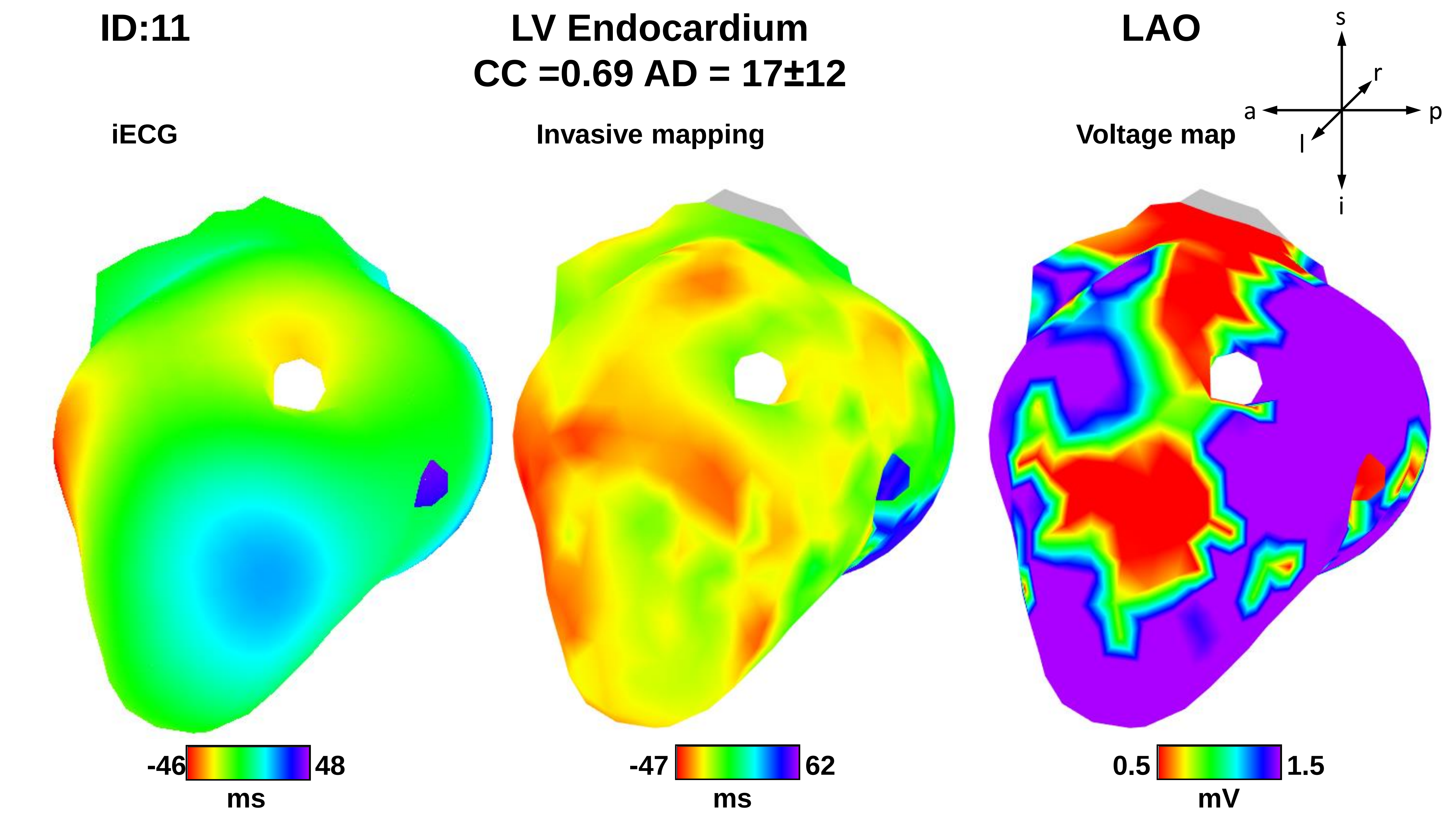
ID:11
LV Endocardium
CC =0.69 AD = 17±12
LAO
s
r
a
p
l
i
iECG
Invasive mapping
Voltage map
-46
48
ms
-47
62
ms
0.5
1.5
mV

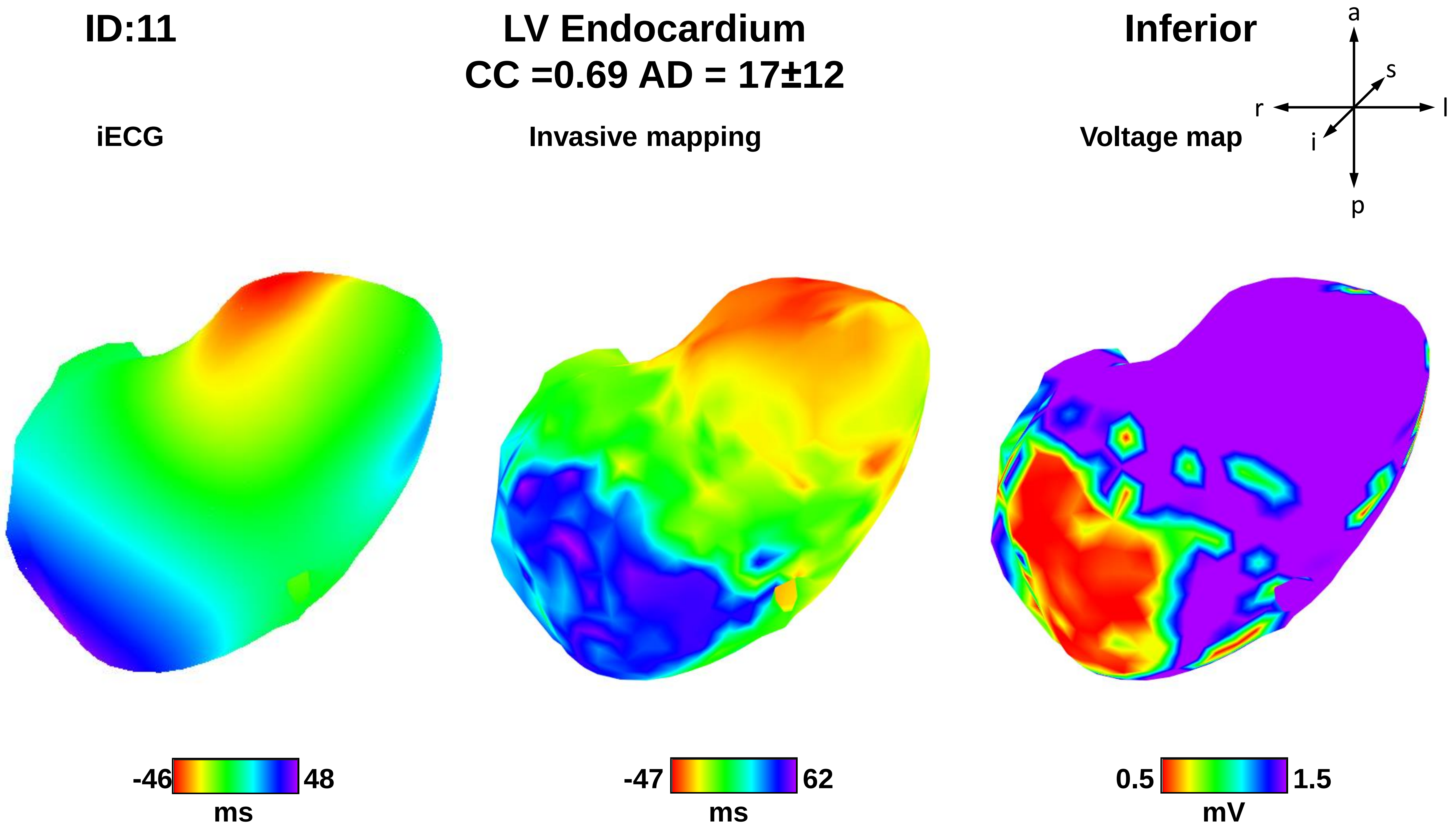
ID:11
LV Endocardium
CC =0.69 AD = 17±12
Inferior
iECG
Invasive mapping
Voltage map
a
s
r
l
i
p
-46
48
ms
-47
62
ms
0.5
1.5
mV

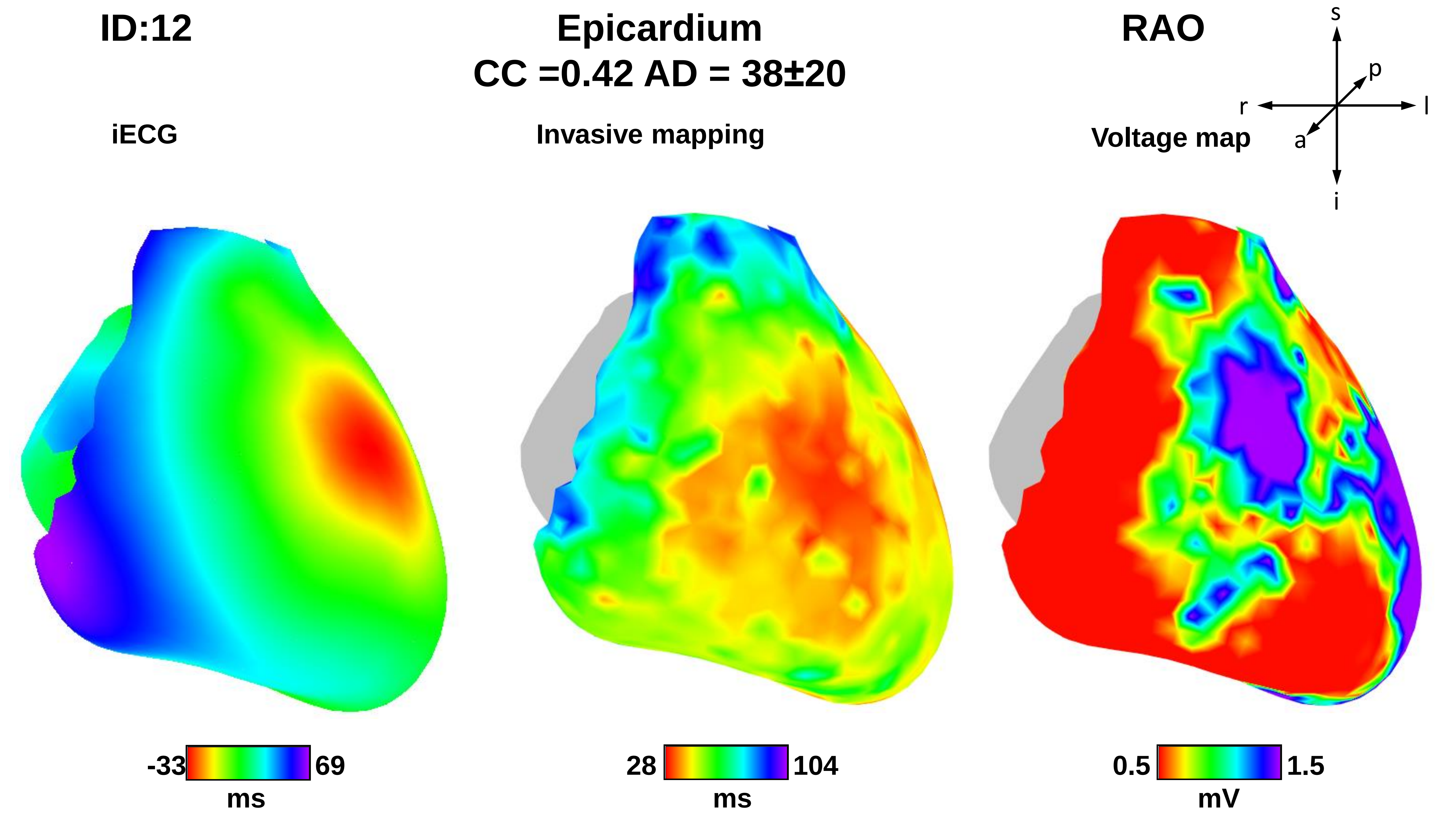
ID:12
Epicardium
CC =0.42 AD = 38±20
RAO
s
p
r
l
a
i
iECG
Invasive mapping
Voltage map
-33
69
ms
28
104
ms
0.5
1.5
mV

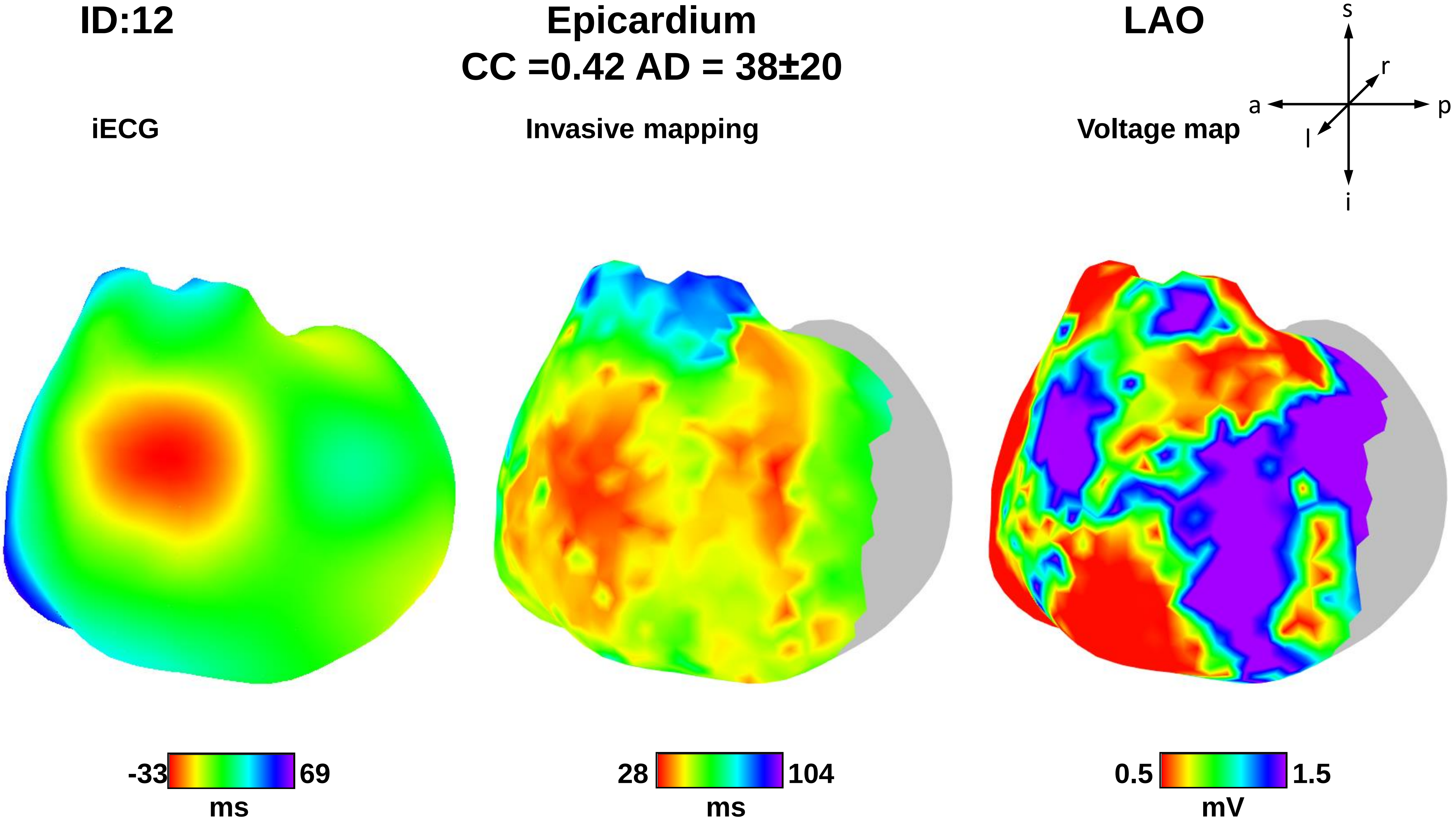
ID:12
Epicardium
CC =0.42 AD = 38±20
LAO
s
r
a
p
l
i
iECG
Invasive mapping
Voltage map
-33
69
ms
28
104
ms
0.5
1.5
mV

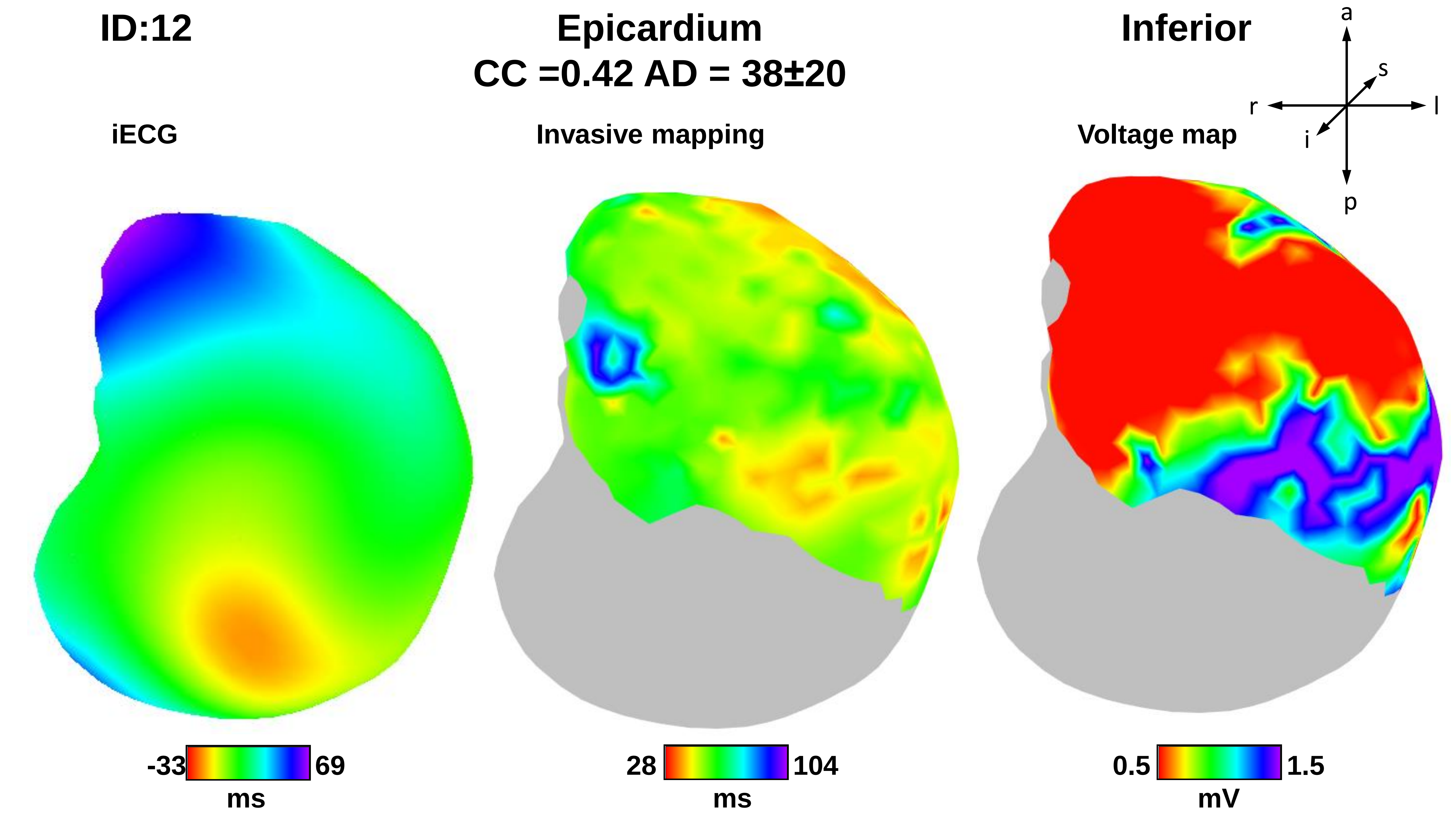
ID:12
Epicardium
CC =0.42 AD = 38±20
Inferior
iECG
Invasive mapping
Voltage map
a
s
r
l
i
p
-33
69
ms
28
104
ms
0.5
1.5
mV

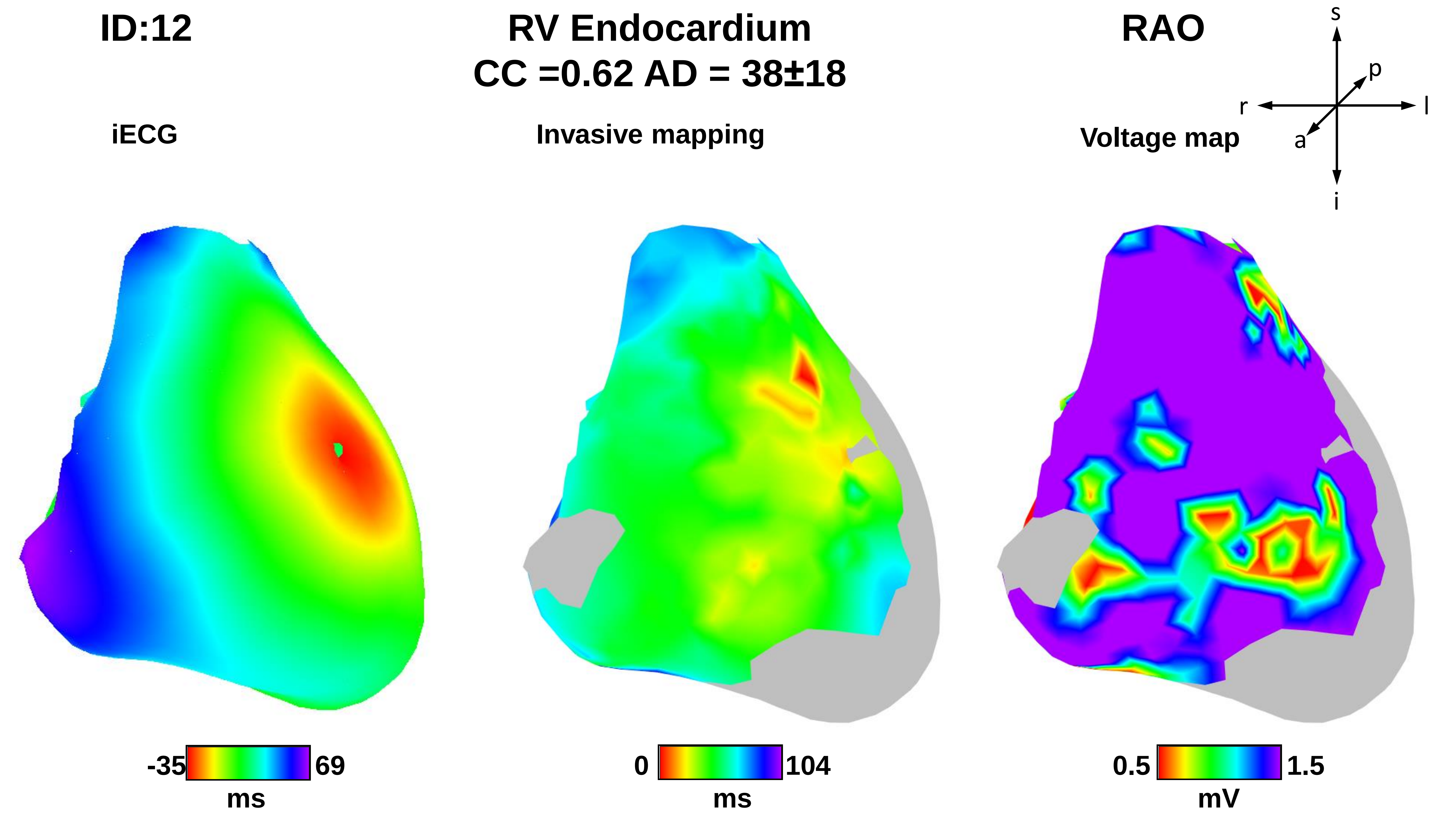
ID:12
RV Endocardium
CC =0.62 AD = 38±18
RAO
s
p
r
l
a
i
iECG
Invasive mapping
Voltage map
-35
69
ms
0
104
ms
0.5
1.5
mV

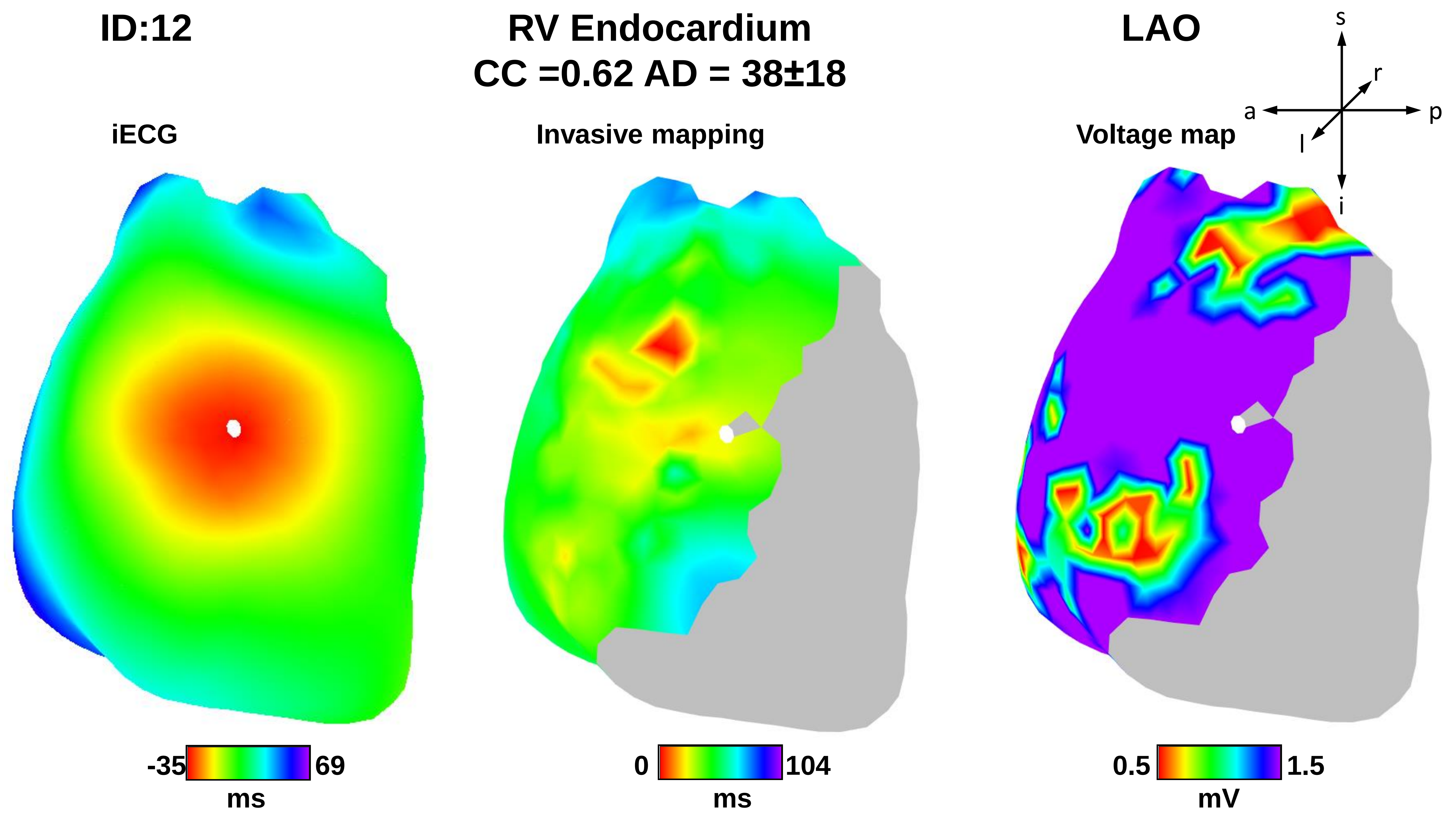

ID:12
RV Endocardium
CC =0.62 AD = 38±18
LAO
s
r
a
p
l
i
iECG
Invasive mapping
Voltage map
-35
69
ms
0
104
ms
0.5
1.5
mV

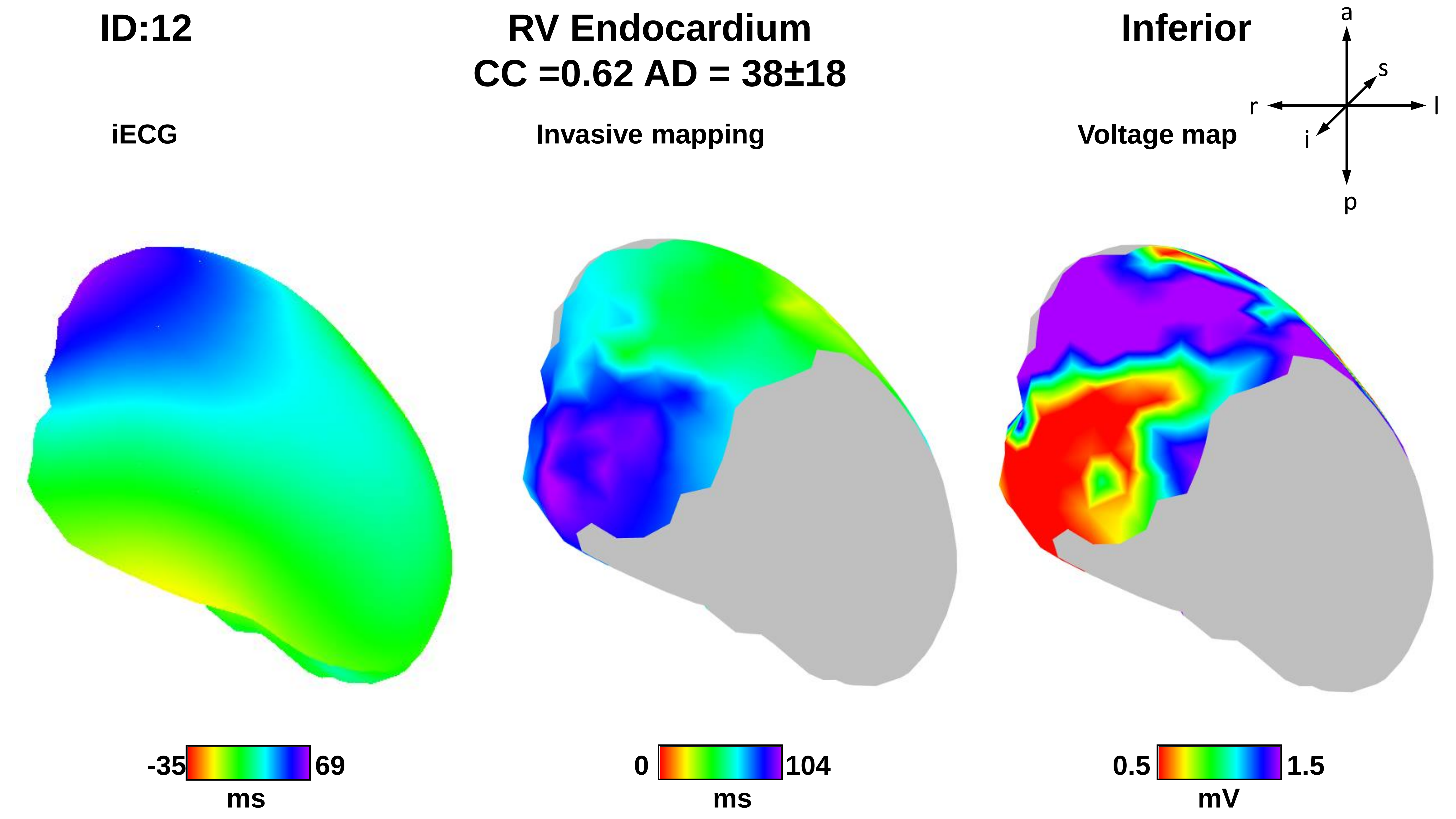

ID:12
RV Endocardium
CC =0.62 AD = 38±18
Inferior
a
s
r
l
i
p
iECG
Invasive mapping
Voltage map
-35
69
ms
0
104
ms
0.5
1.5
mV

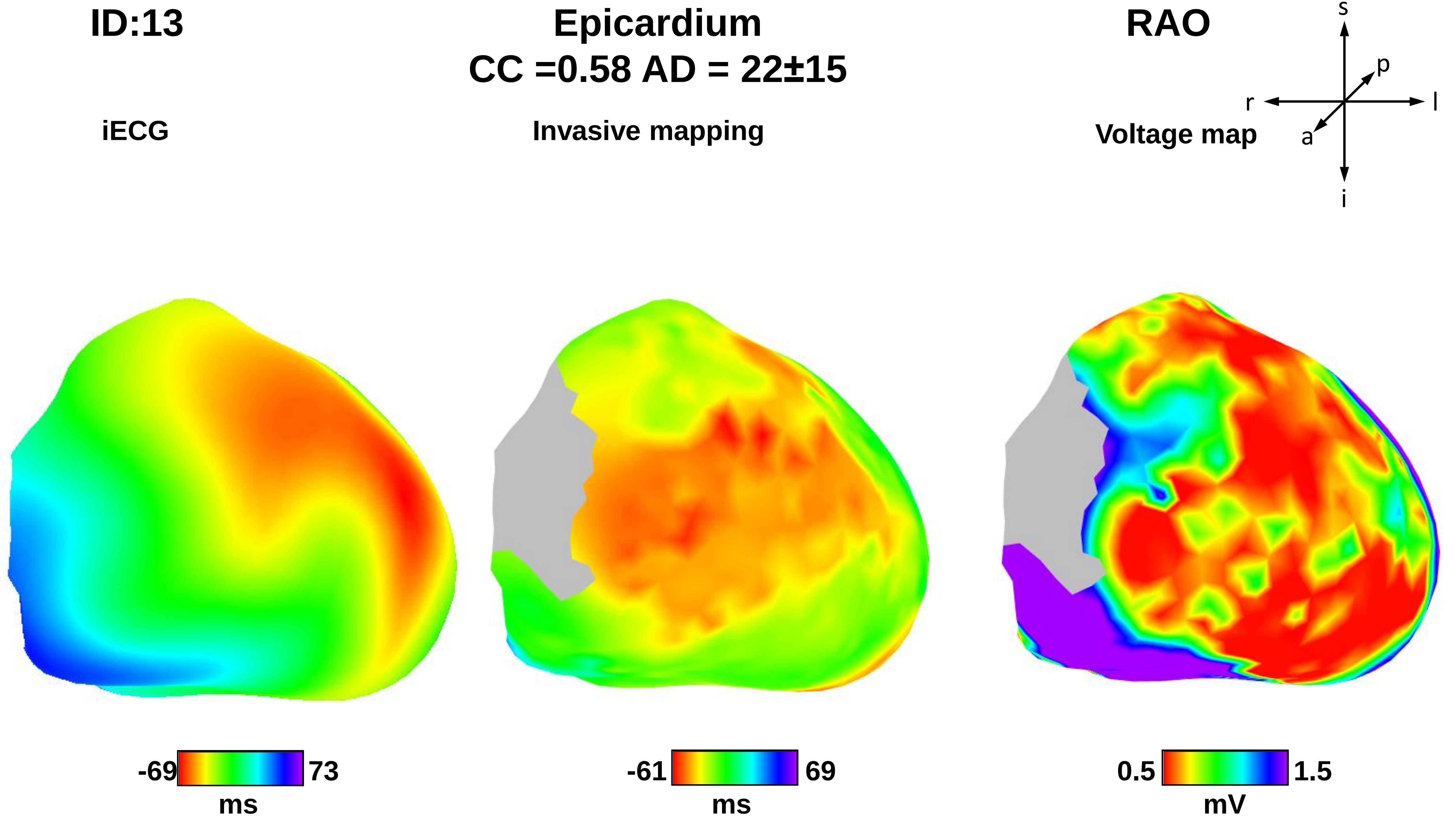

ID:13
Epicardium
CC =0.58 AD = 22±15
RAO
iECG
Invasive mapping
Voltage map
s
p
r
l
a
i
-69
73
ms
-61
69
ms
0.5
1.5
mV

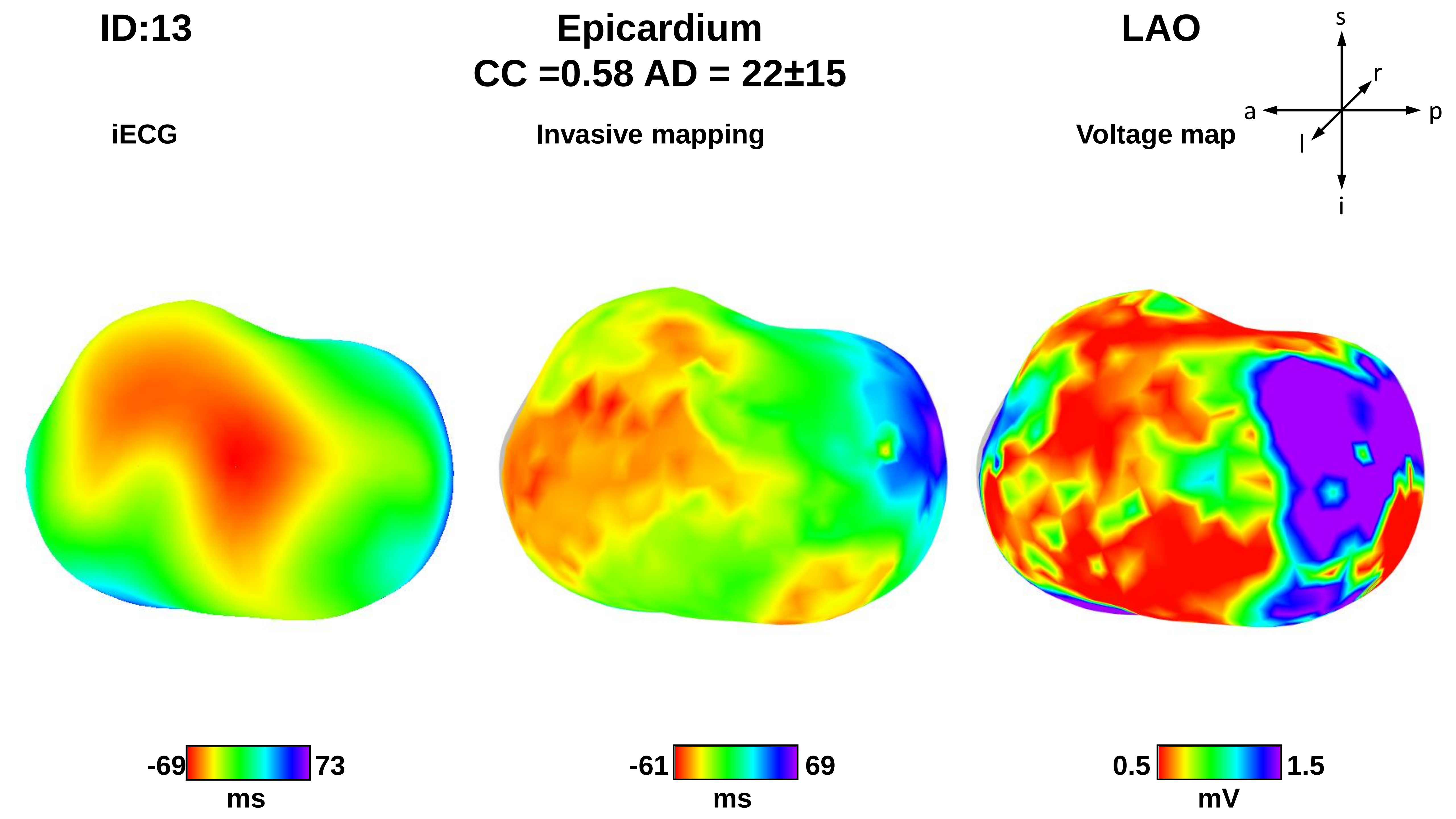
ID:13
Epicardium
CC =0.58 AD = 22±15
LAO
s
r
a
p
l
i
iECG
Invasive mapping
Voltage map
-69
73
ms
-61
69
ms
0.5
1.5
mV

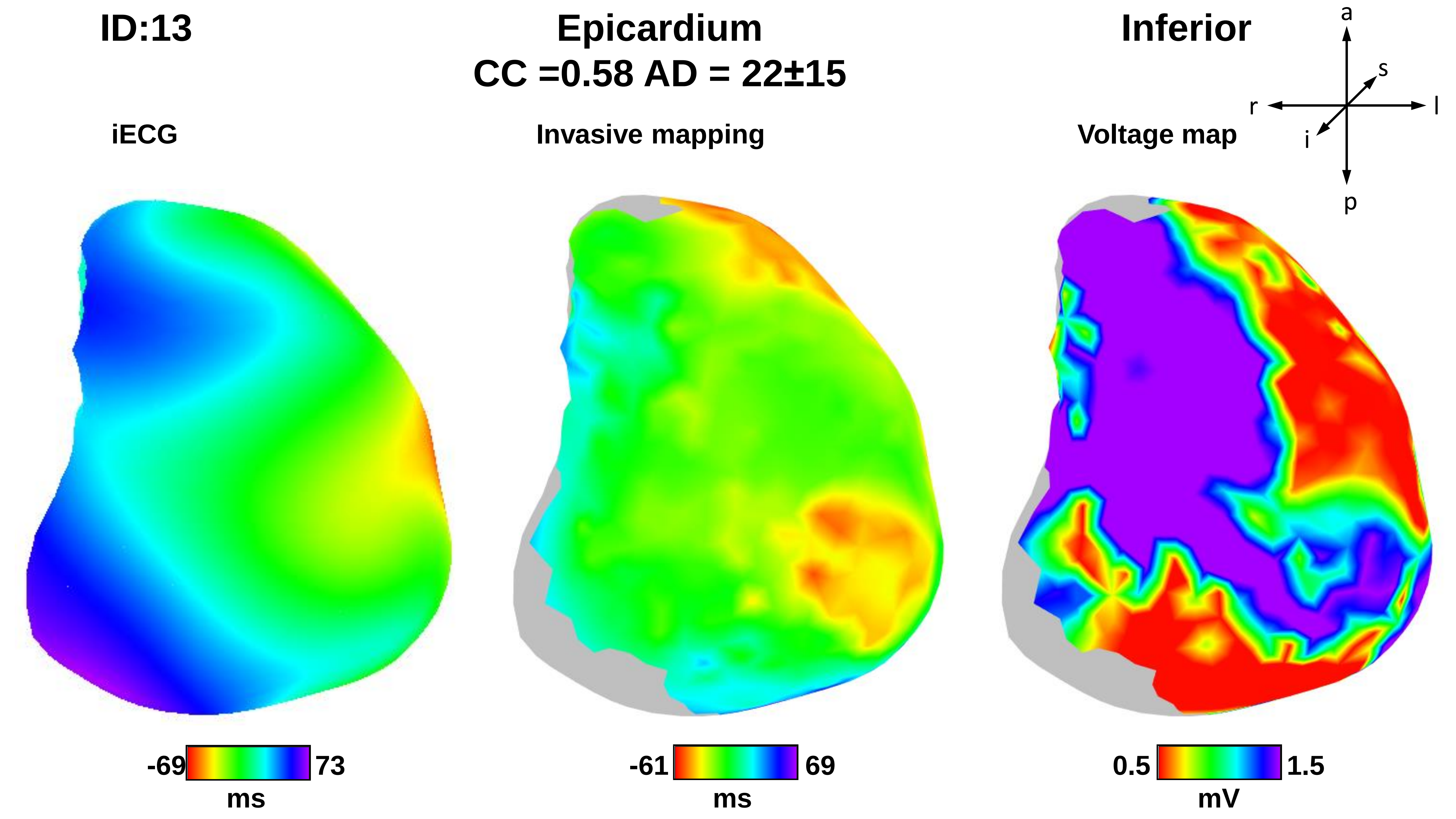
ID:13
Epicardium
CC =0.58 AD = 22±15
Inferior
a
s
r
l
i
p
iECG
Invasive mapping
Voltage map
-69
73
ms
-61
69
ms
0.5
1.5
mV

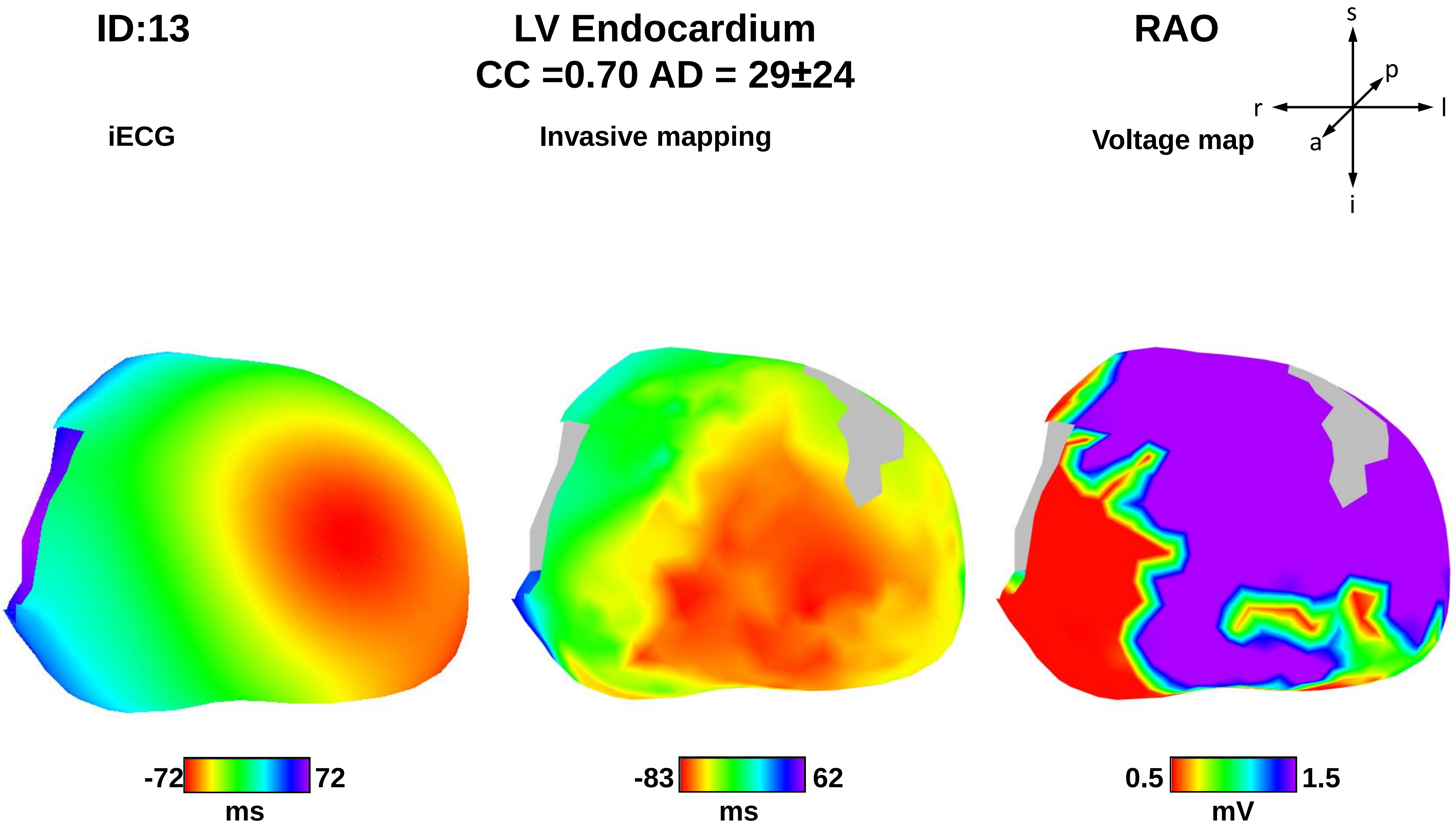
ID:13
LV Endocardium
CC =0.70 AD = 29±24
RAO
s
p
r
l
a
i
iECG
Invasive mapping
Voltage map
-72
72
ms
-83
62
ms
0.5
1.5
mV

ID:13

LV Endocardium
CC =0.70 AD = 29±24

LAO

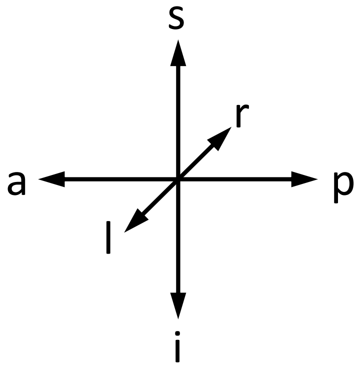

iECG

Invasive mapping

Voltage map

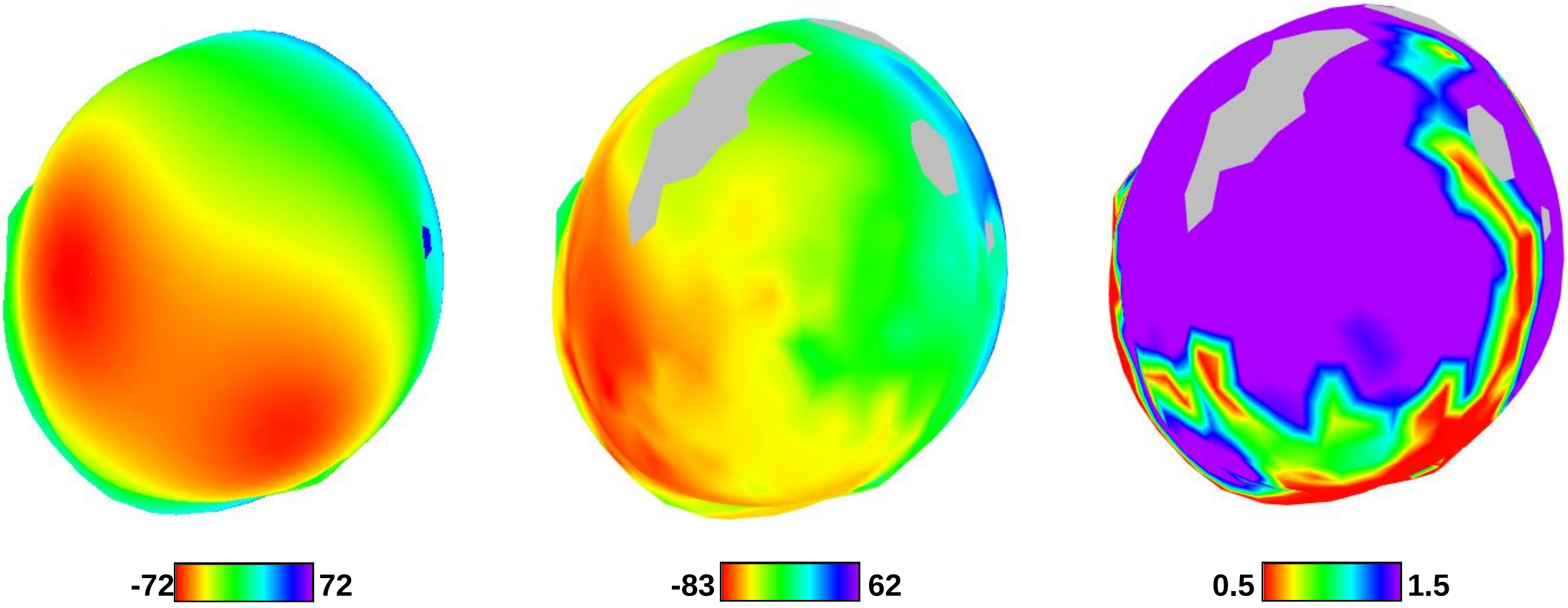

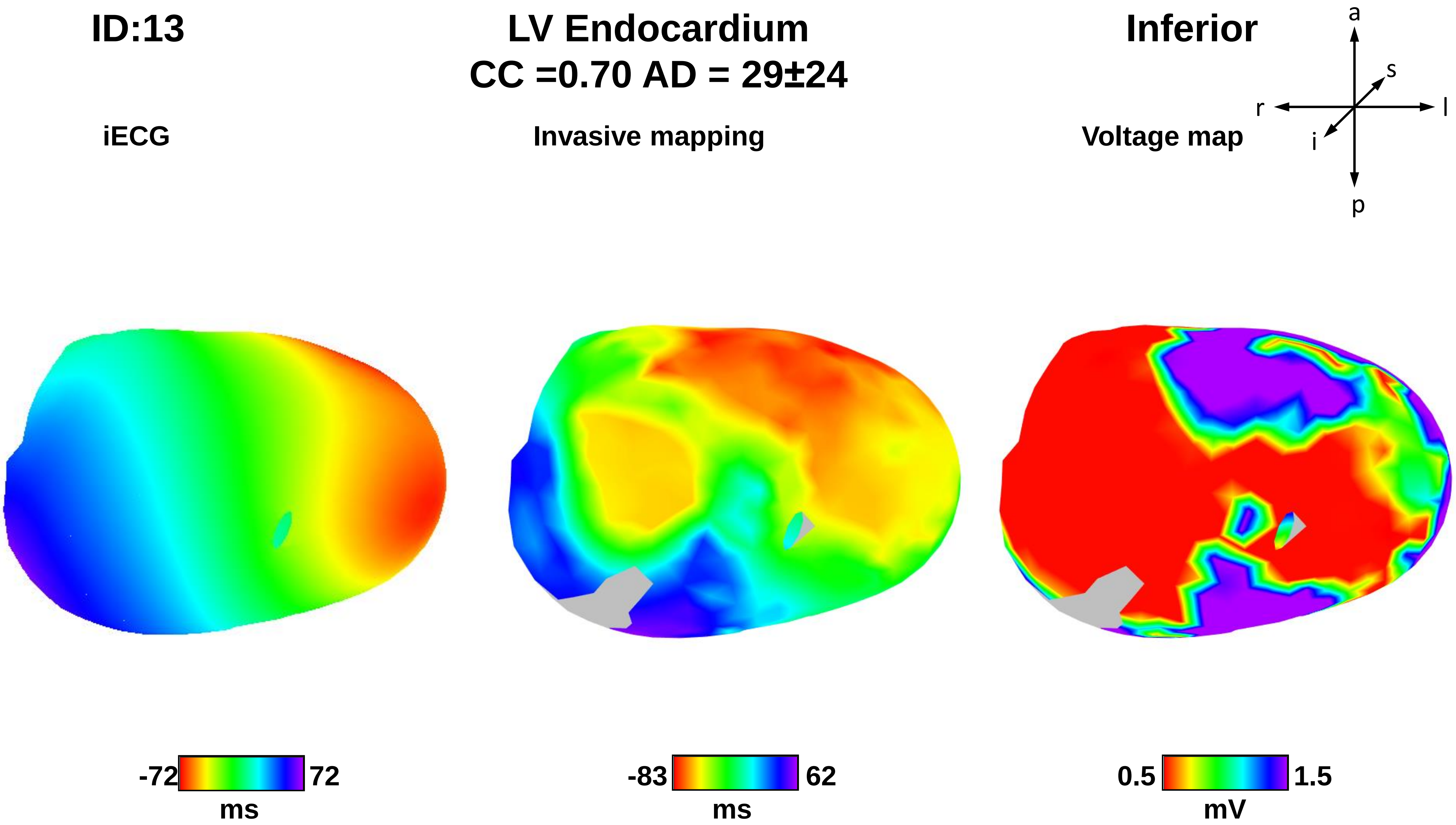
ID:13
LV Endocardium
CC =0.70 AD = 29±24
Inferior
iECG
Invasive mapping
Voltage map
a
s
r
l
i
p
-72
72
ms
-83
62
ms
0.5
1.5
mV
