# Supplementary material for: Comparing Non-invasive Inverse Electrocardiography With Invasive Endocardial and Epicardial Electroanatomical Mapping During Sinus Rhythm
Source: Front Physiol. 2021 Oct 4;12:730736. doi: 10.3389/fphys.2021.730736 (PMC8521153; doi:10.3389/fphys.2021.730736)
Supplement: Supplementary Figure 2 — Factors associated with correlation coefficients and absolute differences. [file Data_Sheet_2.docx]

**Supplementary Material**

**1.1 Supplementary Methods. Multi-wave initial estimation.**

Equivalent double layer (EDL) based inverse electrocardiography (*i*ECG) non-invasively estimates the activation sequence of **both** the endocardium and the epicardium simultaneously.(1-6) Local cardiac currents over the cardiac surface are simulated as transmembrane potentials, thereby directly relating to cardiac electrophysiology. Body surface potentials are then computed using the currents simulated by these transmembrane potentials. However, computed body surface potentials non-linearly depend on activation and recovery timings. Therefore, EDL-based *i*ECG requires an initial estimate, which can be constrained to ventricular electrophysiology. This initial estimation is then optimized through a dedicated Levenberg-Marquardt algorithm wherein the differences between recorded and computed body surface potentials are minimized by tuning local activation and recovery timings. Therefore, a second order Tikhonov regularization was performed with regularization parameter chosen that the value is close to $5*{10}^{-6}$mV^2^ms^2^/m^2^, which has been shown empirically to correspond to realistic smoothness in earlier studies. (3, 5, 7, 8)

In our study we optimized the initial estimation for His-Purkinje mediated ventricular activation. In this supplementary document we describe the new multi-wave *i*ECG method for the estimation during sinus rhythm. In multi-wave *i*ECG, physiological and anatomical information about the His-Purkinje system is taken into account to mimic His-Purkinje mediated initiation of ventricular activation. Multiple wavefronts are initiated at distinct endocardial regions associated with a dense distribution of Purkinje-myocardial junctions, based on the regions described by Durrer et al (9-11). Several endocardial regions are associated with early ventricular activation: the bases of the two left ventricular papillary muscles, the right ventricular moderator band and several septal regions.(9-18) Therefore, in multi-wave *i*ECG, anatomical structures associated with early ventricular activation are incorporated in the ventricular model (Figure, Panel C).

Subject specific ventricular geometries were created (Figure, Panel A and B) and distinct foci were localized at the insertion of the two left ventricular papillary muscles and the moderator band on the ventricular free wall (Figure Panel C). On the septal wall, six regions with a radius of 10 mm were selected containing multiple potential foci (Figure, Panel C). At the left ventricular septal wall, one region was localized at the inferior one-third from base to apex of the septal wall and one region was localized at superior one-third from base to apex of the antero-septal wall and two other regions were localized between those locations. At the right ventricular septal wall, the localized region was close to the RV apex and at the middle of the RV septal wall. Per septal region, one focus was selected, as described in the following paragraph.

Using the fastest route algorithm (FRA) with multiple distinct foci and initial activation timings, the initial activation sequence was computed.(5, 19) A set myocardial conduction velocity is used. In this study, activation sequences were calculated using a myocardial conduction velocity of 0.85 mm/ms.(12, 14, 20, 21) To account for increased subendocardial conduction velocities, myocardial conduction velocity in close vicinity of a focus was set at 1.7 mm/ms.(14, 15, 21, 22) With these measures we aimed to take into account the complex nature of His-Purkinje mediated activation.(23, 24) An anisotropy ratio of two was also used, meaning that the conduction velocity perpendicular to the myocardial fibers was two times lower than conduction velocity longitudinal to myocardial fibers.(20, 25-27)

First, activation sequences emerging from the insertion of the papillary muscles and the ventricular free wall insertion of the moderator band were calculated. Initial timing of these sites was tested throughout the first 35 ms of QRS duration, as the His-Purkinje system initiates ventricular activation. Then, per septal region, activation sequences from all potential foci were merged with activation sequences emerging from the papillary muscles and the moderator band. Initial timing of septal foci was tested throughout the first 25 ms of QRS duration.(9-13, 17) Activation timings may differ per initial region as observed in mapping studies, where the septum is mostly the first structure to be activated. Therefore, a smaller time range is tested to determine the initial timing of the septal foci. The procedure resulted in nine single foci activation sequences initiated at one of the His-Purkinje associated regions with distinct initial activation timings and were selected based on the best matching computed and recorded BSPM.

To account for inter-individual diversity in His-Purkinje anatomy and the number of active foci at the endocardium affected by for example bundle branch blocks, all possible permutations of active foci were tested.(16-18) This resulted in 511(=(2^9)-1 (all foci inactive)) possible permutations of foci and the resulting merged activation sequences were tested. Merged activation sequences with a total activation duration >115% and <85% of **measured** **QRS** duration in the BSPM were excluded. The activation sequence yielding highest correlation between recorded BSPM and computed BSPM was selected as the initial estimation and used as input for the optimization procedure as described in the main manuscript.


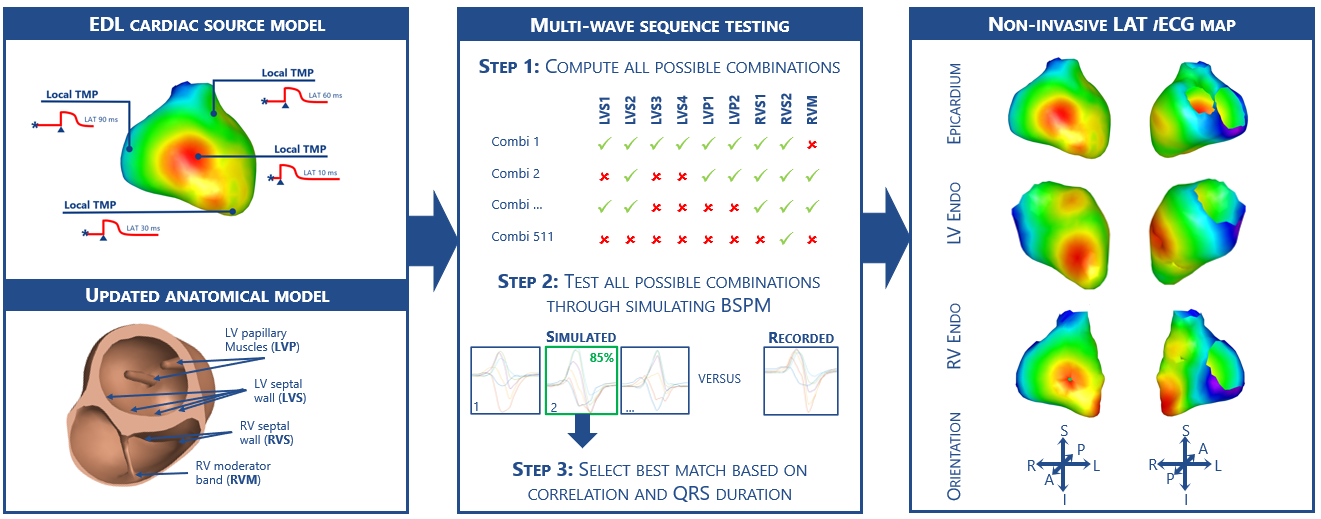


**1.2 Figure –** Multi-wave *iE*CG. First, (cardiac) imaging and body surface potential maps (BSPM) data are acquired (Panel A). Using the volume conductor, BSPM are simulated. Multi-wave *i*ECG selects the best matching activation sequence by testing 511 combinations of initial activation sequences. The output of the procedure is local activation timing maps (D). In Panel D, three examples of the 511 possible activation maps are shown with respectively six, four or two initial sites of activation. Local activation timing is depicted from red (early activation) to (blue latest activation).

**1.3 References**

1. Geselowitz DB. On the Theory of the Electrocardiogram. Proc IEEE. 1989;77/6:857-76.

2. Geselowitz DB. Description of cardiac sources in anisotropic cardiac muscle. Application of bidomain model. Journal of Electrocardiology. 1992;25 Sup.:65-7.

3. Huiskamp GJM, van Oosterom A. The depolarization sequence of the human heart surface computed from measured body surface potentials. IEEE Transactions on Biomedical Engineering. 1988;35(12):1047-58.

4. Huiskamp GJM, Greensite F. A New Method for Myocardial Activation Imaging. IEEE Trans Biomed Eng. 1997;44(6):433-46.

5. van Dam PM, Oostendorp TF, Linnenbank AC, van Oosterom A. Non-invasive imaging of cardiac activation and recovery. Annals Biomedical Engineering. 2009;37(9):1739-56.

6. Janssen AM, Potyagaylo D, Dössel O, Oostendorp TF. Assessment of the equivalent dipole layer source model in the reconstruction of cardiac activation times on the basis of BSPMs produced by an anisotropic model of the heart. Medical & biological engineering & computing. 2018;56(6):1013-25.

7. Cuppen JJM. Calculating the Isochrones of Ventricular Depolarization. SIAM J Scie Stat Comp. 1984;5:105-20.

8. Roozen H, van Oosterom A. Computing the activation sequence at the ventricular heart surface from body surface potentials. Med Biol Eng & Comput. 1987;25:250-60.

9. Tawara S. Das Reizleitungssystem des Säugetierherzens. Eine Anatomisch-Histologische Studie über das Atrioventrikularbündel und die Purkinjeschen Faden. Jena: Gustav Fischer; 1906.

10. Keith A, Flack M. The auriculo-ventricular bundle of the human heart. The Lancet. 1906;168(4328):359-64.

11. Ho SY, McCARTHY KP, Ansari A, Thomas PS, Sánchez-Quintana D. Anatomy of the atrioventricular node and atrioventricular conduction system. International Journal of Bifurcation and Chaos. 2003;13(12):3665-74.

12. Durrer D, van Dam RT, Freud GE, Janse MJ, Meijler FL, Arzbaecher RC. Total excitation of the isolated human heart. Circulation. 1970;41:899-912.

13. Wyndham CRM, Smith T, Saxema A, Engleman S, Levitsky RM, Rosen KM. Epicardial activation of the intact human heart without conduction defect. Circulation. 1979;59/1:161-8.

14. Taccardi B, Punske BB, Macchi E, MacLeod RS, Ershler PR. Epicardial and intramural excitation during ventricular pacing: effect of myocardial structure. American Journal of Physiology-Heart and Circulatory Physiology. 2008;294(4):H1753-H66.

15. Porta-Sánchez A, Angaran P, Massé S, Nair K, Farid T, Umapathy K, et al. The effect of left ventricular pacing on transmural activation delay in myopathic human hearts. Ep Europace. 2018;20(4):719-28.

16. Cassidy DM, Vassallo JA, Miller JM, Poll DS, Buxton AE, Marchlinski FE, et al. Endocardial catheter mapping in patients in sinus rhythm: relationship to underlying heart disease and ventricular arrhythmias. Circulation. 1986;73(4):645-52.

17. Hatala R, Savard P, Tremblay G, Page P, Cardinal R, Molin F, et al. Three Distinct Patterns of Ventricular Activation in Infarcted Human Hearts : An Intraoperative Cardiac Mapping Study During Sinus Rhythm. Circulation. 1995;91(5):1480-94.

18. Kastor JA, Goldreyer BN, Moore EN, Shelburne J, Manchester J. Intraventricular conduction in man studied with an endocardial electrode catheter mapping technique. Patients with normal QRS and right bundle branch block. Circulation. 1975;51(5):786-96.

19. van Dam PM, Oostendorp TF, van Oosterom A. Application of the fastest route algorithm in the interactive simulation of the effect of local ischemia on the ECG. Med Biol Eng Comput. 2009;47(1):11-20.

20. Greenbaum RA, Ho SY, Gibson DG, Becker AE, Anderson RH. Left ventricular fibre architecture in man. Br Heart J. 1981;45:248-63.

21. Myerburg RJ, Gelband H, Nilsson K, Castellanos A, Morales AR, Bassett AL. The role of canine superficial ventricular muscle fibers in endocardial impulse distribution. Circulation research. 1978;42(1):27-35.

22. Strik M, Ploux S, Vernooy K, Prinzen FW. Cardiac resynchronization therapy. Circulation Journal. 2011;75(6):1297-304.

23. Myerburg RJ. The gating mechanism in the distal atrioventricular conducting system. Circulation. 1971;43(6):955-60.

24. Veenstra RD, Joyner RW, Rawling DA. Purkinje and ventricular activation sequences of canine papillary muscle. Effects of quinidine and calcium on the Purkinje-ventricular conduction delay. Circ Res. 1984;54(5):500-15.

25. Sano T, Takayama N, Shimamoto T. Directional Differences of Conduction Velocity in the Cardiac Ventricular Syncytium Studied by Microelectrodes. Circulation Research. 1959;VII:262-7.

26. Roberts D, Hersh L, Scher A. Influence of cardiac fiber orientation on wavefront voltage, conduction velocity, and tissue resistivity in the dog. Circulation Research. 1979;44:701-12.

27. Clerc L. Directional differences of impulse spread in trabecular muscle from mammalian heart. J Physiol. 1976;255:335-46.

**2.1 Supplementary Table 1. Study population summary**

| Study Population | Mean ± SD, median [IQR] or n (%) | |
| --- | --- | --- |
| Demographics | | |
| Age (years) | | 48 ± 20 |
| Male sex | | 10 (77) |
| Sustained ventricular tachycardia | | 10 (77) |
| Symptomatic premature ventricular complexes | | 3 (23) |
| 12-lead ECG | |  |
| Sinus rhythm | | 10 (77) |
| Atrial pacing | | 3 (23) |
| QRS duration (ms) | | 112 ± 26 |
| Electroanatomical Mapping | |  |
| Epicardial mapping (number of electrograms) | | 4611 [3369 - 5633] |
| RV endocardial mapping (number of electrograms) | | 910 [280 - 1638] |
| LV endocardial mapping (number of electrograms) | | 605 [247 - 1412] |
| Carto mapping system | | 10 (77) |
| EnSite Precision mapping system | | 3 (23) |
| Substrate | |  |
| Arrhythmogenic cardiomyopathy | | 5 (39) |
| Dilated cardiomyopathy | | 2 (15) |
| Healed myocarditis | | 3 (23) |
| Pathogenic genetic variant* | | 8 (62) |
| Treatment prior to ablation procedure | |  |
| Sotalol | | 7 (77) |
| Beta blocker | | 2 (15) |
| Beta blocker combined with anti-arrhythmic drugs | | 3 (23) |
| Implantable cardioverter defibrillator | | 10 (77) |
| Prior failed endocardial ablation | | 6 (46) |

**2.2 Supplementary Table 2. Detailed Population**

|  | 01 | | 02 | | | 03 | | | 04 | | | 05 | | | 06 | | | 07 | | | 08 | | | 09 | | | 10 | | | 11 | | | 12 | | | 13 | |
| --- | --- | --- | --- | --- | --- | --- | --- | --- | --- | --- | --- | --- | --- | --- | --- | --- | --- | --- | --- | --- | --- | --- | --- | --- | --- | --- | --- | --- | --- | --- | --- | --- | --- | --- | --- | --- | --- |
| *Demographics* | | | |  | | |  | | | |  | | |  | | |  | | |  | | |  | | |  | | |  | | |  | | |  | | |
| Age | | 18 | | | 63 | | | 60 | | 21 | | | 61 | | | 59 | | | 65 | | | 48 | | | 68 | | | 16 | | | 56 | | | 28 | | | 61 |
| Gender | | M | | | F | | | M | | M | | | M | | | M | | | F | | | M | | | M | | | M | | | M | | | M | | | F |
| Pathogenic Variant | | *PKP2* | | | *-* | | | *DSG2* | | *** | | | *PKP2* | | | *PKP2* | | | *PLN* | | | *** | | | *-* | | | *** | | | *DSG2* | | | *PKP2* | | | *PLN* |
| *ECG* | |  | | |  | | |  | |  | | |  | | |  | | |  | | |  | | |  | | |  | | |  | | |  | | |  |
| Rhythm | | SR | | | SR | | | SR | | SR | | | SR | | | SR | | | SR | | | Atrial pacing | | | SR | | | SR | | | Atrial pacing | | | SR | | | Atrial Pacing |
| PR interval | | 160 | | | 218 | | | 172 | | 144 | | | 126 | | | 180 | | | 238 | | | 186 | | | 154 | | | 136 | | | 204 | | | 124 | | | 298 |
| QRS duration | | 92 | | | 88 | | | 104 | | 90 | | | 114 | | | 104 | | | 142 | | | 108 | | | 74 | | | 154 | | | 118 | | | 110 | | | 162 |
| QTc interval | | 407 | | | 444 | | | 434 | | 380 | | | 464 | | | 461 | | | 479 | | | 504 | | | 400 | | | 450 | | | 404 | | | 436 | | | 492 |
| *Imaging* | |  | | |  | | |  | |  | | |  | | |  | | |  | | |  | | |  | | |  | | |  | | |  | | |  |
| LVEF | | 57 | | | 41 | | | 55 | | 57 | | | 65 | | | 51 | | | 31 | | | 48 | | | 53 | | | 61 | | | 40 | | | 59 | | | 40 |
| LGE | | RV | | | - | | | * | | - | | | * | | | RV | | | LV | | | - | | | RV & LV | | | LV | | | LV | | | - | | | * |
| *Phenotype* | | | | |  | | |  | |  | | |  | | |  | | |  | | |  | | |  | | |  | | |  | | |  | | |  |
| Ventricular Arrhythmias | | VT | | | PVC | | | VT | | PVC | | | VT | | | VT storm | | | VT | | | PVC | | | VT | | | VT & VF | | | VT | | | VT | | | VT |
| Diagnosis | | ACM | | | Symp.  PVC | | | ACM | | Symp.  PVC | | | ACM | | | ACM | | | DCM | | | Symp.  PVC | | | Myocarditis ** | | | Myocarditis ** | | | Myocarditis ** | | | ACM | | | DCM |
| *Treatment* | | | | |  | | |  | |  | | |  | | |  | | |  | | |  | | |  | | |  | | |  | | |  | | |  |
| AAD treatment | | Sotalol | | | Sotalol | | | Sotalol | | - | | | Sotalol | | | BB + Disopyramide | | | BB + Amiodaron | | | Sotalol | | | Sotalol | | | BB | | | Sotalol | | | BB | | | BB + Amiodaron |
| Prior endocardial ablation | | - | | | + | | | + | | + | | | - | | | - | | | - | | | + | | | + | | | - | | | - | | | - | | | + |
| Device | | ICD | | | - | | | ICD | | - | | | ICD | | | ICD | | | ICD | | | PM | | | ICD | | | ICD | | | ICD | | | ICD | | | ICD |

**Abbreviations Supplementary Table 2:**

AAD = anti-arrhythmic drugs; ACM = arrhythmogenic cardiomyopathy; BB = betablocker; DCM = dilated cardiomyopathy; DSG2 = desmoglein-2; ICD: implantable cardioverter defibrillator; LGE = late gadolinium enhancement ; LV = left ventricle; LVEF = left ventricular ejection fraction; *PKP2* = plakophillin-2; *PLN* = phospholamban; PM = pacemaker; PVC = premature ventricular complex; RV = right ventricle; SR = sinus rhythm; VT = ventricular tachycardia; * = not tested, ** = healed myocarditis based on cardiac magnetic resonance imaging findings.

**2.3 Supplementary Table 3.** **Ranges of earliest and latest activation per subject**

|  |  | *i*ECG | | |  |  | | EAM | | | |
| --- | --- | --- | --- | --- | --- | --- | --- | --- | --- | --- | --- |
| ID | Total Activation Timing  Mapped (ms) | | Earliest Activation  Mapped Surfaces (ms) | Latest Activation  Mapped Surfaces (ms) | | | Total Activation Timing Mapped (ms) | | Earliest activation  Mapped Surfaces (ms) | Latest activation  Mapped Surfaces (ms) |  |
| 1 | 91.6 ± 3.0 | | -32.0 ± 1.6 | 58.5 ± 3.5 | | | 74 | | -17 | 57 |  |
| 2 | 93.3 ± 8.4 | | -33.5 ± 0.4 | 50.5 ± 0.6 | | | 90 | | -22 | 68 |  |
| 3 | 111.0 ± 4.7 | | -30.9 ± 1.9 | 73.0 ± 4.3 | | | 172 | | -22 | 150 |  |
| 4 | 90.9 ± 5.4 | | -35.6 ± 1.7 | 50.8 ± 5.2 | | | 83 | | -45 | 38 |  |
| 5 | 103.0 ± 1.3 | | -64.6 ± 0.8 | 38.1 ± 1.2 | | | 124 | | -85 | 39 |  |
| 6 | 101.0 ± 4.1 | | -37.1 ± 1.3 | 57.9 ± 2.9 | | | 94 | | -20 | 74 |  |
| 7 | 147.0 ± 11.6 | | -50.4 ± 2.8 | 92.2 ± 8.7 | | | 201 | | -88 | 113 |  |
| 8 | 108.0 ± 2.2 | | -31.6 ± 8.1 | 72.7 ± 4.3 | | | 104 | | -36 | 68 |  |
| 9 | 82.0 ± 4.5 | | -32.2 ± 1.2 | 42.5 ± 1.5 | | | 147 | | -45 | 102 |  |
| 10 | 156.0 ± 5.7 | | -25.3 ± 2.1 | 118.0 ± 6.0 | | | 159 | | -67 | 92 |  |
| 11 | 103.0 ± 5.5 | | -47.1 ± 3.1 | 50.5 ± 1.5 | | | 110 | | -47 | 63 |  |
| 12 | 107.0 ± 2.8 | | -34.8 ± 1.3 | 70.4 ± 2.4 | | | 104 | | 0 | 104 |  |
| 13 | 145.0 ± 0.9 | | -71.6 ± 0.9 | 73.0 ± 1.0 | | | 152 | | -83 | 69 |  |
| Total | 111.0 ± 23.4 | | -40.5 ± 13.8 | 65.1 ± 21.5 | | | 124 ± 39 | | -44 ± 29 | 80 ± 32 |  |

Legend Supplementary Table 3: ranges in total activation duration of both the iECG LAT maps and the invasive LAT maps over all invasively mapped surfaces per subject. The timing of the earliest activated node and the latest activated node were stated. Abbreviations: *i*ECG = inverse electrocardiography; EAM = electro-anatomical mapping.

**3.1 Supplementary Figure 1. All *i*ECG, EAM maps and voltage maps**

Legend: Epicardial and endocardial local activation timing (LAT) maps and voltage maps derived from iECG estimation and invasive electroanatomical mapping for each patient. Areas of earliest activation are depicted with red and areas with latest activation are depicted in blue. Grey areas in the invasive maps represent areas without annotated electrograms. Abnormal voltage EGMs were defined as bipolar voltage amplitude <0.5 mV for both the epicardial and endocardial surface. Areas with the lowest voltage EGMs are depicted with red and areas with >1.5 mV voltage EGMs are depicted with purple. See Supplementary Table 3 for LAT ranges. Imaging views are based on the anatomical approach of Cosio et al (1). Abbreviations: iECG: inverse electrocardiography; LAO = left anterior oblique; RAO = right anterior oblique.

Per slide, a representative example per subject of all invasively mapped surfaces are displayed in RAO, LAO and Inferior view. Maps are displayed from early (red) to late activation for the iECG estimation (left panel) and invasive map (middle panel). Per surface, time scales are displayed below the maps. Additionally, the invasive voltage map (right panel) is displayed. Correlation coefficient (CC) and absolute difference (AD) of the activation times between the iECG and invasive map are displayed at the top of each slide per presented surface.

**3.2 Supplementary Figure 2. Factors associated with Correlation Coefficients and Absolute Differences.**

Legend: EGM = electrogram; LV = left ventricle; RV = right ventricle.

Both QRS duration or the surface did not significantly affect the correlation coefficient or absolute differences (upper row). There was a trend between the number of annotations and abnormal voltage EGMs of the invasive map and the correlation coefficient, low density and percentages were associated with lower CC.


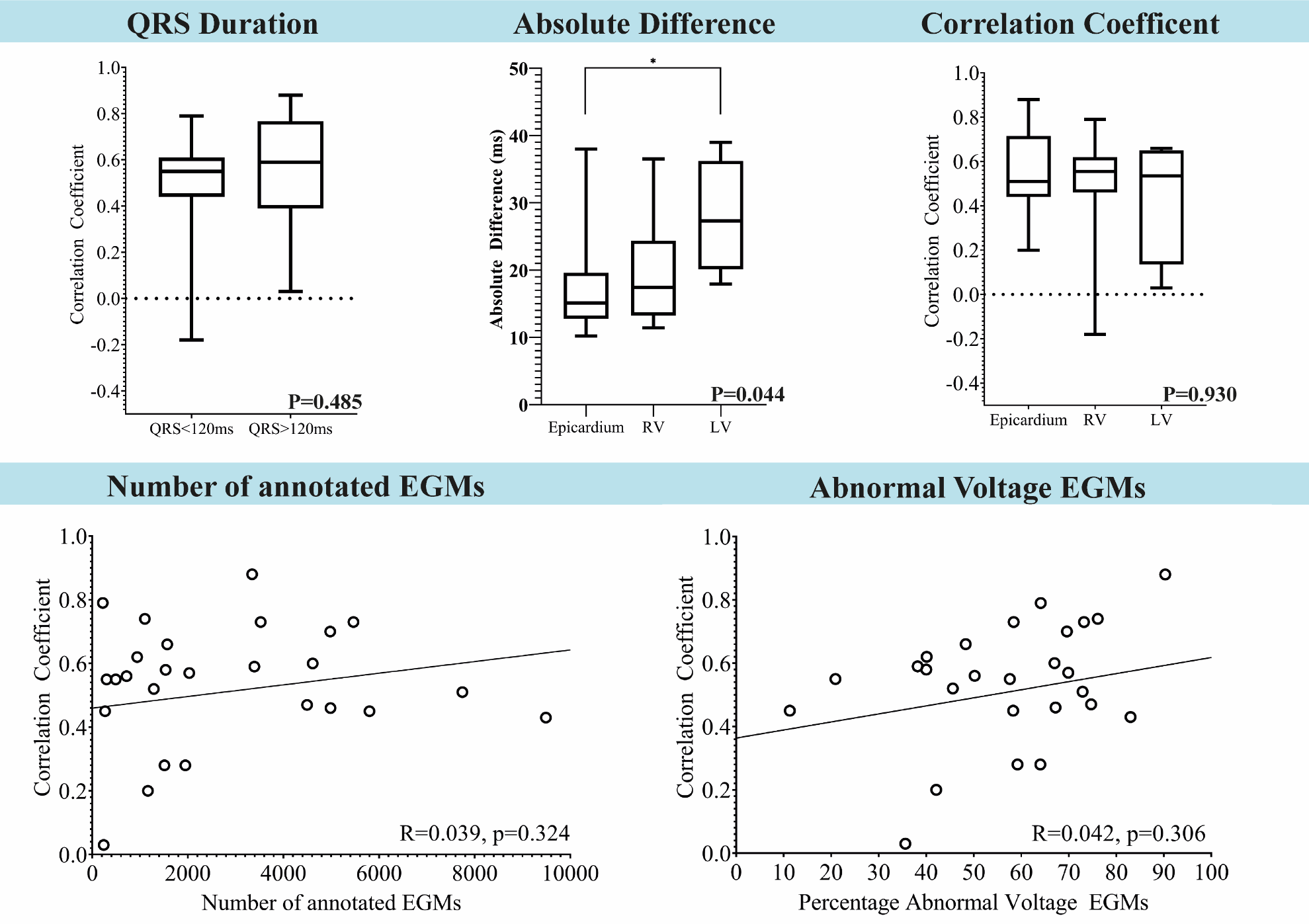


**3.3 Supplementary Figure 3. Scatter plots and Bland-Altman plots per surface and for each patient**

Legend: Scatter plots for local activation timing (LAT) between inverse electrocardiography (iECG) and electroanatomical mapping (EAM), stratified for the epicardium, right ventricular (RV) endocardium and left ventricular (LV) endocardium. Bland-Altman plots for LAT derived from iECG and EAM (2).

In this supplement, the beat to beat variation in noninvasive iECG local activation time (LAT) estimation and invasive LAT is shown per invasively mapped surface. LATs were paired based on location on the surface on the CT-based model, as invasive LATs were registered to the CT-based model. For all simulated beats (beat 1-5), a scatter plot of invasive LAT to non-invasive LAT is shown with corresponding regression line in the left panel of each slide. Corresponding correlation coefficient (CC) and P-values of the regression analysis are displayed in the bottom of each slide. In the right panel, a Bland-Altman plot is displayed to show the agreement between the two methods (e.g. invasive versus non-invasive LAT). In the plot, the X-axis the average of paired LATs and the Y axis represents the differences between two paired LATs.

**Reference**

1. Cosio FG, Anderson RH, Kuck KH, Becker A, Borggrefe M, Campbell RW, et al. Living anatomy of the atrioventricular junctions. A guide to electrophysiologic mapping. A Consensus Statement from the Cardiac Nomenclature Study Group, Working Group of Arrhythmias, European Society of Cardiology, and the Task Force on Cardiac Nomenclature from NASPE. Circulation. 1999;100(5):e31-7.
2. Bland JM, Altman DG. Statistical methods for assessing agreement between two methods of clinical measurement. Lancet. 1986 Feb 8;1(8476):307-10. PMID: 2868172.
