# Supplementary material for: Comparing Non-invasive Inverse Electrocardiography With Invasive Endocardial and Epicardial Electroanatomical Mapping During Sinus Rhythm
Source: Front Physiol. 2021 Oct 4;12:730736. doi: 10.3389/fphys.2021.730736 (PMC8521153; doi:10.3389/fphys.2021.730736)
Supplement: Supplementary Figure 3 — Scatterplot and Bland-Altman plot per included patient. [file Data_Sheet_1.PDF]

Supplementary Figure 3

Scatter plots and Bland-Altman plots per surface and for each patient

Legend: Scatter plots for local activation timing (LAT) between inverse electrocardiography (*i*ECG) and electroanatomical mapping (EAM), stratified for the epicardium, right ventricular (RV) endocardium and left ventricular (LV) endocardium. Bland-Altman plots for LAT derived from *i*ECG and EAM. Abbreviations as in manuscript.

In this supplement, the beat to beat variation in noninvasive *i*ECG local activation time (LAT) estimation and invasive LAT is shown per invasively mapped surface. LATs were paired based on location on the surface on the CT-based model, as invasive LATs were registered to the CT-based model. For all simulated beats (beat 1-5), a scatter plot of invasive LAT to non-invasive LAT is shown with corresponding regression line in the **left panel** of each slide. Corresponding correlation coefficient (CC) and P-values of the regression analysis are displayed in the bottom of each slide. In the **right panel**, a Bland-Altman plot is displayed to show the agreement between the two methods (e.g. invasive versus non-invasive LAT). In the plot, the X-axis the average of paired LATs and the Y axis represents the differences between two paired LATs.

ID: 1. Epicardium

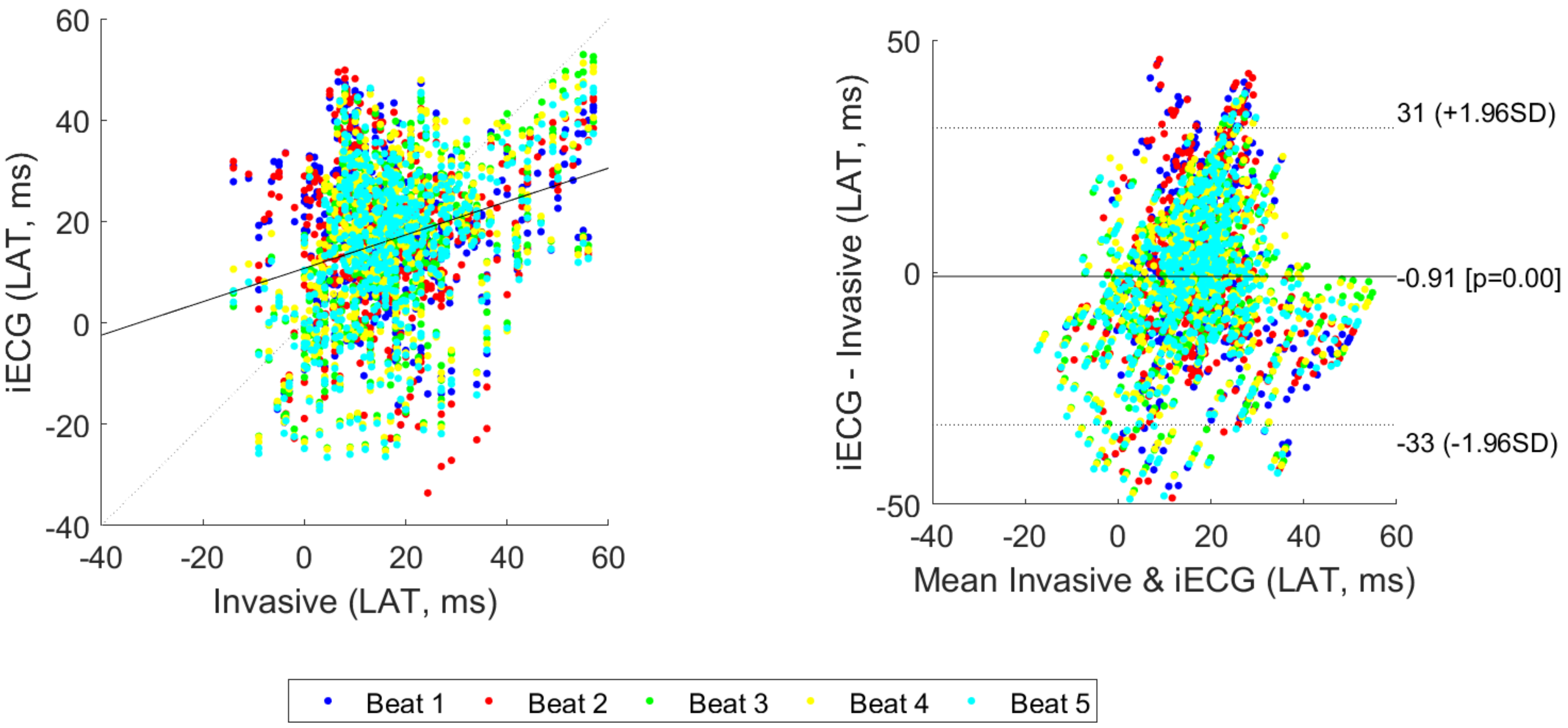

CC = 0.277; P = 0.009

ID: 1. RV Endocardium

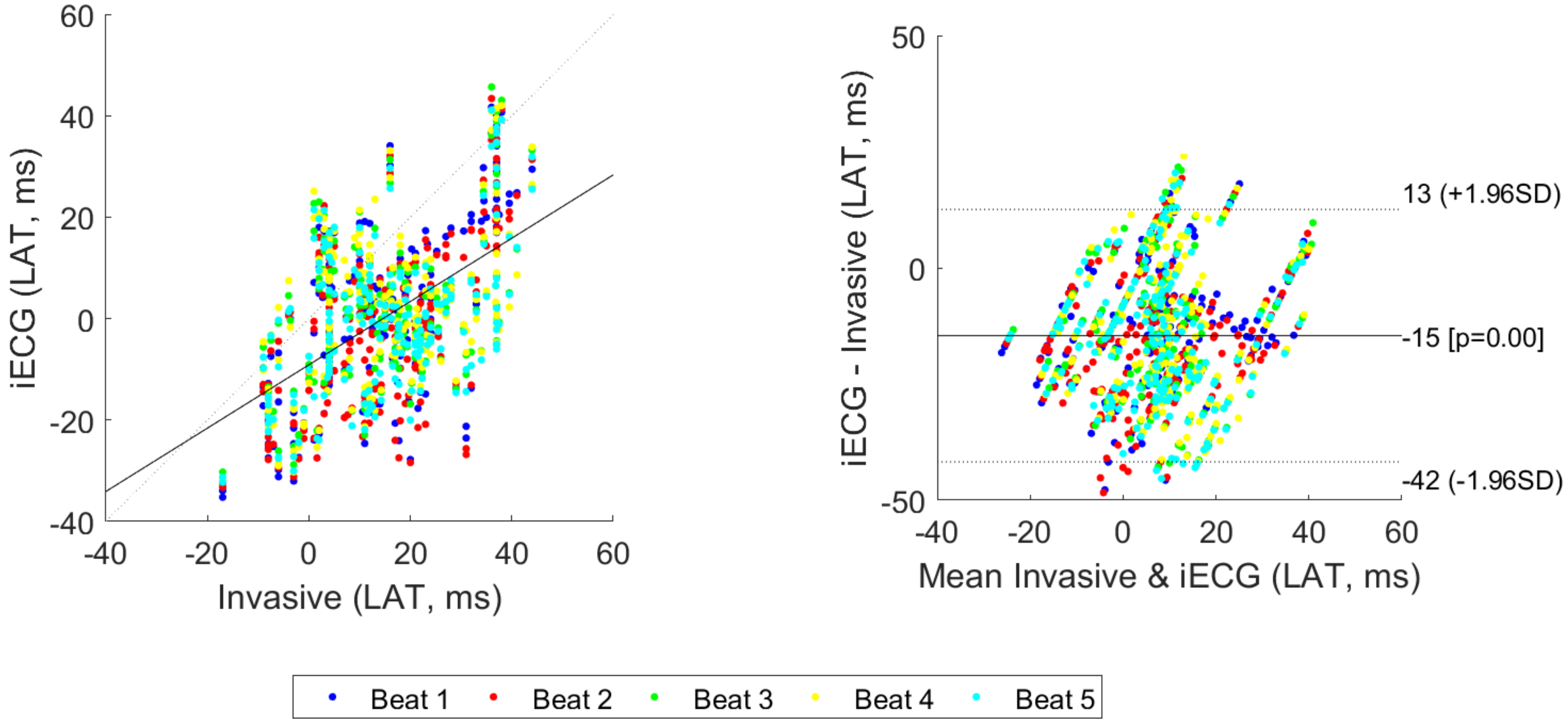

CC = 0.549; P < 0.001

ID: 2. Epicardium

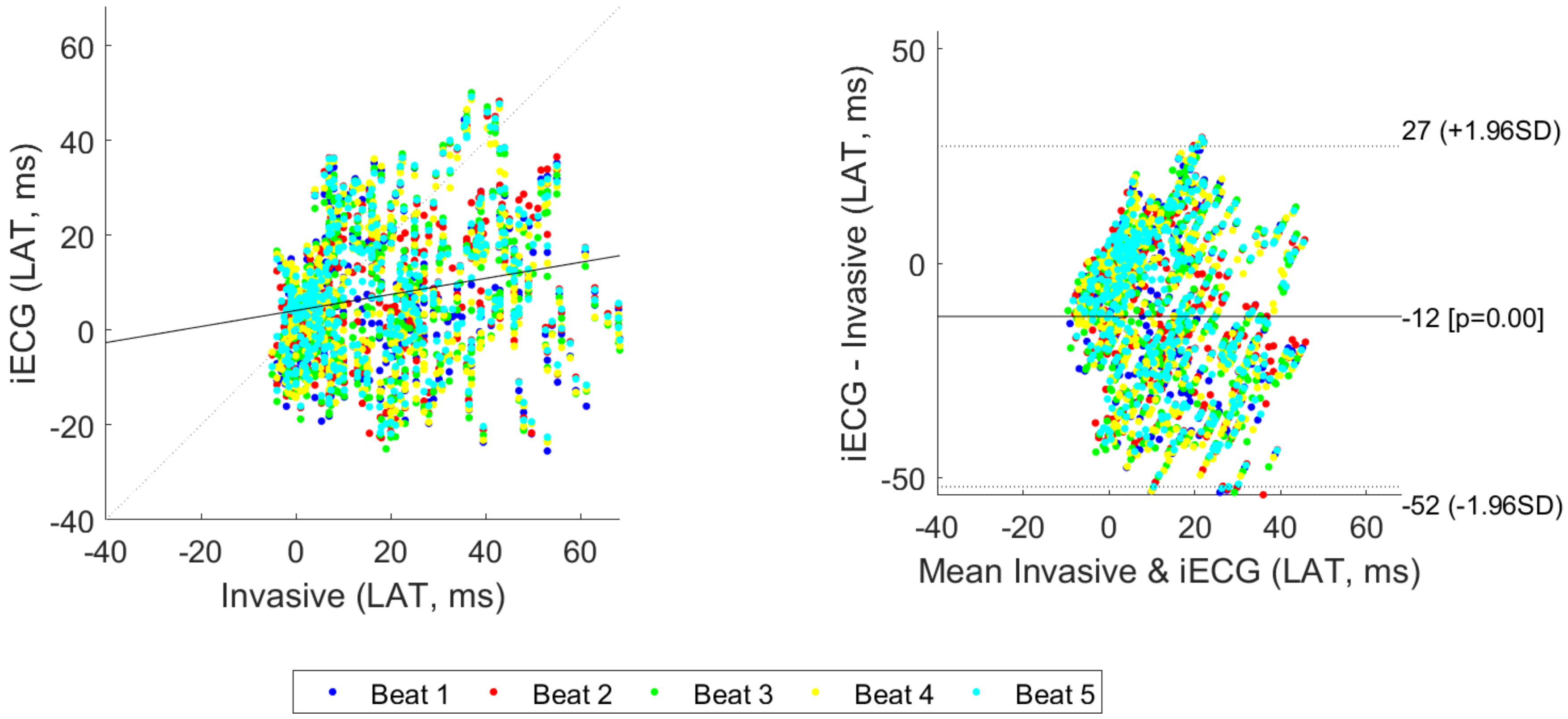

CC = 0.202; P = 0.002

ID: 2. RV Endocardium

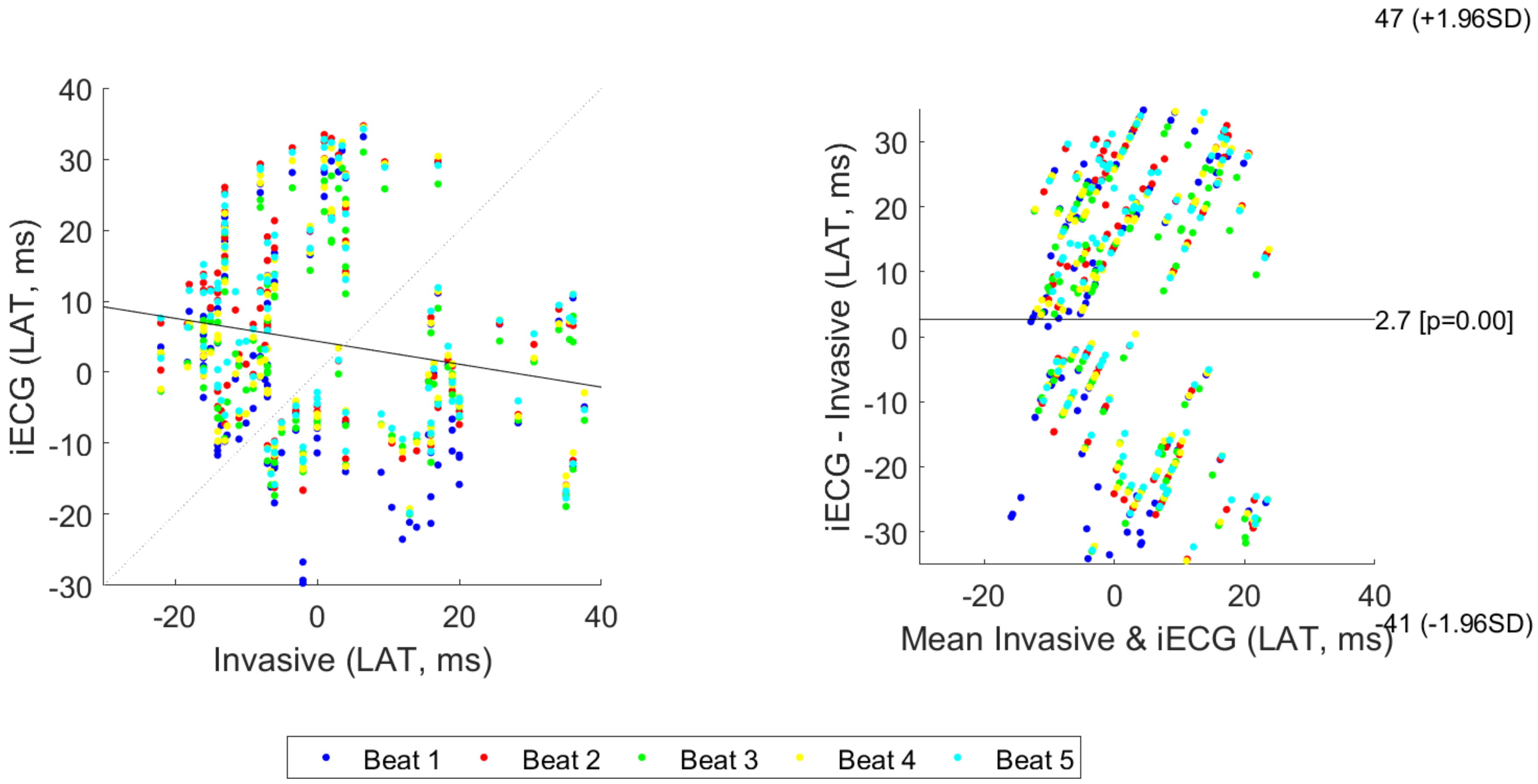

CC = -0.178; P = 0.098

ID: 3. Epicardium

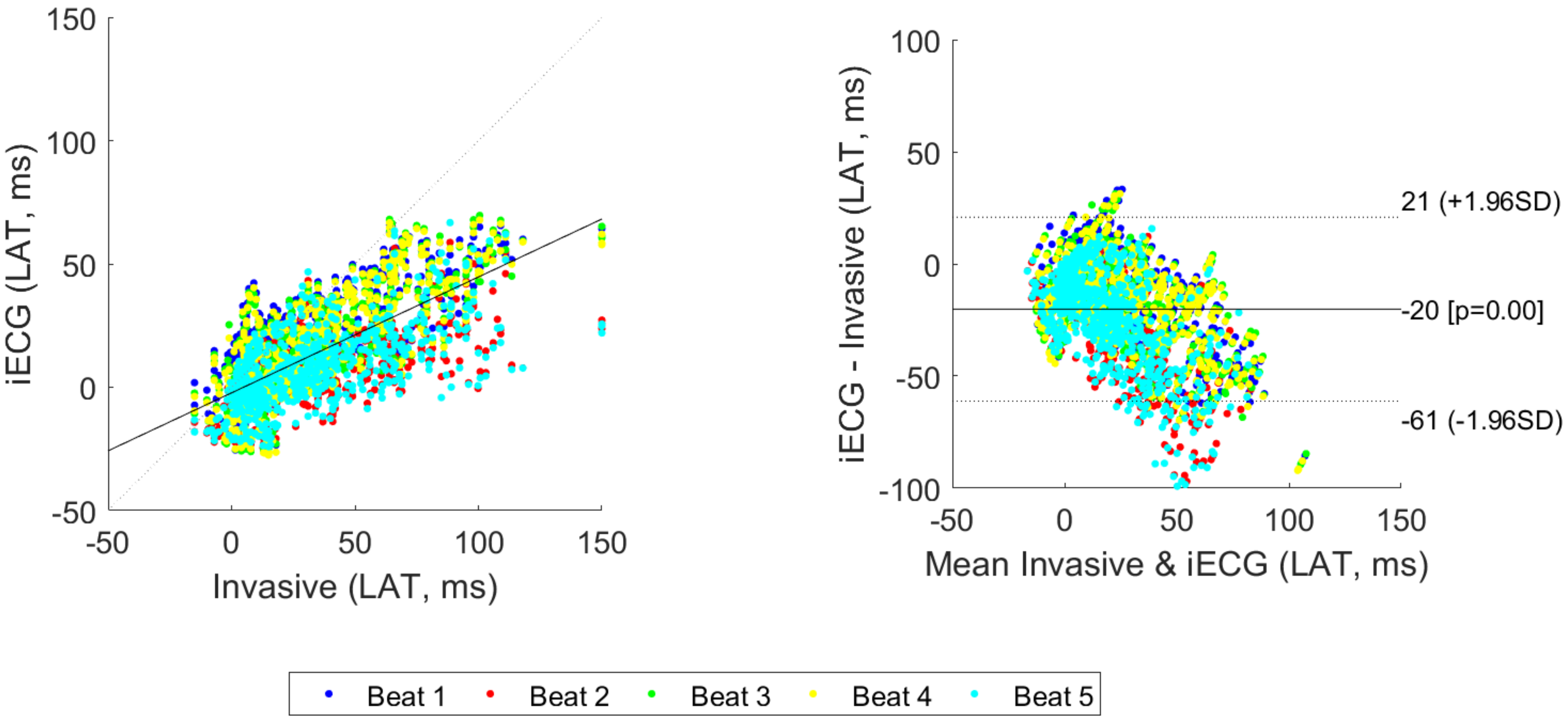

CC = 0.732; P < 0.001

ID: 3. RV Endocardium

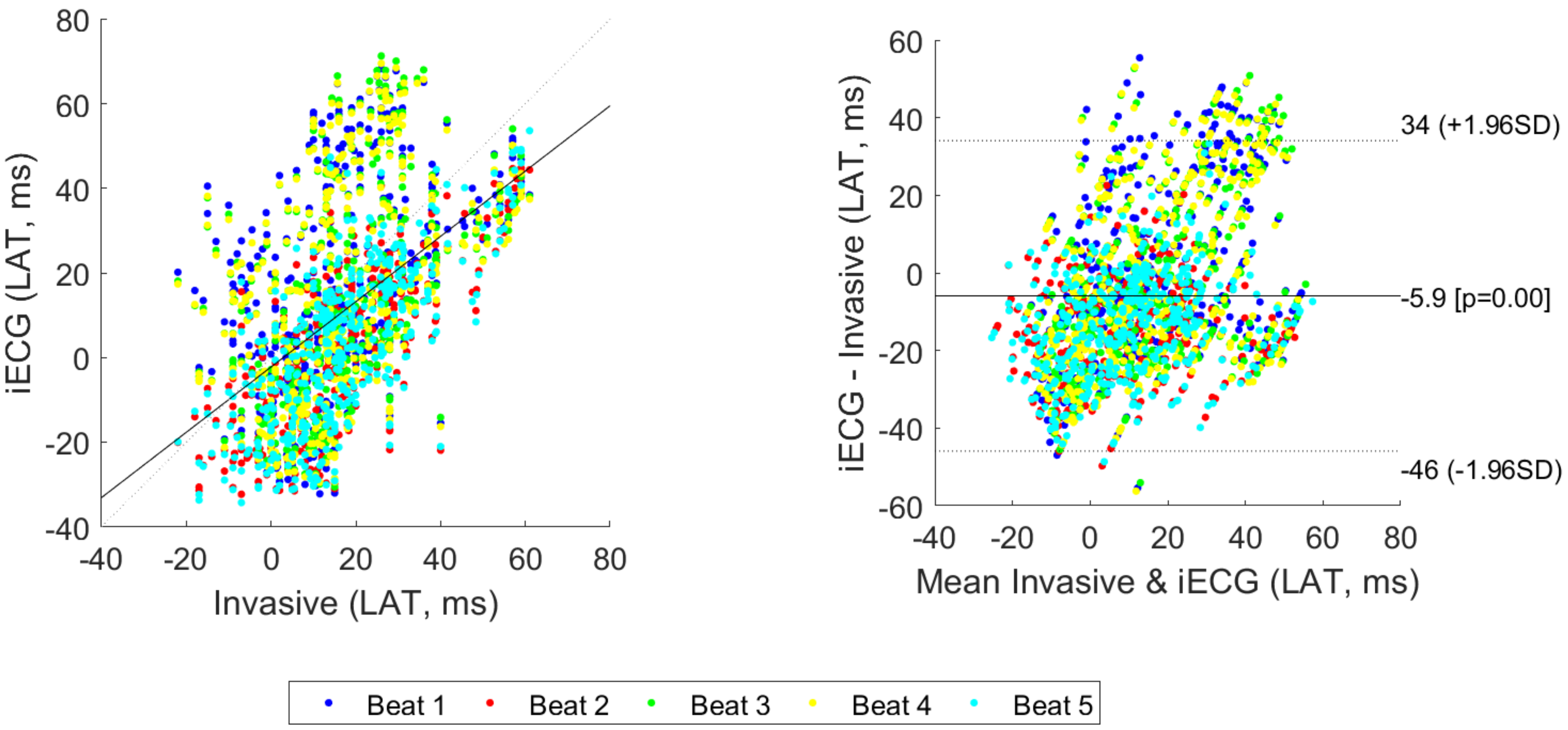

CC = 0.560; P < 0.001

ID: 4. Epicardium

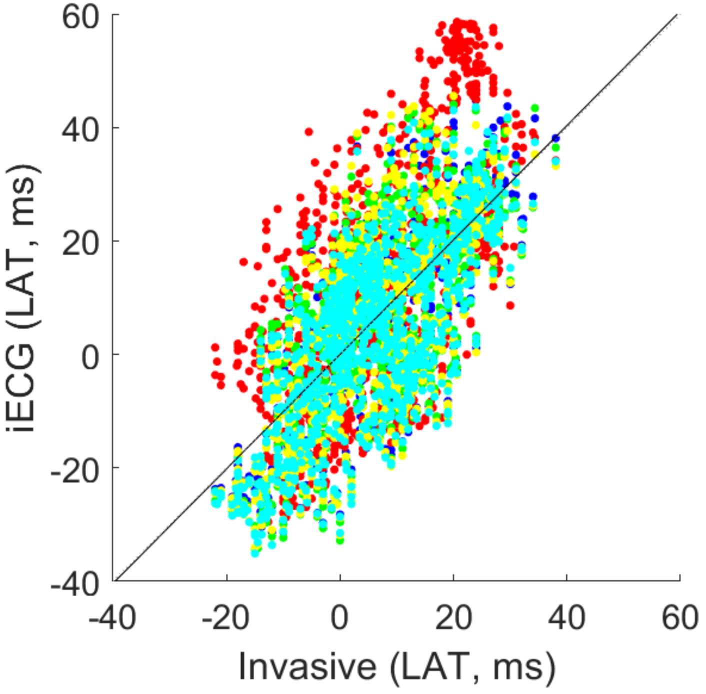

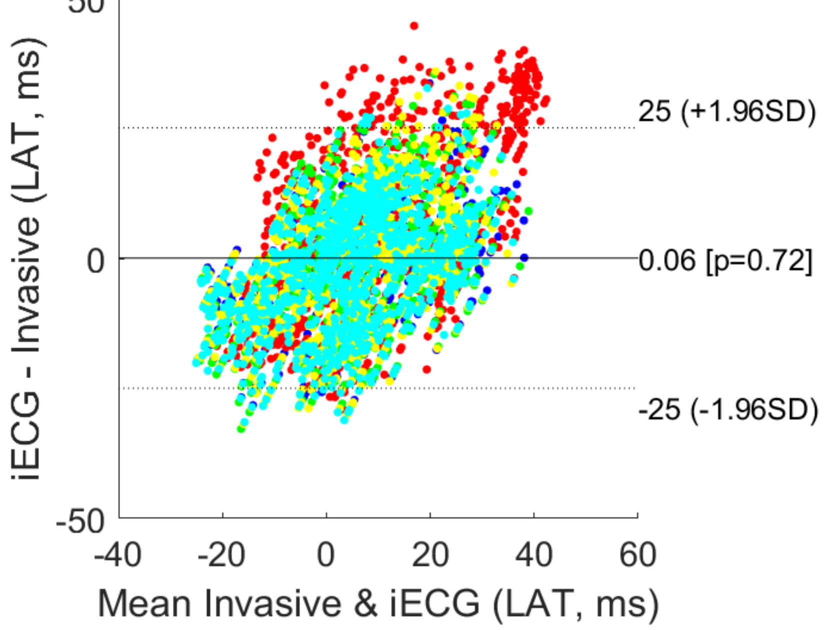

Beat 1 Beat 2 Beat 3 Beat 4 Beat 5

CC = 0.698; P < 0.001

ID: 4. RV Endocardium

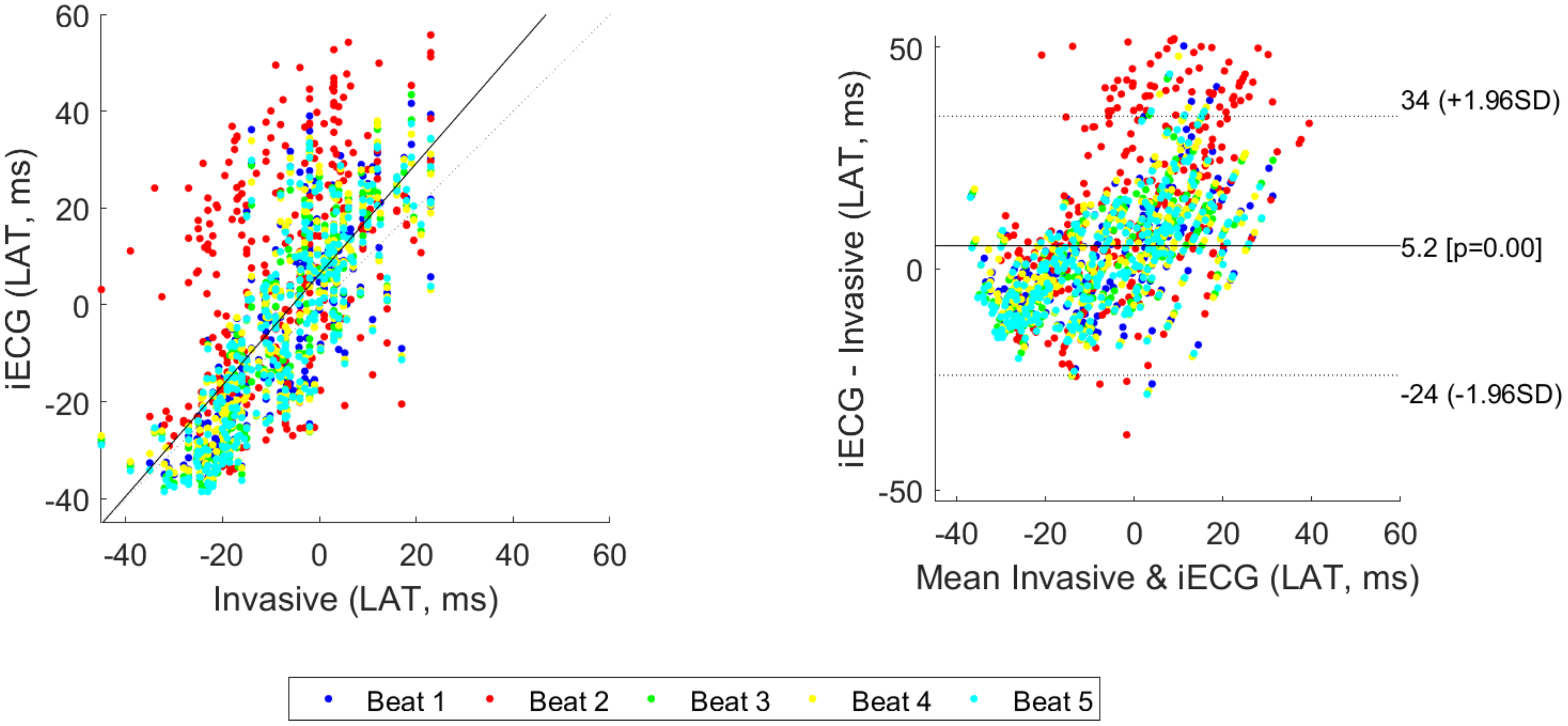

CC = 0.737; P < 0.001

ID: 5. Epicardium

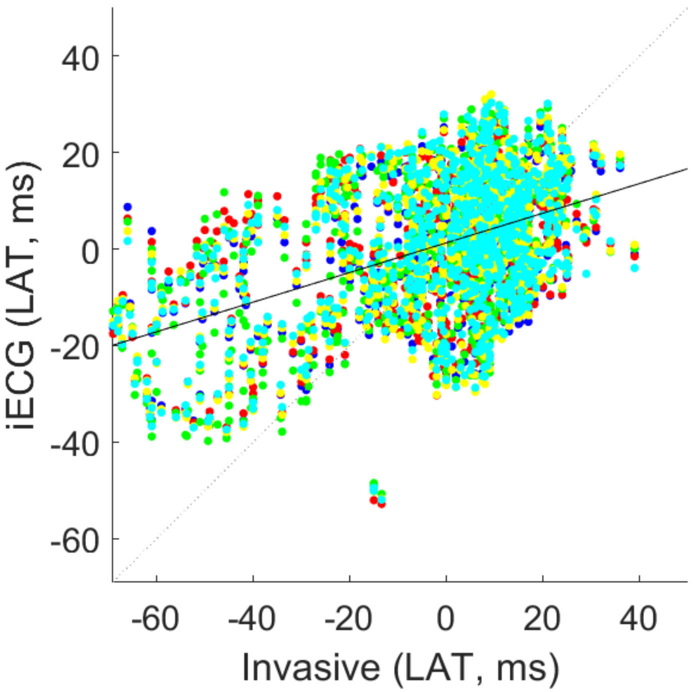

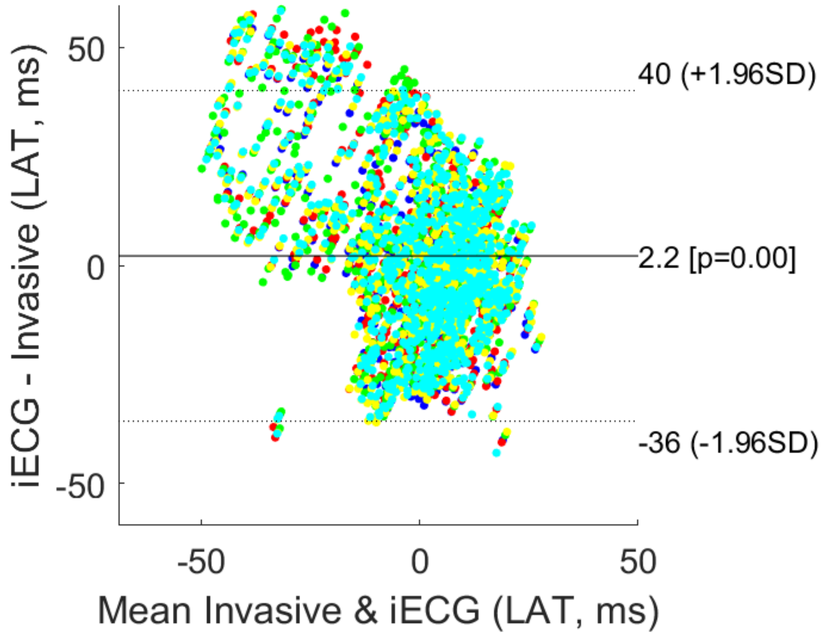

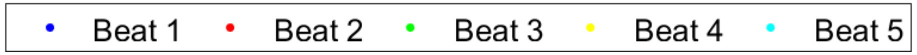

CC = 0.461; P < 0.001

ID: 5. RV Endocardium

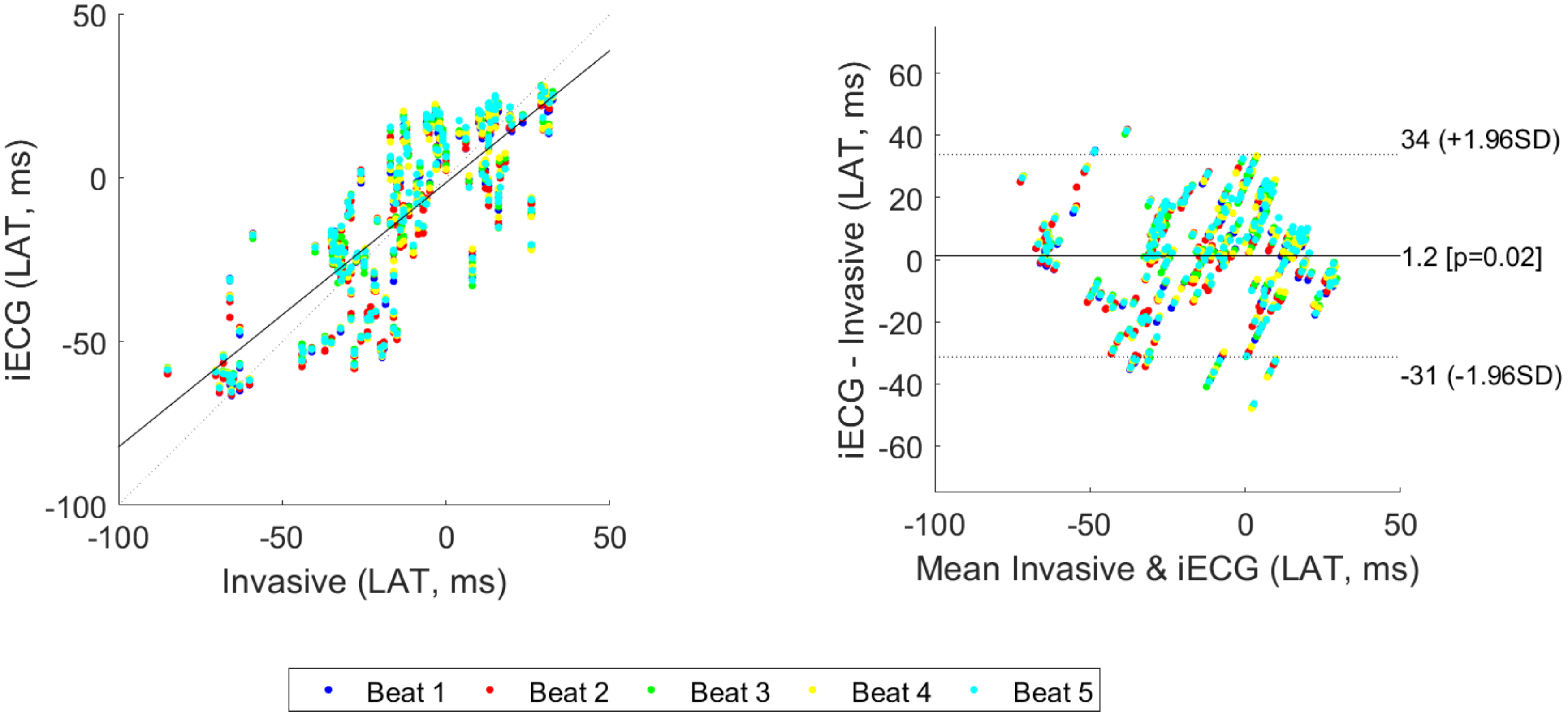

CC = 0.788; P < 0.001

ID: 6. Epicardium

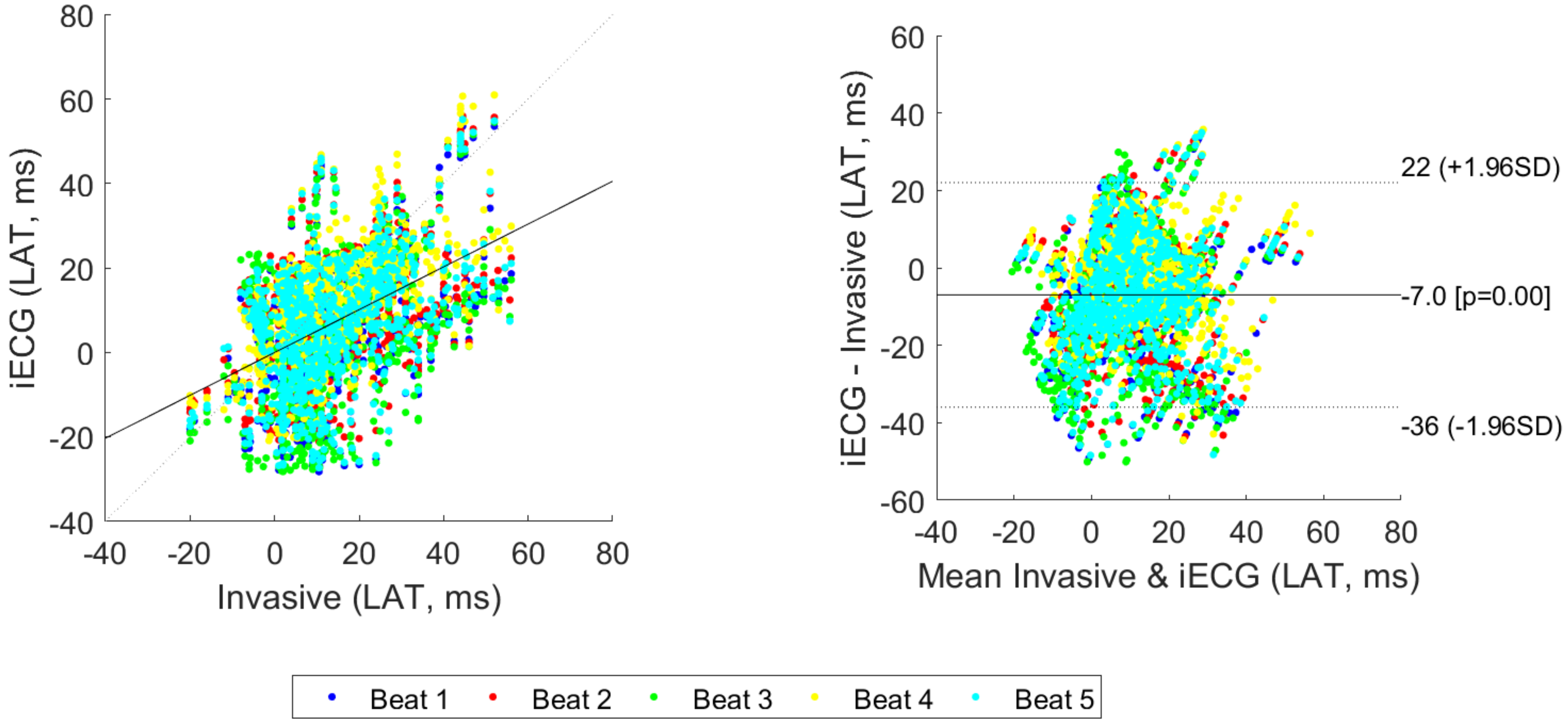

CC = 0.471; P < 0.001

ID: 6. RV Endocardium

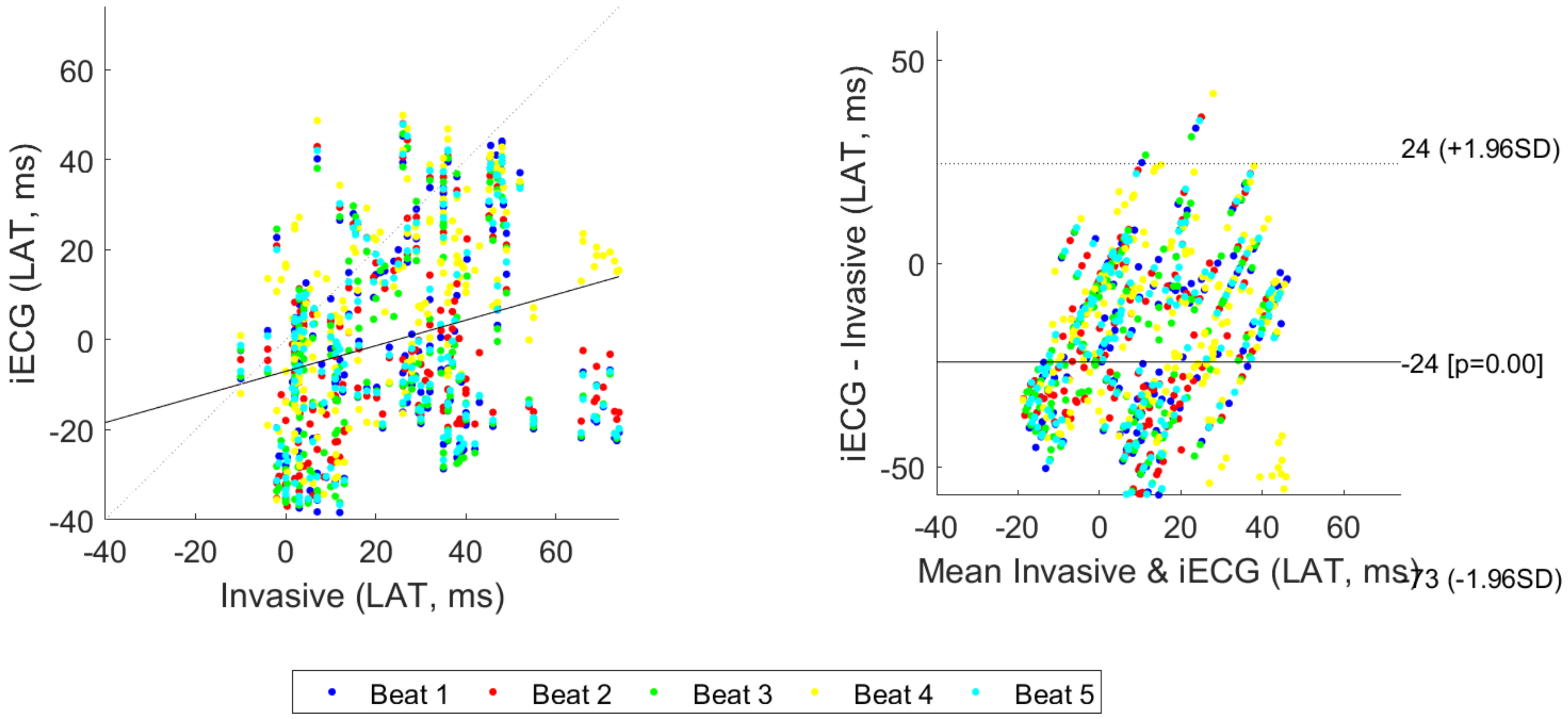

CC = 0.277; P = 0.004

ID: 7. Epicardium

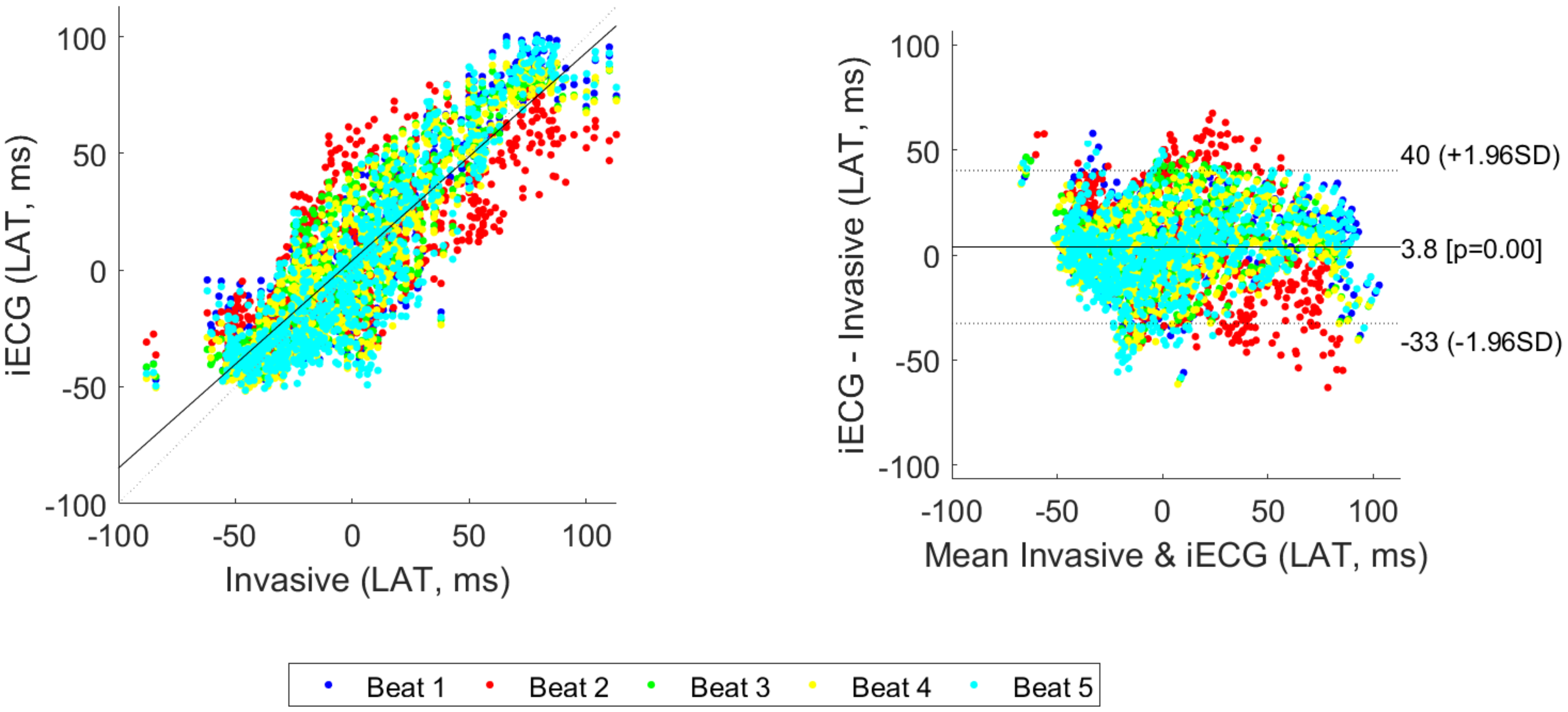

CC = 0.875; P < 0.001

ID: 7. RV Endocardium

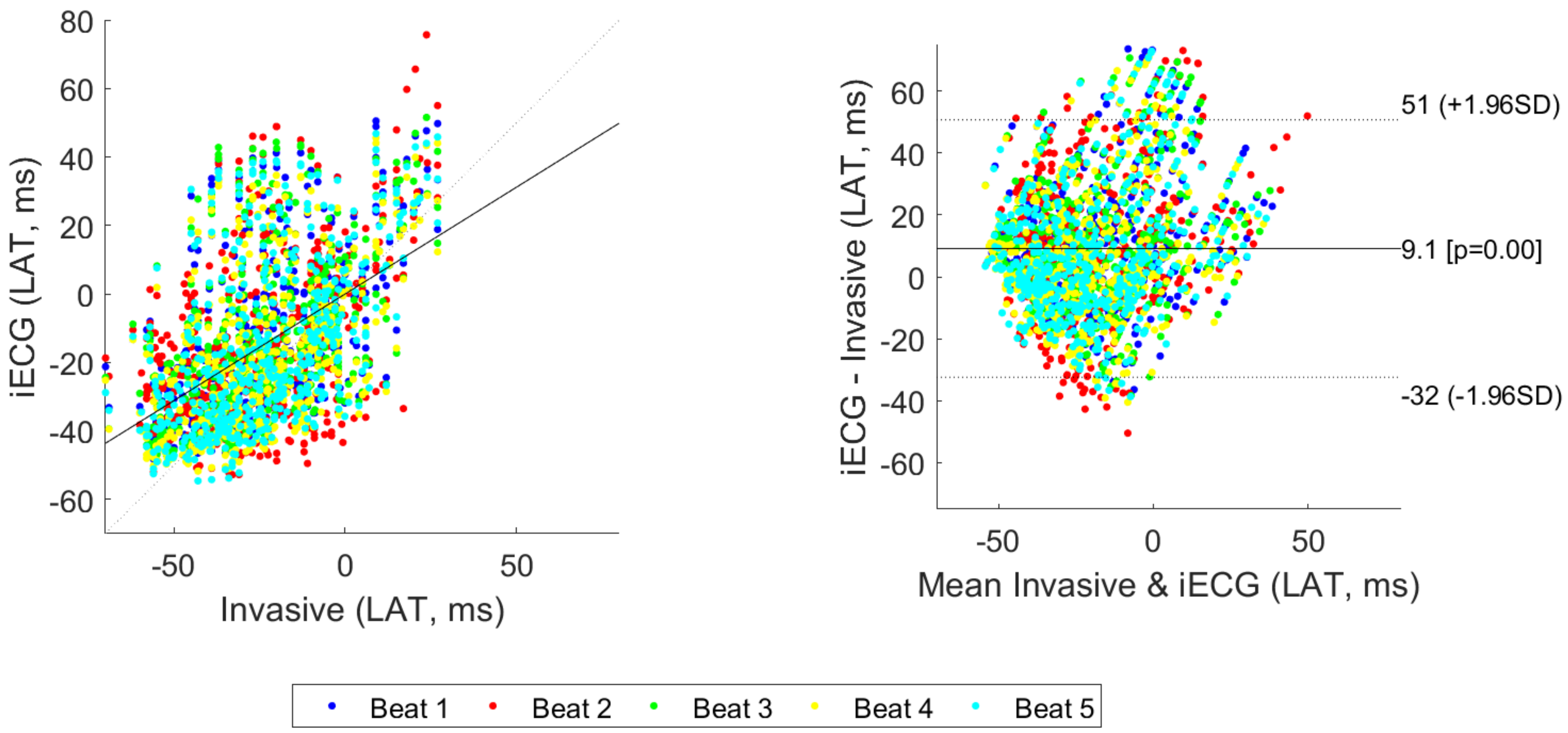

CC = 0.515; P < 0.001

ID: 8. Epicardium

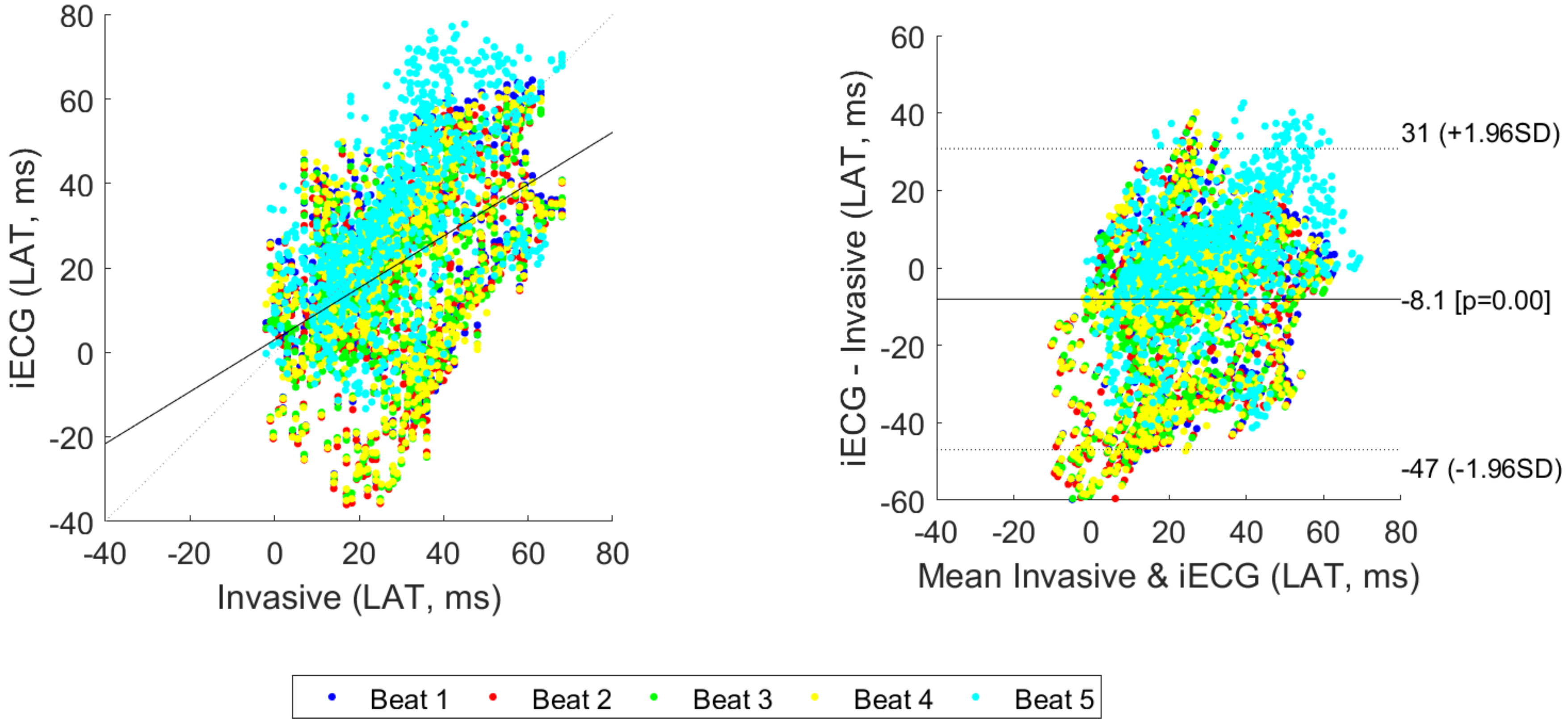

CC = 0.452; P < 0.001

ID: 8. RV Endocardium

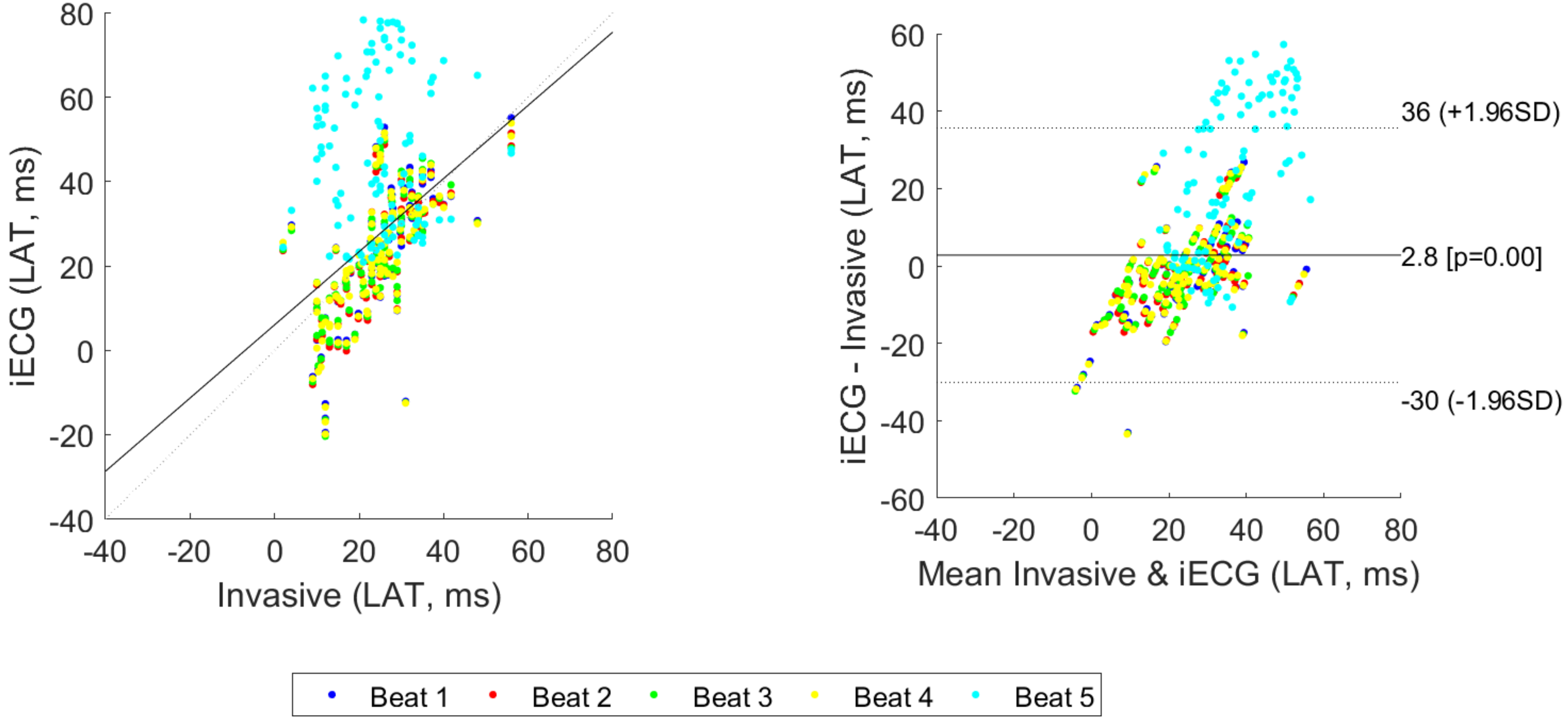

CC = 0.551; P = 0.136

ID: 8. LV Endocardium

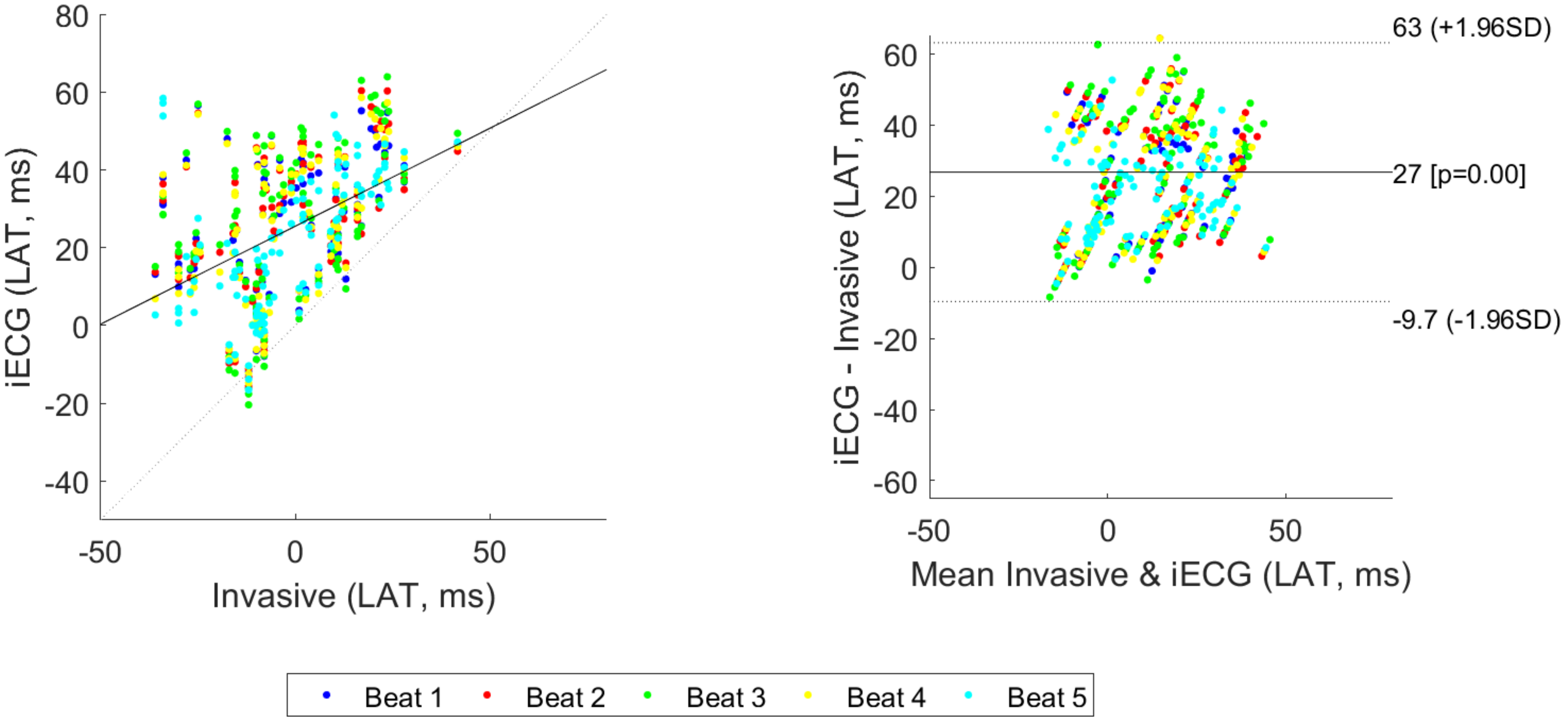

CC = 0.449; P < 0.001

ID: 9.   Epicardium

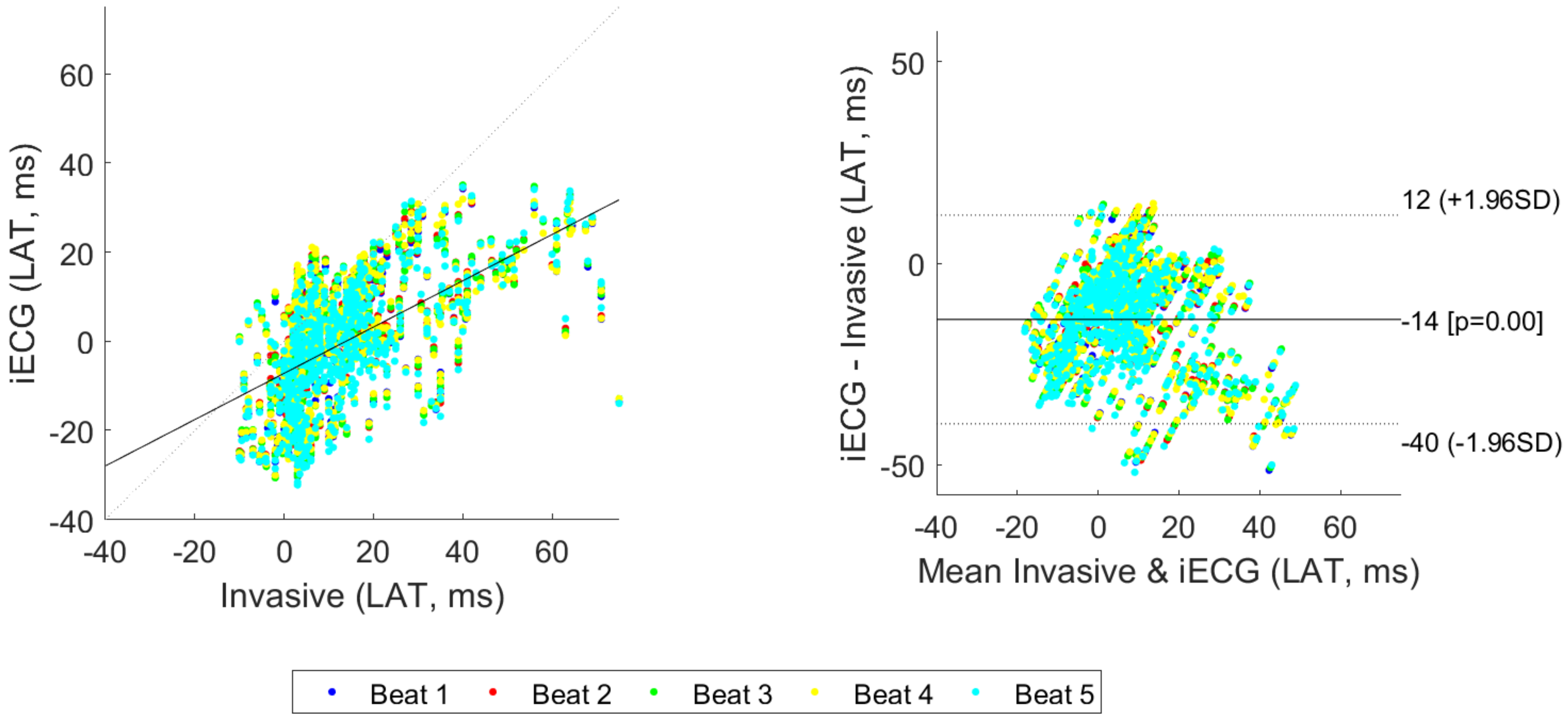

CC = 0.596; P < 0.001

ID: 9. RV Endocardium

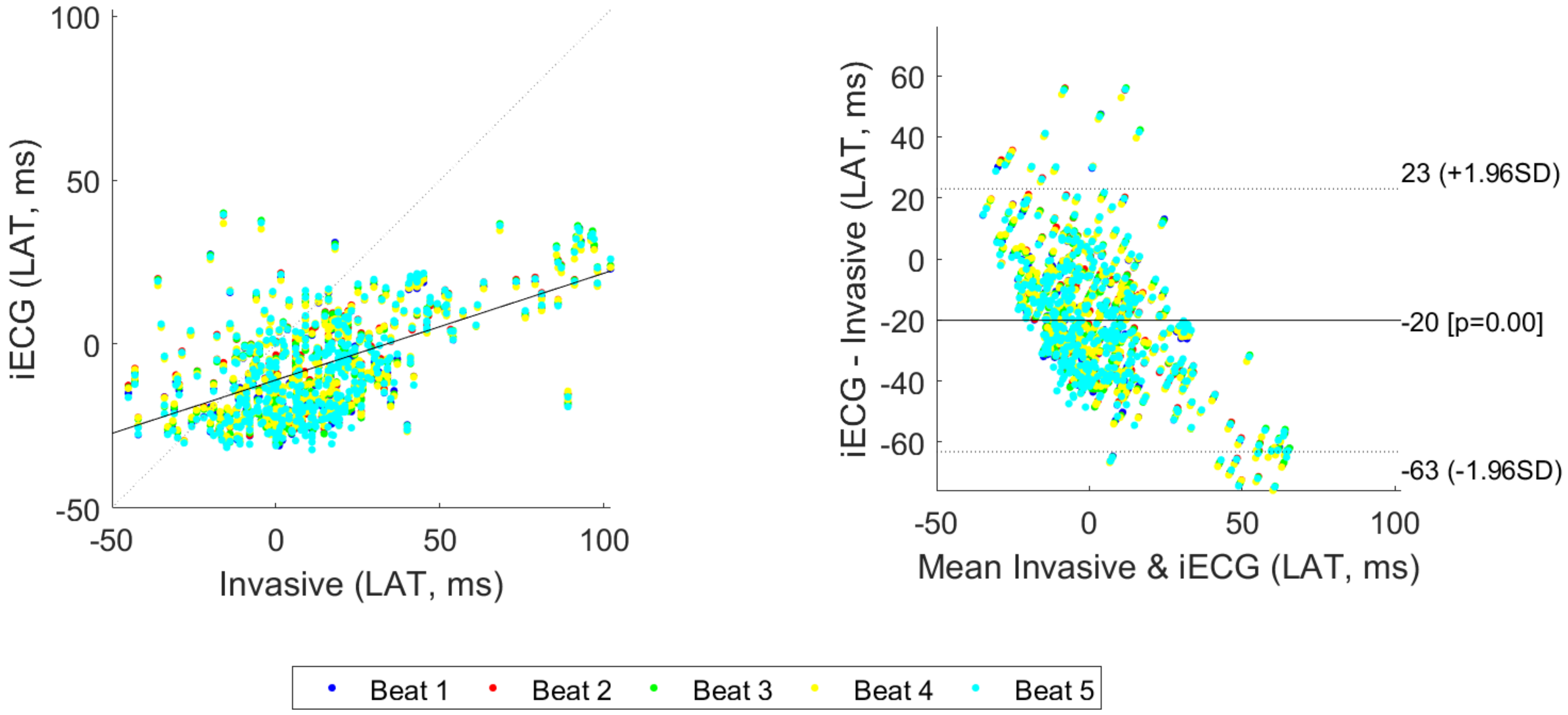

CC = 0.573; P < 0.001

ID: 10. Epicardium

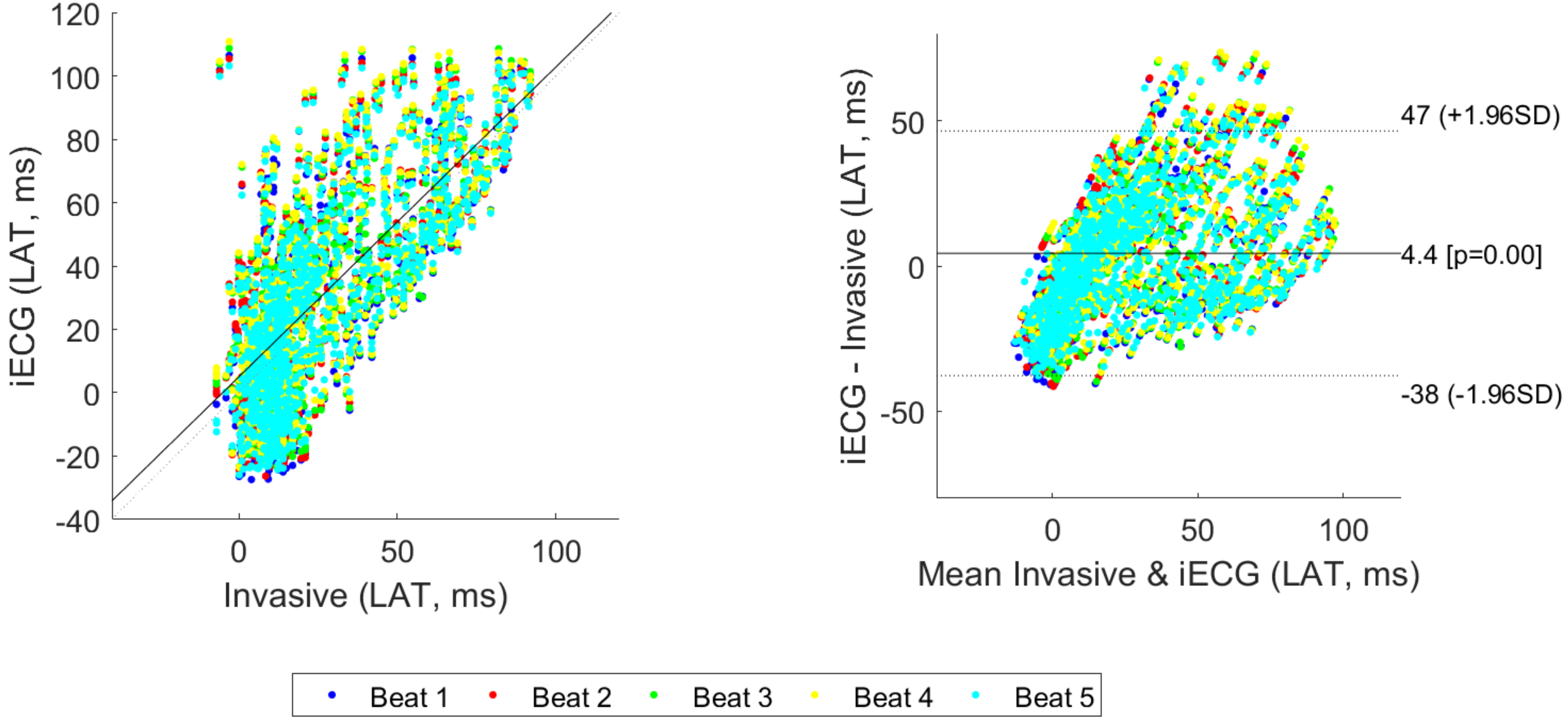

CC = 0.727; P < 0.001

ID: 10. LV Endocardium

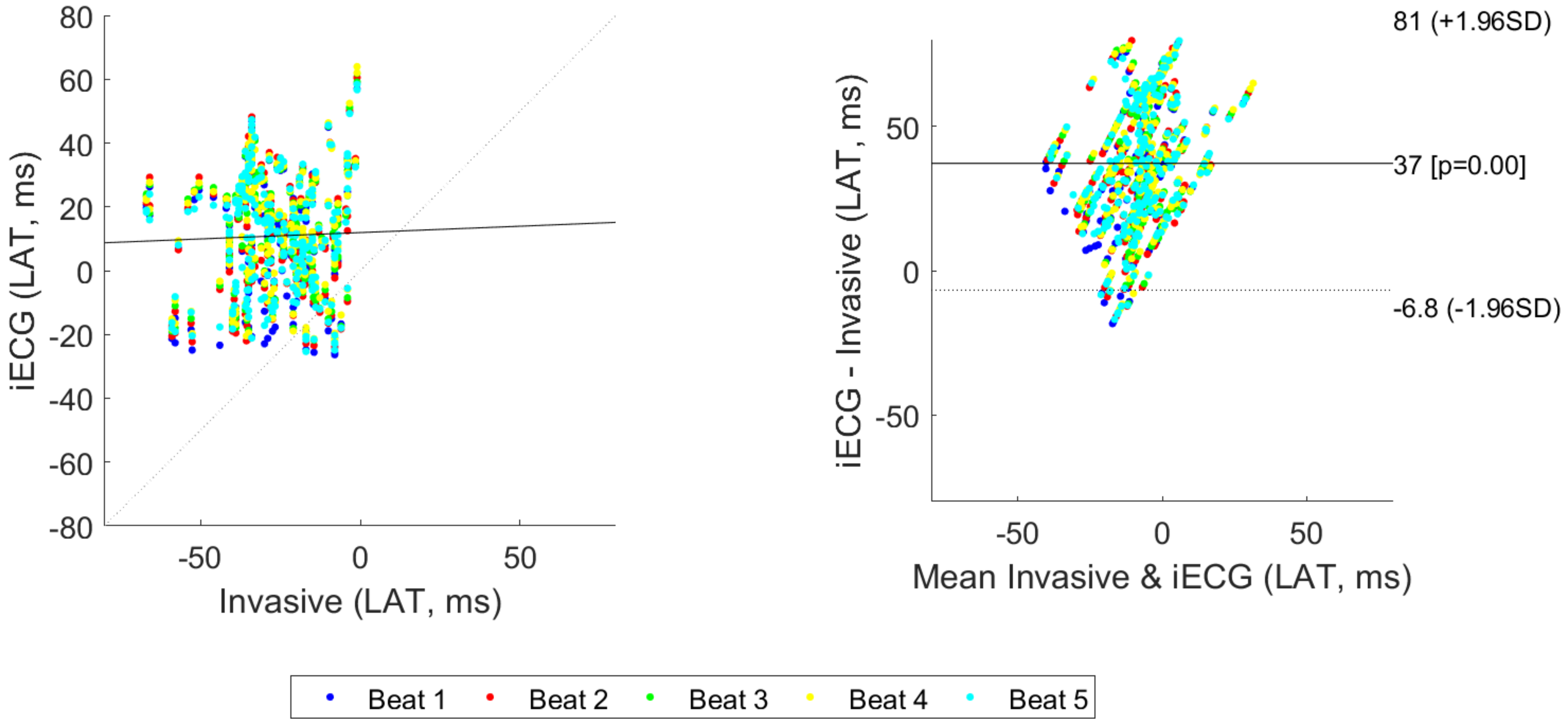

CC = 0.030; P = 0.658

ID: 11. Epicardium

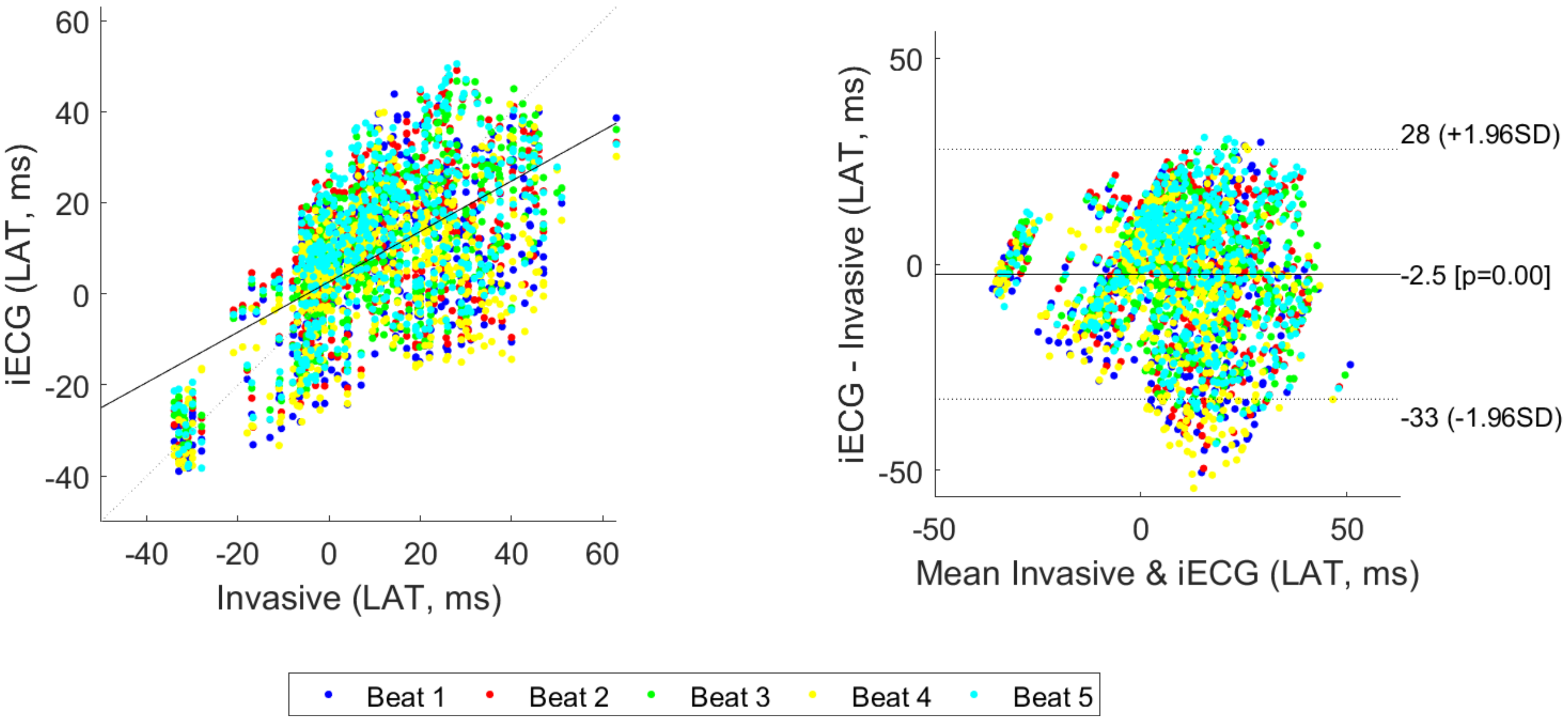

CC = 0.588; P < 0.001

ID: 11. LV Endocardium

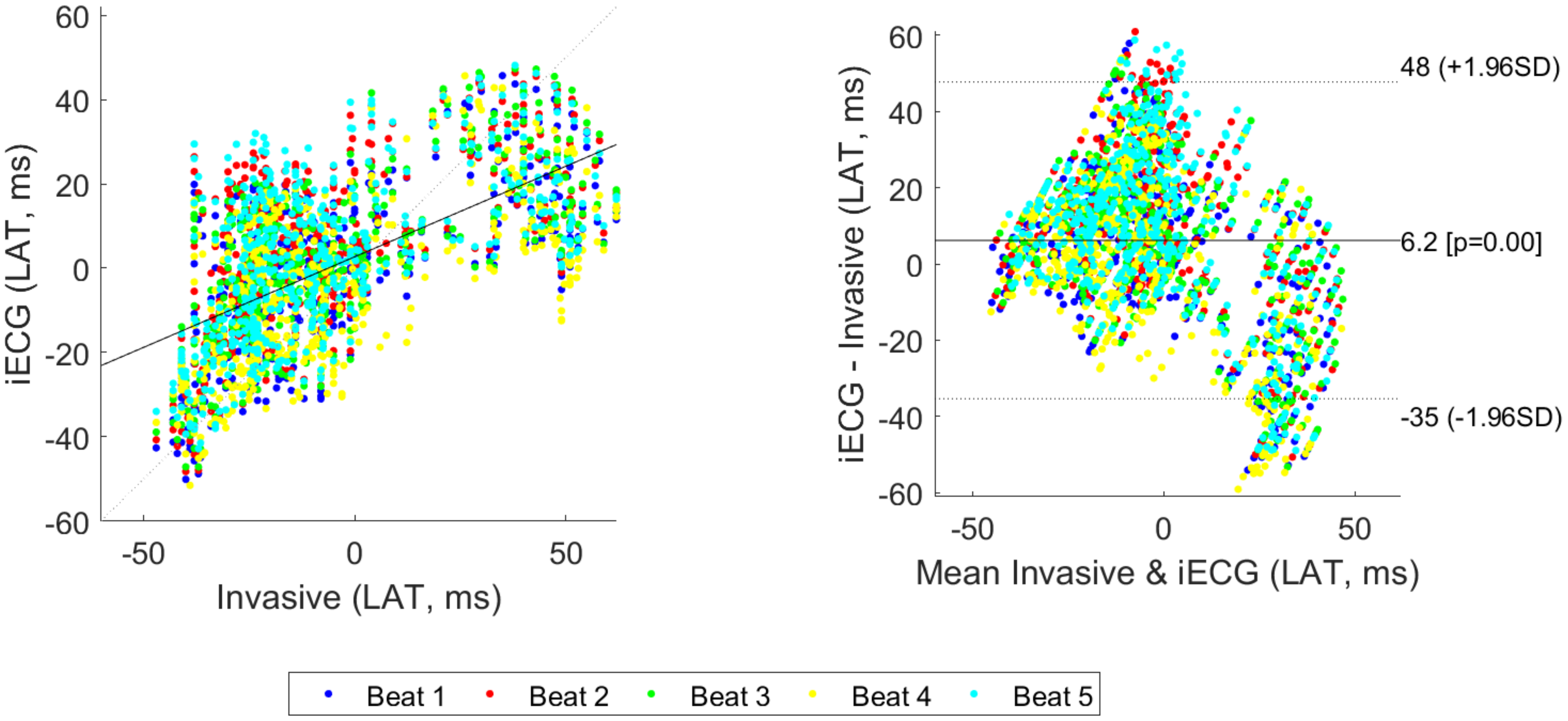

CC = 0.625; P < 0.001

ID: 12. Epicardium

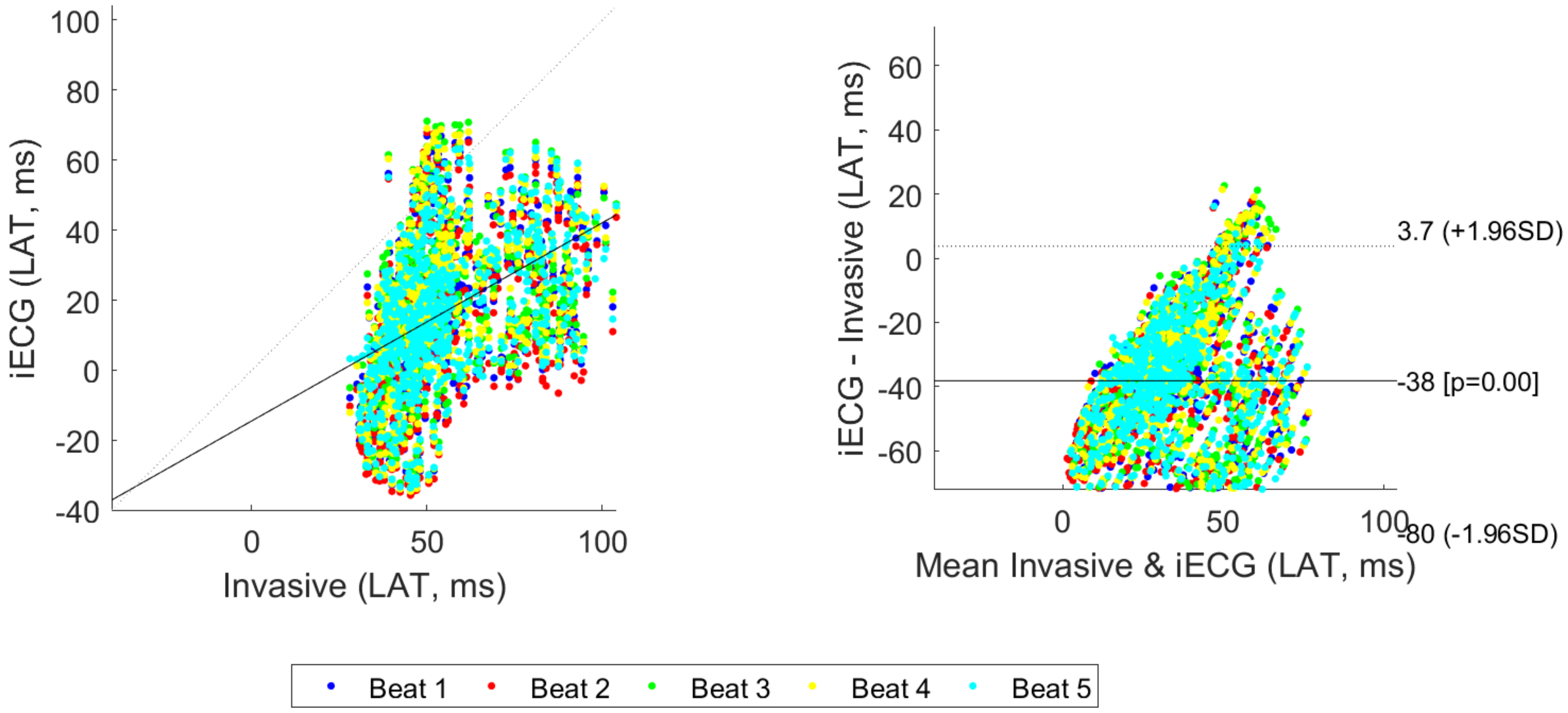

CC = 0.427; P < 0.001

ID: 12. RV Endocardium

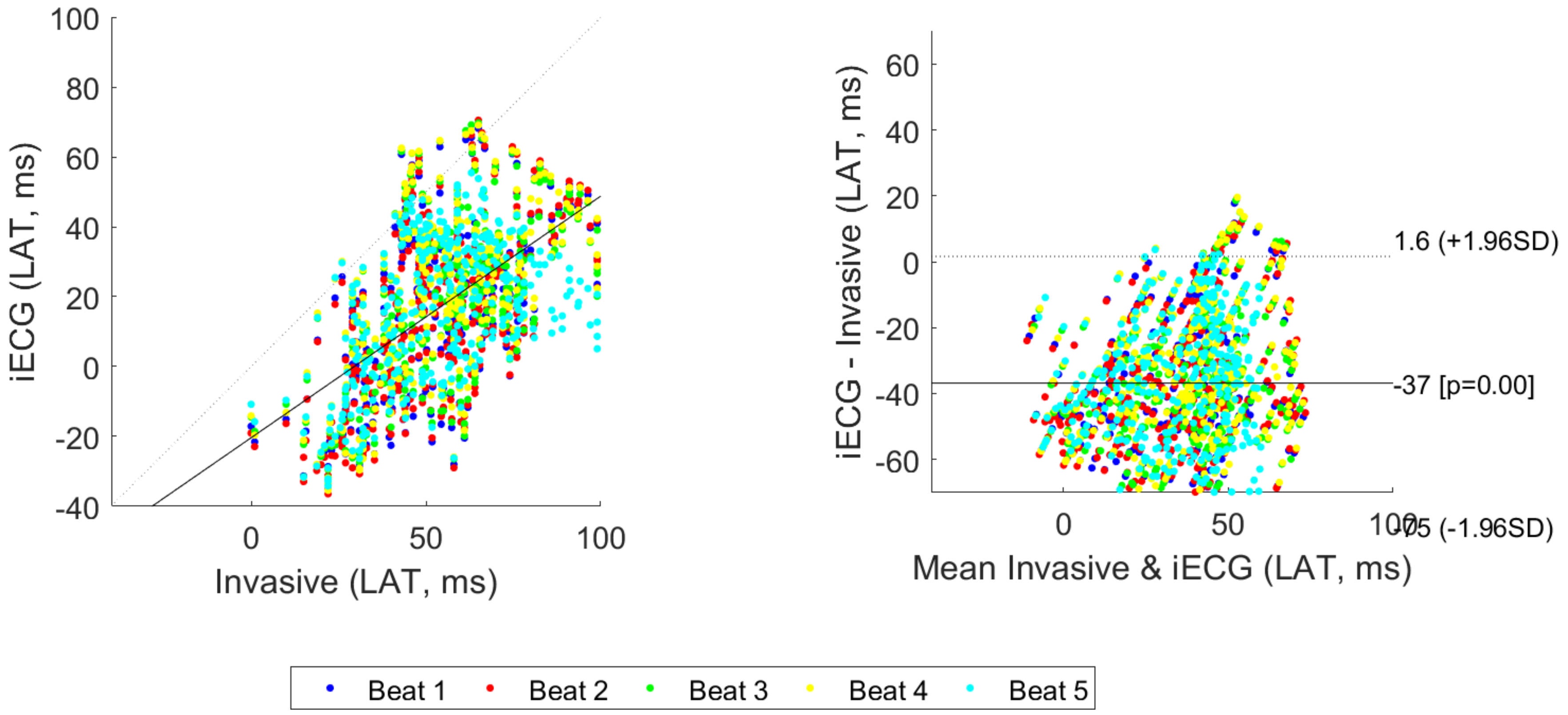

CC = 0.577; P < 0.001

ID: 13. Epicardium

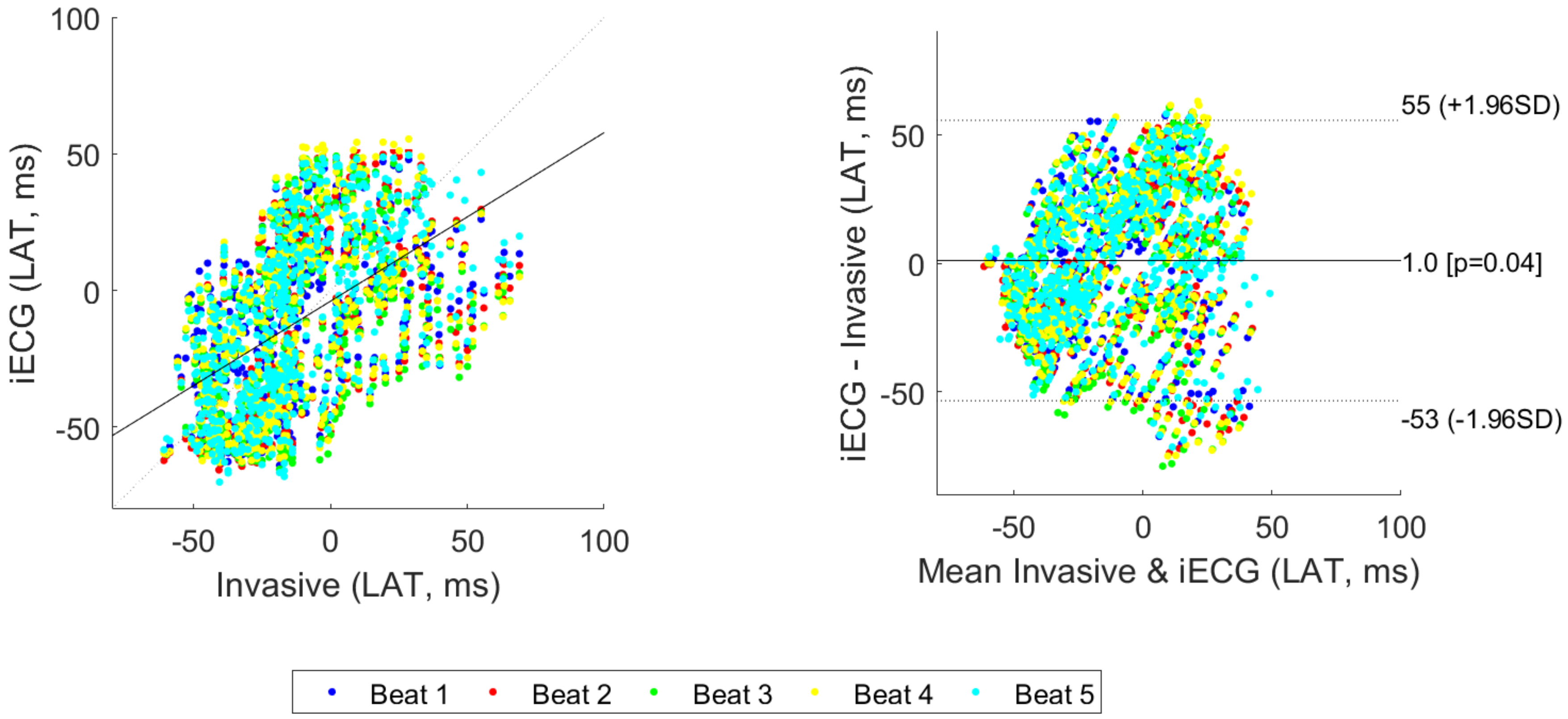

CC = 0.512; P < 0.001

ID: 13. LV Endocardium

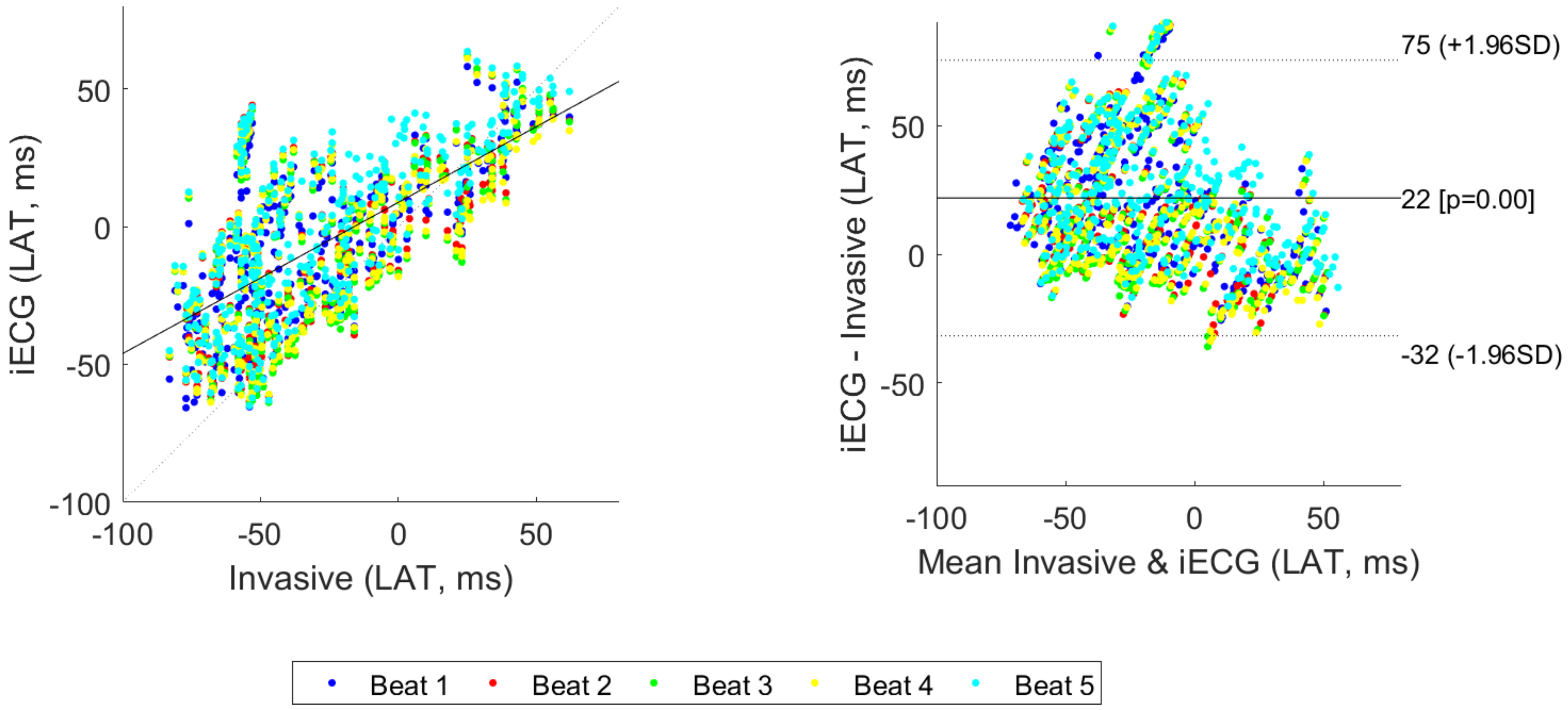

CC = 0.660; P < 0.001
